# Climate vulnerability assessment for Pacific salmon and steelhead in the California Current Large Marine Ecosystem

**DOI:** 10.1371/journal.pone.0217711

**Published:** 2019-07-24

**Authors:** Lisa G. Crozier, Michelle M. McClure, Tim Beechie, Steven J. Bograd, David A. Boughton, Mark Carr, Thomas D. Cooney, Jason B. Dunham, Correigh M. Greene, Melissa A. Haltuch, Elliott L. Hazen, Damon M. Holzer, David D. Huff, Rachel C. Johnson, Chris E. Jordan, Isaac C. Kaplan, Steven T. Lindley, Nathan J. Mantua, Peter B. Moyle, James M. Myers, Mark W. Nelson, Brian C. Spence, Laurie A. Weitkamp, Thomas H. Williams, Ellen Willis-Norton

**Affiliations:** 1 Northwest Fisheries Science Center, National Marine Fisheries Service, National Oceanic and Atmospheric Administration, Seattle, Washington, United States of America; 2 Southwest Fisheries Science Center, National Marine Fisheries Service, National Oceanic and Atmospheric Administration, Monterey, California, United States of America; 3 Southwest Fisheries Science Center, National Marine Fisheries Service, National Oceanic and Atmospheric Administration, Santa Cruz, California, United States of America; 4 Department of Ecology and Evolutionary Biology, University of California, Santa Cruz, California, United States of America; 5 Forest & Rangeland Ecosystem Science Center, U.S. Geological Survey, Corvallis, Oregon, United States of America; 6 Center for Watershed Sciences, University of California, Davis, California, United States of America; 7 Department of Wildlife, Fish and Conservation Biology, University of California, Davis, California, United States of America; 8 ECS Federal, Inc. Under Contract to Office of Sustainable Fisheries, National Marine Fisheries Service, National Oceanic and Atmospheric Administration, Silver Spring, Maryland, United States of America; Universidade de Aveiro, PORTUGAL

## Abstract

Major ecological realignments are already occurring in response to climate change. To be successful, conservation strategies now need to account for geographical patterns in traits sensitive to climate change, as well as climate threats to species-level diversity. As part of an effort to provide such information, we conducted a climate vulnerability assessment that included all anadromous Pacific salmon and steelhead (*Oncorhynchus* spp.) population units listed under the U.S. Endangered Species Act. Using an expert-based scoring system, we ranked 20 attributes for the 28 listed units and 5 additional units. Attributes captured biological sensitivity, or the strength of linkages between each listing unit and the present climate; climate exposure, or the magnitude of projected change in local environmental conditions; and adaptive capacity, or the ability to modify phenotypes to cope with new climatic conditions. Each listing unit was then assigned one of four vulnerability categories. Units ranked most vulnerable overall were Chinook (*O*. *tshawytscha*) in the California Central Valley, coho (*O*. *kisutch*) in California and southern Oregon, sockeye (*O*. *nerka*) in the Snake River Basin, and spring-run Chinook in the interior Columbia and Willamette River Basins. We identified units with similar vulnerability profiles using a hierarchical cluster analysis. Life history characteristics, especially freshwater and estuary residence times, interplayed with gradations in exposure from south to north and from coastal to interior regions to generate landscape-level patterns within each species. Nearly all listing units faced high exposures to projected increases in stream temperature, sea surface temperature, and ocean acidification, but other aspects of exposure peaked in particular regions. Anthropogenic factors, especially migration barriers, habitat degradation, and hatchery influence, have reduced the adaptive capacity of most steelhead and salmon populations. Enhancing adaptive capacity is essential to mitigate for the increasing threat of climate change. Collectively, these results provide a framework to support recovery planning that considers climate impacts on the majority of West Coast anadromous salmonids.

## Introduction

Anthropogenic climate change poses a direct threat to existing global biodiversity. In fact, climate-related population extinctions have already occurred in 47% of 976 plant and animal species surveyed in a recent review of the literature [1]. Moreover, local extinction percentages are higher in freshwater (74%) than in terrestrial (46%) or marine habitats (51%) [1]. Such impacts are expected to increase in the future [2–4], and managers are actively seeking information regarding the species or populations most vulnerable to climate change. Information of this kind is needed to prioritize resources for restoration and climate adaptation efforts. Climate vulnerability assessments are an important tool in these efforts because they provide systematic summaries of the relative threat level to a set of species or populations [5–7].

We conducted a comprehensive climate vulnerability assessment for Pacific salmon and steelhead (*Oncorhynchus* spp.) in the U.S. portion of the California Current Large Marine Ecosystem (CCLME) and associated watersheds. Partly as a consequence of natal homing to diverse watersheds, Pacific salmon display significant life history diversity evolved through local adaptation and limited dispersal [8]. In considering the conservation importance of this diversity, NOAA Fisheries applied the concept of evolutionarily significant units [9] to define 52 distinct population segments (DPSs) of Pacific salmon that could potentially be protected under the US Endangered Species Act (ESA). Our analysis focuses primarily on those DPSs that have been identified as species of concern, threatened or endangered (31/52). We also included one chum (*O*. *keta*) and one pink (*O*. *gorbuscha*) non-listed DPS to represent these species, which have few or no listed DPSs. In total we compared the relative vulnerability of 33 *Oncorhynchus* DPSs in the CCLME.

Our assessment was based on three components of vulnerability: 1) biological sensitivity, which is a function of individual species characteristics; 2) climate exposure, which is a function of geographical location and projected future climate conditions; and 3) adaptive capacity, which describes the ability of a DPS to adapt to rapidly changing environmental conditions [10]. Objectives were to characterize the relative degree of threat posed by each component of vulnerability across DPSs and to describe landscape-level patterns in specific threats and cumulative vulnerability at the DPS level.

### Species units, spatial domains, and life histories

**Fig 1:**
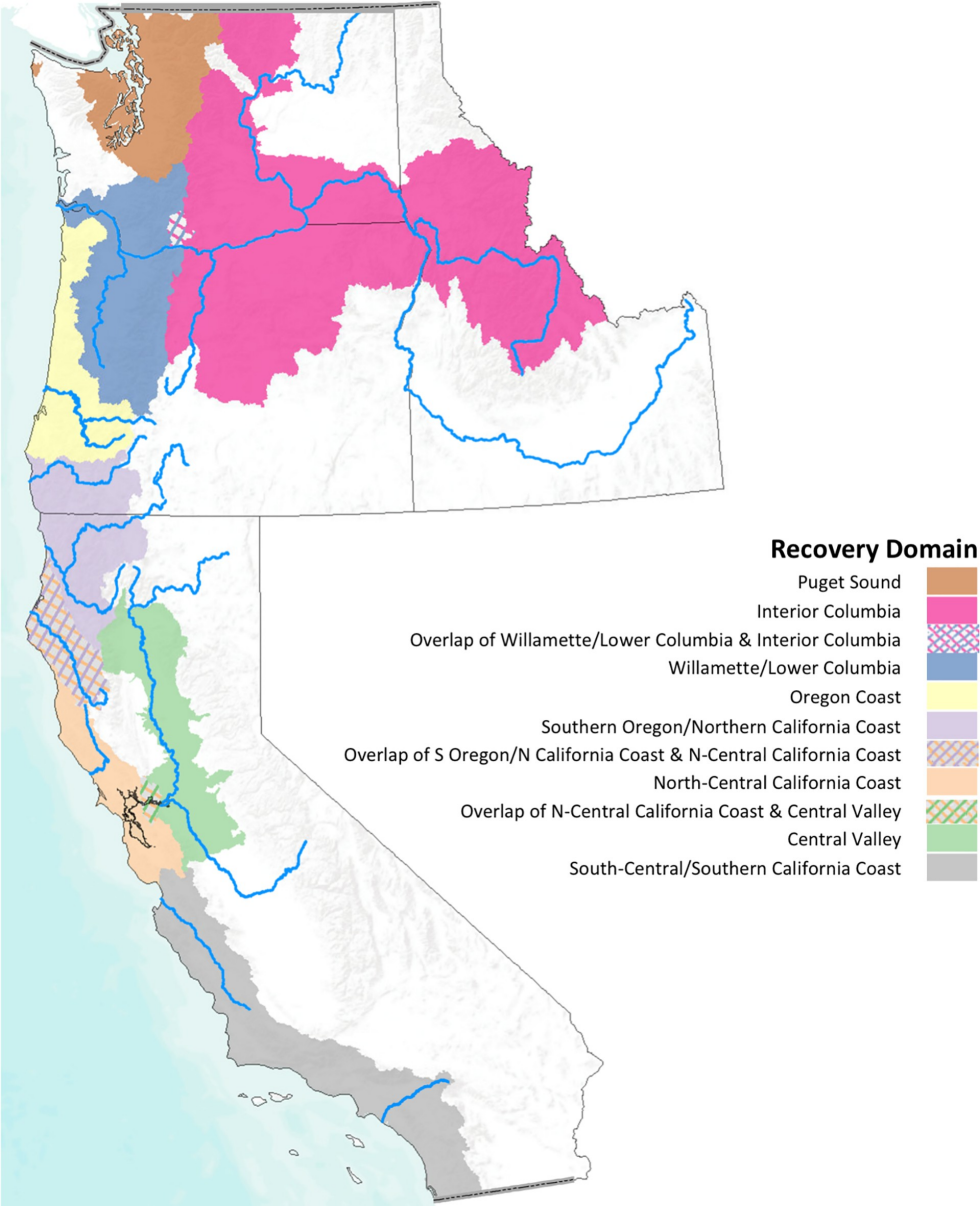
Salmon recovery domains. We analyzed patterns in vulnerability across DPSs within geographic recovery domains used to manage West Coast salmon and steelhead populations listed under the ESA [12]. The DPSs inhabiting each domain are listed in Table 1.

Pacific salmon are native to coastal regions of northeastern Asia (Japan, Korea and Russia) and western North America from California to Alaska. Of the seven species of *Oncorhynchus* [11] within the CCLME, we included the six that have primarily anadromous life histories: climate change will profoundly impact both the freshwater and marine life stages for these species (Table 1). A seventh species, cutthroat trout (*O*. *clarkii*) has an anadromous component, but is generally considered an inland species. Among the six species included in our analysis, there are 52 DPSs occupying eight recovery domains (Fig 1), or ecoregions with distinct climatic and ecological characteristics.

**Table 1:** Salmon and steelhead distinct population segments (DPSs) included in the assessment. Species names are shown with number of DPSs in parenthesis. Name, listing status, and recovery domain is also shown for each DPS.

Species/distinct population segment name		Listing status	Recovery domain
**Steelhead *O*.* mykiss* (11)**			
	Northern California steelhead	*Threatened*	North-Central California Coast
	California Central Valley steelhead	*Threatened*	Central Valley
	Central California Coast steelhead	*Threatened*	North-Central California Coast
	South-Central California Coast steelhead	*Threatened*	S-Central/Southern CA Coast
	Southern California Coast steelhead	*Endangered*	S-Central/Southern CA Coast
	Puget Sound steelhead	*Threatened*	Puget Sound
	Upper Columbia River steelhead	*Threatened*	Interior Columbia
	Snake River Basin steelhead	*Threatened*	Interior Columbia
	Middle Columbia River steelhead	*Threatened*	Interior Columbia
	Upper Willamette River steelhead	*Threatened*	Willamette/-Lower Columbia
	Lower Columbia River steelhead	*Threatened*	Willamette/-Lower Columbia
**Chinook salmon *O*.* tshawytscha* (11)**			
	Lower Columbia River Chinook	*Threatened*	Willamette/-Lower Columbia
	Upper Willamette River Chinook	*Threatened*	Willamette/-Lower Columbia
	Puget Sound Chinook	*Threatened*	Puget Sound
	Snake River fall-run Chinook	*Threatened*	Interior Columbia
	Snake River spring/summer-run Chinook	*Threatened*	Interior Columbia
	Middle Columbia River spring-run Chinook	Sensitive*	Interior Columbia
	Upper Columbia River spring-run Chinook	*Endangered*	Interior Columbia
	Central Valley fall/late fall-run Chinook	Species of concern	Central Valley
	Central Valley spring-run Chinook	*Threatened*	Central Valley
	Sacramento River winter-run Chinook	*Endangered*	Central Valley
	California Coastal Chinook	*Threatened*	North-Central California Coast
**Coho salmon *O*.* kisutch* (5)**			
	Central California Coast coho	*Endangered*	North-Central California Coast
	Southern Oregon/Northern California Coast coho	*Threatened*	Southern Oregon/Northern CA Coast
	Oregon Coast coho	*Threatened*	Oregon Coast
	Lower Columbia River coho	*Threatened*	Willamette/Lower Columbia
	Puget Sound coho	Species of concern	Puget Sound
**Chum salmon *O*.* keta* (3)**			
	Columbia River chum	*Threatened*	Willamette/Lower Columbia
	Puget Sound chum	Not listed	Puget Sound
	Hood Canal summer-run chum	*Threatened*	Puget Sound
**Sockeye salmon *O*.* nerka* (2)**			
	Lake Ozette sockeye	*Threatened*	Puget Sound
	Snake River sockeye	*Endangered*	Interior Columbia
**Pink salmon *O*.* gorbuscha* (1)**			
	Odd-year pink	Not listed	Puget Sound

* Middle Columbia spring-run Chinook are identified as sensitive by Oregon

At present, more than half of all anadromous Pacific salmon and steelhead DPSs remaining in the contiguous U.S. are threatened with extinction [13]. Suboptimal climate conditions within the historical range of climate variability have been associated with detectable declines in many of these DPSs, highlighting their sensitivities to climatic drivers [14–17]. In some cases, the synergistic effects of suboptimal climate conditions and intense anthropogenic stressors precipitated the population declines that led to these listing decisions.

**Fig 2:**
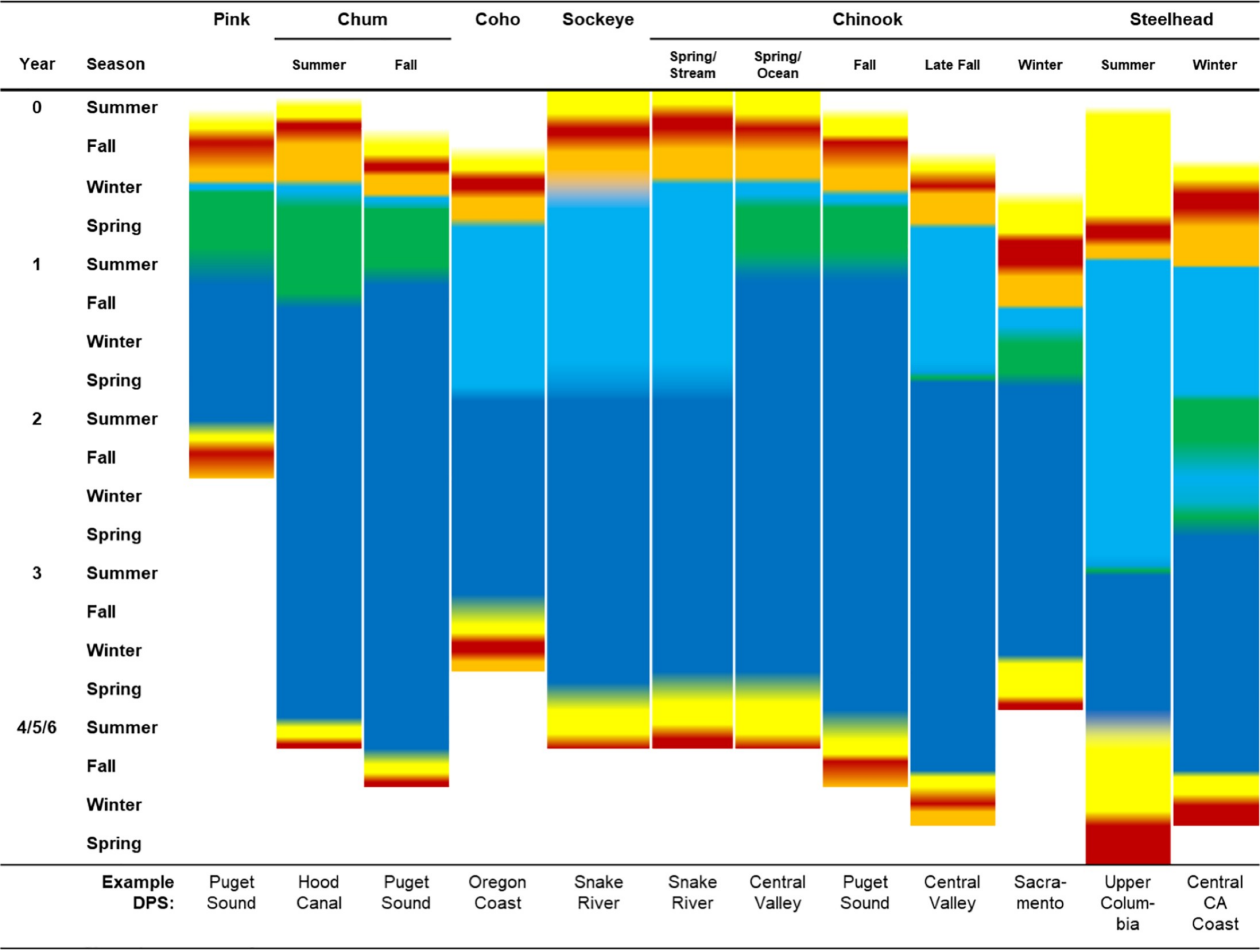
Schematic of Pacific salmon life histories for example ecotypes. Colors represent life stages, where yellow indicates adult freshwater migration and holding, red spawning, orange incubation, light blue juvenile freshwater rearing and migration, green estuary and nearshore rearing, and dark blue marine stage.

There is tremendous life history diversity among and within Pacific salmon species (Fig 2) [18, 19]. Anadromous species hatch in freshwater, migrate to the ocean to feed and grow, and return to freshwater to spawn. Most adults die after spawning, although some steelhead (*O*. *mykiss*) spawn successfully in multiple years. Juveniles can remain in freshwater anywhere from days to years, with populations that spawn near the ocean typically having shorter freshwater phases [20].

The seasonal timing of the juvenile and adult migrations varies across DPSs and species, as does the extent to which this variation is associated with genetic differentiation [21, 22]. Within the CCLME, Chinook salmon (*O*. *tshawytscha*) and steelhead exhibit the greatest life-stage variability. For example, some Chinook juveniles spend a full year in freshwater before migrating as yearlings, whereas others enter the marine environment as subyearlings. Adults of different life history types enter freshwater to commence the spawning migration in spring, summer, fall, or winter, with maturation either in the ocean or in freshwater.

Salmon life histories are highly variable within the marine stage as well. In the CCLME, pink salmon (*O*. *gorbuscha*) characteristically spend 1.5 years at sea, while coho (*O*. *kisutch*), chum (*O*. *keta*), Chinook, sockeye (*O*. *nerka*), and steelhead mature at various ages, with some males and hatchery offspring returning to freshwater within 1 year. Typically, adult coho return after 1.5 years at sea, whereas the other salmon species spend 2-5 years in the ocean. *O*. *mykiss*, *O*. *nerka*, and to a lesser extent other species have some populations or portions of populations that forego the marine migration altogether. Freshwater-resident populations, most notably rainbow trout (*O*. *mykiss*) and kokanee (*O*. *nerka*) are generally not included in DPSs.

Anadromous salmonids exhibit a high degree of homing fidelity during the adult migration, which fosters local adaptation to conditions in a particular watershed. Differences in behavior, body shape, thermal tolerance, and disease tolerance reflect genetic adaptations to characteristic patterns in temperature and stream flow (e.g., [23–26]). Differences in environmental cues, habitat conditions, and growth rates also trigger extensive life history variation among and within populations, even in the absence of genetic differentiation (e.g., [27–29]). Many traits appear to have responded to recent climate change, apparently without genetic adaptation [30]. However, to keep pace with climate change, genetic adaptation may be necessary in the long-run [31–33]; thus, maintaining genetic diversity within DPSs and species as a whole is a high priority for salmon conservation [34].

## Methods

Our approach followed the climate vulnerability assessment method developed by Hare et al. [35], which is now being implemented for U.S. marine and anadromous species by NOAA Fisheries [36]. This method was designed for rapid assessment across a wide variety of taxa using available qualitative and quantitative data. It assumes that vulnerability will be periodically re-assessed, and methods refined as status reviews are updated and more information can be considered for individual DPSs.

Hare et al. [35] had four components in their analysis: exposure, sensitivity, probability of directional shift, and net direction of climate effects. They used exposure and sensitivity attributes to calculate total vulnerability, while range shift and net climate effect scores provided supplemental information. Hare et al. [35] intentionally incorporated adaptive capacity attributes into the sensitivity component. Nonetheless, they emphasized specific elements of adaptive capacity that had special relevance by reporting separate scores for range shift and net climate effect.

We used the same analytical structure as Hare et al. [35], but with specific attributes tailored to account for characteristics most relevant to individual life stages and habitats used by salmon and steelhead. This approach allowed us to capture within-species differences at the DPS level, or scale of management units presently used in salmon recovery planning.

We differentiated DPSs by exposure and sensitivity metrics applied to each life stage. Exposure attributes summarized the magnitude of change expected in climate variables with the potential to affect species productivity in a specific region. Sensitivity attributes were based on life history characteristics and proximity to climate thresholds (i.e., sensitivity to climate limits was not assumed to be linear), as well as attributes associated with adaptive capacity, such as population size and artificial breeding programs. We also developed a separate attribute for adaptive capacity that focused on the perceived likelihood of a phenotypic change that could mitigate the effects of climate change. This separate adaptive capacity score was not included in vulnerability ranks but provided additional information useful for conservation planning.

In the following sections, we describe the specific DPSs included in this assessment and the attributes evaluated to determine relative vulnerability. We then detail the process of collecting information on attributes, scoring each attribute, and ranking DPSs into low, moderate, high, and very high vulnerability categories. Finally, we describe additional analyses performed to identify key drivers of climate vulnerability and their likely consequences for species-level diversity if the most vulnerable DPSs are lost.

### Spatial and biological scope

Our assessment included all 28 ESA-listed DPSs of Pacific salmon and steelhead in seven recovery domains (Table 1). Five of these DPSs are listed as endangered and 23 listed as threatened [14, 16]. Two additional Chinook and one coho salmon DPSs are considered species of concern by NOAA or sensitive species by state agencies. We also included Puget Sound pink salmon, which combines even-year and odd-year DPSs, because no pink salmon are listed under the ESA. We also analyzed Puget Sound chum salmon because this species has only 2 listed DPSs. Most remaining non-listed DPSs either lacked specific information that could cause their score to differ from that of a neighboring DPS (e.g., Washington Coast Chinook salmon), or were hatchery-dominated to an extent that the effects of climate change will depend more on hatchery management than on the attributes included in our analysis. While hatchery management will also need to adjust to climate change [37], different metrics than those evaluated here are needed to characterize vulnerability in hatchery stocks.

### Sensitivity attributes

Attributes describing biological sensitivity to climate change included life-stage specific metrics that largely reflect the intrinsic biological characteristics and geographic range of each DPS. These attributes also included population-level stressors that reduce natural resilience. All biological sensitivity attributes except *sensitivity to ocean acidification* were modified from species-level assessments [35], which would have produced the same score for all DPSs. Note that we included *exposure to ocean acidification* as a separate attribute to characterize the amount of physical change expected in the CCLME. Salmon generally occupy tributary, mainstem, estuary, and marine habitats sequentially over their life cycle. Therefore, in assessing climate vulnerability, it is important to consider sensitivity at each life stage and corresponding habitat. To standardize scoring across DPSs, we developed a rubric for each sensitivity attribute (Table 2, S1 Appendix).

**Table 2:** Overview of sensitivity and exposure attributes. We developed a rubric for each sensitivity and exposure attribute to standardize scoring across DPSs. We included four freshwater and five marine exposure attributes, each considered within the habitat of the respective DPS and life stage. Full descriptions of scoring criteria are included in the S1 and S2 Appendices.

Attributes	Low vulnerability	Very high vulnerability
**Sensitivity**		
**Early life history**	Minimal flow & temperature stress in egg/early fry stage	Flow or temperature stress already apparent
**Juvenile freshwater stage**	Flexible subyearling migration strategy	Constrained yearling or stressed subyearling migratory strategy
**Estuary stage**	Short estuarine residence or wide window for migration timing	Long estuary residence or climate-related threats in the estuary stage already apparent
**Marine stage**	Low correlation between marine survival & climate indicators, overlapping cohorts with variable age at return	High correlation between marine survival & climate indicators; simple age structure
**Adult freshwater stage**	Adult migration distance & duration short; low climate stress during migration, holding & spawning	Adults encounter peak summer temperatures or flow constraints during migration, holding, or spawning
**Cumulative life-cycle effects**	Low risk of loss for defining characteristic of DPS or link between life stages	Imminent climate threshold or life history type already at risk
**Hatchery influence**	No hatchery-origin populations released within DPS boundaries	Production hatcheries dominate naturally spawning populations
**Other stressors**	Non-climate threats are relatively minor	Multiple threat categories severe relative to other DPSs
**Population viability**	Extinction risk low based on viable salmon population criteria	Extinction risk high based on viable salmon population criteria
**Ocean acidification sensitivity**	Non-specialist on prey highly sensitive to ocean acidification	The DPS is a sensitive taxon, see text
**Freshwater exposure**		
**Stream temperature**	Z-score in August mean stream temperature of spawning, rearing, and migration habitats < 0.5	Z-score for August mean stream temperature exceeds 2
**Summer water deficit**	Z-score for water balance in summer freshwater habitat < 0.5	Z-score for water deficit exceeds 2
**Flooding**	Relatively small change projected or freshwater habitat not influenced by floods	Large change in flood events with potentially severe habitat effects expected
**Hydrologic regime**	Expected regime change in < 5% of spawning area	Expected regime change in > 25% of spawning area
**Marine exposure**		
**Sea level rise**	Sea level rise minimal (projection range includes 0)	Sea level rise > global average
**Sea surface temperature**	Z-score in the ocean migration area < 0.5	Z-score in the ocean habitat exceeds 2
**Ocean acidification exposure**	Z-score for pH in ocean range < 0.5	Z-score for ocean pH exceeds 2
**Upwelling**	Little projected change in intensity or phenology of upwelling-favorable winds	Significant projected change in intensity or phenology of upwelling-favorable winds
**Ocean currents**	Large-scale ocean circulation patterns affecting the northern CCLME are projected to change relatively little	Major changes in ocean circulation are projected

#### Life-stage sensitivity

We calculated habitat-specific sensitivity scores associated with five stages of the salmon life cycle. These stages are seasonally and spatially defined, so the particular habitats occupied in each life stage are potentially affected by different exposure attributes. The ***early life history*** stage included egg incubation and fry emergence; the ***juvenile freshwate****r* ***stage*** encompassed the fry-to-smolt transition; and the ***estuary*** and ***marine stages*** were distinguished physically by location. The ***adult freshwater stage*** included freshwater entry, migration, holding, and spawning.

For each life stage, biological sensitivity was scored from low to very high based on the extent of present climate stress within the DPS habitat (S1–S8 Figs) and on the level of habitat and behavioral diversity within the DPS. For a given life stage and DPS, sensitivity was ranked very high if high mortality had been directly linked to a climate driver in recent history. For example, Sacramento River winter-run Chinook recently experienced high rates of egg mortality due to warm water temperatures [38]. If behavioral or habitat diversity allows a substantial portion of the population to avoid detrimental conditions in a given year, for example by shifted phenology or habitat selection, then sensitivity was ranked lower.

We also included an attribute for ***cumulative life-cycle effects*** to reflect the necessity of completing all stages and maintaining a life history pattern characteristic of the DPS. This attribute accounted for the possibility that individuals might avoid a climate stressor during a given life stage at a cost to subsequent stages. For example, earlier migration in the *juvenile freshwater stage* could increase survival to ocean entry but decrease survival during the *marine stage* because of smaller body size or a mismatch between prey abundance and ocean-entry timing. The *cumulative life-cycle effects* attribute also captured any expert judgment that a given life stage was at such critical risk that reduction in survival at that stage would threaten the entire life cycle or an essential characteristic of the DPS (e.g., anadromy).

#### Sensitivity to ocean acidification

Salmon ***sensitivity to ocean acidification*** most likely occurs through ecological mechanisms mediated by changes to the food web [39–41]. Taxa directly affected by declining marine pH include invertebrates such as pteropods, crabs, and krill, which play a significant role in some salmon diets [42]. Physiological effects of acidification may also impair olfaction, which could hinder homing ability [43], along with other developmental effects [44]. Using the criteria of Morrison et al. [45] for scoring, all salmon had low-to-moderate *sensitivity to ocean acidification*. Slight differences among DPSs stemmed from marine diet: sockeye, chum, and pink salmon consume more zooplankton than Chinook, coho and steelhead, which are mostly piscivorous.

#### Population viability

Scores for population viability were based on indices of extinction risk, as evaluated in recent ESA status reviews and viability assessments [14, 16]. As part of each status review, all listed salmon were formally evaluated with respect to 1) present vs. historical population abundance; 2) population growth rate; 3) spatial structure, or the distribution of populations within a DPS; and 4) genetic and phenotypic diversity [34]. For DPSs not included in status reviews, we asked experts to apply these same criteria to the greatest extent possible, given the information available. These population viability criteria were developed by NOAA Fisheries to monitor long-term evolutionary potential [34], and therefore they relate to adaptive capacity. More specifically, evolutionary potential is strongly related to genetic variability and the risk of demographic extinction, both of which are correlated with population size [33, 46] and growth rate [47].

#### Hatchery influence

Numerous hatcheries release artificially propagated juvenile salmon into freshwater, estuary, or marine habitats to supplement natural production. After completing the ocean stage, these hatchery-origin fish generally return to tributaries concurrently with natural-origin salmon. Unless they are harvested or collected for broodstock or removal, hatchery-origin fish spawn in natural habitat.

Hatcheries may have mixed effects on the resilience of natural populations to climate change. In the best-case scenario, hatcheries provide a temporary demographic buffer for catastrophic declines in abundance [48]. However, hatchery populations could eventually be more susceptible to large-scale climate forcing than natural populations due to the absence of behavioral, physiological, and genetic adaptation in the wild [15, 49]. Although some hatcheries follow careful genetic protocols to minimize loss of genetic variation, many reduce the effective size of wild populations in proportion to their relative abundance [50–52], which reduces adaptive capacity. In this assessment, we assumed that conservation hatcheries practicing best-management procedures and high-quality monitoring posed lower risks to DPSs than production hatcheries. Thus we ranked no hatchery influence as low vulnerability, influence from conservation hatcheries as moderate, and influence from production hatcheries as high or very high, depending on the proportion of natural-origin adults spawning in streams across the DPS.

#### Other stressors

Salmon populations are affected by numerous stressors not directly related to climate but that potentially reduce their ability to cope with climate change. The most common of these are habitat loss, habitat degradation, toxic chemicals, pathogens endemic to fish culture, displacement by invasive species through competition and predation, and harvest. All DPSs have experienced habitat loss and degradation along with recent changes in ecosystem composition. The highest scores for this attribute were reserved for the most severe cases, in which DPSs were subjected to a combination of multiple stressors.

Primary factors leading to past declines of wild salmon populations have included migration barriers, overfishing, habitat loss and degradation, and negative effects from hatchery production, which are captured in the *hatchery influence* attribute [53]. Although some of these stressors are now less severe than in the past (especially overfishing), they continue to affect population status in all DPSs, and are often exacerbated by climate stressors [54, 55]. We refer to *population viability*, *hatchery influence*, and *other stressors* as ***extrinsic*** factors, because they are imposed extrinsically by human activity.

### Exposure attributes

Climate exposure attributes were used to describe the magnitude of projected change in the physical environment by mid-century. Projected climate change was based on the "business-as-usual" trajectory of greenhouse gas emissions, relative concentration pathway 8.5 [2]. It is important to note that our scores for climate exposure reflected physical change relative to a historical reference period but did not assess whether these conditions were stressful for salmon. Thus, some locations that are already extremely arid or hot may be only marginally suitable for salmon, yet did not score high in the exposure component if they were not expected to change much. Proximity to environmental thresholds was captured in the biological sensitivity rather than the exposure attributes.

We included four freshwater and five marine exposure attributes (Table 2), and each attribute was considered within the habitat of the respective DPS and life stage. For freshwater attributes, we quantified the amount of change projected to occur in the spawning and rearing habitat and in migration corridors delineated for each DPS in the *StreamNet* data repository [56] (S1–S8 Figs, S1 Table). Scores for marine exposure considered the ocean migration patterns of the respective DPSs (e.g., [57–59]).

#### Freshwater attributes

Temperature and flow patterns affect all aspects of salmon behavior and physiology in freshwater, often with consequences for the marine life stage as well. Freshwater exposure attributes and scoring criteria are summarized in Table 2, and the specific rationale for each exposure metric is detailed in the S2 Appendix.

Briefly, we summarized temperature change by focusing on summer, when lethal temperatures often occur (14–25°C, depending on life stage) [60–62]. Summer low flows and drought reduce available wetted habitat and can sever connections between habitat areas, causing mortality from stranding; during these periods, water quality in remaining habitats is reduced. Low summer flows and warmer temperatures often work together in altering prey composition, riparian vegetation, and stream morphology.

Conversely, high flows can have positive or negative effects, depending on life stage, season, and watershed characteristics such as connected vs. disconnected floodplains and side channels. For example, migrating smolts generally benefit from higher flows, whereas eggs and fry exposed to higher flows can be scoured from their nests, inundated by sedimentation, or flushed out of preferred habitat especially in areas where floodplain connections have been lost or impaired. Maximum flows can result either from large precipitation events, melting of accumulated snow, or a combination of both.

We selected four metrics to capture projected change in these environmental drivers: August mean *stream temperature*, mean *summer water deficit*, extreme precipitation or *flooding* events, and change in *hydrologic regime*, which is determined by the ratio of rain to snow in winter precipitation.

To describe change in summer stream conditions, we used modeled *stream temperatures* [63, 64] and the evapotranspiration differential (potential minus actual), also known as the *summer water deficit* [65]. The latter attribute served as a proxy for low flows and drought stress on riparian vegetation. For both attributes, conditions within the spawning and rearing habitat projected for 2030–2059 were standardized statistically (*z*-score transformed) using means and standard deviations of the reference periods (1993–2011 for *stream temperature* and 1916–2006 for *summer water deficit*). The reference period for *stream temperature* is much more recent than the other metrics simply because long-term historical records are extremely rare. However, given the strong correlation between stream and air temperature [63] and the trends in longer historical records of air temperature, a longer reference period would only have increased the projected change. Because the standardized rates of change for this metric were often in our highest category (z > 2), a longer reference period would likely have had a minimal impact on our conclusions.

Changes in the magnitude of peak flows have been modeled directly for most northern streams [66], but were evaluated by proxy in California and coastal Oregon based on changes in the frequency and intensity of heavy rain events [67]. In western North America, the most extreme rain events stem from narrow corridors of water vapor called atmospheric rivers, which carry moisture over thousands of kilometers of ocean from the tropical mid-Pacific. We focused on changes in extreme events, represented by the 99th percentile in precipitation or *flooding*. Both analyses used 1970–1999 as the historical period, but the projection period for atmospheric rivers was 2070–2099, whereas the projection period for *flooding* was 2040–2069. Changes in extreme events were not amenable to *z*-score transformation; hence peak flow exposures were left as a raw percent change for experts to rank from low to very high.

*Hydrologic regime* reflects the annual pattern of flows and whether they are primarily driven by rainfall, snowmelt, or groundwater. This attribute was designed to provide a holistic description of the watershed characteristics most often correlated with salmon life history traits, and hence those directly relevant to potential loss of diversity [68, 69]. Peak flows occur during fall or winter in rain-dominated basins and during spring or early summer in snow-dominated basins. Groundwater-dominated basins are relatively insensitive to either rainfall or snowmelt.

As temperatures warm, the seasonal transition from rainfall to snowfall begins later in the year, producing higher flows in early winter and shrinking cumulative snowpack. Spring/summer snowmelt is also expected to begin earlier in most basins, causing earlier and smaller spring freshets with lower minimum flows in late summer. We characterized projected change in these flow characteristics by quantifying *hydrologic regime* change in areas within and upstream from spawning and rearing habitat. For scoring, we used threshold criteria defined by Hamlet et al. [70] and most recently modeled by Littell et al. [65]. Any change from snow-dominant to transitional or from transitional to rain-dominant regimes increased the *hydrologic regime* score.

#### Marine attributes

Ocean conditions are a major driver of salmon abundance. Marine survival tends to be correlated across stocks and species in the northeast Pacific, generally following patterns in *sea surface temperature* (SST) and large-scale climate indices [71–73]. Ocean distributions of salmon species are strongly correlated with SST [74–76], and various climate indices associated with salmon survival are related to or derived from this attribute. These include the Pacific Decadal Oscillation [77], North Pacific Gyre Oscillation, and various El Niño-Southern Oscillation indices [78]. Each of these indices reflect large-scale patterns of variation in multiple ocean characteristics such as horizontal currents, upper ocean temperature and stratification, upwelling, and vertical mixing between deep and surface waters [79–81].

In contrast, future warming trends in the north Pacific Ocean are projected to be dominated by thermal forcing associated with increased greenhouse gas concentrations and the thermodynamic feedbacks they trigger [82]. Furthermore, the relative importance of large-scale climate indices for salmon tends to change over time [78], making it difficult to determine which index will be most applicable in the future. For these reasons, we elected to focus on SST itself as the exposure factor.

In addition to climate indices, historical variations in west coast salmon marine survival have been associated with the strength of *ocean currents* that alter the proportion of prey from cold, subarctic waters [71, 72, 83–87]. *Upwelling* also impacts salmon prey composition and is a defining feature of the CCLME; thus, the strength and timing of upwelling-favorable winds was included as an exposure attribute. Upwelling intensity is tightly correlated with input and retention of cold, nutrient-rich waters to the euphotic zone, which promote high levels of primary productivity and a lipid-rich food-web in the CCLME [81, 88, 89].

*Sea level rise* was included as an exposure attribute because many salmon rear in estuaries for months before they complete the transition to marine life stages. For these fish, transitional estuarine rearing periods strongly influence later survival. Sea level rise will alter estuary and nearshore habitats, likely intensifying the impact of high tides, storms, and floods [90]. Sea level rise will also alter estuarine hydrodynamics, with additional implications for salmon habitat quality and abundance [91]. Sea level rise is associated with a net loss of estuary habitat for juvenile salmon in some assessments; [92] however, estuary dynamics are complicated, especially in terms of sand-bar formation and breaching, and we lacked detailed models with which to project these processes. We therefore differentiated DPSs by their relative rates of sea level rise at ocean entry, assuming a higher rate was more detrimental.

Finally, pH levels in the CCLME have been declining, resulting in reduced abundance and increased corrosion in the shells of calcifying organisms [93–95]. Negative effects of lower pH have been shown for many taxa in the CCLME [41], although the cumulative effects of *exposure to ocean acidification* on salmon are still uncertain.

We examined a total of five attributes reflecting ocean conditions: *sea surface temperature* (SST), *ocean acidification* (OA), *sea level rise*, timing and intensity of *upwelling*, and change in *ocean currents*. For SST and OA, we calculated standardized change in grid cells of 1° latitude by 1° longitude based on output from 27 (SST) and 11 (OA) earth system models downloaded from the NOAA Ocean Climate Change Web Portal [96]. In each grid cell, we calculated the magnitude of change as the difference between mean climate projected for 2006-2055 and mean climate from historical simulations during 1955–2005. We normalized the projected change for these exposure attributes by dividing by the historical standard deviation (*z-*score), then taking the average of *z*-scores across grid cells within the migratory range of the DPS. We calculated the mean magnitude of change at both annual and seasonal time steps to account for seasonal variation. Scorers determined the most relevant season and location for individual DPSs.

For exposure to *sea level rise*, we based scores primarily on analyses conducted by the National Research Council [97] on sea levels projected for the U.S. West Coast in the 2050s. This report projected the highest rates of sea level rise at latitudes south of Cape Mendocino, California, with slower increases at higher latitudes (scoring bins detailed in the S2 Appendix).

Projections of change in the timing and intensity of *upwelling* constitute an active area of research, but consensus across studies is weak. Our *upwelling* scores relied primarily on the analyses of Rykaczewski et al. [98]. They compiled output from 21 GCMs for the period 2071-2100 under the representative concentration pathway 8.5 scenario. They then compared projected oceanic and atmospheric metrics to those from the early industrial period of 1861-1890. Their results can be summarized as a slight “poleward shift” in the seasonal climatological cycle, wherein the average intensity of upwelling increases in the northern and decreases in the central and southern CCLME, and upwelling begins earlier in the year from central California through central Oregon.

Our S2 Appendix, *Exposure attributes*, describes the present state of the literature regarding potential change in *ocean currents* [99–102]. Ultimately, net projections were considered highly uncertain. However, our scoring method explicitly accounted for this type of uncertainty, as explained below (*Scoring process*). Reference and projection periods varied for different exposure metrics, depending on the available information. Experts used qualitative judgements ranging from low to very high to account for these differences.

### Adaptive capacity

The Intergovernmental Panel on Climate Change defined adaptive capacity as the potential for a system to respond to environmental change by genetic adaptation or by a non-genetic, phenotypic change that mitigates negative environmental impacts (Working Group II Report 2, Table 18.5 in [2]). Adaptive capacity can be characterized in various ways, including genetic richness, life history plasticity, and dispersal ability [6, 103, 104]. Additional work is needed to explore the consequences of different methods used to characterize adaptive capacity. Although differing methods can produce different rank orders in vulnerability, there is no consensus on which methodological approach can best predict responses to climate change [105–107].

Several aspects of adaptive capacity were included in our sensitivity attributes. High scores in extrinsic factors reflected lower available levels of genetic and habitat diversity to cope with climate change [108]. For example, genetic variation is reduced in small populations, simplified habitats, and populations heavily influenced by hatcheries [109, 110]. Furthermore, fish altered by artificial selection in breeding programs may introduce maladaptive genotypes into wild populations, and these may potentially swamp genotypes that have evolved through natural selection [111]. Thus the attributes of *other stressors*, *population status*, and *hatchery influence* were intended as proxies for evolutionary potential to some extent. Furthermore, the *life cycle complexity* score addressed the likelihood that a present life history would continue to be viable in future climates, and thus whether phenotypic change would be needed to cope with climate change.

In defining a separate attribute for *adaptive capacity*, we sought an index of whether change in a phenotypic trait was considered likely. For example, if a given life history trait appeared optimal in a future climate, did scorers believe the DPS was likely to change adaptively toward this trait? For this attribute, we included behavioral, physiological, and morphological traits. It was not possible to quantify the extent to which change in relevant traits would result from plastic vs. evolutionary processes because many traits were both highly plastic and heritable. Thus, an initial *adaptive capacity* trait response would likely be plastic but would be subject to selection over time.

Rates of evolutionary response depend on the full genetic architecture of all traits under selection, especially their correlation structure, temporal pattern of the selection gradient at different life stages, and existing genetic variation within the optimal phenotype [47, 112, 113]. None of these elements are known at present, making adaptive capacity scores more subjective than those of other attributes. Nonetheless, changes in key traits have important management implications, especially those that define characteristics of a DPS, such as smolt or adult run-timing, anadromy, or spatial distribution. We therefore asked scorers to evaluate the likelihood that a trait alteration could mitigate negative effects of climate change and to allocate four tallies to low, moderate, or high bins.

Movement or dispersal is an important component of adaptive capacity [104]. We focused on shifts in range or habitat usage within existing geographic boundaries and accessible habitats, although other types of range shift are possible. Salmon DPSs are defined in terms of their watershed boundaries, so dispersal of a DPS outside its existing freshwater domain would likely involve colonization of habitat occupied by another DPS, and we did not address this possibility. Shifts in salmon marine distributions have been projected based on associations with SST [74–76]; however, these projections are not available at the DPS scale. Moreover, they are based largely on Canadian and Alaskan salmon, which have migratory constraints that differ from those of DPSs included in our assessment. For these reasons, we did not attempt to quantify marine range shifts for the *adaptive capacity* attribute.

Overall, the *adaptive capacity* score was intended to capture perceived potential for behavioral, physiological, or other adaptive response to ameliorate climate stress. We assumed that experts would be familiar with a range of possible responses based on their knowledge of diversity across DPSs. Adaptive capacity scores spanned three levels (low, moderate, or high). If adaptation in a critically threatened life stage was deemed unlikely, the DPS received a low score. A moderate score indicated that some adaptive response might occur, although not in the most sensitive or exposed life stage, or that its magnitude might be fairly small. A high score indicated that some adaptive shift was likely in response to climate change. These scores were not formally integrated into relative vulnerability rankings; they provide additional information to help develop management strategies that support a range of life history expressions.

### Data quality

A crucial component of any vulnerability assessment is the quality and specificity of information on which it is based. We characterized data quality for each sensitivity and exposure attribute based on the type of data used. We scored each data quality attribute from 0, representing qualitative expert judgement alone, to 3, representing quantitative studies focused on the specific DPS being evaluated. When quantitative studies were available, data-quality scores reflected the breadth of analyses synthesized, for example, the number of GCMs included in an ensemble projection or the number of studies documenting a given relationship, as well as the extent of agreement across studies. A score of 3 indicated broad agreement over a relatively large number of GCMs or studies focused specifically on the DPS region or on populations within the DPS [45].

### Scoring process

We collected information on exposure attributes for the entire CCLME and associated watersheds and conducted a scoring workshop wherein experts discussed data-quality scores for each exposure attribute. For each sensitivity attribute, *profilers*, or scientists familiar with an individual DPS, wrote a description of each life stage, the seasonality, duration and known climate stressors at that stage, and variability within that life stage across the DPS. Behavior and habitat information has been summarized for each DPS in the NOAA Fisheries biological status reviews and their respective 5-year updates. However, this information is often focused on particular populations, tributaries, or time periods, and therefore may not necessarily represent the entire DPS [114]. Additional literature was cited in many of the DPS profiles, and profilers also assigned a data-quality score for each sensitivity attribute.

Once all of the required information was collated, a separate panel of 16 expert *scorers* rated all freshwater and marine exposure, biological sensitivity, and adaptive capacity attributes based on the guidelines summarized in Table 2 and detailed in the S1 and S2 Appendices. Each DPS was scored by four experts, with each expert scoring 5–22 salmon and steelhead DPSs, plus other species included in the West Coast Fish Climate Vulnerability Assessment. To ensure consistency across groups of DPSs, scoring groups were rearranged over several sessions. Each expert independently scored their assigned DPSs based on information contained in the profiles as well as their general knowledge, using the pre-defined scoring bins shown in Table 2 and detailed in the S1 and S2 Appendices.

Each scorer allocated five tallies across four bins (low, moderate, high, or very high) for each sensitivity and exposure attribute as described in Morrison et al. [45]. Adaptive capacity was scored by allocating four tallies across three bins (low, moderate, or high). Following preliminary scoring, all experts participated in a second workshop discussion to ensure that common definitions were applied and that all scorers were aware of DPS or location-specific factors affecting vulnerability. Final scores were then submitted.

The bins were assigned a numerical value (low = 1, moderate = 2, high = 3, very high = 4) to calculate a weighted-mean attribute score. The number of tallies in a bin served as the weighting factor. A greater spread of tallies among bins reflected greater uncertainty in scores and was captured by the standard deviation of the mean score for each attribute.

### Vulnerability categories

We calculated climate vulnerability for each DPS from its attribute scores in three steps [45]. First, we calculated the weighted mean of tallies for each sensitivity and exposure attribute. Second, we applied a logic model to determine cumulative sensitivity and exposure component ranks from their constituent attributes (Table 3). Rankings from the logic model depended on the number of attribute means that exceeded a specified threshold. For example, if at least two attributes in one component had a mean score equal to or above 3.5, that component was ranked very high.

**Table 3:** Logic rule for ranking sensitivity and exposure components and cumulative vulnerability. We used the logic rule across attributes to assign a numeric score and vulnerability category to sensitivity and exposure components (top section). We then used the product of the numeric component scores to assign cumulative vulnerability for each DPS (bottom section).

**Overall sensitivity or exposure score**	**Numeric** **score**	**Logic rule**
Very High	4	More than 3 attribute means ≥ 3.5
High	3	More than 2 attribute means ≥ 3
Moderate	2	More than 2 attribute means ≥ 2.5
Low	1	All other scores
**Cumulative** **vulnerability**	**Component product**	**Component combinations**
Very High	≥12	Very high/high or Very high/very high
High	8-11	Very high/moderate or High/high
Moderate	4-6	Very high/low, High/moderate, or Moderate/moderate
Low	≤ 3	High/low, Moderate/low, or Low/low

Sensitivity and exposure component ranks were then assigned a numerical value (very high = 4, high = 3, moderate = 2, low = 1), which was used in the final step. Overall vulnerability was determined by multiplying the numeric values for sensitivity and exposure and assigning a total score for each DPS based on the product (Table 3).

We used a bootstrap analysis to characterize uncertainty in the assignment of a climate vulnerability category [45]. The 20 tallies for each attribute (four scorers per DPS with five tallies each) were randomly sampled with replacement 1,000 times. From the resampled tallies, we calculated new climate vulnerability attribute means and final vulnerability categories using the three steps described above.

If the bootstrap outcome matched the original vulnerability category at least 75% of the time, we considered the score for that DPS to be likely. When 25% or more of the bootstrapped outcomes were either above or below the original climate vulnerability category, we considered the DPS to be borderline between the original and secondary vulnerability categories. Individual bootstrap results are shown in the S3 Appendix.

### Vulnerability profiles

To explore which attributes were most important in determining overall vulnerability and how specific threats varied across DPSs, we conducted a hierarchical cluster analysis on the full suite of mean scores for all attributes, implemented in the R “cluster” package [115]. These clusters helped visualize differences in specific threats over broad geographical and biological gradients. To group similar DPSs, we applied the Ward’s minimum variance algorithm and a Euclidean distance measure. We cut the resulting dendrogram into six groups. We then used a classification and regression tree analysis implemented in the R “tree” package [116] to identify which attributes best predicted cluster assignments.

We characterized vulnerability profiles for each cluster by computing the average score for each attribute across DPSs within each cluster. To show the general source of the threats, we grouped exposure and sensitivity attributes into four categories: freshwater exposure, marine exposure, life-stage sensitivity, and extrinsic sensitivity. We highlighted attributes with a mean cluster score of 3 or greater within each attribute category. We excluded all attributes that did not differ across DPSs, such as exposure and sensitivity to ocean acidification and ocean currents. All analyses were performed in R [117].

### Highly vulnerable life stages

The overarching principle of this vulnerability assessment is that the most vulnerable DPSs are those most sensitive to climate change *and* most exposed to changing environmental conditions [5]. We applied that same logic to determine which life stages within each DPS were most vulnerable. Because life stages are typically segregated from each other in space and time, they tend to be affected by different exposure attributes. It was thus possible to pair specific sensitivity and exposure attributes. For example, freshwater life stages occurring over fall and winter are most exposed to extreme rain events and *flooding*, whereas those occurring in summer are exposed to *stream temperature* and *summer water deficit*.

The specific attributes most relevant at each life stage varied among DPSs due to differences in life history timing (Fig 2). However, for all DPSs, *hydrologic regime* was paired with the *juvenile freshwater stage* and *sea level rise* was paired with *estuary stage*. All other marine exposure attributes were paired with the *marine stage*. To identify highly vulnerable life stages, we examined these sensitivity/exposure pairs and identified cases with scores higher than 3 in both attributes.

## Results

### Relative vulnerability

**Fig 3:**
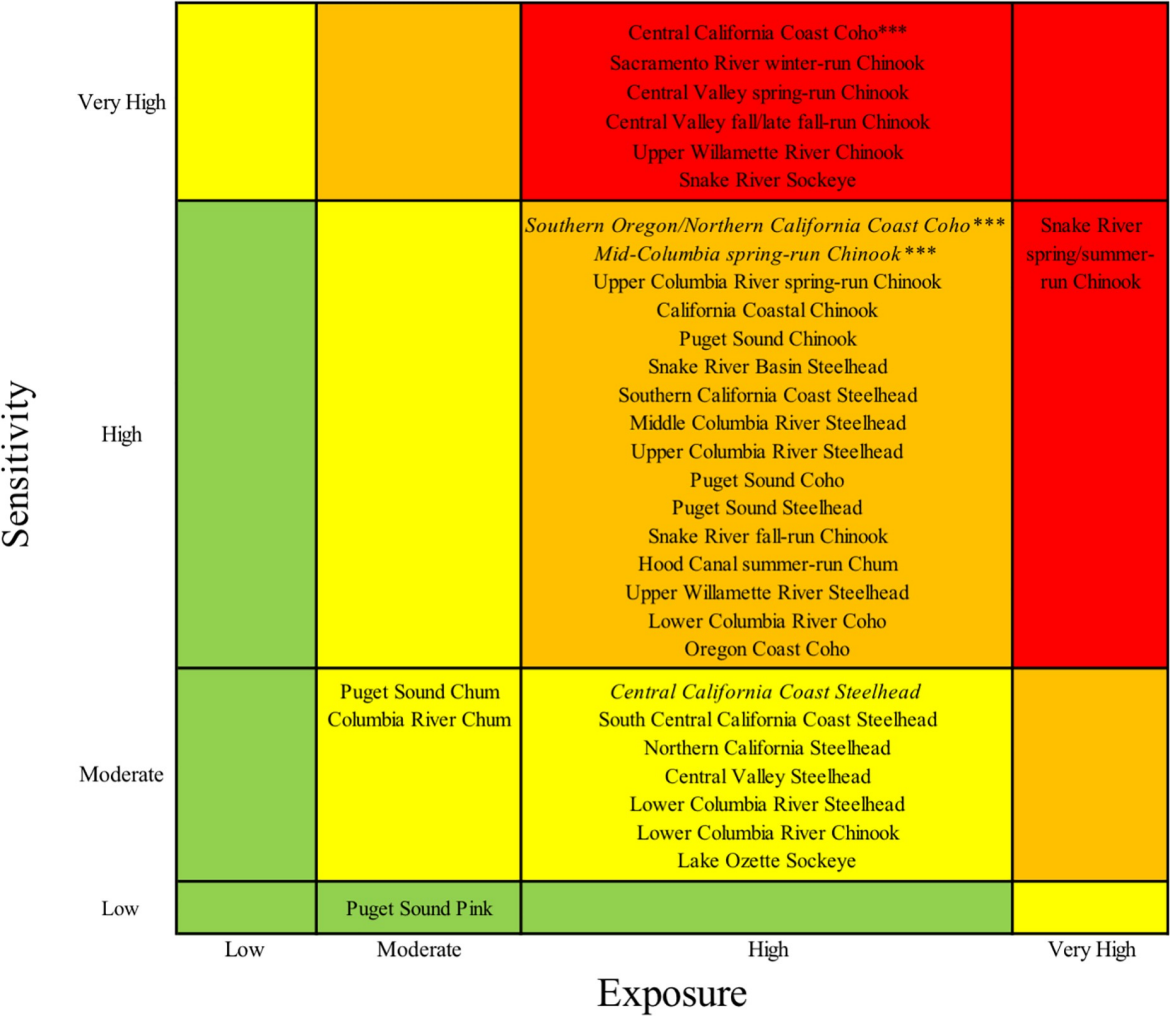
Final cumulative vulnerability ranks. Box colors show final vulnerability rank for each DPS as a product of sensitivity and exposure scores: red indicates very high vulnerability, orange high, yellow moderate, and green low. Uncertainty in final ranks was represented with a bootstrap analysis. Borderline DPSs were those that placed in a higher rank in at least 25% of resampled data. Borderline sensitivity ranks are shown in italic, and borderline exposure ranks indicated with asterisks (***). All other cumulative vulnerability ranks were considered likely.

**Fig 4:**
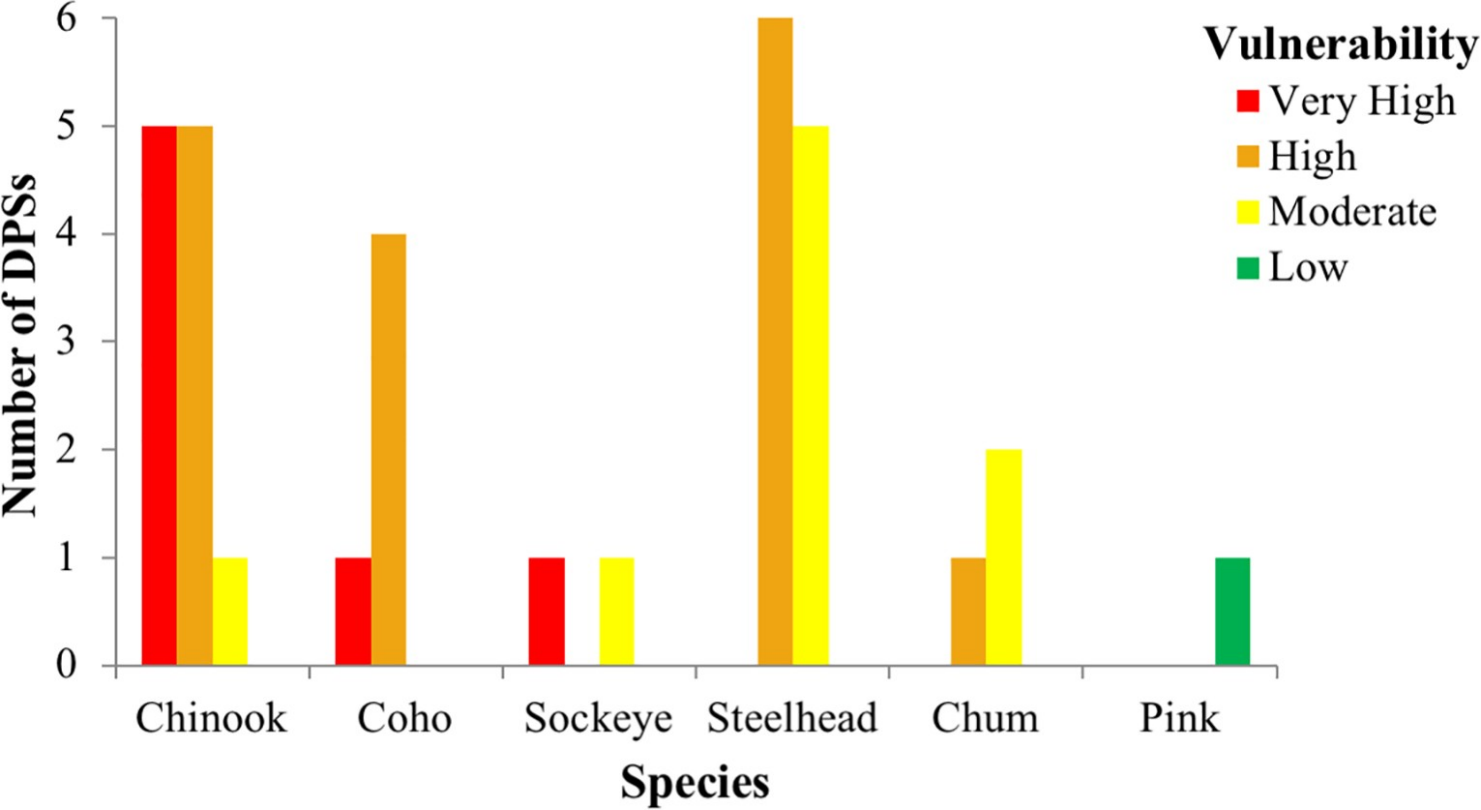
Number of DPSs in each vulnerability rank by species.

Five Chinook, one coho, and one sockeye salmon DPSs ranked very high in total vulnerability to climate change due to a combination of high and very high scores for sensitivity and exposure (Figs 3 and 4, red boxes). Bootstrap analyses indicated that two additional DPSs, Southern Oregon/Northern California Coast coho and Mid-Columbia spring-run Chinook, were borderline between high and very high (S3 Appendix). Among species, Chinook salmon had the highest vulnerability rankings overall (mostly very high and high rankings), followed by coho and sockeye (Fig 4). Steelhead and chum DPS scores were generally lower and nearly equally spread across high and moderate vulnerability categories. The only species in the low vulnerability category was pink salmon, which was represented by a single, unlisted DPS. Individual DPS scores are presented in the S2 Table and discussed in the S3 Appendix.

The preponderance of coho DPSs ranked very high in vulnerability to climate change were those occupying regions from southern Oregon to central California. Chinook and sockeye DPSs that ranked very high in vulnerability were concentrated in the two interior recovery domains: Central Valley and Interior Columbia. These results suggest that a combination of life history characteristics and geographic influences (including anthropogenic factors) contributed to high vulnerability for coho near its southern range limit and for Chinook and sockeye throughout the interior domains.

The sensitivity component spanned all vulnerability categories across DPSs and generally aligned with cumulative vulnerability ranks (Fig 3). By contrast, the exposure component of vulnerability was relatively homogeneous across DPSs: of the 33 DPSs evaluated, 29 had high exposure to climate change (Fig 3). This consistency stemmed from exposure scores that were uniformly very high for *exposure to ocean acidification* and mostly high for *sea surface temperature* and *stream temperature*.

Only pink and chum, both typically coastal species, received low or moderate scores for these temperature-related attributes. In the Interior Columbia, exposure scores for both *stream temperature* and *hydrologic regime* were near or above the threshold for very high. Sensitivity to loss of snowpack was generally higher for spring-run Chinook than for steelhead and sockeye due to differences in spawn timing and habitat, respectively. For coho, threats from exposure to *stream temperature*, *flooding*, and *sea level rise* pushed some DPS scores near the edge of the very high category.

To ensure that high scores in multiple categories did not reflect “double counting,” we assessed all pairwise correlations between attributes. Attributes that were not strongly correlated were assumed to capture different aspects of climate change, and therefore not double counting. Two pairs of attributes had a Spearman’s *rho* correlation coefficient over 0.75: *sea level rise* and *estuary stage*, and *sea surface temperature* and *flooding*. *Sea surface temperature* and *flooding* reflect independent effects of climate change and hence represented distinct impacts of concern rather than double counting. The *sea level rise* and *estuary stage* pair may reflect some shared impacts; however, the populations most dependent on *the estuary stage* are also those exposed to the highest rates of *sea level rise*. Nonetheless, we confirmed that neither of these correlations affected final vulnerability categories by removing one from each pair and recalculating vulnerability scores.

### Adaptive capacity

**Fig 5:**
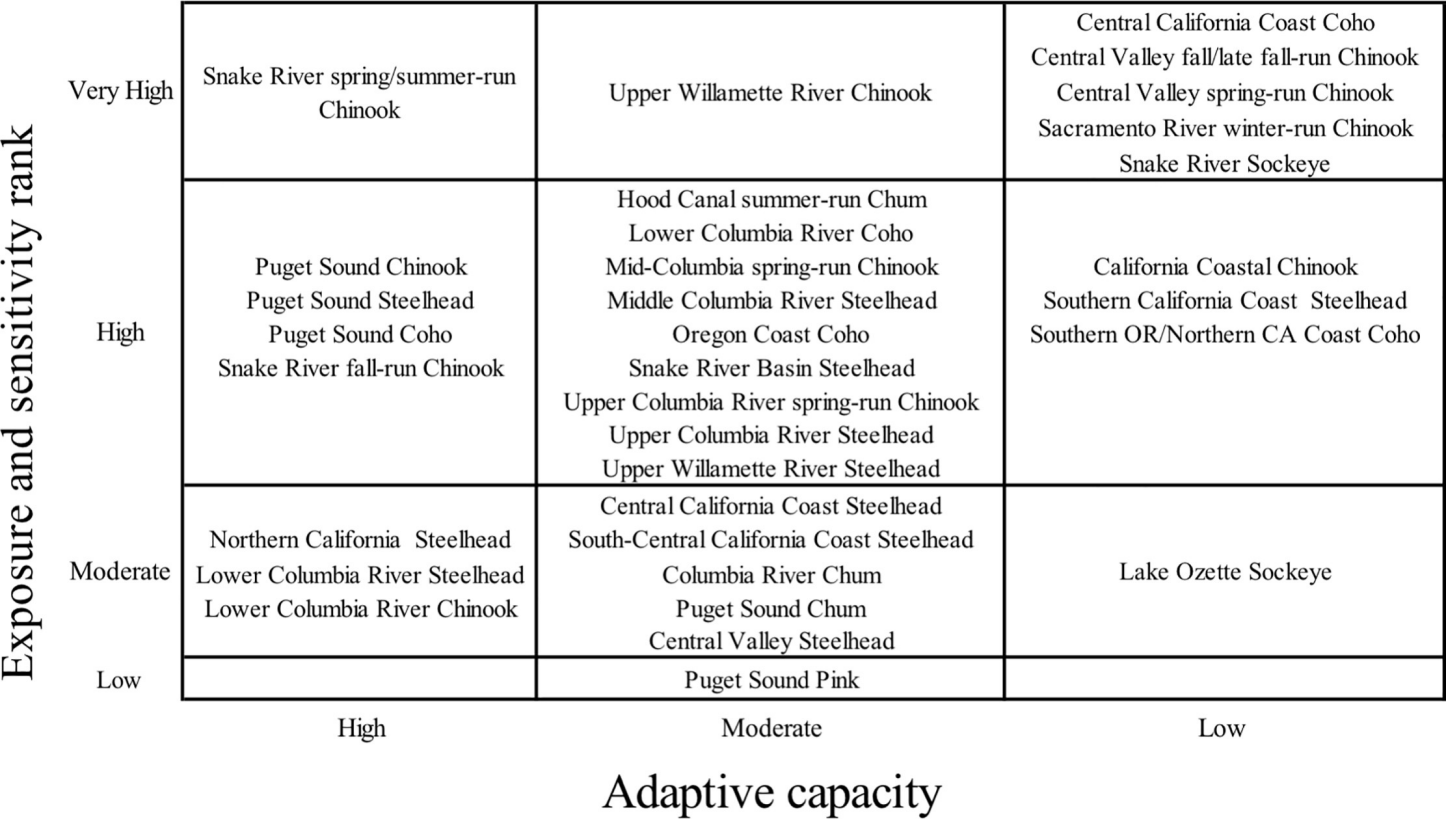
Adaptive capacity rank plotted against vulnerability rank. Vulnerability ranks were determined by exposure and sensitivity attributes (Fig 3). Adaptive capacity attribute scores reflected the opportunity perceived by scorers that some trait change would help mitigate the negative effects of climate change.

Adaptive capacity scores reflected the opportunity perceived by scorers for trait plasticity to help mitigate the negative effects of climate change (S3 Appendix). Results showed strong geographical patterns (Fig 5). All California Chinook and coho DPSs, the southernmost steelhead DPS, and both sockeye DPSs scored low in adaptive capacity. The southernmost DPSs within each species may already be near tolerance limits, but these DPSs also have some of the most severe anthropogenic impacts and therefore limited scope for potential adaptations to a warmer climate. This explanation applied to Snake River sockeye. In contrast, Lake Ozette sockeye is not climate stressed at present and was simply not expected to change phenotypically.

The DPSs that scored highest in adaptive capacity were Puget Sound Chinook, coho, and steelhead; Lower Columbia Chinook and steelhead; and Snake River spring/summer Chinook and fall Chinook. Northern California steelhead also scored high in adaptive capacity. Higher scores reflected extensive life history diversity in both juvenile and adult stages. Most high-scoring DPSs display extensive juvenile life history variation, such as the subyearling and yearling Chinook smolts, or 1- to 3-year-old steelhead smolts. Puget Sound and Lower Columbia Chinook display both spring and fall adult migration patterns, and Northern California steelhead migrate over two protracted periods, from late fall to spring for the winter-run and from spring to summer for the summer-run ecotype.

Chinook, coho, and steelhead DPSs had high variation in *adaptive capacity* scores, which ranged from low to high, whereas in other species, all DPSs fell into a single category. For example, all chum and pink DPSs scored moderate, while both sockeye DPSs scored low. There was uncertainty about whether sockeye rearing conditions would become less suitable, but the scorers' best estimate was that smolt age was unlikely to change, and any changes in adult migration timing would not substantially reduce climate stress. Selection for earlier adult run timing in Snake River sockeye could be occurring at present [31]. However, the long migration through exceptionally warm reaches of the Snake and Salmon River will likely continue to challenge this DPS. The existing population is largely supported by captive broodstock and large hatchery releases; therefore, it is not subjected to the full effects of natural selection. How this might change in the future is uncertain.

### Vulnerability profiles

**Fig 6:**
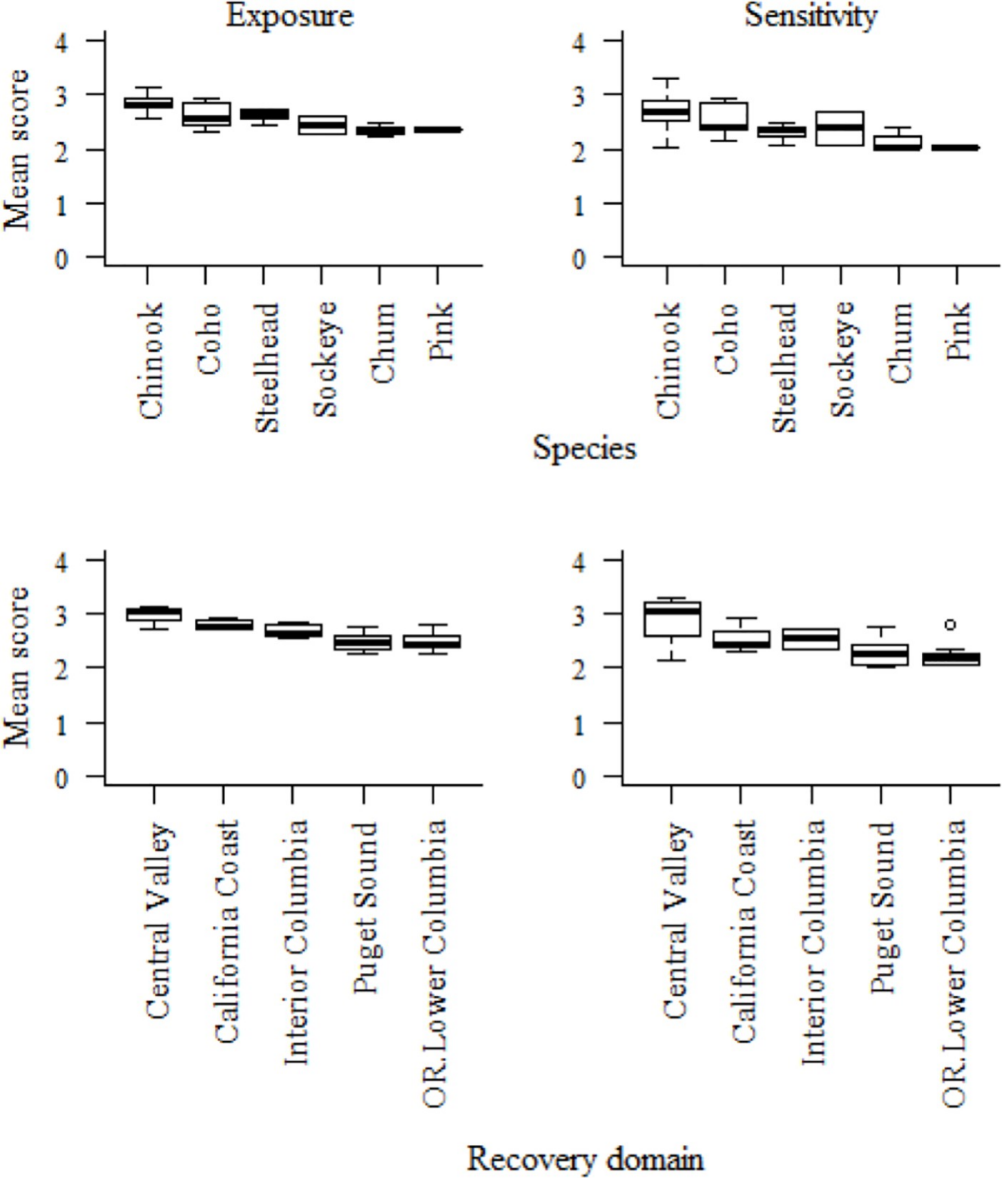
Mean exposure and sensitivity scores by species and recovery domain. Exposure scores are shown at left and sensitivity scores at right by species in upper panels and by recovery domain in lower panels. Because of the small number of DPSs in some domains, in Fig 6 the three recovery domains from southern Oregon to southern California are lumped into a California Coast group, and Oregon Coast is lumped with Lower Columbia. Boxes indicate the interquartile range of the data, whiskers show 1.5 * the interquartile range, and the black line shows the median value.

Broad geographic trends in both exposure and sensitivity attributes were seen across DPSs, owing to the large spatial scale of climate drivers (Fig 6). In both in the Central Valley and Interior Columbia domains, DPS scores trended higher in both exposure and sensitivity than corresponding scores from their respective adjacent coastal domains (Fig 6, lower panels). In coastal domains, DPSs benefitted from the buffering effects of the Pacific Ocean and California Current, both of which ameliorate climate extremes. Fish in coastal domains also encountered fewer anthropogenic hindrances to migration.

Southern DPSs also tended to score higher in vulnerability than northern DPSs. For example, coho from the southernmost Central California Coast DPS ranked higher in vulnerability than those from the mid-latitude Southern Oregon/Northern California Coast, which in turn ranked higher than the three northernmost coho DPSs. This latitudinal pattern was also evident at the scale of recovery domains, where DPSs of the three coastal domains in California and Oregon were generally more vulnerable than those of the two coastal domains in western Washington. However, exceptions to these general trends were not uncommon.

**Fig 7:**
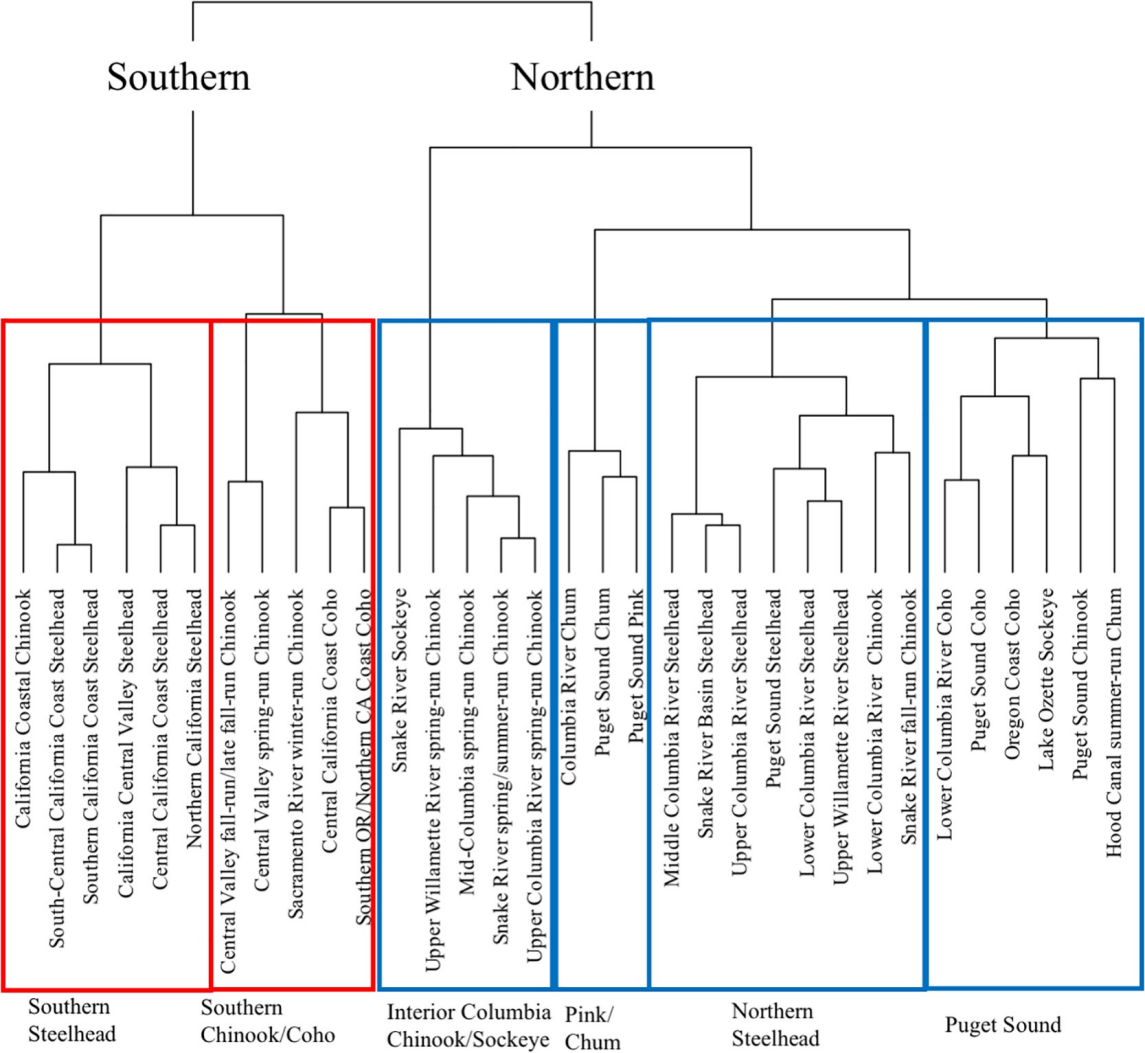
Cluster dendrogram based on attribute scores for each DPS. Groupings that define each cluster are outlined with red (southern) and blue (northern) boxes, with the cluster name below each box.

To better elucidate general patterns of vulnerability, we used a cluster analysis to group DPSs with similar vulnerability characteristics and examined these groupings in relation to geographical gradients and species characteristics. At the highest level of the dendrogram, DPSs clustered into southern and northern groups (Fig 7). *Flooding* was the best predictor of separation between southern and northern branches and the second best between coastal and interior branches. Southern and coastal DPSs faced higher *flooding* due to intensification of atmospheric rivers, which were projected to change more in southern than northern latitudes. Interior Columbia DPSs were less affected by these extreme rain events.

The next level of the dendrogram primarily separated DPSs by species (Fig 7), especially steelhead vs. other species. We noted that both southern and northern steelhead clusters included some fall-run Chinook DPSs. Classification and regression tree results pointed to the sensitivity attributes of *early life history* and *juvenile freshwater stage* as key predictors of separation between clusters at the species level (S9 Fig).

Steelhead spawn in late winter and spring, and hence are less sensitive to changes in fall and winter precipitation than fall-spawning salmon. Heat tolerance and behavioral flexibility also tended to reduce threat levels for steelhead in the *juvenile freshwater* stage. The Chinook DPSs that grouped with steelhead were primarily those with subyearling type juveniles. Their shorter freshwater period produced relatively low vulnerability scores during the *early life history* and *juvenile freshwater* stages. In particular, the fall-run subyearling juvenile type avoids dependency on rearing in freshwater during summer, when thermal impacts, hydrologic regime shifts, and low-flow impacts are expected to be highest. We grouped more similar DPSs into the six groups indicated with rectangles in Fig 7. Group names reflect the predominant species and region of DPSs in each group.

In the northern cluster of the dendrogram, spring-run Chinook and sockeye from the Interior Columbia grouped with Upper Willamette River Chinook. These three DPSs share a temperature-stressed adult migration and summer holding period. Puget Sound pink and both fall-run chum DPSs formed another group (with no listed DPS). The final group included all other DPSs from central and northern Oregon and western Washington.

Each cluster also displayed a unique vulnerability profile (Table 4). Profiles varied widely, from high scores in *ocean acidification* only (pink/fall chum), to high scores for freshwater and marine exposure but not for sensitivity to extrinsic stressors (Western Washington and Oregon), to high scores for freshwater and marine exposure and for extrinsic stressors (northern and southern steelhead), and finally, high scores in freshwater, marine, life stage, and extrinsic attributes (southern Chinook/coho as well as interior Columbia Chinook/sockeye).

**Table 4:** Vulnerability profiles by cluster. Mean cluster score was the mean attribute score across DPSs within the cluster. Scores were rounded down for each attribute. Red cells indicate a mean score of 3 or higher for exposure and sensitivity or lower than 1.5 for adaptive capacity.

		Vulnerability profile cluster group					
	Attribute	Southern Chinook/coho	Interior Columbia Chinook/sockeye	Southern Steelhead	Northern Steelhead	Western WA/OR	Pink/ Chum
**Freshwater Exposure**							
	Stream temperature	3	3	2	3	3	2
	Flooding	3	2	3	2	1	1
	Hydrologic regime	2	3	1	2	2	2
	Summer water deficit	2	2	2	2	2	1
**Marine Exposure**							
	Ocean currents	1	1	1	1	1	1
	Sea level rise	3	1	2	1	2	2
	Upwelling	3	2	2	1	1	1
	SST	3	3	3	3	2	2
	OA exposure	4	4	4	4	4	4
**Life Cycle Sensitivity**							
	Early life history	2	1	1	1	2	2
	Adult freshwater	2	3	2	2	1	1
	Juvenile freshwater	3	3	2	2	2	1
	Cumulative life-cycle	3	3	2	2	2	2
	Estuary	3	1	2	1	2	2
	Marine	3	2	2	2	2	2
	OA sensitivity	1	1	1	1	1	2
**Extrinsic Stressors**							
	Other stressors	3	3	3	3	2	2
	Population viability	3	3	2	2	2	2
	Hatchery influence	2	2	1	3	2	1
**Adaptive Capacity**							
	Adaptive capacity	1	2	2	3	2	2

In *adaptive capacity*, the southern Chinook/coho cluster had the lowest mean score. The northern steelhead cluster had the highest mean score, although this resulted from high scores for the two Chinook DPSs included in this cluster (see Fig 7). When the Lower Columbia River Chinook DPS was included in the Western Washington/Oregon cluster, that group had the highest adaptive capacity. Steelhead DPSs from the northern recovery domains had moderate adaptive capacity on average.

Specific attributes often scored in similar rank order across clusters (Table 4), although regional and biological variations were frequent and provided important insights for recovery planning. Among freshwater exposure attributes, *stream temperature* scored high in most clusters, but *flooding* was high only in the two California clusters, and *hydrologic regime* was high only in the interior cluster. Both of these latter attributes reflected changes in flow and/or precipitation, with increased flooding and drought more relevant in southern locations and loss of snowmelt more relevant in northern locations.

Among marine attributes, *exposure to ocean acidification* and *sea surface temperature* were highest in all clusters, with *sea level rise* second or a close third in both southern clusters as well as the cluster for pink/fall chum. For southern coho, sea level rise may not affect DPSs directly, but may represent a general threat to the freshwater/marine interface, triggering changes in lagoon habitat or sand-bar breaching. The most sensitive life stage differed among clusters, with adult stages more sensitive for interior DPSs, and juvenile stages more sensitive for southern coho/Chinook DPSs. Finally, among extrinsic sensitivity factors, *other stressors* was the most common attribute to score high, and paired with *hatchery influence* for the northern steelhead cluster and with *population viability* for the Interior Columbia Chinook/sockeye and California Chinook/coho clusters.

**Fig 8:**
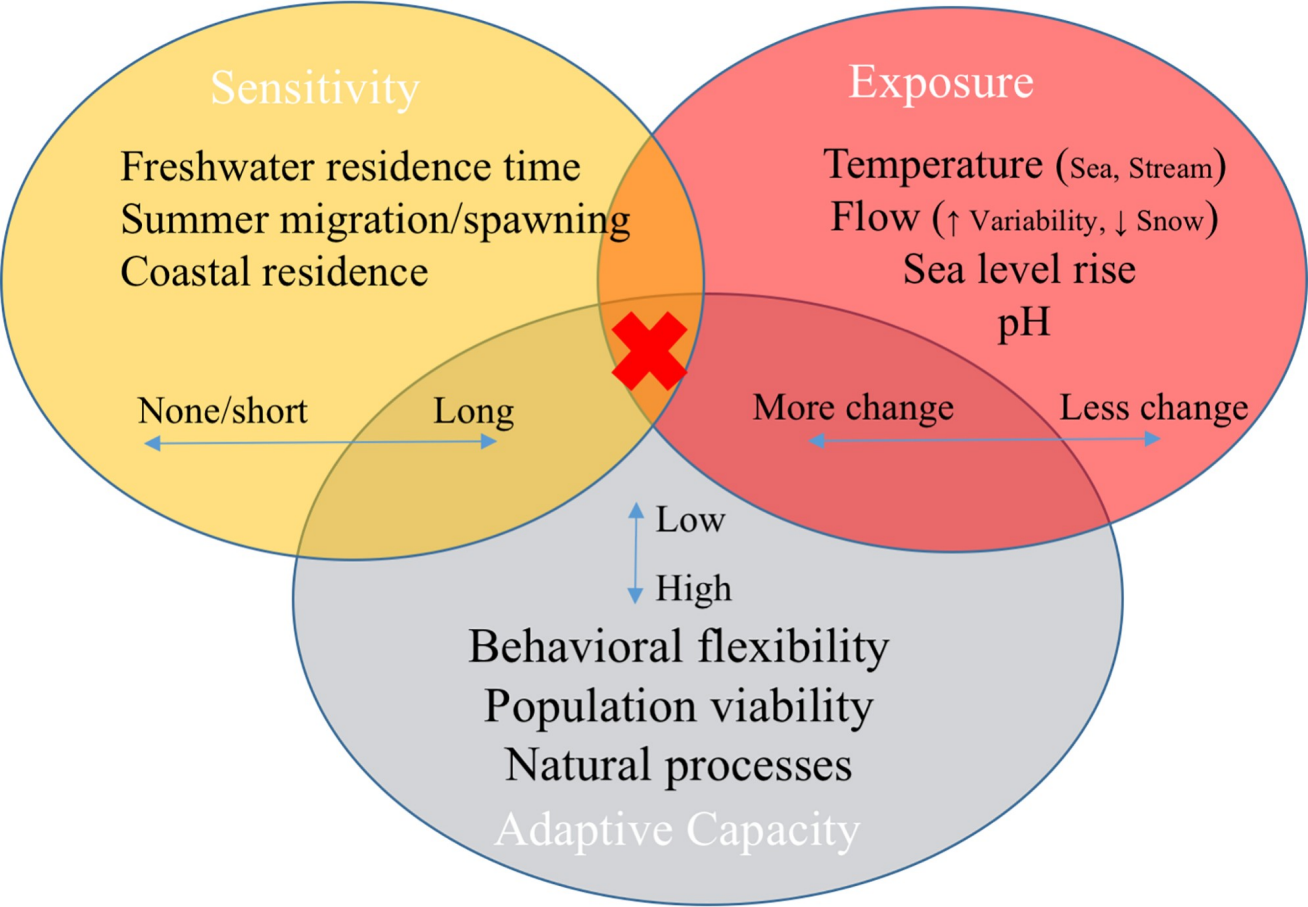
Conceptual model of highly influential attributes in final vulnerability ranks. The most vulnerable DPSs had scores in the intersection of high rates of change in exposure, long periods of sensitivity, and low adaptive capacity attributes (red x), as detailed in Table 4. Natural processes here refer to the absence of other stressors and hatchery influences.

Attributes that varied most across clusters reflected the major factors that differentiated the most vs. least vulnerable DPSs across the entire study (Table 4; Fig 8). Overall, the least sensitive DPSs spent the least amount of time in freshwater (pink and fall chum), while the most sensitive spent more time in freshwater, had long summer adult migrations, or were heavily dependent on estuaries and near-shore coastal rearing habitat. Exposure factors that indicated the highest vulnerabilities to climate change were encountered in both freshwater and marine environments.

Adaptive capacity also differentiated DPS clusters, especially in relation to behavioral flexibility, which relies on both inherent plasticity and habitat heterogeneity. Conceptually, we refer to "natural processes" as the absence of anthropogenic stressors, such as hatcheries and habitat loss (Fig 8). Anthropogenic stressors scored in the sensitivity component are linked to adaptive capacity in a broad sense because they are inherently more malleable than life histories. In general, DPSs with the highest sensitivity and exposure and lowest adaptive capacity were the most vulnerable to climate change, as indicated by the red x in Fig 8.

### Highly vulnerable life stages

Among life stages that scored high in both sensitivity and exposure, regional differences were pronounced. For coho in the two southern recovery domains and for Chinook in the Central Valley, DPS sensitivity scores were high at both the *estuary* and *marine stages*, and exposure scores were high for *sea level rise* and *sea surface temperature* (Table 5). For coho, steelhead, and some fall Chinook in the southern recovery domains, estuary conditions also affect access to freshwater spawning habitat, where watersheds are seasonally blocked by sand bar formation. Specific projections of how estuary and lagoon dynamics will change adult access were not available, and thus did not greatly change scores for the *estuary* or *adult stages* for these DPSs. However, potential obstruction to spawning habitat was noted as a concern by scorers.

**Table 5:** Highly vulnerable life stages by DPS with associated exposure attributes. Criterion for inclusion was a score of 3 or higher in both sensitivity and exposure attributes in each subheading. Additional high exposure scores for upwelling (a), flow regime (b), and flooding (c) also occurred in some DPSs.

Life stage and DPS	Exposure attribute
**Early life history**	Summer water deficit/Hydrologic regime
Sacramento River winter-run Chinook	Summer water deficit
Puget Sound Chinook	Hydrologic regime
**Juvenile freshwater stage**	Stream temperature
Mid-Columbia spring-run Chinook^**b**^	
Snake River spring/summer-run Chinook^**b**^	
Upper Columbia River spring-run Chinook^**b**^	
Lower Columbia River coho	
Oregon Coast coho	
Central California Coast coho^**c**^	
Southern Oregon/Northern California Coast coho^**c**^	
Puget Sound coho^**b**^	
**Estuary stage**	Sea level rise
Central Valley fall/late fall-run Chinook	
Central Valley spring-run Chinook	
Sacramento River winter-run Chinook	
Central California Coast coho	
Southern Oregon/Northern California Coast coho	
**Marine stag**e	Sea surface temperature
Central Valley fall/late fall-run Chinook^**a**^	
Sacramento River winter-run Chinook	
Central California Coast coho	
Southern Oregon/Northern California Coast coho	
**Adult freshwater stage**	Stream temperature
Mid-Columbia spring-run Chinook	
Middle Columbia River steelhead	
Snake River Basin steelhead	
Snake River sockeye	
Snake River spring/summer-run Chinook	
Upper Columbia River spring-run Chinook	
Upper Columbia River steelhead	
Upper Willamette River Chinook	
Central Valley spring-run Chinook	

^a^Exposure to upwelling also scored high

^b^Exposure to hydrologic regime also scored high

^c^Exposure to flooding also score high

Because of the highly modified Sacramento-San Joaquin Delta, all Central Valley DPSs were vulnerable at the *estuary stage*. Diversion of water from the delta supports the largest agricultural economy in the U.S. and provides drinking water to more than 20 million people [118]. Extensive water infrastructure in the estuary has dramatically altered flows and reduced survival of migrating fish. Furthermore, juveniles from all three Central Valley Chinook DPSs migrate predominantly as subyearlings, and as such are greatly dependent on estuary and near-shore habitat during the critical first year. These DPSs are therefore more vulnerable to sea level rise than DPSs with yearling-type juveniles.

All coho were highly vulnerable at the *juvenile freshwater stage* because of its extended duration (1+ years) and corresponding longer exposure to rapidly rising summer *stream temperatures*. In three of the five coho DPSs, *flooding* or *hydrologic regime* posed an additional high exposure at the *juvenile freshwater stage*.

Yearling Chinook, which are characteristic of many populations in the Interior Columbia recovery domain, were also highly vulnerable at the *juvenile freshwater* stage because of the year-round reliance during this stage on freshwater habitat. Although specific habitat preferences differ, both coho and Chinook are sensitive to changes in summer flow and *stream temperature*. Most Interior Columbia Chinook DPSs were also highly vulnerable to temperature in the *adult freshwater stage* due to long adult migrations in spring and summer through highly modified rivers, along with exposure to high summer *stream temperatures* during the holding period prior to spawning. Upper Willamette spring-run [119, 120] and Central Valley spring-run Chinook [121] face similar thermal challenges and high mortality between adult migration and spawning. Snake River fall-run Chinook did not score high in sensitivity to *stream temperature*, although adults do encounter high temperatures during late-summer migrations and have experienced compromised fecundity as a result [122, 123].

For most DPSs, sensitivity attributes were not scored high at the *early life history* stage. Puget Sound Chinook was an exception due to sedimentation and scour during flood events. Sacramento River winter-run Chinook also scored high in sensitivity at this stage. This DPS incubates eggs over summer, when *stream temperature* can be high if cold water in the Shasta reservoir is insufficient to cool the upper Sacramento River throughout the incubation period [38]. A recent analysis of Upper Willamette River spring-run Chinook indicated high temperatures are projected to increase mortality in the egg stage for this DPS as well, because spawning habitat is constrained by dams to the lower river reaches [124].

### Data quality

Most evidence used for scoring was based on quantitative data specific to each DPS, although DPSs were often represented by only a few index populations that were monitored consistently. In some cases, information on one DPS had to be inferred from a similar DPSs. Still, relative to information available for most marine fish, data quality was quite strong.

With the exceptions of the highly quantified projections for *exposure to ocean acidification* and *sea surface temperature*, sensitivity attributes tended to be based on higher-quality data than exposure attributes. Likewise, data for freshwater attributes was generally of higher quality than that for marine attributes (S10 Fig, top panel). Specifically, the freshwater life-stage sensitivity attributes of *early life history* and of *juvenile* and *adult freshwater stage* had relatively high data quality, as did assessments of *population viability* and *other stressors*. The weakest data for sensitivity attributes concerned sensitivity to *ocean acidification* and survival during the *marine stage*. In both of these cases, impacts on salmon were mediated by numerous potential food web interactions, which made net effects difficult to predict.

Some of the highest data-quality scores among the exposure attributes were from hydrological and stream temperature models. *Flooding* and *water deficit* exposures were less certain, and this was also reflected in high standard deviations in scores (wider spread across bins, S10 Fig, bottom panel). In the marine environment, data for *exposure to ocean acidification* and *sea surface temperature* were of very good quality, with consistent results across many models. However, projected changes in *ocean currents* and *upwelling* were inconsistent across models. Of all attributes, *upwelling* had the highest mean standard deviation of tallies across bins, indicating the largest uncertainty.

## Discussion

### Spatial and biological patterns in vulnerability

Patterns in climate vulnerability have important implications for Pacific salmon across the Pacific Coast, both in terms of total variation in life history diversity and in the likelihood of southern or interior range-edge contractions. The DPSs most vulnerable to climate change were those with life history types presently rare in the CCLME but prevalent further north, such as spring-run Chinook, and those unique to the species as a whole, such as late-fall and winter-run Chinook and summer-run chum. For Chinook, the highest vulnerability scores were for DPSs of the Central Valley and Interior Columbia recovery domains. For sockeye and steelhead as well, DPSs of the Interior Columbia scored higher than those of the coastal domains. This geographical pattern suggested a potential range contraction toward the coast for anadromous life histories unless access to higher-elevation habitats is restored and habitat quality in rearing areas and migration corridors is improved [108].

For coho, which have been extirpated from interior basins, vulnerability was very high in the entire southern portion of the range throughout California and southern Oregon. Finally, for steelhead, the southernmost salmonid in the CCLME, low *adaptive capacity* (potential loss of anadromy) and proximity to critical thresholds in the present climate raise the possibility of impending range contraction. Lower exposure scores for southern coastal DPSs suggest such a contraction could be coastward rather than northward. Resident forms of *O*. *mykiss* may persist in the inland areas, although these populations may become increasingly isolated [125].

#### Species-level results and similarities with other vulnerability assessments

Although we employed a rapid-assessment, our findings were of sufficient detail to provide conclusions similar to those of more geographically or ecologically focused studies [126–130]. Among species we considered, Chinook and coho had the greatest proportion of highly vulnerable DPSs. Climate vulnerability for the two sockeye DPSs was split between very high and moderate (Fig 4), while steelhead and chum DPSs were intermediate between high and moderate vulnerability. Puget Sound pink salmon scored lowest in vulnerability.

This species-level ordering was consistent with results from the West Coast Fish Climate Vulnerability Assessment (M. Haltuch, NOAA Fisheries, personal communication), which ranked Chinook salmon vulnerability *very high*, and coho, sockeye, steelhead, and chum *high*. In the Eastern Bering Sea Climate Vulnerability Assessment, all five salmon species scored high in sensitivity but low in exposure (P. Spencer, NOAA Fisheries, Seattle, personal communication). Lower exposure further north as well as increasing abundance and apparent range expansion of Chinook [131, 132] and Atlantic salmon (*Salmo salar*) [133] suggest that salmon species may shift the centroid of their respective ranges northward, as predicted by other niche-mapping studies [75, 76].

Several other groups have conducted vulnerability assessments that included Pacific salmon and steelhead. Both the NOAA Fisheries Multi-species Recovery Plan [126] and Moyle et al. [127] ranked California salmonids. Pacific Northwest steelhead were ranked by Wade et al. [128, 129], and all species were included in an assessment by the Washington Department of Fish and Wildlife [130]. Our relative ranks were similar to ranks from other studies, especially for Chinook and coho salmon, unlike recent reviews where systematic comparisons of vulnerability assessment results for terrestrial species found poor congruence [107, 134]. Salmon assessments may be more similar to each other both in the data that is analyzed and the categories of threats that are considered than across the broad spectrum of terrestrial taxa; a comparison of lizards produced a similar congruence [106]. Nonetheless, variation in spatial resolution and criteria for vulnerability do produce different results, which should be kept in mind when using these results in management decisions.

Steelhead vulnerability was somewhat more variable across studies than Chinook and coho, partly depending on whether authors rated loss of ecotypes vs. loss of the DPS as a whole. Studies with finer spatial and temporal resolution had greater differentiation of risk and generally higher vulnerability scores, potentially resulting from severe local stressors and specialized ecotypes. For example, certain parts of the Northern California steelhead DPS, specifically the summer-run ecotype, were scored as critically vulnerable in the *Multi-Species Recovery Plan*. On the other hand, studies with coarser spatial and biological resolution placed nearly all salmon and steelhead in a single, moderate-high or high-very high risk category [130].

Overall, the factors that caused highest vulnerability ratings among salmon DPSs are the same factors that caused higher vulnerability in many diadromous species compared with marine species [35]. As a functional group, diadromous species (e.g., sturgeon, *Acipenser* spp, Blueback herring, alewife and American shad, *Alosa* spp., and Atlantic salmon), had the highest proportion of vulnerable species in the Northeast Climate Vulnerability Assessment. The risk to Atlantic salmon was considered very high. Thus, salmon populations on both coasts are likely to contract northward for similar reasons [135]. Diadromous species rely on sequential freshwater, estuarine, and marine habitats; therefore, these species face a diverse suite of threats from climate change throughout their complex life cycles.

#### Specific climate threats

High exposure ratings throughout our results stemmed from a relatively consistent suite of exposure attributes (Table 4, Fig 8). Nearly all populations face high exposure to changes in sea surface temperature and ocean acidification, and most will confront considerable increases in summer stream temperatures. Accordingly, scores for these attributes were generally quite high (Table 4).

In freshwater and estuarine environments, other impacts varied by latitude and proximity to the coast. Exposure scores were generally higher for southern than for northern DPSs in *sea level rise*, *flooding*, and *upwelling*. Sea level is projected to rise more slowly in the northern CCLME, where geological uplift compensates somewhat for an expanding ocean [97, 136]. Dramatic increases in projected flooding along the West Coast stem from intensification of atmospheric rivers—a consequence of warmer temperatures over the Pacific Ocean [67]. Among present global models, California is projected to experience the greatest change in atmospheric rivers [67, 137–140]. Changes in the intensity and timing of *upwelling* are less certain. Nonetheless, present models suggest that the largest changes will manifest off the coast of California [98, 141], where relatively mild summer stream temperatures depend fundamentally on upwelling and the closely associated fog regime.

Salmon and steelhead in interior regions, as well as those in Puget Sound, had generally high DPS exposure scores for *hydrologic regime* due to loss of snowpack in mid- and high-elevation watersheds. Snowpack is already declining in response to warmer winters throughout the western U.S. [4, 142, 143]. In mountainous regions, warmer winters will transform snow-dominated hydrographs with low winter flows followed by a protracted spring snowmelt to systems characterized by rapid snowmelt and high-flow events during the incubation period [142]. In western Washington, salmon populations may soon lose snow-dominated watersheds entirely [144]. Such losses are expected to reduce life history diversity within these DPSs [68]. While these DPSs may be buffered from outright extinction by their existing behavioral diversity, losses of habitat diversity and cooling influences of snowmelt may increase vulnerability [104].

Interior Columbia DPSs face the largest percentage loss of snow-dominated habitat [144]. These populations are dominated by life history types specifically adapted to elevated flows in spring, which expedite juvenile migrations of up to 1500 km. Summer stream temperatures are also cooler in snow-dominated basins. Characteristic life history strategies in these regions, such as summer juvenile rearing and adult holding depend on these cooling influences. Hence, these genetically distinct life histories are perhaps most threatened by loss of snow cover.

California steelhead tended toward more moderate exposure scores for stream temperature because of a weaker link between rising atmospheric and *stream temperature* in coastal California. Heat-moderating factors such as coastal fog, riparian evapotranspiration [145], and groundwater inputs, are especially relevant in some locations and contribute to a decoupling of stream and air temperatures [146]. This decoupling suggests a potential capacity for thermal refuges from rising air temperatures, all else being equal. However, the buffering capacity of mitigating factors such as fog could diminish in a warmer climate [147], increasing exposure to *stream temperature* well beyond the moderate levels we scored.

Decoupling of air and water temperatures can also result from a high frequency of intermittent streams in rain-dominated basins such as those in southern California [148, 149], and elsewhere [150]. Climate change could entail an end to this decoupling process as well, limiting future habitat to a greater extent than reflected in our stream temperature exposure scores [151, 152]. In short, coastal steelhead in California may be somewhat protected by thermal refuges, but the factors maintaining those refuges themselves likely have climatic thresholds beyond which they cease to operate.

#### Most vulnerable life stages

Salmon life history types are closely tied to hydrological conditions, so the geographical patterns in exposure factors parallel trends in highly vulnerable life stages. At the scale of this assessment, Chinook demonstrated these patterns most clearly because DPSs differ systematically in the duration of freshwater stages [20, 68, 153]. Southern Chinook DPSs currently lack access to snow-cooled juvenile habitat, so they characteristically smolt as subyearlings. Subyearling juveniles are more vulnerable to near-shore development, *sea level rise*, and *upwelling*. Thus for southern Chinook DPS juveniles, the *estuary* and *marine stages* were highly vulnerable (Table 5). For yearling Chinook and coho migrants, sensitivity scores were higher at the *juvenile freshwater stage* than the *estuary stage* because of their extended freshwater rearing strategies. These strategies, however, make them more vulnerable to *stream temperature* increases and loss of snowpack (*hydrologic regime shift*).

All of the DPSs with a highly vulnerable *adult freshwater stage* migrate in spring or summer, so they are exposed to high *stream temperatures* and pre-spawning mortality. The interior populations also confront long migrations. For southern coastal species, sensitivity in the adult stage might have scored higher due to difficulties accessing freshwater habitat. However, we could not quantify this difficulty owing to uncertainty regarding net change in sand-bar breaching. Our results primarily reflected the fact that longer migrations and freshwater phases expose salmon to more numerous freshwater climate threats and anthropogenic stressors.

Nevertheless, the steelhead we considered, including those with extended freshwater phases and migrations up to 1500 km (Table 1), tended to score lower in sensitivity than Chinook in the same region. Greater resilience in steelhead stems from several factors. First, steelhead inhabit streams warmer than those used by Chinook or coho salmon [61, 154, 155]. Compared to spring-run Chinook and sockeye salmon, steelhead also display greater mobility during migration, utilizing high-elevation, high-velocity, and hard-to-reach or ephemeral and intermittent stream habitats, as well as cool-water tributaries for temporary staging [156]. Despite these advantages, steelhead access to freshwater habitats can be intermittent and hindered by changes in storm frequency [157]. Second, although both species have a strong genetic component in life history traits, *O*. *mykiss* typically expresses more life history strategies within DPSs, so the DPS as a whole appears less vulnerable than Chinook DPSs [22, 158].

Relatively few DPSs appeared highly vulnerable in their *marine stage*. However, this was also the stage with the greatest uncertainty in scores. While physical conditions in freshwater are clearly and directly linked to salmon survival, factors that influence ocean survival are more complex [159]. Physical processes in the ocean affect salmon through their influence on prey availability and abundance, as well as through the spatial distribution of competitors and predators. Ocean food webs contrast sharply in cold vs. warm years [88, 160, 161]. The combination of increasing *sea surface temperature* and *ocean acidification* heightens the risk for a major, novel reorganization of marine ecosystems.

Marine biological regime shifts of the past are well documented [162–164], and demonstrate widespread ecological responses to change in ocean conditions. These regime shifts were associated with climate changes much more subtle than those projected over the next few decades; hence they provide only hints of potential of future impacts. For salmon especially, specific consequences of ocean regime change are hard to predict, owing in part to the general non-linearity of marine ecosystem dynamics, along with the numerous possible fish communities that could establish themselves [40]. Nonetheless, prolonged periods of poor ocean survival have been observed during generally warm decades [165]. In recent warm years, a high proportion of empty stomachs were observed in juvenile salmon, as well as poor body conditions, despite an abundance of prey biomass [166]. Thus although we have highlighted risks in freshwater stages, these findings suggest that warmer oceans could be catastrophic for salmon populations throughout the CCLME, as has also been suggested for Atlantic salmon [135].

### Adaptive capacity

Although the *adaptive capacity* score was not as formalized as the rest of the assessment, results are consistent with larger patterns in habitat and life history diversity. Among DPSs with similar life history diversity, those that scored higher in a*daptive capacity* occupied habitat that was climatically diverse but generally closer to optimal for salmon. Such habitats featured moderate temperatures and wetter overall climates—conditions that support a large range of salmon life histories. Northern California steelhead occupy the interface between the more xeric southern and interior eco-climatic zones and the wetter zones of coastal Oregon and Washington, and they exhibit a wide range of juvenile and adult behaviors. Lower Columbia River Chinook and steelhead and Puget Sound Chinook also display various life histories at multiple life stages. Puget Sound and Lower Columbia DPSs benefit from steep elevation gradients of the Cascade and Olympic Mountains, which provide cool water without the hazards of a lengthy migration. This advantage was reflected in the lower life-stage sensitivity scores for the western Washington/Oregon cluster (Table 4).

For some DPSs, high *adaptive capacity* scores reflected direct evidence of adaptive change. For example, for Snake River fall-run Chinook, a shift in the proportion of fish adopting yearling vs. subyearling juvenile life history strategies has been observed [7, 167]. Increased proportions of yearling type fish appear to have evolved in response to anthropogenic habitat modifications. If climate change favors a reversal of this trend, then this DPS may be expected to continue such adaptive responses. Shifts in adult run timing have also been observed for multiple DPSs in the Interior Columbia recovery domain. Evolutionary changes in run timing were associated with environmental change [31], as well as hatchery supplementation [168]. Some of these DPSs inhabit heavily modified areas; for example, most Snake River salmon must pass eight large hydroelectric dams during both the juvenile and adult migration. Puget Sound DPSs inhabit an area of rapidly expanding human population, with a projected increase of 42% by 2050 [169]. However, unusual behaviors have emerged under altered conditions [170–172], suggesting that adaptive responses to climate change will continue to arise.

Most DPSs that scored high in adaptive capacity benefit from complex terrain that includes snow-cooled streams. However, these snow-dominated hydrological regimes have been consistently projected to disappear during the present century [144, 173], potentially causing a net contraction in life history variability. Thus, the selective landscape could shift to favor a different balance of traits, including some that are not exhibited now. Other DPSs scored moderate in *adaptive capacity*, either due to life history constraints in the case of chum and pink salmon, or due to habitat loss and degradation in the case of interior steelhead and other Chinook.

*Adaptive capacity* was ranked *low* for the three Central Valley Chinook DPSs, along with Central California Coast coho and Snake River sockeye. These ranks were associated with *high* scores for extrinsic exposure attributes and cumulative *life-cycle complexity* (Fig 5 and Table 4). Chinook, coho, and steelhead DPSs in the two southern clusters had the lowest population viability scores and highest exposure to anthropogenic impacts (Table 4). These impacts included significant *hatchery influence* and *other stressors* such as water withdrawals/diversions, habitat degradation, loss of access to higher elevation (cooler) spawning and rearing habitats [53, 108], and potential competition or predation from invasive species. Many of these stressors are expected to increase with climate change, as human demand for water increases [3, 4], warm-water invasive predators expand their range [174–180] and the virulence of some diseases intensifies [181–184].

Reductions in abundance, genetic and phenotypic variation, along with proximity to environmental tolerance limits, has brought many DPSs to a threshold of critical impairment to life history types. Low *adaptive capacity* and high *cumulative life cycle effect* scores reflected the fact that without access to historical habitats [108, 185, 186], southern DPSs have fewer options for behavioral mitigation of climate impacts [187] than their conspecifics to the north.

In part, low adaptive capacity scores for Central Valley Chinook resulted from its various life history types that have differentiated over evolutionary time and are considered distinct from one another at the DPS level. Each of the three Central Valley Chinook run types is specialized to a particular aspect of the hydrologic profile, and thus each is especially vulnerable to hydrologic change. In contrast, summer and winter steelhead run types are less genetically distinct and currently considered part of the same DPS [158]. Moreover, anadromy itself is more variable in steelhead than in Chinook. Many steelhead populations interbreed with resident forms of *O*. *mykiss*, with the frequency of alleles relating to anadromy fluctuating over time [125, 188]. Climate risks to steelhead include loss of the anadromous life history type as a major component of the DPS.

### Historical trends in loss of diversity

We identified patterns of climate vulnerability that mirrored patterns of extinction estimated for all six species we assessed. Gustafson et al. [189] enumerated loss of historical populations and DPSs in the western U.S., with the concurrent loss of ecological, genetic, and life history diversity. Although overall estimated losses were considerable (29%), they found evidence of fewer extinctions along the northern coastal regions (<20%) compared with southern California (35%), the Central Valley (57%), and the interior Columbia Basin (35-62%). These patterns typified the north-to-south and coast-to-interior gradients in our vulnerability scores.

Greater losses in the interior domains were primarily due to large, impassable dams, which eliminated many populations simultaneously. Gustafson et al. [189] also found that for Chinook salmon and steelhead, extinction rates of stream-maturing populations with longer freshwater phases were higher than those of ocean-maturing populations that reside in freshwater for shorter periods. They found greater extinction rates in sockeye and coho compared with pink and chum salmon, also reflecting the predominant patterns seen in our assessment. Such similarities supported our conclusion that freshwater-dependent life history types are more vulnerable, and that climate change will likely continue the direction of anthropogenic pressures that have accumulated over the past two centuries.

Overall, both historical and future losses of diversity pose a critical challenge for all Pacific salmon species. At both the DPS and species level, the most fundamental components of *adaptive capacity* are life history diversity, physiological performance, behavioral and morphological plasticity, and genetic variability. However, for West Coast salmon populations, some of the most distinctive and rare characteristics are those at greatest risk. At the same time, large proportions of stream habitat that could provide refuges to help sustain these populations have been lost to anthropogenic barriers [190].

### Methods of increasing climate resilience

Most of the DPSs we evaluated are listed as species of concern, threatened or endangered under the ESA or are considered by states as sensitive, almost entirely as a result of anthropogenic stressors. Reducing anthropogenic stressors could greatly improve responses to climate change by improving the overall status of these DPSs in terms of abundance, productivity, spatial structure, and diversity.

A robust DPS has greater resilience by virtue of strong population dynamics that make stochastic extinction less likely. Such strengths rely on population spatial structures that provide refuge from disturbances and can allow adaptation to occur at fine scales, as well as diversity in genetic makeup, life history, behavior, and morphology [34, 108]. These processes provide the needed raw material to respond to climate change, allowing for a “portfolio effect” that reduces volatility and risk to the larger demographic unit [191–196]. Increasing synchrony in both climate [152, 197] and salmon population responses [198] indicates declining inter-population diversity and presents a major threat to DPS persistence.

Climate change presents an array of specific threats that can act synergistically with other threats, dramatically increasing the impacts of each [108]. In particular, the loss of population spatial structures, as well as habitat heterogeneity and connectivity, removes the means by which salmon have historically persisted through frequent disturbances and climate extremes. Recent analyses in terrestrial environments found a correlation between habitat loss and climate stress [199]. An analysis of bull trout (*Salvelinus confluentus*) also found that genetic richness is lower in habitats with the highest climate exposure [110]. Thus, due to past adaptation or recent stressors, *adaptive capacity* may already be at its lowest levels precisely where salmon need it most. In prioritizing conservation actions, it is therefore worth exploring specific interactions between existing threats and climate drivers.

Habitat restoration is especially important in allowing salmon to express their intrinsic life history diversity. Salmon are highly adapted to disturbance regimes, but they need access to a wide variety of physical and thermal conditions within a watershed if they are to respond to increasing climate variability, such as frequent flooding or persistent droughts. Three main themes have emerged from recent literature (e.g., [55, 108, 200]). First, reconnection of habitats blocked by artificial barriers, either longitudinally or laterally (floodplains), can be highly effective in expanding the effective climate space of a watershed. Reconnected habitats restore natural processes and provide refuges from extremes in both temperature and flow. Second, amelioration of temperature or flow constraints can actively reduce climate stress, for example, through hypolimnetic releases from reservoirs, reconnection to historical sources of cool water, riparian restoration, and other techniques. Finally, identifying and improving access to food-rich environments can improve tolerance of climate stress by reducing bioenergetic constraints and mortality risks that are often lower for larger fish.

Projects focused on restoration and protection of accessible habitat are underway in numerous river systems, although the scope of work needed for species recovery sometimes involves nearly all existing habitat [201]. Nonetheless, when estuarine and freshwater habitats and processes are restored, natural environmental complexity provides a buffer against climate impacts in some cases [202]. Model results show that restoration can mitigate for declines that would otherwise result from climate change [203–205]. Guidelines to identify habitat restoration actions that will have a climate benefit have been developed [55] and are being used to realign priorities in some watersheds [206], but have not become the norm [207]. Management of freshwater stream temperatures and flows to support a diversity of salmon life history strategies, as well as to improve survival (and thus abundance and productivity) will be a crucial tool for increasing resilience to climate change [108, 208, 209].

Large, impassable dams block access to large areas that could serve as climate refuges as well as supporting more diversity and larger populations in general [208, 209]. There have been major improvements in fish passage at dams on the mid and lower Columbia and Snake Rivers [210], and reintroduction of coho to the interior Columbia is currently underway [211]. Furthermore, removal of dams has become much more frequent in recent years, including dams on the Elwha, Rogue, White Salmon, Sandy, and Carmel Rivers [212–215]. Salmon responded rapidly when multiple dams were removed in the Rogue [216], Sandy [217] and Elwha River basins [218–220], as did other salmonids, including re-establishment of the anadromous life history in bull trout (*Salvelinus confluentus*) [221].

Nonetheless, a large fraction of historical salmon habitat is still completely inaccessible [190, 222]. Pilot efforts to establish experimental populations above some dams are underway. For example, reintroduction of winter-run Chinook to historical habitat in the Sacramento River Basin has involved removal of migration barriers and restoration of more natural flow (Battle Creek [223]), as well as transport above barriers that will continue to be impassable (McCloud River, above Shasta Dam [224]). Similar projects exist for coho, Chinook, and sockeye salmon and steelhead in the Columbia River Basin [225–228]. However, because certain dams will not be removed in several of these plans, assisted migration using trap-and-haul operations will continue to be essential [185, 186, 224], adding uncertainty for long-term population viability. In other cases, the inadequacy of existing dams to cope with new extremes of flow and sediment movement may support removal as a tool to mitigate climate change impacts.

Hatchery supplementation can reduce fitness in wild salmon populations both through introducing maladaptive genotypes and reducing the effective population size of wild populations [229, 230]. Therefore, reducing the number of hatchery-origin fish in general can be expected to improve the adaptive capacity of wild populations in the face of increasing exposure to climate change. In the case of highly endangered populations, however, hatcheries can provide a short-term buffer from extinction risks [231], which is the primary risk for salmon during adaptation to climate change. Criteria for limiting introgression between hatchery and natural-origin fish have been developed to reduce the risks of domestication [232]. Furthermore, improvements in hatchery spawning techniques, mating designs, incubation and rearing protocols, may reduce the potential for inbreeding and domestication selection [233].

Harvest practices also could be adjusted based on periods and conditions when populations are less stressed. For example, catch-and-release fisheries or fishing closures are used to restrict angling to cool temperature periods. Such practices mitigate the interaction between handling and temperature stress [234], but run the risk of accidentally selecting on run timing [235] and other traits [236, 237]. Consideration of how all anthropogenic factors exacerbate or possibly mitigate for climate stressors is much needed [238]. For example, fisheries typically select for smaller body size and shorter generation time, which could also be advantageous in a warming climate [239, 240]. However, these traits also reduce fecundity and population stability, which is ultimately disadvantageous for both humans and salmon population viability [239–243].

More active proposals of assisted gene flow and gene editing are being proposed to introduce more heat tolerant genotypes into hatchery programs [244] and wild populations [245–247]. However, as with any new technology, risks are difficult to quantify and there are many factors that need to be considered [248–255]. In many cases, humans have intentionally or unintentionally caused traits to shift in direction or variability that are maladapted for climate change [238, 256–258], putting some DPSs at additional risk. More research is needed to identify best practices in relation to anthropogenic selection (e.g., [242]). Though many uncertainties remain to be addressed, all of these avenues can potentially improve opportunities for local adaptation and overall survival.

## Conclusion

Loss of the southernmost populations within a species’ range is widely predicted with climate change [259], but our assessment also highlighted that unique life histories are at high risk. Both the late-fall and winter-run Chinook ecotypes exist only at the southern end of the species range, and both face extinction without continued intensive management. Similarly, for chum salmon, the summer-run is rare and faces relatively greater vulnerability than the more common fall or winter-run life history types in northern regions. Local adaptations to distinct flow and temperature conditions are the characteristics that contribute to high vulnerability for these life history types and make them particularly sensitive to climate change.

In addition to southern range contractions, we found that interior losses may be even greater, due in part to greater change to interior climates and anthropogenic constraints on migration pathways. Some life histories ranked highly vulnerable by us or others, such as spring-run Chinook and northern California summer-run steelhead, will still be represented further north. However, Chinook salmon and steelhead that evolved distinct lineages in interior basins [21, 22] are at risk of losing some of their unique life histories not only in the Columbia River Basin but also in the neighboring Fraser River Basin in Canada [32, 260, 261]. The evolution of early adult migration (spring-run Chinook and summer-run steelhead) appears to reflect a rare event that would be quickly lost if these migratory pathways are selected against [158]. Declines in these life histories could entail significant loss of diversity in these species as a whole.

The highest scores for extrinsic effects (anthropogenic stressors) occurred in interior and southern regions (Table 4), exactly where climate is expected to change the most. A similar pattern in smaller-scale genetic analyses [110] suggests this could be a widespread phenomenon. Efforts to promote resilience to climate change are similar to those that increase viability more generally and have been part of historical conservation practices. However, our assessment indicates that more intense and perhaps novel efforts will be needed to compensate for the added pressure from climate change. Additional research to refine this assessment and explore adaptive capacity would be especially valuable. For DPSs that scored high in adaptive capacity, particular care is warranted to avoid loss of life history diversity and thus maintain the flexibility to continue adapt to climate change in the future. Resource managers should expect changes in fish characteristics, such as run timing and body size, but also other responses which have unknown consequences for population viability.

By pointing to the most vulnerable DPSs, identifying the most vulnerable life stages within each DPS, and assessing where life histories are most likely to change, these results provide a framework to support recovery planning for climate change impacts on West Coast salmon. This assessment considered present conditions, and therefore present risks confronted by Pacific salmonids that are related to climate change. Most, if not all, Pacific salmonid habitat in the western U.S. has diverged significantly from historical conditions and processes. Where dams block passage and interrupt ecological and physical processes, dam removals will likely result in habitat that diverges less from those seen historically. This is likely to reduce impacts of climate change for fish at all life stages. As demonstrated by recent dam removals and restoration activities that reconnect floodplains, physical and ecological responses can be rapid and can effectively reduce habitat constraints on these systems [217, 218]. Thus, we may be able to provide some relief to the extensive climate change risks highlighted in this vulnerability analysis.

## Supporting information

S1 Appendix Biological sensitivity attributes. (PDF)

S2 Appendix Climate exposure factors. (PDF)

S3 Appendix Distinct population segment scores and narratives. (PDF)

S1 Table Exposure factor data. (XLSX)

S2 Table Distinct population segment scores. (XLSX)

S3 Table Adaptive capacity scores. (XLSX)

S1 Fig Chinook salmon habitat. (JPG)

S2 Fig Fall Chinook salmon habitat. (JPG)

S3 Fig Winter Chinook salmon habitat. (JPG)

S4 Fig Chum salmon habitat. (JPG)

S5 Fig Coho salmon habitat. (JPG)

S6 Fig Pink salmon habitat. (JPG)

S7 Fig Sockeye salmon habitat. (JPG)

S8 Fig Steelhead habitat. (JPG)

S9 Fig Classification and regression tree results. (PDF)

S10 Fig Data quality scores and standard deviation of tallies for each attribute. (DOCX)
