# Supplementary material for: Climate vulnerability assessment for Pacific salmon and steelhead in the California Current Large Marine Ecosystem
Source: PLoS One. 2019 Jul 24;14(7):e0217711. doi: 10.1371/journal.pone.0217711 (PMC6655584; doi:10.1371/journal.pone.0217711)
Supplement: S1 Appendix — (PDF) [file pone.0217711.s001.pdf]

S1: Biological Sensitivity Attributes

Supporting information for:

Climate vulnerability assessment for Pacific salmon and steelhead in the California Current Large Marine Ecosystem

Lisa G. Crozier, Michelle M. McClure, Tim Beechie, Steven J. Bograd, David A. Boughton, Mark Carr, Thomas D. Cooney, Jason B. Dunham, Correigh M. Greene, Melissa A. Haltuch, Elliott L. Hazen, Damon M. Holzer, David D. Huff, Rachel C. Johnson, Chris E. Jordan, Isaac C. Kaplan, Steven T. Lindley, Nathan J. Mantua, Peter B. Moyle, James M. Myers, Mark W. Nelson, Brian C. Spence, Laurie A. Weitkamp, Thomas H. Williams, Ellen Willis-Norton

Biological Sensitivity Attributes.. 1
Early Life History.. 2
Juvenile Freshwater Stage 3
Estuary Stage 5
Marine Stage.. 6
Adult Freshwater Stage 7
Cumulative Life-Cycle Effects... 9
Hatchery Influence 10
Other Stressors... 11
Population Viability... 14
Ocean Acidification Sensitivity.. 15
Literature Cited.. 17

Early Life History

Goal: Identify climate-limiting factors in the early life history, especially egg incubation and early fry stages

Questions for profile:

- When is the egg incubation period?
- What climatic factors are most relevant for this DPS?
- How close are egg incubation conditions to critical maximum limits in temperature or flow in the present climate, and how much does this margin vary across the DPS?

Explanation:

Salmon populations are inherently highly sensitive to environmental factors during the egg incubation stage. Salmon eggs are buried in gravel and thus subjected to whatever environmental conditions impact the redd location. Salmon eggs have well-established physiological constraints, with the narrowest range of temperature tolerance of any life stage (0-15°C). Eggs also require dissolved oxygen levels of at least 8 mg/L (Davis 1975), and dissolved oxygen can reach stressful levels under low flow or high sediment conditions (Martin et al. 2017). Redds can also be physically dislodged during high flow events. These risks vary with adult behavior, such as spawn timing and the depth and exact location of redds, as well as with watershed geomorphology and climate.

Because exact redd locations and microclimate will never be known for all populations, we rely on a qualitative assessment of whether the DPS is confronting or close to confronting a limiting factor in this life stage.

A change in hydrological regime that alters precipitation patterns, as from a snow-dominated to a transitional or from a transitional to a rain-dominated regime, is likely to affect the egg incubation stage because most Pacific salmon spawn in the cool/wet season. Low flows generally do not pose a problem along the West Coast during the cool/wet season, but redds can be threatened by elevated flows. For many populations, intense precipitation or rain-on-snow events may elevate flows enough to scour redds or increase sedimentation loads enough to suffocate them.

Some populations are threatened by high temperatures during this stage, especially if eggs incubate over summer. For eggs that incubate during winter, thermal stress is also a potential threat in the shoulder seasons (fall and spring). However, adults usually delay fall spawning until temperatures drop below the lethal limit for eggs. In spring, emergence is typically advanced by warmer temperatures, such that eggs are

unlikely to be exposed to thermal stress. Stress in the fry stage is discussed in the following attribute.

Response Bins

Low: The DPS seems to be relatively protected from flow and temperature stress during the egg incubation stage.

Moderate: The DPS seems to be relatively protected from flow and temperature stress during egg incubation. However, high uncertainty about this status or high variability across populations may expose the DPS as a whole to moderate risk during this life stage.

High: At present, the DPS does not experience much climate-related mortality during egg incubation, but at least some populations are near environmental thresholds.

Very high: Flow-related or temperature stress already occurs within this DPS during egg incubation.

Juvenile Freshwater Stage

Goal: Identify climate-limiting factors and the potential for adaptation in the fry-to-smolt stage

Questions for profile:

- When do eyed eggs hatch and when do fry initiate movement?
- Do juveniles typically migrate at age-0, age-1, or a mix?
- Are rearing conditions limited by temperature or flow?

Explanation:

Salmon have highly variable freshwater rearing periods that are generally adapted to local environmental conditions. Fry and parr have bioenergetic constraints that are very sensitive to temperature, and their behavior is strongly responsive to temperature and flow. Survival is also strongly correlated with temperature or flow in many cases.

Timing of the smolt migration (or absence of migration entirely for life-long freshwater residents) reflects a trade-off between growth opportunity and mortality risk in freshwater vs. saltwater habitats. Typically, salmon migrate at a younger age in warmer locations, particularly if rearing habitat becomes stressful. A warmer climate is likely to reduce smolt age in general, both as a plastic and an evolutionary response.

Vulnerability of a DPS to climate change depends on the present distribution of life history types, as well as existing direct environmental constraints. Juveniles that spend one year or more in streams may be especially vulnerable to low or high flows and high summer temperatures.

Subyearling life history types are also highly responsive to environmental conditions during the freshwater stage and may be unable to avoid thermal stress or stranding due to low flows. However, in many cases subyearlings can avoid peak temperatures by transitioning to the smolt stage earlier in the year. This strategy may ultimately transfer potential stressors to the next life stage, but reduces vulnerability in the parr or fry stage relative to yearling life histories.

Populations with predominantly yearling juvenile life history types may be most at risk from climate change. Where migratory behavior is more evenly mixed between yearling and subyearling life history types, behavioral flexibility has already been demonstrated. Thus, we assume less risk in shifting the proportion toward more subyearling life history strategies. Populations that already display subyearling behavior are somewhat preadapted to a warmer climate. However, the ability to avoid mortality by transitioning to the smolt stage may be limited. Thus, even mixed populations may experience reduced survival under warm/low flow conditions.

Response Bins

Low: Populations least vulnerable in the freshwater juvenile stage exhibit an entirely subyearling life history, with ample migratory opportunity to avoid environmental stress in fresh water.

Moderate: Populations are comprised of some yearlings, but mostly subyearlings that face no imminent climate limitation. Loss of the yearling life history would not threaten the DPS per se, because the subyearling type already dominates the populations; however, within the DPS, some diversity will likely be lost.

High: Populations exhibit a mix of subyearling and yearling behaviors. Loss of the yearling life history would constitute a significant reduction of diversity within the DPS, and effects on later life stages are not fully known. Also at high risk are DPSs that presently experience moderate constraints on migration from temperature or flow, regardless of juvenile life history type.

Very high: Populations are dominated by yearling (or older) life history types, and the ability to switch or consequences of switching to a subyearling migration strategy is unknown. Loss of yearling smolts will directly threaten either the persistence or characteristic life history of the DPS. Alternatively, populations are dominated by the subyearling life history type, but many of them already experience reduced survival in this life stage due to environmental conditions.

Estuary Stage

Goal: Identify climate-limiting factors in the estuary stage

Question for profile:

- Describe estuarine location, residence time and existing climate-related stressors in this habitat

Explanation:

Salmon spend variable amounts of time in estuarine habitats, with periods of residence ranging from hours to months. All anadromous salmon use estuary habitat to acclimate to reversals in osmoregulation, both when they migrate downstream as juveniles and when they return as adults. Because salmon are well adapted for osmoregulatory switches, acclimation in estuaries may occur within hours or days.

Nevertheless, acclimation can be a stressful process such that additional stresses from high temperature, hypoxia, or low pH can have harmful effects during estuary residence. Hypoxia and low pH in the Columbia River estuary are linked to an influx of upwelled coastal water (Roegner et al. 2011), so changes in upwelling intensity, timing, or duration may have negative effects on estuary residents.

In addition to their importance from an osmoregulatory standpoint, estuaries provide sheltered rearing habitat for juveniles that migrate from upstream habitat at relatively small size. Estuary habitats allow these fish to grow rapidly prior to entering the ocean. If juveniles spend less time in freshwater habitats because they become unsuitable, or if they reach growth thresholds for smolt transition earlier in the year, they may need to spend more time in estuarine habitats.

These habitats are likely to experience changes from sea level rise, storm surge, and increased temperatures. Estuary habitat capacity may be decreased from reduced river flow, and changes in circulation may alter the extent of brackish water or water quality. In addition, estuaries will likely be focal points of anthropogenic adaptations to climate change, such as increased armoring.

Response Bins

Low: Populations exhibit either relatively short estuarine residence or extensive bet-hedging strategies, such as diverse timing and duration of estuary residency. Populations that make extensive use of estuaries are also less likely to be vulnerable.

Moderate: Populations exhibit moderate estuarine residence time and bet-hedging strategies, estuarine areas facing medium stress factors are utilized.

High: Populations have limited access to estuarine habitat areas and extensive estuary residence periods.

Very high: Populations face existing climate-related threats in the estuary rearing stage, either from habitat stressors or curtailed access.

Marine Stage

Goal: Identify climate-limiting factors in the entire marine stage

Questions for profile:

- Describe early marine to adult migration behavior.
- What climate predictors have been associated with marine survival or behavior, and how strong is this correlation?
- Are there known climate correlates or thermal stressors during the adult return phase (prior to freshwater entry)?
- How much variation exists in age at maturity within the DPS and among years?

Explanation:

All salmon populations have high variability in survival during the marine rearing stage. This variability is generally related to trophic conditions, which magnify the inherent vulnerability to population collapse during prolonged periods of low ocean productivity. Profile questions are largely intended to identify DPSs with relatively greater bet-hedging ability, either because of spatial and temporal diversity in migration behavior or from overlapping age-class cohorts, both of which spread the risk of collapse over multiple ocean-entry years.

Generally, we lack the ability to develop mechanistic models that link specific ocean climate projections to early marine survival such that we might differentiate sensitivity to climate change among DPSs. Therefore, we rely on diversity within the DPS as an indicator of resilience. We request a description of climate correlates of survival in case particular information is available for climate projections now or in the near future.

For populations that arrive in the ocean over a consistent, narrow temporal window and migrate through the same corridors from year to year, we assume greater vulnerability to a change in ocean conditions within those spatial and temporal windows. Although the exact mechanisms that determine survival might be unknown, these

populations typically show strong correlations between ocean conditions and smolt-to-adult return rates (SARs). Greater diversity in juvenile migration timing typically leads to a larger "portfolio effect" that buffers declines in any particular aspect of ocean productivity.

Adults also display variable flexibility in the spawning migration, with some holding at sea until freshwater conditions become suitable and others moving rapidly through the final migratory stage with consistent survival over existing climate variability.

Response Bins

Low: Populations exhibit bet-hedging strategies that include diverse timing and rate of the juvenile and adult migrations with highly variable marine distributions. This DPS exhibits low correlations between SARs and climate indicators.

Moderate: Populations exhibit moderate bet-hedging strategies with diverse timing and rate of the juvenile and adult migrations, highly variable marine locations and weak correlation between SAR and climate indicators. Overlapping age cohorts also reduces risk.

High: Populations exhibit either variable smolt timing behavior or a high correlation between SARs and climate indices, but not both.

Very high: Smolt and adult behavior is relatively consistent across time and populations, ***and*** there is a high correlation between SARs and climate indices.

Adult Freshwater Stage

Goal: Identify climate-limiting factors that affect the adult migration, holding, and pre-spawn stage

Questions for profile:

- Describe the freshwater adult migration, including distance, timing, holding patterns, and use of thermal refugia
- Are there imminent climate barriers to migration (temperature or flow) or anthropogenic factors that exacerbate climate stress?

Explanation:

For largely semelparous salmonids, survival through the adult migration to reach spawning grounds for a once-in-a-lifetime chance at reproduction is extremely important for population persistence. Pacific salmon typically have locally adapted phenology that

is most evident in this stage: migration timing is critically linked to exposure risk, such as high temperatures or high or low flows that can completely or partially block migration. Migration patterns typically reflect a trade-off between multiple pressures on both migration and spawn timing.

Environmental cues that trigger freshwater entry might also be disrupted by climate change. Fall rains, for example, which cue coho spawning in the northwest, might shift in intensity and timing, such that they are not as well matched to optimal migration timing. Changes in spring flows could also affect spring spawners such as steelhead.

The basic pattern of migration will determine the most relevant exposure factor and potential conflicts between multiple stressors. Qualitative assessment of the proximity to a climate-limiting factor will assist in interpreting the severity of risk from climate change. A description of anthropogenic stressors informs the likelihood of adaptation in this life stage, as well as the potential for management actions to improve climate resilience.

We assume that populations already experiencing climate stress are at greatest risk. If information about present climate stress is unavailable, we assume that fall/winter migrants are less vulnerable than spring/summer migrants. We further assume that long-distance migrants are more likely to encounter multiple stressors that constrain adaptation to climate change.

Response Bins

Low: Populations do not experience climate stress in the adult freshwater stage and have few known constraints on adaptation in response to climate change. Short-distance migrants that spend little time in freshwater before spawning are also at relatively low risk.

Moderate: Populations have a flexible adult freshwater migration period that appears minimally constrained by environmental factors.

High: Populations migrate or hold in shoulder periods around peak summer temperatures or low flows, or have long-distance migrations subject to multiple constraints on adaptation.

Very high: Adults migrate or hold through peak summer temperatures or face direct flow constraints on migration timing or holding locations (e.g., very limited deep pools). Populations at high risk for pre-spawn mortality are the most vulnerable in the adult freshwater stage.

Cumulative Life-Cycle Effects

Goal: This category is aimed specifically at cumulative risk to the DPS. This explicitly includes the risk that a potential change in life history as an adaptive response to climate change would entail a significant change in the character of the DPS.

Questions for profile:

- If this DPS adapted to climate change by avoiding a climate-related limit or threat, would the adaptation entail a change in the recognized life history strategy that characterizes the DPS?
- Are there other cumulative life cycle implications that have not been captured in other attributes?
- Is any individual life stage at such critical risk that it overrides all else in making this DPS highly vulnerable?

Explanation:

This attribute focuses on the cumulative summation of threats to the DPS. It is slightly different from identification of individual threats in each life stage in that it considers higher-level implications of climate change for the entire life cycle, such as loss of life history diversity. In some cases, populations might be unable to modify their behavior due to conflicting constraints that put them at high risk to any level of climate change. In other cases, although populations will likely modify their behavior as the environment changes, other important attributes might be lost. The DPS might ultimately lose its original distinctiveness and characteristics.

Response Bins

Low: Populations are not thought to be near a climate limit or are likely to adapt with minimal risk.

Moderate: Populations are not thought to be near a climate limit except under the most extreme scenarios, and could adapt without losing life history diversity.

High: Populations may be near a temperature- or flow-related limit, but are expected to adapt phenologically without loss of life history diversity within the DPS. Consequences of adaptation may not be fully known.

Very high: Populations are already critically near a climate limit for at least one life stage with no known ability to adapt without losing a life history type (assuming no major human intervention).

Hatchery Influence

Goal: Focus specifically on the impact of hatcheries on the resilience of salmonid populations

Questions for profile:

- Are production or conservation hatcheries present, and how prevalent are they?
- Describe current understanding of hatchery benefit or risk to the DPS.

Explanation:

Many Pacific salmon are reared in hatcheries to mitigate for other anthropogenic stressors that have reduced freshwater productivity. Hatchery programs are diverse and ever changing. Some hatcheries are managed as conservation tools intended to restore or maintain genetic and demographic variation, often while natural habitat is being restored. Temporary periods of unsuitable climate could be considered analogous to this role in concept. Others are production hatcheries intended to support fisheries with the intention of minimizing impact on populations of conservation concern.

Although in principle hatcheries can buffer wild populations from certain environmental stressors in freshwater, the overall role of hatcheries in enhancing climate resilience is not clear. For Pacific salmon, hatchery populations could be more susceptible to large-scale climate forcing than natural populations. This susceptibility results from the loss of behavioral, physiological, and adaptive genetic diversity over the long time horizons relevant with climate change (Lindley et al. 2009).

Moreover, hatchery and fishing practices may increase synchronization of population characteristics, which reduces the natural buffering of population variability against climate impacts. These practices can also select for traits that are maladaptive under a directionally changing climate, such as decreased size at age or migration timing that increases exposure to climate stress. Clearly the influence of hatchery operations on the capacity of salmonid populations for resilience to change in environmental conditions is variable, context dependent, and highly influenced by specific hatchery practices.

Where hatcheries have a detrimental impact on wild populations, they present a specific stressor that is not climate-related. On the other hand, they also provide a last-resort genetic reservoir if the wild population is driven to extinction by a prolonged period of poor climate.

We assume that conservation hatcheries practicing best-management procedures and high-quality monitoring have the greatest potential to reduce climate vulnerability.

However, unintended consequences from hatchery conditions are sufficiently likely that even the best hatchery cannot compensate completely for natural climate resilience. Thus we rank a full natural response as *low* in vulnerability and the best conservation hatchery as *moderate*, while production hatcheries increase vulnerability to *high* or *very high* ranks, depending on their prevalence.

Response Bins

Low: No hatchery-origin populations included in the DPS.

Moderate: Conservation hatcheries are present and using best-management practices and adequate monitoring to assess impacts on natural-origin fish within and among populations in the DPS.

High: Production hatcheries are present within the DPS or conservation hatcheries are present but lack monitoring programs to determine the impacts on natural-origin fish within and among populations in the DPS.

Very high: Production hatcheries dominate populations within the DPS.

Other Stressors

Goal: Characterize dominant threats to the DPS and elucidate specific anthropogenic threats that interact with climate drivers

Questions for profile:

- What are the major threats to this DPS?
- Which threats directly affect temperature or flow constraints?

Explanation:

Most U.S. salmon populations face a variety of anthropogenic threats in addition to climate change. These include habitat loss and degradation, toxic chemicals, pathogens endemic to fish culture, and displacement by invasive species. Salmon may also encounter simplified foodwebs, competition and hybridization in the wild with hatchery fish, obstructions to migration, and overfishing. All of these threats reduce population resilience by lowering demographic resilience, with smaller populations more prone to extinction from stochastic processes.

Stressors such as these usually reduce habitat diversity, as well as behavioral and genetic diversity that might otherwise facilitate adaptation to climate change. The sum of such stressors influences vulnerability to *any* additional stressor, including climate change. However, some of these stressors are more likely to act synergistically with

climate changes, as they interact directly with the ability of fish to adapt, while others do not interact directly with climate drivers. This distinction is not absolute, but rather simply to emphasize the most worrisome synergistic effects that otherwise might not be accounted for.

Thus we have two goals in assessing climate-related threats. The first is to rank the severity of the sum of other threats to a population relative to other West Coast salmon populations. This rank is already partially reflected in the population viability score. However, this section prioritizes the most important anthropogenic threats.

The second goal is to call out threats whose impacts are more likely to increase with climate change. In addition to affecting the population viability score, the sum of other threats provides information for the species narrative to aid future management decisions. Such information can help identify specific opportunities that might most improve resilience to climate change.

Adaptive responses to climate change include use of climate-buffered habitat such as floodplains and off-channel habitat, use of thermal refugia, or changes in migration timing. Some anthropogenic threats specifically affect these responses, such as loss of hydrologic connectivity (see Beechie et al. 2013 for a list of habitat alterations that directly affect temperature and flow). Other anthropogenic threats in this category include loss of riparian vegetation and run-off from impermeable surfaces, both of which tend to increase stream temperature and flash flows. Hatchery practices that homogenize or impose artificial selection on migration and spawn timing may also constitute climate-related anthropogenic threats, especially if they push traits in a direction that is non-adaptive in a directionally changing climate.

Freshwater fishing can also directly interact with the ability of fish to adapt to climate change: fish that select thermal refugia in very limited deep pools with cool water are often targeted by fisherman and face higher mortality as a result. Although not all of these interactions are fully understood at present, those that are better understood are more amenable to management actions that limit their impact.

In this profile, we attempt to differentiate between anthropogenic threats that specifically interact with adaptive climate behavior, and those that are most likely non-interacting stressors.

1. Threats that interact with climate drivers (temperature or flow).
   a. Habitat loss and degradation that affect temperature and flow, or availability of refugia such as floodplains, cold water pools, etc.
   b. Warm-adapted invasive species likely to gain advantage under higher temperatures (e.g., small-mouth bass, *Micropterus dolomieu*) or to limit use of

thermally preferable habitat

c. Fisheries in thermal refugia such as holding pools, and catch-and-release fisheries during warmer periods, when handling increases mortality

d. Diseases that are more virulent at higher temperatures, e.g., *Ceratomyxta shasta* infection (for some Pacific Northwest pathogen thermal sensitivities, see list in Hanson and Peterson 2014, Table 3)

e. Pesticides and contaminants with increased toxicity at higher temperatures (Laetz et al. 2014)

f. Other factors not mentioned above, with known climate interactions

2. Threats independent of climate drivers

    a. Loss, degradation, or limited access to other habitat that is similar climatically to existing habitat, or loss of usable habitat area rather than loss of habitat diversity or quality

    b. Disease introduction from farmed or hatchery fish, pollution, or water quality issues independent of temperature

    c. Ocean fisheries or fishing outside of thermal refugia or thermally stressful conditions

    d. Other factors not mentioned above, with no known climate interaction

Response Bins

Populations will be ranked from *low* to *very high* based on expert judgment given the severity of the cumulative threats. Those ranked very high must include some climate-interacting threats.

Low: Threats are relatively minor at present, and anticipated potential threats are likely to be alleviated by management actions.

Moderate: Threats are present in multiple categories but are relatively minor, or are potentially severe in only one category that is amenable to management action.

High: Threats are present in multiple categories, some of which are severe.

Very high: Threats are present in multiple categories, including anthropogenic factors that affect temperature or flow constraints, some of which are severe.

Population Viability

Goal: Characterize DPS resilience in terms of the current status of its component populations in a manner that differentiates among salmon DPSs.

Question for profile:

- How does the DPS rank in terms of its component population ratings for each of the four viable salmon population (VSP) parameters of abundance, productivity, diversity, and spatial structure?

Explanation:

The viable salmon population (VSP) concept was developed specifically to characterize conservation status and to inform specific objectives for recovery plans. This index characterizes the four VSP components of abundance, productivity, diversity, and spatial structure for Pacific salmon DPSs. In addition to characterizing overall extinction as an independent risk factor, VPS component scores also inform the status of populations in relation to specific aspects of climate resilience. High spatial structure and diversity scores in particular are likely to indicate greater climate resilience: spatial structure is related to the importance of habitat diversity for climate-buffering, while diversity reflects asynchrony in population dynamics, which stabilizes abundance at the meta-population or DPS level (the “portfolio” effect).

Overall VSP scores from regional technical recovery teams are available for all Pacific Northwest DPSs. In each case, VSP status of the DPS is expressed in terms of population-level ratings for all four components, with populations often being considered collectively in major population groups. Requirements for achieving a viable rating at the level of major population group reflect the historical number, relative size, life-history diversity, and spatial juxtaposition of component populations. At the DPS level, factors considered in establishing VSP criteria include the potential for catastrophic loss, for exposure to long-term demographic processes, and for the maintenance of long-term evolutionary potential.

Those criteria, or modified versions adopted in regional recovery plans, are used as a frame of reference in NOAA 5-year status reviews, and hence are regularly updated. The most recent 5-year review summary for each DPS should be the primary source for overall VSP ratings in scoring sensitivity for this attribute.

For DPSs not included in status reviews and that hence lack formally developed VSP ratings, expert judgment can be used for a qualitative ranking of DPS status within each attribute. Such rankings are not comparable to the extensive evaluation processes conducted for ESA-listed species. However, they can provide a best-available description of the relative risk of the DPS within each category.

Response Bins

Low: Extinction risk is low for this DPS at present.

Moderate: Extinction risk is low to moderate for this DPS at present.

High: Extinction risk is moderate to high for this DPS at present.

Very high: Extinction risk is high for this DPS.

Ocean Acidification Sensitivity

Goal: Estimate the sensitivity of a DPS to ocean acidification based on its relationship with "sensitive taxa." Note this scale was developed for all marine species by Hare et al. (2016), and is not salmon-specific.

Question for profile:

- Is this DPS directly dependent on a pH-sensitive species, or is it strongly correlated with the dynamics of these species?

Explanation:

Impacts of ocean acidification on marine organisms can vary considerably between taxa and species (Kroeker et al. 2013). Therefore, we estimate the impact of ocean acidification by examining DPS dependence on pH-sensitive taxa. For example, recent research shows a consistent negative impact of ocean acidification on calcifying organisms (e.g., pteropds). Therefore, species in this class, or those directly dependent on species in this class, should be considered more sensitive to changes in ocean pH. However, the extent to which prey-switching or compensation across other trophic levels will moderate impacts on salmon is uncertain (Busch et al. 2013, Sunday et al. 2017). We expect an increase in the volume of research into both direct and indirect effects of ocean acidification at higher trophic levels, so this attribute will be updated as new information becomes available in the near future.

The direct effect of ocean acidification on finfish is not well understood. Recent research suggests impacts on finfish DPSs will be most prevalent at the egg and early larval stages (Frommel et al. 2012, Frommel et al. 2014, Frommel et al. 2016). However, juvenile and adult olfaction and behavior may also be affected (Munday et al. 2009, Leduc et al. 2013, Munday et al. 2016, Porteus et al. 2018). Despite these studies, not enough is known to be able to predict which finfish DPSs will be more sensitive. This attribute will be updated when more information is available on which finfish DPSs are more likely to be directly impacted by ocean acidification.

Response Bins

Sensitive taxa are those that consistently show negative effects from ocean acidification, such as pteropods, mollusks, calcified algae, and echinoderms (Kroeker et al. 2013). Salmon are not sensitive taxa themselves, but some DPSs are more dependent on sensitive prey than others.

Low: Populations do not use sensitive taxa for food or habitat.

Moderate: Populations are somewhat reliant on sensitive taxa. The DPS utilizes sensitive taxa as either food or habitat, but can switch to non-sensitive taxa when necessary.

High: Populations rely on sensitive taxa. The DPS depends on sensitive taxa for either food or habitat and cannot switch to a non-sensitive alternative.

Very high: The DPS is a sensitive taxon. Note that this attribute definition was included for consistency with other Climate Vulnerability Assessments.

Literature Cited

Beechie, T., H. Imaki, J. Greene, A. Wade, H. Wu, G. Pess, P. Roni, J. Kimball, J. Stanford, P. Kiffney, and N. Mantua. 2013. Restoring salmon habitat for a changing climate. River Research and Applications **29**:939-960.

Busch, D. S., C. J. Harvey, and P. McElhany. 2013. Potential impacts of ocean acidification on the Puget Sound food web. ICES Journal of Marine Science **70**:823-833.

Davis, J. C. 1975. Minimal dissolved-oxygen requirements of aquatic life with emphasis on Canadian species -- review. Journal of the Fisheries Research Board of Canada **32**:2295-2332.

Frommel, A. Y., R. Maneja, D. Lowe, A. M. Malzahn, A. J. Geffen, A. Folkvord, U. Piatkowski, T. B. H. Reusch, and C. Clemmesen. 2012. Severe tissue damage in Atlantic cod larvae under increasing ocean acidification. Nature Climate Change **2**:42-46.

Frommel, A. Y., R. Maneja, D. Lowe, C. K. Pascoe, A. J. Geffen, A. Folkvord, U. Piatkowski, and C. Clemmesen. 2014. Organ damage in Atlantic herring larvae as a result of ocean acidification. Ecological Applications **24**:1131-1143.

Frommel, A. Y., D. Margulies, J. B. Wexler, M. S. Stein, V. P. Scholey, J. E. Williamson, D. Bromhead, S. Nicol, and J. Havenhand. 2016. Ocean acidification has lethal and sub-lethal effects on larval development of yellowfin tuna, Thunnus albacares. Journal of Experimental Marine Biology and Ecology **482**:18-24.

Hanson, K. C., and D. P. Peterson. 2014. Modeling the potential impacts of climate change on Pacific salmon culture programs: An example at Winthrop National Fish Hatchery. Environmental Management **54**:433-448.

Hare, J. A., W. E. Morrison, M. W. Nelson, M. M. Stachura, E. J. Teeters, R. B. Griffis, M. A. Alexander, J. D. Scott, L. Alade, R. J. Bell, A. S. Chute, K. L. Curti, T. H. Curtis, D. Kircheis, J. F. Kocik, S. M. Lucey, C. T. McCandless, L. M. Milke, D. E. Richardson, E. Robillard, H. J. Walsh, M. C. McManus, K. E. Marancik, and C. A. Griswold. 2016. A vulnerability assessment of fish and invertebrates to climate change on the northeast U.S. continental shelf. Plos One **11**:e0146756.

Kroeker, K. J., R. L. Kordas, R. Crim, I. E. Hendriks, L. Ramajo, G. S. Singh, C. M. Duarte, and J. P. Gattuso. 2013. Impacts of ocean acidification on marine organisms: Quantifying sensitivities and interaction with warming. Global Change Biology **19**:1884-1896.

Laetz, C. A., D. H. Baldwin, V. R. Hebert, J. D. Stark, and N. L. Scholz. 2014. Elevated temperatures increase the toxicity of pesticide mixtures to juvenile coho salmon. Aquat Toxicol **146**:38-44.

Leduc, A., P. L. Munday, G. E. Brown, and M. C. O. Ferrari. 2013. Effects of acidification on olfactory-mediated behaviour in freshwater and marine ecosystems: a synthesis. Philosophical Transactions of the Royal Society B-Biological Sciences **368**.

Lindley, S. T., C. B. Grimes, M. S. Mohr, W. Peterson, J. Stein, J. T. Anderson, L. W. Botsford, D. L. Bottom, C. A. Busack, T. K. Collier, J. Ferguson, J. C. Garza, A. M. Grover, D. G. Hankin, R. G. Kope, P. W. Lawson, A. Low, R. B. MacFarlane, K. Moore, M. Palmer-Zwahlen, F. B. Schwing, J. Smith, C. Tracy, R. Webb, B.

K. Wells, and T. H. Williams. 2009. What caused the Sacramento River fall Chinook stock collapse? , National Marine Fisheries Service, Southwest Fisheries Science Center.
Martin, B. T., A. Pike, S. N. John, N. Hamda, J. Roberts, S. T. Lindley, and E. M. Danner. 2017. Phenomenological vs. biophysical models of thermal stress in aquatic eggs. Ecology Letters **20**:50-59.
Munday, P. L., D. L. Dixson, J. M. Donelson, G. P. Jones, M. S. Pratchett, G. V. Devitsina, and K. B. Doving. 2009. Ocean acidification impairs olfactory discrimination and homing ability of a marine fish. Proceedings of the National Academy of Sciences of the United States of America **106**:1848-1852.
Munday, P. L., M. J. Welch, B. J. M. Allan, S. A. Watson, S. J. McMahon, and M. I. McCormick. 2016. Effects of elevated CO2 on predator avoidance behaviour by reef fishes is not altered by experimental test water. Peerj **4**.
Porteus, C. S., P. C. Hubbard, T. M. U. Webster, R. van Aerie, A. V. M. Canario, E. M. Santos, and R. W. Wilson. 2018. Near-future CO2 levels impair the olfactory system of a marine fish. Nature Climate Change **8**:737-+.
Roegner, G. C., J. A. Needoba, and A. M. Baptista. 2011. Coastal upwelling supplies oxygen-depleted water to the Columbia River estuary. PLoS ONE **6**:e18672.
Sunday, J. M., K. E. Fabricius, K. J. Kroeker, K. M. Anderson, N. E. Brown, J. P. Barry, S. D. Connell, S. Dupont, B. Gaylord, J. M. Hall-Spencer, T. Klinger, M. Milazzo, P. L. Munday, B. D. Russell, E. Sanford, V. Thiyagarajan, M. L. H. Vaughan, S. Widdicombe, and C. D. G. Harley. 2017. Ocean acidification can mediate biodiversity shifts by changing biogenic habitat. Nature Climate Change **7**:81-86.
