# Supplementary material for: Climate vulnerability assessment for Pacific salmon and steelhead in the California Current Large Marine Ecosystem
Source: PLoS One. 2019 Jul 24;14(7):e0217711. doi: 10.1371/journal.pone.0217711 (PMC6655584; doi:10.1371/journal.pone.0217711)
Supplement: S2 Appendix — (PDF) [file pone.0217711.s002.pdf]

S2 Appendix: Climate Exposure Factors

Supporting information for:

Climate vulnerability assessment for Pacific salmon and steelhead in the California Current Large Marine Ecosystem

Lisa G. Crozier, Michelle M. McClure, Tim Beechie, Steven J. Bograd, David A. Boughton, Mark Carr, Thomas D. Cooney, Jason B. Dunham, Correigh M. Greene, Melissa A. Haltuch, Elliott L. Hazen, Damon M. Holzer, David D. Huff, Rachel C. Johnson, Chris E. Jordan, Isaac C. Kaplan, Steven T. Lindley, Nathan J. Mantua, Peter B. Moyle, James M. Myers, Mark W. Nelson, Brian C. Spence, Laurie A. Weitkamp, Thomas H. Williams, Ellen Willis-Norton

S2 Appendix: Climate Exposure Factors 1
- Stream Temperature 2
- Summer Water Deficit 4
- Flooding 6
- Hydrologic Regime 9
- Sea Level Rise 11
- Sea Surface Temperature 12
- Ocean Acidification Exposure 14
- Upwelling 16
- Ocean Currents 19
- Literature Cited 21

Stream Temperature

Goal: Use spatially explicit projections of warming stream temperature to quantify the increase in thermal stress for each DPS.

Explanation:

Salmon freshwater residence time varies, and a key differentiating feature among populations is whether they spend summer in freshwater. For those that do, either as eggs, juveniles, or adults, high water temperature can be a strongly limiting feature.

Salmon have adapted to exploit heterogeneity in stream temperature, including the use of thermal refugia. However, productivity can be limited by the extent and distribution of refugia as well as by the general availability of aquatic habitat. Non-lethal effects of increased water temperature include less-efficient conversion of food to somatic growth and lower scope for strenuous behaviors such as migration or agonistic interactions with conspecifics.

In summer, weekly mean stream temperature often equilibrates with air temperature and humidity at broad spatial scales. Nonetheless, stream temperature is still highly heterogeneous within basins and even within individual streams. This heterogeneity is due to local factors such as vegetative cover and evapotranspiration, fog, snowmelt, groundwater inputs, water velocity, hydrologic stratification, and hyporheic exchange. These factors sometimes lower the sensitivity of streams to air temperature, which reduces the modeled impact of future increases in air temperature.

Isaak et al. (2016) used historical and future mean August stream temperatures in spatial statistical network models to model climate scenarios at 1-km resolution. We summarized their results by USGS hydrologic unit code at the 6-digit (accounting) level and averaged across each DPS as a whole.

August temperature is strongly correlated with temperature in other summer months, and hence is considered an index of summer temperature change in general. Historical stream temperatures are based on the 1993-2011 baseline. We used the Norwest modeled future scenario S30_2040D, which is based on the same 10 global climate model ensemble averages in the A1B warming trajectory scenario for 2030-2059. In other words, the same ensemble used for climate water deficit and regime shift.

For basins dominated by managed flows, water temperature depends much more on the presence or size of a cold pool in the reservoir and on the timing and quantity of water released. As water flows downstream, it tends to equilibrate with air temperature,

and the effect of the dam on temperature is reduced. Some dams with large stratified reservoirs, such as Shasta Dam, are specifically managed for downstream temperature control.

In the case of such managed flows, statistical models based on historical observations are not appropriate for projections under climate change. For example, the risk of exhausting the cold pool in Shasta Reservoir will increase with warming temperatures, especially during years with low snow accumulation. Unfortunately this risk is not fully represented in models appropriate for this assessment.

For the Sacramento River, we used two climate change scenarios produced for the 2008 Biological Assessment submitted by the U.S. Bureau of Reclamation to the National Marine Fisheries Service. Climate change scenarios were part of the Endangered Species Act consultation on long-term operations of the federal Central Valley Project and the California State Water Project (NMFS 2009).

We present the average of two scenarios (9.2 and 9.5) to represent a projection in the warmer and drier range, represented by the top 10% in mean annual temperature increase and the bottom 10% in change in annual precipitation, respectively. This projection was compared across 112 combinations of GCMs, emissions scenarios, and downscaling methods for the 2011-2040 period compared with a 1971-2000 baseline. This projection is from the United Kingdom Meteorological Office Hadley Centre coupled model, version 3 with emissions pathway A2. The GCM was downscaled using the bias-corrected spatial disaggregation method (Wood et al. 2004).

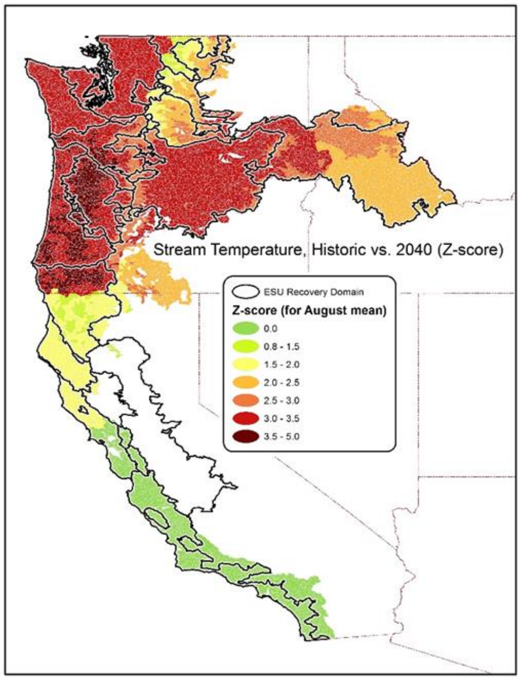

Figure S2-1. Change in stream temperature from 1993-2011 historic mean to 2040s, based on results from Isaak (2016).

Response Bins

Low: Z score <0.5

Moderate: Z score 0.5-1.5

High: Z score 1.5-2

Very high: Z score >2

Summer Water Deficit

Goal: Represent the risk of climate change on summer water availability for streams and ecosystems

Explanation:

Low summer flows effect salmon directly though drought stress and indirectly through effects on surrounding vegetation. Drought stress reflects an interaction between precipitation and temperature. Although projections from some global climate models show increases in precipitation, drought stress might still increase under these scenarios due to rising temperatures, which generate higher water demand from vegetation. During the low-flow season, streamflow, water depth, and water velocity often decrease as demand from plants for soil moisture increases. In arid areas, increased demand from plants may convert perennial streams to intermittent.

The summer water deficit or water balance deficit is defined by Littell et al. (2014) as the difference between potential and actual evapotranspiration by local vegetation, and is measured in total mm of water from June through August. The composite presented here reflects the average of 10 GCMs run under the A1B emissions scenario for the 2040s and compared to the historic period of 1916-2006.

An increasing water balance deficit intensifies stress on plants and heightens the risk of wildfire and of streams converting from perennial to intermittent. Such deficits also increase the potential for major changes in vegetation structure and function over time. Vegetation changes in turn affect stream flow, geomorphology, and stream temperature. Therefore, increases in water deficit will ultimately have negative effects on salmon that utilize streams in summer.

In the present climate, coastal Washington and Oregon have the lowest summer water deficit in the study domain, with no deficit in some places. The wettest areas of these states are expected to face somewhat fewer changes in the future than drier areas in the study domain.

Western Washington and high-elevation regions of Idaho also have relatively low water deficits at present. The southern portion of the interior Columbia River Basin and the Oregon Coast Range have intermediate summer water deficit.

In California, summer water deficit is high throughout most regions, with Northern California projected to become much drier. High levels of change are expected

across the latitudinal gradient from Puget Sound southward through the Oregon and Northern California Coast recovery domains. In time, the Willamette Valley may look more like the Klamath Basin and California's Central Valley.

Central and southern California are already very arid and may become somewhat drier. However, drying along coastal regions is offset somewhat by the moderating influence of the ocean and by seasonally dormant vegetation that reduces water demand in late summer.

Response Bins

Low: Z-score <0.5

Moderate: Z-score 0.5-1.5

High: Z-score 1.5-2

Very high: Z-score >2

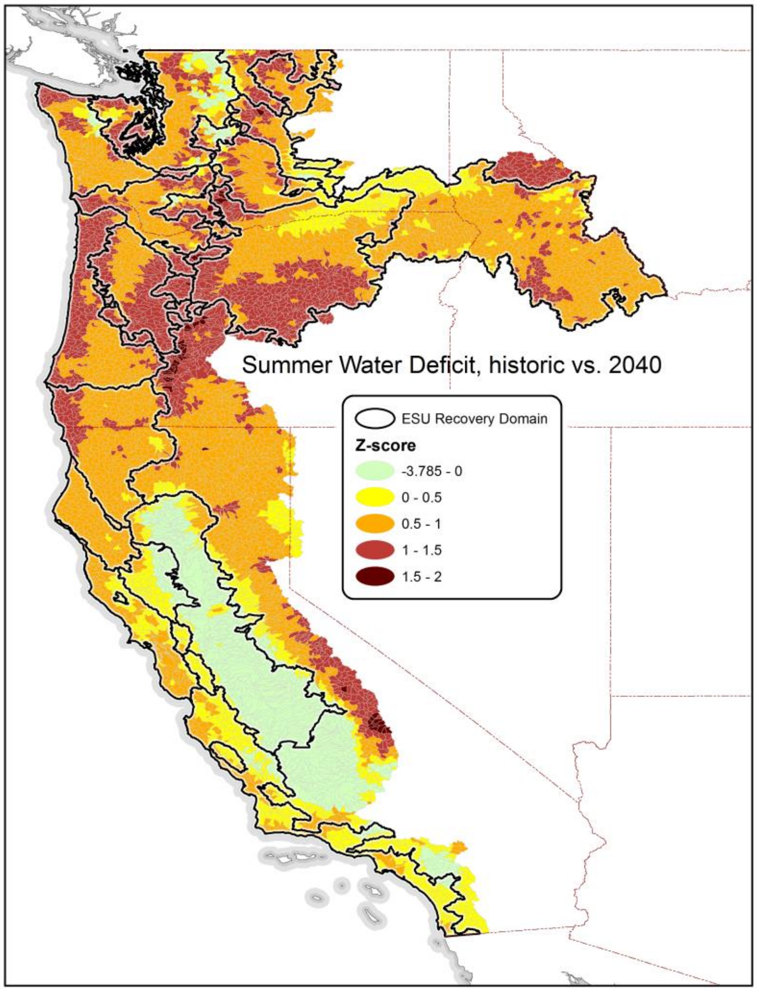

Figure S2-2. Change in summer water deficit from 1916-2006 historic mean to 2030 to 2059 future mean, based on results from Littell et al. (2014).

Flooding

Goal: Characterize a change in exposure to flood events.

Explanation:

Major flood events have the greatest potential impact during the incubation stage for most salmon populations. Flooding may also affect salmon at the juvenile stage, and for populations that spawn during late winter or spring, at the adult stage as well.

Flooding can have positive or negative impacts on a given population. Negative impacts occur when eggs are dislodged from protective gravel or when redds are suffocated by floodwater deposits of fine sediment. Substantial amounts of sediment bury salmon eggs and limit their oxygen supply. Floods that produce high water velocities may kill juveniles or displace them to unsuitable habitat downstream.

Positive effects of flooding occur when fine sediment is pushed out of spawning habitat or when habitat connectivity is increased by high flows that elevate water levels. In some cases, flooding recharges stream habitats by moving and depositing large woody debris or spawning gravel or by rejuvenating beneficial channel forms such as pool-riffle systems.

Generally large floods occur during major storm events, which are associated with atmospheric rivers that carry large quantities of warm, wet air from the Pacific Ocean (Warner et al. 2015). Such storms may substantially boost annual rainfall totals. Major storm events are associated with flooding in coastal or low-elevation mountain ranges and with large accumulations of snowpack at higher elevations. At mid elevations, major storms can cause large rain-on-snow events, which elevate the snowline.

Rising sea surface and atmospheric temperatures are expected to increase both the intensity and severity of major storm events. A change in the latitudinal temperature gradient, along with Hadley cell expansion associated with global warming, may shift the storm track northward. Such shifts may in turn change the latitudinal gradient of atmospheric rivers, which presently occur just south of the jet stream axis, thus altering the timing and intensity of coastal precipitation (Warner et al. 2015).

Warner et al. (2015) estimated mean projected change in water vapor transport by atmospheric rivers along the west coast. These estimates are based on the mean of 10 global climate models from the CMIP5 effort running the RCP 8.5 emissions scenario. Their models cover the historical period of 1970-1999 and the forecast period of

2070-2099. This forecast is further out than others considered here, but they provide a coarse estimate of potential change, where the direction and pattern of effects is of primary interest rather than the exact magnitude.

For these estimates, extreme events are calculated in terms of the 99th percentile in the amount of precipitation expected offshore; hence the forecasts are not influenced by local topography.

For interior populations, atmospheric rivers are usually not the primary driver of major floods. For these populations, we provide modeled flood frequency based on a dynamic downscaling method using the weather research and forecasting model (WRF). This model simulates both incoming atmospheric river events and the impact of local topography and other features. Estimates are more direct than changes in atmospheric rivers for overlapping areas, and also have a time period and emissions scenario more comparable with others considered here.

Flood estimates from (Salathé et al. 2014) are based on a regional climate model (WRF), comparing 1970-1999 with 2040-2069, running a single GCM (ECHAM5) under the CMIP3, A1B emissions scenario. The weather model then drives the variable infiltration capacity (VIC) hydrological model to simulate streamflow in historical and future climates. Expected 100-year flood magnitude is estimated using the generalized extreme value distribution.

For both of these metrics, we focus on change in extreme events, represented by the 99th percentile in precipitation or flooding. These are not amenable to standard transformations, and hence were left as a raw percent change for experts to rank from low to very high risk. We show the percent change expected in the 99th percentile rain event in the first case, and the ratio of the magnitude of a 100-year flood in the future climate compared to the historical climate.

Response Bins

Low: Relatively small change in flooding is projected, or spawning habitat for the DPS is not negatively influenced by flood events (e.g., more likely to increase snowpack in strongly snow-dominated basins, water recharge, or locations where flood events are beneficial).

Moderate: Moderate change in flood events is projected.

High: Large change in flood events is expected.

Very high: Large change in flood events and potentially severe effects on the DPS are expected.

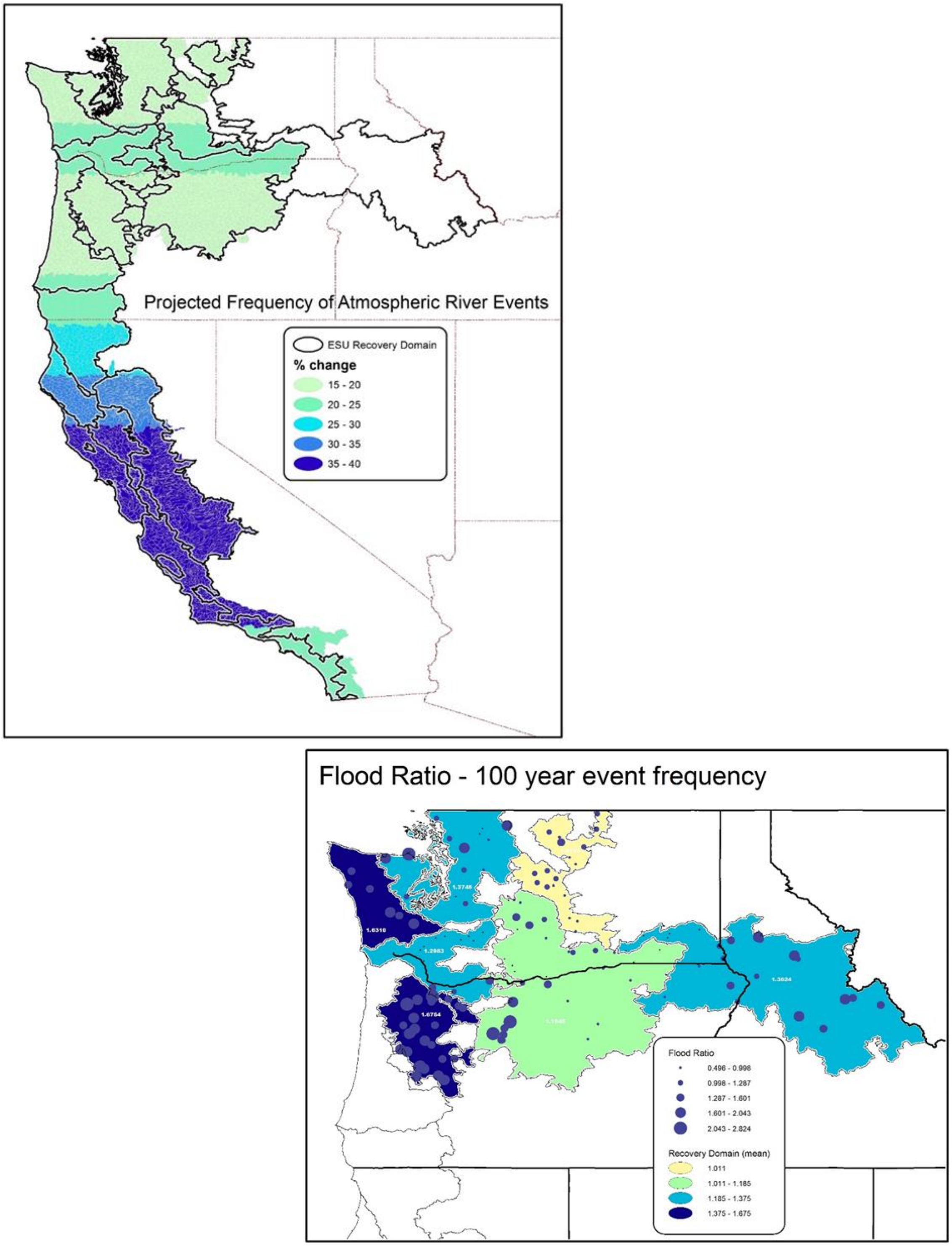

Figure S2-3. Map at upper left shows percent change in frequency of atmospheric river events between 1970-1999 and 2070-2099 (from Warner et al. 2015). Map at lower right shows flooding frequency between 1970-1999 and 2040-2069 (from Salathé et al. 2014).

Hydrologic Regime

Goal: Identify watersheds where the fundamental hydrologic regime may shift from snow-dominated to transitional or from transitional to rain-dominated

Explanation:

Salmon life histories reflect adaptation to many characteristics of local climate and freshwater habitat. One of the most fundamental of these characteristics is the seasonality of stream flows. Along the U.S. West Coast, most annual precipitation occurs in winter, resulting in higher flows during fall, winter, or spring. Dry seasons generally lead to minimum flows by late summer, although reservoir releases can sometimes alter this natural pattern.

The timing of high flow varies depending on whether winter precipitation falls predominately as rain or snow. In rain-dominated basins, peak flows occur in fall or winter, during the storm season; in snow-dominated basins, peak flows occur during the spring freshet. In transitional basins, high flows occur during major rain events in both fall and early winter, before temperatures have cooled sufficiently to produce snow consistently, and during spring, when the snow melts.

The primary index used to characterize hydrologic regime within a watershed is the ratio between snowpack on 1 April and cumulative precipitation from October through March (Hamlet et al. 2013). This index defines rain-dominant watersheds as those where less than 10% of cool-season precipitation is stored in snowpack as of 1 April. In transitional and snow-dominated basins respectively, 10-40% and over 40% of precipitation is stored in snowpack through 1 April.

To help assess and measure hydrologic regime shifts, Littell et al. (2014) constructed a gridded historic dataset that includes precipitation, temperature, and surface wind from 1915 through 2010. This composite dataset provides downscaled projections of climatologic scenarios for the western U.S. that reflect the average of 10 GCMs under the IPCC A1B emissions scenario for the 2040s (Littell et al. 2014).

For salmon, the crucial factor is whether the life history types expressed at present may become maladaptive in future climate scenarios. Because salmon life history types are strongly correlated with hydrologic regime type, we classify them by degree of exposure to shift. Populations that experience a major shift in hydrologic regime are classed as having high exposure, whereas those facing no regime shift are classed as having low exposure.

Note that change in the severity of precipitation events is captured in the *Flooding* exposure attribute, which measures storm frequency and flood magnitude rather than hydrologic regime type itself. The final component of exposure is determined by percentage of the basin affected, the extent to which regime shift impacts spawning areas, and the magnitude of the hydrologic shift.

Response Bins

Low: Populations experience no shift in regime type within or directly upstream from spawning habitat.

Moderate: Hydrologic regime is reclassified for headwater areas or for habitat within the range of some populations within the DPS, but the shift is unlikely to directly affect spawning habitat or the timing or intensity of spring freshets.

High: Hydrologic regime is reclassified over much of the basin, with high or unknown effects on spawning habitat.

Very high: Shifts in hydrologic regime occur in core spawning or rearing habitats.

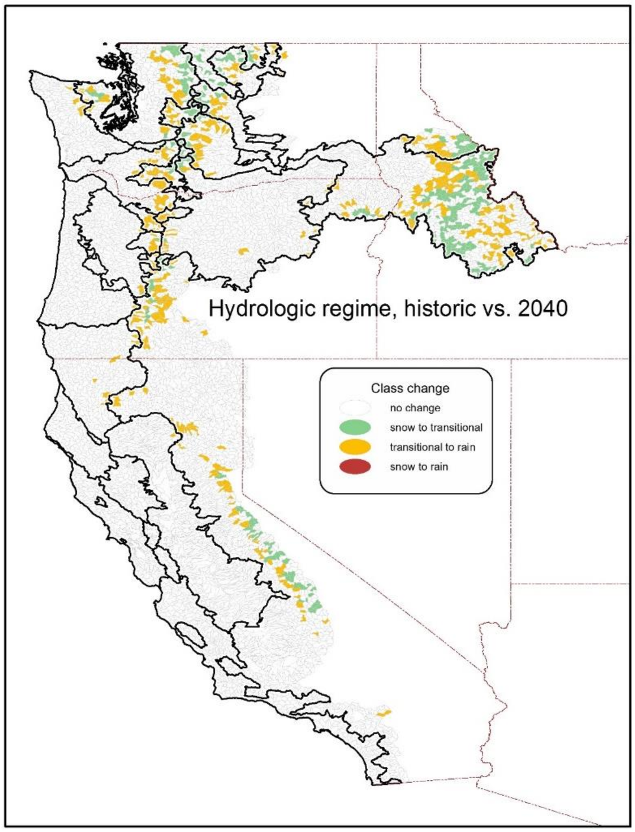

Figure S2-4. Regions expected to change hydrological regime from snow-dominated to transitional or from transitional to rain-dominated between the historic mean of 1915-2010 mean and the mean during 2013-2059 (Littell et al. 2014).

Sea Level Rise

Goal: Identify regions where sea level rise may reduce the availability and quality of marginal habitat that juvenile salmonids rely on.

Explanation:

With sea level rise, the sinuosity of estuarine margins will likely decrease, the tidal limit will move upstream, and salt marsh and brackish marsh areas will be flooded. Intense storms are expected to become more frequent, leading to more frequent sea level extremes (Cayan et al. 2008, Hansen et al. 2015). For example, the incidence of extreme water heights in San Francisco Bay is predicted to increase from 9 hours per decade to hundreds of hours per decade by 2050 (NRC 2012). Consequently, sea level rise will likely result in salinity intrusion and damage marginal ecosystems. This loss of complex estuarine habitat may reduce the quality and quantity of nursery habitat for juvenile salmonids.

Sea level rise occurs with climate change for two principal reasons: 1) ocean warming causes the expansion of seawater and 2) melting of land ice results in a freshwater influx to the ocean (NRC 2012). The current rate of sea level rise is 3.3 mm/yr (Hansen et al. 2015). Researchers agree there will be global sea level rise, but there is uncertainty in how rapidly it will occur, with predictions ranging 0.28-9 m by 2100 (NRC 2012, IPCC 2013, Hansen et al. 2015). The majority of this uncertainty stems from whether there will be nonlinear ice mass loss (NRC 2012).

Projections of sea level rise along the West Coast have also been estimated. In 2012 the National Research Council (NRC) predicted future sea level rise in California, Oregon, and Washington for the years 2030, 2050, and 2100 (NRC 2012). The NRC included in their models how sea level rise is affected by regional factors including: 1) ENSO events, 2) the rising and sinking of land along the coast, and 3) the proximity of Alaskan glaciers, which exert a gravitational pull on sea water.

Results are listed in Table 1. Sea level rise south of Cape Mendocino is similar to global projections, where the coast is sinking 1 mm/y; while north of Cape Mendocino projections are lower because much of the coast is rising (around 1.5-3.0 mm/y), causing seismic strain. If there were a large earthquake north of Cape Mendocino to reduce the strain, sea level could rise an additional 1-2 m above these projections.

In Puget Sound, sea level rise will likely result in a substantial loss of tidal flats unless former salt and brackish marshes are allowed to expand (Park et al. 1993). Land in

central and south Puget Sound is subsiding while land closer to the coast is rising (Miller et al. 2018). Therefore, toward the coast sea level is projected to increase from 0.09 to 0.51 meters and in the southern reaches of Puget Sound sea level is projected to rise from 0.58 to 1 meter by 2100 (Miller et al. 2018). There is a 0.1% probability of up to a 2.5 meter increase in sea level across Puget Sound by 2100 (Miller et al. 2018).

Table 1. Sea level rise projections (m) for the U.S. West Coast relative to the year 2000.

	2030	2050	2100
South of Cape Mendocino	0.04 - 0.3 m	0.12 - 0.61 m	0.42 - 1.67 m
North of Cape Mendocino	-0.04 - 0.23 m	-0.03 - 0.48 m	0.10 - 1.43 m

Response Bins

Low: Relatively low exposure to sea level rise for the DPS (e.g., the DPS is north of Cape Mendocino, or key habitat is not located in estuarine systems).

Moderate: Moderate exposure to sea level rise is projected.

High: High exposure to sea level rise is expected (e.g, DPS enters ocean south of Cape Mendocino).

Very High: High exposure to regional sea level rise in estuaries that are at low elevation.

Sea Surface Temperature

Goal: Quantify the DPS's exposure to changing sea surface temperatures in the California Current System using an ensemble of global climate models.

Explanation:

Changes in sea surface temperature (SST) have corresponded with differing marine survival rates for salmon DPSs. To characterize projections of future changes in SST, we used the Climate Change Web Portal of the NOAA Earth System Research Laboratory (www.esrl.noaa.gov/psd/ipcc/ocn/). We compared model output from 25 GCMs and earth system models on a 1° × 1° scale. In each grid cell, we quantified the magnitude of change as a z-score, defined as the difference between mean SST in the future (2006-2055) and historical simulations (1956-2005) divided by the average interannual standard deviation from the historical simulation. Resulting units were standard deviates.

Following Hare et al. (2016), tallies were placed in the *very high* bin if the future climate was more than 2 SDs different than the historical climate; *high* if the future climate was 1.5-2 SDs from the historical climate. In southern, central, and northern regions of the California Current System, the z-score for SST was always 2 or higher, indicating that DPSs found in those regions will have high or very high exposure to increasing SST by 2055.

Table 2. Z-scores for sea surface temperature averaged over the entire, southern, central or northern portions of the California Current System

	Entire CCS	Southern CCS	Central CCS	Northern CCS
Z-Score	2	2	2	2.1

Response Bins

Low: Z score <0.5

Moderate: Z score 0.5-1.5

High: Z score 1.5-2

Very High: Z score >2

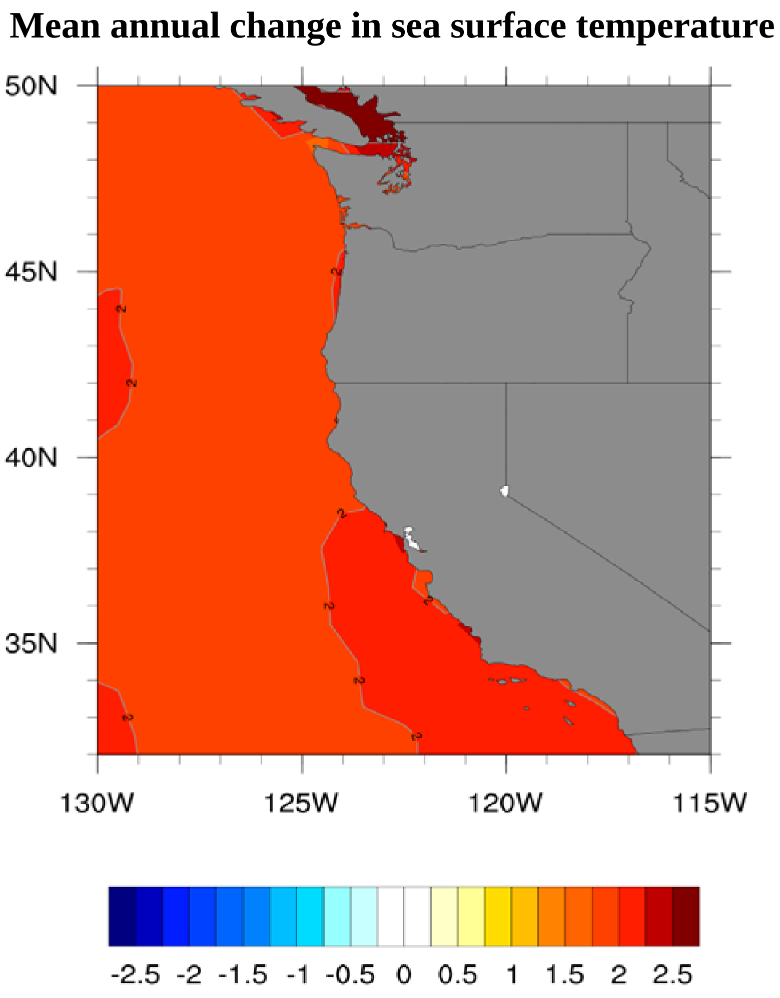

Ocean Acidification Exposure

Goal: Quantify DPS exposure to changing pH in the California Current System.

Explanation:

Ocean acidification is caused by rising atmospheric carbon dioxide concentrations, which reduce seawater pH and alter carbonate chemistry (IPCC 2013) The effects of ocean acidification on salmon species are uncertain. Indirect effects are possible through changes in food web structure and productivity. Additionally, juvenile salmonids often prey on marine arthropods and pteropods, which will likely be directly affected by increasing acidity.

We used the Climate Change Web Portal of the NOAA Earth Systems Research Laboratory to download ocean acidification (OA) projections to 2055 (https://www.esrl.noaa.gov/psd/ipcc/ocn/). An ensemble of 11 GCMs and earth system models on a 1° × 1° scale were used to calculate projected change in OA.

The metric used to quantify the magnitude of change was a z score: the difference between the mean OA in the future (2006-2055) and the mean OA from the historical simulation (1956-2005) divided by the average interannual standard deviation from the historical simulation. The units are standard deviates.

In the southern, central, and northern regions of the California Current, the z score for OA differed by at least 13 standard deviates from the historical OA concentrations. For this climate vulnerability assessment, a z score of 2 or higher indicates the DPS will have a *very high* exposure to increasing OA concentrations by 2055. Therefore, all DPSs were scored as having *very high* exposure to changes in OA.

Table 3. Z scores for ocean acidification averaged over the entire, southern, central or northern portions of the California Current System.

	Entire CCS	Southern CCS	Central CCS	Northern CCS
Z score	-14.4	-13.5	-14.8	-14.8

Response Bins

Low: Z score < 0.5

Moderate: Z score 0.5-1.5

High: Z score 1.5-2

Very High: Z score >2

Annual change in pH

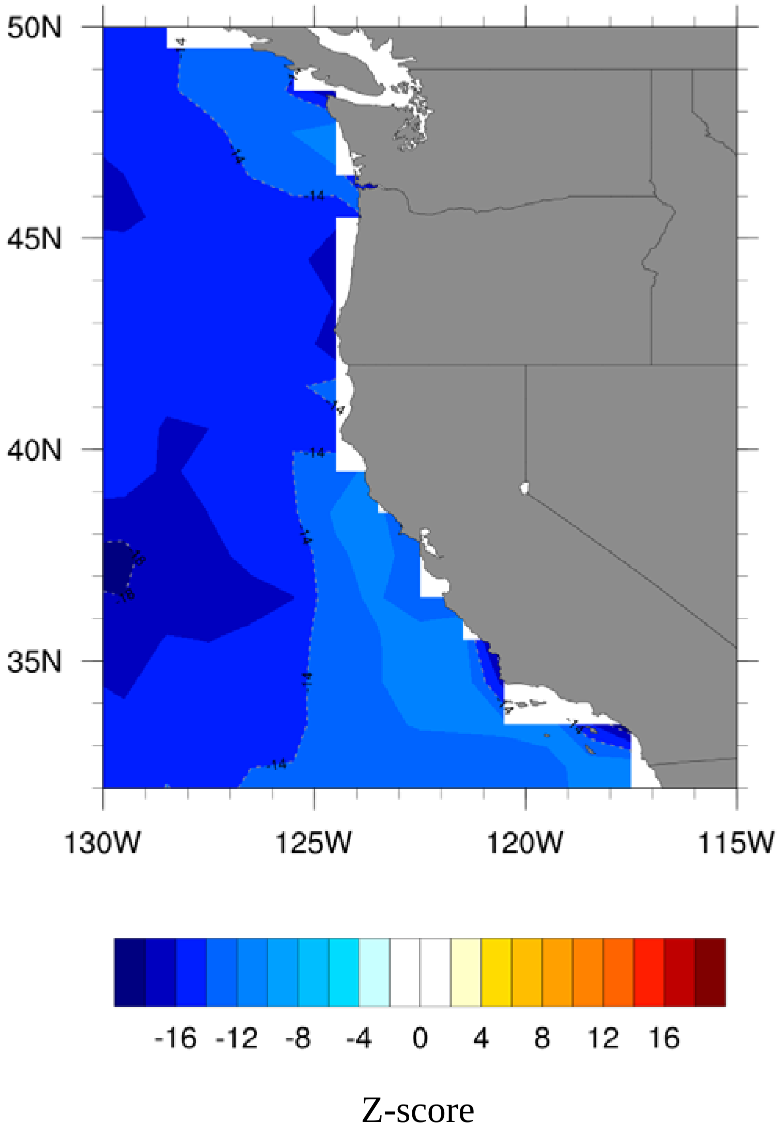

Upwelling

Goal: Characterize DPS exposure to changes in upwelling-favorable winds and the phenology of upwelling.

Explanation:

Upwelling occurs when equatorward, alongshore winds drive offshore Ekman transport, and surface waters are replaced with nutrient-rich deep waters. The input of nutrient-rich water to the euphotic zone promotes high levels of primary productivity, and Eastern Boundary Upwelling Systems such as the California Current System (CCS) support nearly 20% of global fish catch, despite occupying less than 1% of the surface area of the global ocean (Pauly and Christensen 1995, Mote and Mantua 2002).

There is natural variability in the timing and strength of coastal upwelling along the meridional extent of the California Current. The timing of the "spring transition" to upwelling conditions varies latitudinally, with a nearly year-round climatological upwelling season at the southern end of the California Current and a progressively later transition and shorter upwelling season at the more northerly latitudes (Strub and James 1988, 2000, Bograd et al. 2002, Bograd et al. 2009).

If upwelling intensifies, increased nutrient input could stimulate added productivity; however, too much wind will increase turbulence and offshore transport of surface waters, reducing nearshore productivity. Conversely, reduced upwelling would decrease input of nutrients to the euphotic zone. Many organisms in the CCS have life history traits (foraging, reproduction, migration) adapted to the present timing of annual upwelling events (Bograd et al. 2009). Thus, a delayed or early start to spring-summer upwelling may result in a temporal mismatch between predator needs and prey availability (Bograd et al. 2009, Bakun et al. 2015).

Upwelling intensity is correlated with large-scale modes of climate variability such as the El Niño Southern, Pacific Decadal, and North Pacific Gyre Oscillations (ENSO, PDO, and NPGO). On longer timescales, several possible outcomes have been proposed regarding changes in CCS upwelling. Bakun (1990) suggested that climate change would heat the continents faster than the oceans, steepening cross-shore air-pressure gradients and intensifying upwelling-favorable winds. Several other modeling studies have also forecast intensified upwelling winds under climate change (Snyder et al. 2003, Auad et al. 2006), and one meta-analysis found support for increased CCS upwelling since 1950 (Sydeman et al. 2014). However, time series forecast to date have been too short to differentiate between natural climate variability and anthropogenic

forcing (Sydeman et al. 2014).

A change to the phenology of upwelling refers to potential shifts in the seasonal cycle of upwelling (Bograd et al. 2009). From 1967 to 2007 there has been a trend toward later spring transition dates and shorter upwelling seasons in the northern CCS and longer upwelling seasons in the southern CCS. These trends may indicate the direction of future change (Bograd et al. 2009).

Delayed and weakened upwelling in the central CCS during El Niño years (Bograd et al. 2009), along with the frequency of extreme El Niño events, are predicted to double with climate change (Cai et al. 2014). Furthermore, positive phases of the NPGO appear to be associated with an earlier start to the upwelling season in the central CCS (Chenillat et al. 2012). However, the current set of climate models are not able to project changes to the NPGO, so it is difficult to predict how the onset of the spring-summer upwelling period will be affected.

The most recent studies suggest that the Bakun hypothesis is oversimplified, and changes in upwelling will be season, region, and latitude dependent. The most recent models suggest that in the northern CCS, there will be an expansion of the upwelling season by 1-2 days per decade and a modest increase in upwelling during spring (Rykaczewski et al. 2015, Wang et al. 2015). In both the central and southern CCS, upwelling is projected to increase in April but decrease during June-September (Figure S2 5;Rykaczewski et al. 2015, Wang et al. 2015).

Rykaczewski et al. (2015) predicted an average decrease of 8% (±10% SD) in upwelling favorable winds across the CCS. Wang et al. (2015) and Rykaczewski et al. (2015) were the first to use an ensemble of over 20 coupled atmosphere-ocean general circulation models; therefore, they are at present the most reliable predictions of climate change impacts on upwelling favorable winds in the CCS. However, uncertainty in these predictions remains because the ensembles included relatively coarse-resolution global models from which it is difficult to resolve local dynamics in the CCS.

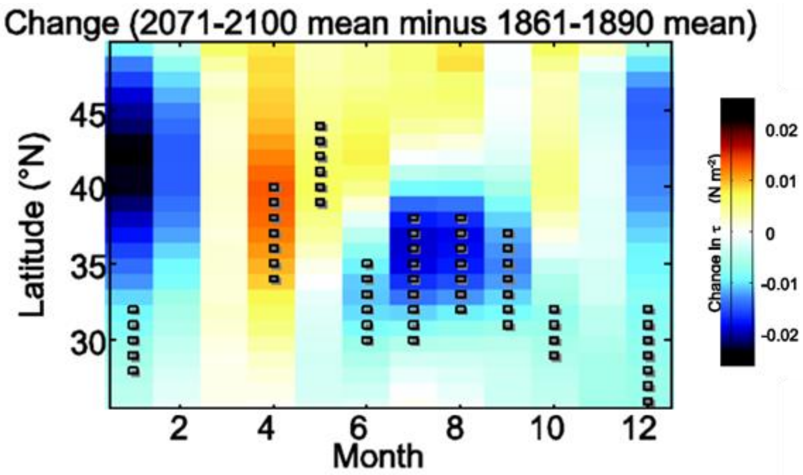

Figure S2-5: Hovmöller diagram of the change in upwelling favorable winds between the two periods (2071-2100 mean minus 1861-1890 mean) for the CCS (Rykaczewski et al. 2015).

Response Bins

Low: Relatively low exposure to a change in upwelling favorable winds.

Moderate: Moderate exposure to a change in upwelling favorable winds is projected.

High: High exposure to a change in upwelling favorable winds is expected.

Very High: High exposure to a change in upwelling favorable winds and potentially severe effects on the DPS are expected.

Ocean Currents

Goal: Establish DPS exposure to changing ocean currents in the California Current System.

Explanation:

There are three principal circulation features in the California Current System: the shallow, equatorward-flowing *California Current*, the deep, poleward-flowing *California Undercurrent*, and the winter-time, poleward-flowing *Davidson Current* (Hickey 1998). The slow, broad California Current moves cool, low-salinity, and nutrient rich waters equatorward year-round (Figure S2-5). The California Current flows about 50-1000 km offshore in the upper 100 m (Hickey 1998). Directly south of Point Conception, a portion of the current turns north and becomes the Southern California Countercurrent, which in summer does not make it completely north and becomes the biologically rich, self-contained Southern California Eddy (Lynn and Simpson 1987, Hickey 1998, King et al. 2011).

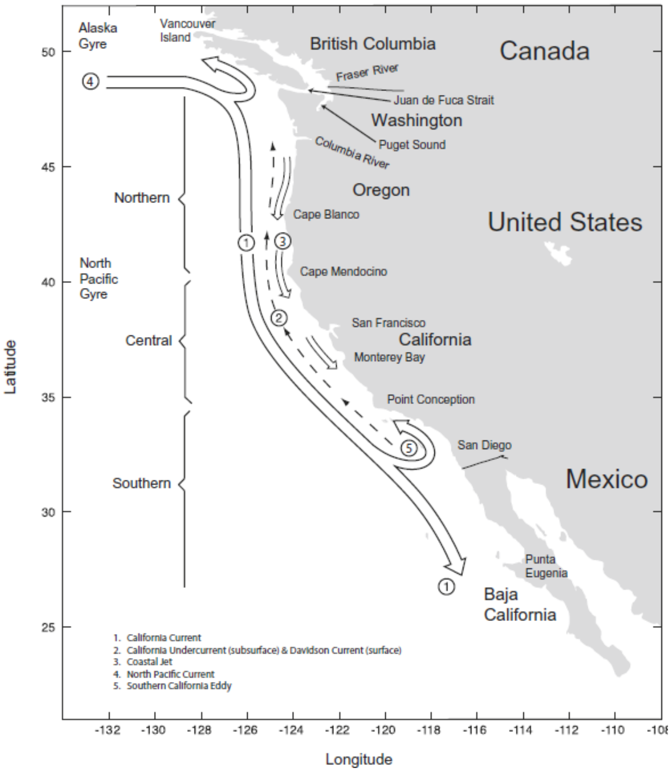

Figure S2-6. From Checkley et al (2009).

The narrow and deep (100-300 m) California Undercurrent brings high-salinity, warm, low-oxygen waters from Baja California northward to Vancouver Island (King et al. 2011). In winter, the Davidson Current also flows poleward from Point Conception to Vancouver Island. Equatorward flow in the California Current is driven by large-scale wind forcing (Checkley and Barth 2003). Therefore, changes in flow are driven by fluctuations in the strength of basin-scale wind forcing (Cummins and Freeland 2007). Increases in wind speed could increase current strength and subsequently increase eddy activity. The strength of the California Current is also driven by fluctuations in the strength of the

North Pacific Current, which affects the amount of water transported respectively into the Alaskan Gyre and California Current (Cummins and Freeland 2007). Furthermore, California Current strength is affected by El Niño Southern Oscillation, Pacific Decadal Oscillation, and North Pacific Gyre Oscillation. Positive, or warm phases of ENSO, PDO, and NPGO are associated with a weaker California Current and vice versa (Chelton et al. 1982, King et al. 2011). There has not been a clear trend of strengthening or weakening currents in the California Current System. However, from 1992a small but statistically significant increase has been observed in eddy kinetic energy in the Eastern North Pacific (O'Donnell 2015).

In global climate models, the combination of substantial natural variability with high uncertainty in predicted nearshore zonal winds makes it difficult to determine future impacts of climate change, either in circulation features of the California Current System or in ocean/climate indices such as the ENSO, PDO, and NPGO (King et al. 2011). Currents in the system may change in location and intensity. For example, the Northern Hemisphere Hadley Cell is predicted to expand poleward, which may result in a poleward displacement of zonal currents (Bakun 1990, Francis et al. 1998). However, other predictions indicate that there will not be dramatic change in the structure of major oceanic currents in the California Current System (Stouffer et al. 2006, King et al. 2011).

Response Bins

Low: Relatively low exposure to a change in ocean currents is projected.

Moderate: Moderate exposure to a change in in ocean currents is expected

High: High exposure to a change in in ocean currents is expected.

Very High: High exposure to a change in ocean currents and potentially severe effects on the DPS are expected

Literature Cited

Auad, G., A. Miller, and E. Di Lorenzo. 2006. Long-term forecast of oceanic conditions off California and their biological implications. Journal of Geophysical Research **111**.

Bakun, A. 1990. Global climate change and intensification of coastal ocean upwelling. Science **247**:198-201.

Bakun, A., B. A. Black, S. J. Bograd, A. P. Garcia-Reyes, A. J. Miller, R. R. Rykaczewski, and W. J. Sydeman. 2015. Anticipated effects of climate change on coastal upwelling ecosystems. Current Climate Change Reports **1**:85-93.

Bograd, S. J., I. D. Schroeder, N. Sarkar, X. Qiu, W. J. Syderman, and F. B. Swchwing. 2009. Phenology of coastal upwelling in the California Current. Geophysical Research Letters **36**:1-5.

Bograd, S. J., F. Schwing, R. Mendelssohn, and P. Green-Jessen. 2002. On the changing seasonality over the North Pacific. Geophysical Research Letters **29**:1-4.

Cai, W., S. Borlace, M. Lengaigne, P. Van Rensch, M. Collins, G. A. Vecchi, and M. H. England. 2014. Increasing frequency of extreme El Niño events due to greenhouse warming. Nature Climate Change **4**:111-116.

Cayan, D. R. B., P.D., K. Hayhoe, M. Tyree, M. D. Dettinger, and R. E. Flick. 2008. Climate change projections of sea level extremes along the California coast. Climatic Change **87**:57-73.

Checkley, D. M., and J. A. Barth. 2003. Patterns and processes in the California Current System. Progress in Oceanography **83**:49-64.

Chelton, D. B., P. A. Bernal, and J. A. McGowan. 1982. Large-scale interannual physical and biological interaction in the California Current. Journal of Marine Research **40**:1095-1125.

Chenillat, F., P. Riviere, X. Capet, E. Di Lorenzo, and B. Blanke. 2012. North Pacific Gyre Oscillation modulates seasonal timing and ecosystem functioning in the California Current upwelling system. Geophysical Research Letters **39**.

Cummins, P. F., and H. J. Freeland. 2007. Variability of the North Pacific Current and its bifurcation. Progress in Oceanography **75**:253-265.

Francis, R. C., S. R. Hare, A. B. Hollowed, and W. S. Wooster. 1998. Effects of interdecadal climate variability on the oceanic ecosystems of the NE Pacific. Fisheries Oceanography **7**:1-21.

Hamlet, A. F., M. M. Elsner, G. S. Mauger, S. Y. Lee, I. Tohver, and R. A. Norheim. 2013. An overview of the Columbia Basin climate change scenarios project: approach, methods, and summary of key results. Atmosphere-Ocean **51**:392-415.

Hansen, J., M. Sato, P. Hearty, R. Ruedy, M. Kelley, V. Masson-Delmotte, and I. Velicogna. 2015. Ice melt, sea level rise and superstorms: evidence from paleoclimate data, climate modeling, and modern observations that 2◦ C global warming is highly dangerous. Atmospheric Chemistry and Physics Discussions **15**:20059-20179.

Hare, J. A., W. E. Morrison, M. W. Nelson, M. M. Stachura, E. J. Teeters, R. B. Griffis, M. A. Alexander, J. D. Scott, L. Alade, R. J. Bell, A. S. Chute, K. L. Curti, T. H. Curtis, D. Kircheis, J. F. Kocik, S. M. Lucey, C. T. McCandless, L. M. Milke, D.

E. Richardson, E. Robillard, H. J. Walsh, M. C. McManus, K. E. Marancik, and C. A. Griswold. 2016. A vulnerability assessment of fish and invertebrates to climate change on the Northeast U.S. Continental Shelf. PLoS ONE **11**:e0146756.

Hickey, B. A. 1998. Western North America, tip of Baja California to Vancouver Island. Pages 345–394 *in* K. Brink and A. Robinson, editors. The Sea. Wiley, New York, NY.

IPCC. 2013. Climate Change 2013: The Physical Science Basis. Contribution of Working Group I to the Fifth Assessment Report of the Intergovernmental Panel on Climate Change. Cambridge University Press. Available from: http://www.climatechange2013.org/ Cambridge, United Kingdom and New York, NY, USA.

Isaak, D. J. 2016. Norwest: regional database and modeled stream temperature. Data tool of the U.S. Department of Agriculture and U.S. Forest Service. Available from www.fs.fed.us/rm/boise/AWAE/projects/NorWeST.html (March 2016).

King, J. R., V. N. Agostini, C. J. Harvey, G. A. McFarlane, M. G. Foreman, J. E. Overland, and K. Y. Aydin. 2011. Climate forcing and the California Current ecosystem. Ices Journal of Marine Science **68**:1199-1216.

Littell, J. S., G. S. Mauger, E. P. Salathé, A. F. Hamlet, S. Lee, M. R. Stumbaugh, M. Elsner, R. Norheim, E. R. Lutz, and N. J. Mantua. 2014. Uncertainty and extreme events in future climate and hydrologic projections for the Pacific Northwest: providing a basis for vulnerability and core/corridor assessments. Report of the University of Washington College of the Environment Climate Impacts Group to the Final report U.S. for Department of the Interior Pacific Northwest Climate Science Center. Available from http://cses.washington.edu/db/pubs/abstract825.shtml (May 2015).

Lynn, R. J., and J. J. Simpson. 1987. The California Current System: The seasonal variability of its physical characteristics. Journal of Geophysical Research **92**:12947.

Miller, I. M., H. Morgan, G. Mauger, T. Newton, R. Weldon, D. Schmidt, M. Welch, and E. Grossman. 2018. Projected Sea Level Rise for Washington State – A 2018 Assessment. A collaboration of Washington Sea Grant, University of Washington Climate Impacts Group, Oregon State University, University of Washington, and US Geological Survey. Prepared for the Washington Coastal Resilience Project.

Mote, P. W., and N. J. Mantua. 2002. Coastal upwelling in a warmer future. Geophysical Research Letters **29**:1-4.

NMFS, National Marine Fisheries Service. 2009. Biological Opinion on the Operating Criteria and Plan (OCAP) for the long-term operations of the Central Valley Project and State Water Project with recommendations for Essential Fish Habitat. Page 844. Sacramento Protected Resources Division.

NRC, Committee on Protection and Management of Pacific Northwest Anadromous Salmonids. 1996. Upstream : salmon and society in the Pacific Northwest. National Academies Press, Washington, D.C.

NRC, National Research Council. 2012. Sea-Level Rise for the Coasts of California, Oregon, and Washington: Past, Present, and Future. The National Academies Press Available at: http://www.nationalacademies.org/nrc/.

O'Donnell, C. J. 2015. Global trends in eddy kinetic energy from satellite altimetry

University of East Anglia.
Park, R. A., J. K. Lee, and D. J. Canning. 1993. Potential effects of sea-level rise on Puget Sound wetlands. Geocarto International **8**: 99-110.
Pauly, D., and V. Christensen. 1995. Primary production required to sustain global fisheries. Nature **374**:255-257.
Rykaczewski, R. R., J. P. Dunne, W. J. Sydeman, M. García-Reyes, B. A. Black, and S. J. Bograd. 2015. Poleward displacement of coastal upwelling-favorable winds in the ocean's eastern boundary currents through the 21st century. Geophysical Research Letters **42**:6424-6431.
Salathé, E. P., Jr., A. F. Hamlet, C. F. Mass, S.-Y. Lee, M. Stumbaugh, and R. Steed. 2014. Estimates of twenty-first-century flood risk in the Pacific Northwest based on regional climate model simulations. Journal of Hydrometeorology **15**:1881-1899.
Snyder, M. A., L. C. Sloan, N. S. Diffenbaugh, and J. L. Bell. 2003. Future climate change and upwelling in the California Current. Geophysical Research Letters **30**:1-4.
Stouffer, R. J., J. Yin, J. M. Gregory, K. W. Dixon, M. J. Spelman, W. Hurlin, ..., and A. Hu. 2006. Investigating the causes of the response of the thermohaline circulation to past and future climate changes. Journal of Climate **19**:1365-1387.
Strub, P. T., and C. James. 1988. Atmospheric conditions during the spring and fall transitions in the coastal ocean off western United States. Journal of Geophysical Research **93**:15561-15584.
Strub, P. T., and C. James. 2000. Altimeter-derived variability of surface velocities in the California Current System: Part 2. Seasonal circulation and eddy statistics. Deep Sea Research II **47**:831-870.
Sydeman, W. J., M. García-Reyes, D. S. Schoeman, R. R. Rykaczewski, S. A. Thompson, B. A. Black, and S. J. Bograd. 2014. Climate change and wind intensification in coastal upwelling ecosystems. Science **345**:77-80.
Wang, D., T. C. Gouhier, B. A. menge, and A. R. Gunguly. 2015. Intensification and spatial homogenization of coastal upwelling under climate change. Nature Climate Change **518**:390-394.
Warner, M., C. F. Mass, and E. P. Salathé. 2015. Changes in winter atmospheric rivers along the North American west coast in CMIP5 climate models. J. Hydrometeorology **16**:118-128.
Wood, A. W., L. R. Leung, V. Sridhar, and D. P. Lettenmaier. 2004. Hydrologic implications of dynamical and statistical approaches to downscaling climate model outputs. Climatic Change **62**:189-216.
