# Supplementary material for: Climate vulnerability assessment for Pacific salmon and steelhead in the California Current Large Marine Ecosystem
Source: PLoS One. 2019 Jul 24;14(7):e0217711. doi: 10.1371/journal.pone.0217711 (PMC6655584; doi:10.1371/journal.pone.0217711)
Supplement: S3 Appendix — (PDF) [file pone.0217711.s003.pdf]

S3 Appendix: Distinct population segment scores and narratives

Supporting information for: *Climate vulnerability assessment for Pacific salmon and steelhead in the California Current Large Marine Ecosystem*

Lisa G. Crozier, Michelle M. McClure, Tim Beechie, Steven J. Bograd, David A. Boughton, Mark Carr, Thomas D. Cooney, Jason B. Dunham, Correigh M. Greene, Melissa A. Haltuch, Elliott L. Hazen, Damon M. Holzer, David D. Huff, Rachel C. Johnson, Chris E. Jordan, Isaac C. Kaplan, Steven T. Lindley, Nathan J. Mantua, Peter B. Moyle, James M. Myers, Mark W. Nelson, Brian C. Spence, Laurie A. Weitkamp, Thomas H. Williams, Ellen Willis-Norton

For each distinct population segment (DPS), we report final ranks for overall vulnerability, biological sensitivity and climate exposure, with respective bootstrap results. Bootstrap results show the percentage of draws that fell into each vulnerability category. Note that it is possible for both sensitivity and exposure bootstrap results to produce a majority of draws that qualify as ***very high***, without the final rank qualifying as ***very high***, because overall rank was based on actual scores rather than replicate draws. We also report the adaptive capacity score and a summary of the data quality. Within each table, we show mean score and data quality for each attribute, as well as a graphical representation of the score distribution.

We then provide a synopsis of life history characteristics and of the major ways in which climate was expected to influence abundance and distribution for each DPS. We then discuss scores for the three extrinsic attributes of *population viability, hatchery influence, and other stressors*, recounting the key factors that led to these scores. Finally, we discuss the potential in each DPS for adaptive capacity. Literature cited in these narratives is not exhaustive: previous literature reviews, original listing documents, and updated status reviews contain many additional references. Literature cited here is meant only to highlight important sources that influenced particular scores. DPSs are ordered by species and from south to north.

Contents

Chinook Salmon (*Oncorhynchus tshawytscha*) 3
Sacramento River winter-run Chinook 3
Central Valley spring-run Chinook 10
Central Valley fall/late fall-run Chinook 15
California Coastal Chinook 20
Upper Willamette River Chinook 26
Middle Columbia River spring-run Chinook 30
Snake River spring/summer-run Chinook 36
Snake River fall-run Chinook 42
Upper Columbia River spring-run Chinook 46
Lower Columbia River Chinook 50
Puget Sound Chinook 55

Coho Salmon (*Oncorhynchus kisutch*) 60
Central California Coast coho 60
Southern Oregon/Northern California Coast coho 66
Oregon Coast coho 71
Lower Columbia River coho 75
Puget Sound coho 79

Chum Salmon (*Oncorhynchus keta*) 84
Columbia River chum 84
Hood Canal summer-run chum 87
Puget Sound chum 91

Pink Salmon (*Oncorhynchus keta*) 95
Puget Sound pink (odd- and even-year pink combined) 95

Sockeye Salmon (*Oncorhynchus nerka*) 99
Snake River sockeye 99
Lake Ozette sockeye 103

Steelhead (*Oncorhynchus mykiss*) 106
Southern California Coast steelhead 106
South Central California Coast steelhead 111
California Central Valley steelhead 116
Central California Coast steelhead 120
Northern California steelhead 124
Lower Columbia River steelhead 129
Upper Willamette River steelhead 133
Middle Columbia River steelhead 139
Snake River Basin steelhead 143
Upper Columbia River steelhead 148
Puget Sound steelhead 153

Chinook Salmon (*Oncorhynchus tshawytscha*)

Sacramento River winter-run Chinook

Overall vulnerability—Very high (100% Very high)
Biological sensitivity—Very high (100% Very high)
Climate exposure—High (86% High, 14% Very high)
Adaptive capacity—Low (1.4)
Data quality—79% of scores ≥ 2

	Sacramento River winter-run Chinook	Expert Scores	Data Quality	Expert Scores Plots (Portion by Category)
Sensitivity attributes	Early life history	4	2.8	
	Juvenile freshwater stage	3.7	2.8	
	Estuary stage	3.5	2	
	Marine stage	3.5	2.3	
	Adult freshwater stage	2.9	3	
	Cumulative life-cycle effects	4	2.5	
	Hatchery influence	2	3	
	Other stressors	3.9	1.8	
	Population viability	3.9	3	
	Ocean acidification sensitivity	1.8	1	
	Sensitivity Score	Very High		
Exposure variables	Stream temperature	3.1	2	
	Summer water deficit	2.7	1.8	
	Flooding	3.2	1.3	
	Hydrologic regime	2.6	1.8	
	Sea level rise	3.5	2.3	
	Sea surface temperature	3.4	2	
	Ocean acidification exposure	4	2.5	
	Upwelling	2.9	1	
	Ocean currents	1.9	1	
	Exposure Score	High		
Overall Vulnerability Rank		Very High		

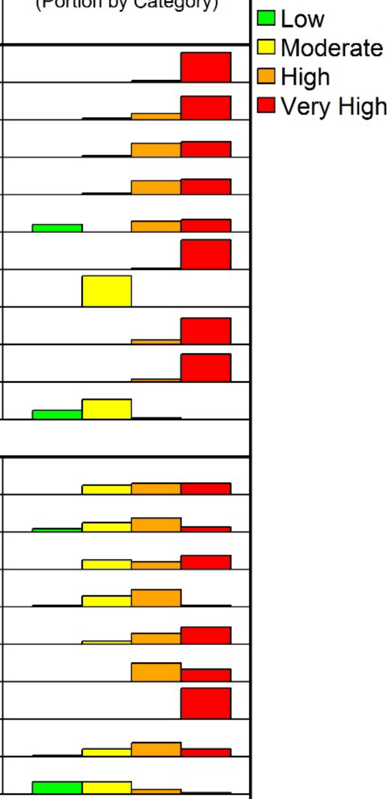

Life History Synopsis

Sacramento River winter-run Chinook adults leave the ocean before fully mature, from December through April, and migrate predominantly as age-3 fish (O'Farrell et al. 2012; Satterthwaite et al. 2017). Adults hold in fresh water until spawning from late-April through September, with peak spawning in July (Fisher et al. 2014; Killam et al. 2016). Fry emerge from July to mid-October, and recent evidence from otolith reconstruction suggests they rear in diverse tributary habitats along the Sacramento and lower Sacramento River and in freshwater reaches of the delta for several months prior to seaward migration (Phillis et al. 2018). Summertime spawning of this DPS is unique

among Chinook salmon, and is presumably an adaptation to hydrologic and thermal conditions in the spring-fed headwaters of the Sacramento River, which have been inaccessible for the past eight decades due to construction of impassable dams. As a result, these winter-run adults now depend on the cold tailwaters of an impassable dam.

Migrating smolts from this DPS experience higher survival during years with greater freshwater flows and ensuing favorable water quality conditions (Perry et al. 2016). In wetter years, a significant proportion of juveniles can also gain access to productive floodplain habitats, where growth rates are very high (Sommer et al. 2001).

Juveniles leave San Francisco Bay between January and April and enter the Gulf of the Farallones (Pyper et al. 2013). For the co-occurring Central Valley fall/late fall-run Chinook DPS, growth in the first ocean year is positively related to summer upwelling and negatively related to sea surface temperature, wind stress curl, and scalar winds (Wells et al. 2012). Faster freshwater growth rates have been shown to influence early marine survival when ocean conditions are poor (Woodson et al. 2013). Sacramento River winter-run Chinook has a more southerly ocean distribution than Central Valley fall-/late fall run Chinook salmon, and ocean distribution is concentrated off the California central coast (Satterthwaite et al. 2015; Johnson et al. 2016). Whether these distributions occur upon ocean entry as juveniles or later during the marine stage is unclear. Sacramento River winter-run Chinook individuals are smaller at a given age than Chinook from other Central Valley DPSs (Satterthwaite et al. 2012).

Climate Effects on Abundance and Distribution

Several factors contributed to the ranking of ***very high*** biological vulnerability to climate change for Sacramento River winter-run Chinook. The greatest risk to this DPS is its poor *population viability*, which was ranked ***very high***, driven primarily by its structure as a single population spawning outside of its historical range. The DPS is not thriving under present climate conditions, which are likely to worsen.

As exemplified by the series of drought conditions and extremely warm ocean temperatures during 2012-2016, this DPS is highly vulnerable to cumulative life-cycle impacts over multiple life stages, as well as in consecutive cohorts (Johnson et al. 2016); thus its sensitivity to *cumulative life-cycle effects* ranked ***very high***. During the 2012-2016 drought, limited availability of cold water resulted in egg exposure to lethal temperatures during summer and early fall (Johnson et al. 2017; Martin et al. 2017), leading to near extirpation of wild year classes. Exposure to *hydrologic regime* shift, *stream temperature*, and *summer water deficit* were ranked on the cusp between ***moderate*** and ***high***. These exposures are expected to worsen under future warming/drier climate scenarios. Adults (their gametes), eggs, and early juvenile life stages all rely on the availability of cold water from the Shasta Reservoir for survival during summer. The amount of cold water available to achieve tolerable, let alone optimal, temperatures for

these life stages is insufficient in some years and varies as a function of cumulative snowfall, rainfall, reservoir stratification, and previous water deliveries (NMFS 2009).

The extent to which Sacramento River winter-run Chinook experience reduced frequency of wetter annual conditions will likely influence recovery opportunities. While *flooding* may pose a risk of redd scour to salmonids in general, it is not thought to be a significant stressor for this DPS based on spawn timing and local habitat characteristics. Large storm events do not typically occur during summer months, inflow to the reservoir is lowest during summer, and outflow for agricultural deliveries are regulated (e.g., flood control releases are uncommon).

Higher flood flows are thought to provide beneficial conditions for juvenile salmon rearing and migration, with increased turbidity and cooler temperatures reducing predation risk (reviewed by Perry et al. 2016). Large floodplain habitats such as the Sutter and Yolo Bypasses are only fully inundated and available to juvenile salmon during flood conditions. Access to productive, shallow-water rearing habitats confers significant growth opportunity and likely survival benefits (Sommer et al. 2001; Woodson et al. 2013). Thus, to the extent that climate change reduces the frequency and duration of floodplain inundation, Sacramento River winter-run Chinook could be negatively impacted. This was reflected in a ***high*** exposure rank for *flooding*, a ***moderate*** exposure rank for *hydrologic regime* shift, and a ***moderate*** to ***high*** sensitivity rank for the *juvenile freshwater stage*.

Sea-level rise is predicted to be more pronounced in southern estuaries along the West Coast (Limburg et al. 2016). Recent projections for California estuaries suggest potential sea-level rise exceeding 10 feet by the end of the century if there is rapid loss of the Antarctic ice sheet (Griggs et al. 2017). Salmon transiting the San Francisco Bay estuary are thought to have higher mortality rates associated with tidal flows in the delta and estuary relative to unidirectional riverine flows (Perry et al. 2016). The extent to which sea-level rise reduces the amount of habitat exposed to unidirectional flow may influence juvenile salmon survival in the migration corridor. There is large uncertainty on how sea-level rise will influence the tidal prism and the suitability of habitats now used by Sacramento River winter-run Chinook. Sea level rise is also expected to reduce the availability of tidal marsh habitats used by juveniles because most estuarine shorelines are or will be armored, which will prevent migration to higher-elevation marshes (Stralberg et al. 2011). Overall, rankings were ***high*** for exposure to *sea level rise* and sensitivity in the *estuary stage*.

Sacramento River winter-run Chinook ranked ***moderate*** for sensitivity in the *marine stage*, and ***high*** for exposure to *upwelling*. This DPS exhibits a relatively unique ocean migratory behavior and a possibly a contracted distribution in the marine environment. Genetic and coded-wire-tag analyses indicate that these fish have a more southerly and nearshore marine distribution than other Chinook salmon ESUs and can be found schooling together (Satterthwaite et al. 2015; Johnson et al. 2016). They also enter

the ocean somewhat earlier and at a larger size than Central Valley fall-/late fall-run Chinook. To the extent that changes in climate conditions vary with latitude, this DPS may respond asynchronously with other Chinook ESUs, and may be more or less vulnerable to changes in ocean conditions.

Extrinsic Factors

Sacramento River winter-run Chinook is listed as endangered under the U.S. and California Endangered Species Acts. This DPS occupies the southern edge of the species range. Adults are blocked from historical spawning habitat, and spawning is now restricted to a single 50-km reach on the mainstem Sacramento River below Keswick Dam. This constricted spawning distribution makes Sacramento River winter-run Chinook particularly vulnerable to extinction (Lindley et al. 2007). Life-cycle modeling evaluations identify Sacramento River winter-run Chinook as highly vulnerable to extinction due to the frequency of drought conditions in its constrained spatial range (Lindley et al. 2007, Hendrix 2008). Multiple life stages are blocked from cooler and ecologically distinct headwaters due to Keswick and Shasta Dams. Therefore, efforts to reintroduce these fish to historical habitats in Battle Creek and the McCloud River above Shasta Dam are a high priority for recovery (NMFS 2009). Comparison of summer temperatures in the McCloud River in 2014 vs. the lethal temperatures in the mainstem Sacramento River highlight the loss of cooler summer temperatures in historical habitats that are now unavailable to this DPS. Sacramento River winter-run Chinook ranked ***very high*** in sensitivity to *population viability* and *other stressors* (including habitat loss).

For this DPS, Livingston Stone National Fish Hatchery plays an increasingly important role in reducing extinction risk when natural returns are low. Conservation hatcheries were scored as moderate in their effect on climate vulnerability in this assessment. During the 2012-2016 drought, egg-to-fry survival in the Sacramento River was exceptionally low for this DPS (<5% in 2014 and 2015: Johnson et al. 2017). A threefold increase in the production and release of hatchery juveniles will likely play an important role in preventing cohort failure of adult returns in 2017 and beyond. However, there is an important tradeoff for this conservation hatchery in responding to potential climate impacts on this DPS: to ensure adequate adult abundance while minimizing impacts to genetic integrity from an increasing proportion of hatchery fish (Johnson et al. 2016).

Adaptive Capacity

Sacramento River winter-run Chinook is sensitive to elevated temperatures at multiple life stages under present climate conditions; thus this DPS is likely at its physiological limit for adaptive capacity. At the southernmost region within the Chinook range, the California Central Valley offers the fewest opportunities for adaptive capacity among all Chinook salmon recovery domains. Thus, this DPS ranked ***low*** in overall adaptive capacity.

Due to historical population bottlenecks, Sacramento River winter-run Chinook lacks genetic diversity relative to other Chinook salmon DPSs (Banks et al. 2000; Lindley et al. 2007). This lack of genetic diversity may ultimately compromise the ability to adapt to future climate change.

Efforts to reintroduce Sacramento River winter-run Chinook to its historical habitats may reduce extinction risk by increasing spatial diversity and reducing reliance on inconsistent cold-water reserves from the Shasta Reservoir. Such reintroductions will provide the habitat and water quality templates upon which this DPS evolved. If access is reinstated to some of the habitats and conditions that historically sustained these fish during severe climate events (e.g., Ingram et al. 2013), locally adapted populations may emerge that are capable of coping with future hydrologic regimes.

Age diversity in adult and juvenile Sacramento River winter-run Chinook is low relative to that in other Chinook salmon DPSs (Satterthwaite et al. 2017). The vast majority (85-100%) of adults leave the ocean and return to spawn at age-3, suggesting very little carryover to older age classes (O'Farrell et al. 2012). All juveniles migrate at age-0; however, they move downstream from natal spawning reaches over a protracted period (Poytress et al. 2014). These juveniles rear in diverse habits as a function of hydrologic conditions prior to entering the ocean over a relatively narrow window of time (Pyper et al. 2013; Phillis et al. 2018). Reconstruction of adult otoliths indicate that juveniles rearing in the delta and in tributaries to the mainstem Sacramento River (Deer and Mill Creeks; American River) contribute significantly to adult returns (Phillis et al. 2018). This work also revealed that juveniles exhibit greater diversity in size, timing, and habitat use than previously thought (Johnson et al. 2017). This remnant phenotypic diversity and migration phenology may become increasingly important in ensuring some component of the DPS experiences favorable riverine, estuarine, and ocean conditions (Satterthwaite et al. 2014; Sturrock et al. 2015). However, impacts from the loss of significant juvenile rearing opportunity in headwater and intermittent streams, floodplains, and freshwater marshes cannot be understated. Such rearing opportunities likely played a significant role in supporting a thriving Sacramento River winter-run Chinook DPS that adapted to past climate extremes.

Literature Cited

Banks, M. A., V. K. Rashbrook, M. J. Calavetta, C. A. Dean, and D. Hedgecock. 2000. Analysis of microsatellite DNA resolves genetic structure and diversity of Chinook salmon (*Oncorhynchus tshawytscha*) in California's Central Valley. Can. J. Fish. Aquat. Sci. 57:915-927.

Fisher, J. P., L. A. Weitkamp, D. J. Teel, S. A. Hinton, J. A. Orsi, E. V. Farley, J. F. T. Morris, M. E. Thiess, R. M. Sweeting, and M. Trudel. 2014. Early ocean dispersal patterns of Columbia River Chinook and coho salmon. Trans. Am. Fish. Soc. 143:252-272.

Griggs, G., J. Arvai, D. Cayan, R. Deconto, J. Fox, H. A. Fricker, R. E. Hopp, C. Tebaldi, and E. A. Whiteman. 2017. Rising seas in California: an update on sea level rise science. California Ocean Science Trust, Oakland, CA.

Hendrix, N. 2008. A statistical model of Central Valley Chinook incorporating uncertainty. Description of *Oncorhynchus* Bayesian Analysis (OBAN) for winter run Chinook. R2 Resource Consultants, Inc, Redmond, WA

Ingram, B., F. Malamud-Roam, and S. Postel. 2013. The West without water: what past floods, droughts, and other climatic clues tell us about tomorrow. University of California Press, Oakland, CA.

Johnson, R., S. Windell, P. Brandes, J. Conrad, J. Ferguson, P. Goertler, B. Harvey, J. Heublein, J. Israel, D. Kratville , J. Kirsch, R. Perry, J. Pisciotto, W. Poytress, K. Reece, and B. Swart. 2017. Science advancements key to increasing management value of life stage monitoring networks for endangered Sacramento River winter run Chinook salmon in California. San Francisco Est. Water Sci. 15.

Johnson, R. C., J. C. Garza, R. B. MacFarlane, C. C. Phillis, P. L. Koch, P. K. Weber, and M. H. Carr. 2016. Isotopes and genes reveal freshwater origins of Chinook salmon (*Oncohynchus tshawytscha*) aggregations in California's coastal ocean. Mar. Ecol. Prog. Ser. 548:181-196.

Killam, D., M. A. Johnson, and R. Reynak. 2016. Chinook salmon populations of the Upper Sacramento River Basin in 2015. California Dept.Fish and Game RBFO Tech. Rep. 03 2016.

Limburg, K., R. Brown, R. Johnson, B. Pine, R. Rulifson, D. Secor, K. Timchak, B. Walther, and K. Wilson. 2016. Round-the-coast: Snapshots of estuarine climate change effects. Fisheries 41:392-394.

Lindley, S., R. Schick, E. Mora, P. Adams, J. Anderson, S. Greene, C. Hanson, B. May, D. McEwan, R. MacFarlane, C. Swanson, and J. Williams. 2007. Framework for assessing viability of threatened and endangered Chinook salmon and steelhead in the Sacramento San Joaquin Basin. San Francisco Est. Water Sci. 5.

Martin, B., A. Pike, S. John, N. Hamad, J. Roberts, S. Lindley, and E. Danner. 2017. Phenomenological vs. biophysical models of thermal stress in aquatic eggs. Ecol. Letters 20.

NMFS (National Marine Fisheries Service). 2009. Biological Opinion on the Operating Criteria and Plan (OCAP) for the long-term operations of the Central Valley Project and State Water Project with recommendations for Essential Fish Habitat. Page 844. U.S. Dep. Commerce NMFS Southwest Region, Long Beach, CA.

O'Farrell, M., M. Mohr, A. Grover, and W. Satterthwaite. 2012. Sacramento River winter Chinook salmon cohort reconstruction: analysis of ocean fishery impacts. U.S. Dep. Commerce NOAA Tech. Memo. NMFS SWFSC 491. Santa Cruz, CA.

Perry, R., R. Buchanan, P. Brandes, J. Burau , and J. Israel. 2016. Anadromous salmonids in the Delta: New science 2006 2016. San Francisco Est. Water Sci. 14:1-28.

Phillis, C., A. Sturrock, R. Johnson, and P. Weber. 2018. Endangered winter-run Chinook salmon rely on diverse rearing habitats in a highly altered landscape. Biol. Conserv. 217:358-362.

Poytress, W., J. Gruber, F. Carrillo, and S. Voss. 2014. Compendium report of Red Bluff Diversion Dam rotary trap juvenile anadromous fish production indices for years 2002-2012. U.S. Fish and Wildlife Service, Red Bluff, CA.

Pyper, B., T. Garrison, S. Cramer, P. Brandes, D. Jacobson, and M. Banks. 2013. Absolute abundance estimates of juvenile spring run and winter run Chinook salmon at Chipps Island. Science of the Delta Stewardship Council Grant Agreement Number 1049.

Satterthwaite, W. H., S. M. Carlson, S. D. Allen-Moran, S. Vincenzi, S. J. Bograd, and B. K. Wells. 2014. Match-mismatch dynamics and the relationship between ocean-entry timing and relative ocean recoveries of Central Valley fall run Chinook salmon. Mar. Ecol. Prog. Ser. 511:237-248.

Satterthwaite, W. H., S. M. Carlson, and A. Criss. 2017. Ocean size and corresponding life history diversity among the four run timings of California Central Valley Chinook salmon. Trans. Am. Fish. Soc. 146:594-610.

Satterthwaite, W. H., J. Ciancio, E. Crandall, M. L. Palmer-Zwahlen, A. M. Grover, M. R. O’Farrell, E. C. Anderson, M. S. Mohr, and J. C. Garza. 2015. Stock composition and ocean spatial distribution inference from California recreational Chinook salmon fisheries using genetic stock identification. Fish. Res. 170:166-178.

Satterthwaite, W. H., M. S. Mohr, M. R. O'Farrell, and B. K. Wells. 2012. A Bayesian hierarchical model of size-at-age in ocean-harvested stocks; quantifying effects of climate and temporal variability. Can. J. Fish. Aquat. Sci. 69:942-954.

Sommer, T., M. Nobriga, W. Harell, W. Batham, and W. Kimmerer. 2001. Floodplain rearing of juvenile Chinook salmon: evidence of enhanced growth and survival. Can. J. Fish. Aquat. Sci. 58:325-333.

Stralberg, D., M. Brennan, J. C. Callaway, J. K. Wood, L. M. Schile, D. Jongsomjit, M. Kelly, V. T. Parker, and S. Crooks. 2011. Evaluating tidal marsh sustainability in the face of sea-level rise: a hybrid modeling approach applied to San Francisco Bay. PLoS ONE 6:e27388.

Sturrock, A. M., J. D. Wikert, T. Heyne, C. Mesick, A. E. Hubbard, T. M. Hinkelman, P. K. Weber, G. E. Whitman, J. J. Glessner, and R. C. Johnson. 2015. Reconstructing the migratory behavior and long-term survivorship of juvenile Chinook salmon under contrasting hydrologic regimes. PLoS ONE 10:e0122380.

Wells, B. K., J. A. Santora, J. C. Field, R. B. MacFarlane, B. B. Marinovic, and W. J. Sydeman. 2012. Population dynamics of Chinook salmon *Oncorhynchus tshawytscha* relative to prey availability in the central California coastal region. Mar. Ecol. Prog. Ser. 457:125-137.

Woodson, L. E., B. K. Wells, P. K. Weber, R. B. MacFarlane, G. E. Whitman, and R. C. Johnson. 2013. Size, growth, and origin-dependent mortality of juvenile Chinook salmon *Oncorhynchus tshawytscha* during early ocean residence. Mar. Ecol. Prog. Ser. 487:163-175.

Central Valley spring-run Chinook

Overall vulnerability—Very high (100% High)
Biological sensitivity—Very high (1% High, 99% Very high)
Climate exposure—High (78% High, 22% Very high)
Adaptive capacity—Low (1.56)
Data quality—53% of scores ≥ 2

	Central Valley spring-run Chinook	Expert Scores	Data Quality	Expert Scores Plots (Portion by Category)
Sensitivity attributes	Early life history	2	1.5	
	Juvenile freshwater stage	2.8	1.8	
	Estuary stage	3.4	2	
	Marine stage	2.6	1.8	
	Adult freshwater stage	3.8	3	
	Cumulative life-cycle effects	3.8	2.8	
	Hatchery influence	3.7	3	
	Other stressors	3.6	2.3	
	Population viability	3.5	2.8	
	Ocean acidification sensitivity	1.8	1	
	Sensitivity Score	Very High		
Exposure variables	Stream temperature	3	2	
	Summer water deficit	3	1.8	
	Flooding	3.3	1.3	
	Hydrologic regime	2.9	1.8	
	Sea level rise	3.5	2.3	
	Sea surface temperature	3.4	2	
	Ocean acidification exposure	4	2.5	
	Upwelling	3.3	1	
	Ocean currents	1.9	1	
	Exposure Score	High		
Overall Vulnerability Rank		Very High		

Low
Moderate
High
Very High

Life History Synopsis

Central Valley spring-run Chinook adults migrate past the Golden Gate Bridge and up the Sacramento River from late January to early February. After reaching spawning areas from March through June, they hold in cold, deep pools to mature during summer (CDFG 2003-2012). Spawning occurs in fall, and eggs incubate from about September to March. Juveniles migrate downstream throughout spring of the same year they hatched, although a small portion remains through summer and enters the ocean the following spring.

Historically, spring-run Chinook inhabited most river systems of the Sierra Nevada, but Chinook stocks have been extirpated from many of these watersheds.

Remaining independent populations occur in relatively small tributaries of the Sacramento River (Mill, Deer, and Butte Creek), each of which is about 500 km from the ocean. A hatchery stock occupies the Feather River; however, this stock is assumed to be a major threat to the genetic integrity of wild spring-run Chinook due to interbreeding and hybridization in the hatchery between spring-run and fall-run fish (Johnson and Lindley 2016).

Some age-0 juveniles rear in natal creeks, while others are thought to rear downstream in the Sutter and Yolo Bypass flood-control channels, the Sacramento River, or the Sacramento-San Joaquin Delta. The Sacramento-San Joaquin Delta is a geographically extensive, highly engineered, and decidedly altered estuary draining nearly the entire Central Valley watershed. It is not known but commonly assumed that some or even many age-0 spring-run juveniles rear in the delta. Therefore, even though estuary rearing time is unknown, some juveniles likely forage for one month or more in the estuary while others simply migrate through (CDFG 1998; Brandes and McLain 2001; Whipple et al. 2012). The number of age-0 juveniles that rear in the delta is poorly estimated because estimates rely solely on length-at-date criteria from recaptures at a rotary-screw-trap (the only means of detection), and this method is inaccurate (Brandes and McLain 2001; Harvey et al. 2014).

Central Valley spring-run Chinook has a relatively broad ocean distribution from central California to Cape Falcon, Oregon, similar to that of the fall run (Satterthwaite et al. 2015). Adults return to fresh water at age 2, 3, or 4, with the majority returning at age 3 or 4. While in the ocean, these fish are subject to commercial and recreational fisheries at ages 3 and 4 (Grover et al. 2004).

In part because of its low diversity, poor spatial structure, and low abundance (Lindley et al. 2007; Williams et al. 2011), Central Valley spring-run Chinook is listed as threatened under the Endangered Species Act.

Climate Effects on Abundance and Distribution

Central Valley spring-run Chinook ranked ***very high*** in overall, vulnerability to climate change, consistent with the findings of Moyle et al. (2017). This DPS ranked ***moderate*** and ***high***, respectively, in exposure to *stream temperature* and *summer water deficit*. Due to present climate stress in the freshwater habitats used for pre-spawn holding, Central Valley spring-run Chinook also ranked ***very high*** in sensitivity at the *adult freshwater stage*. In Butte Creek, which hosts the largest single run from this DPS, high mortality of holding adults was associated with elevated water temperatures and pathogen outbreaks in 2002 and 2003. All accessible spawning areas of Butte Creek are below 1,000 ft in elevation.

Owing to the extremely vulnerable state of the Sacramento-San Joaquin Delta, Central Valley spring-run Chinook ranked ***moderate*** for exposure to *hydrologic regime*

shift and *stream temperature*, but ***high*** for exposure to *sea level rise*. Because this DPS is moderate-to-highly vulnerable to climate risks throughout its entire life cycle, it ranked ***very high*** in sensitivity to *cumulative life-cycle effects* (see also Herbold et al. 2018). Fish in this DPS migrate and rear in the Sacramento-San Joaquin Delta, where mortality rates are high (Moyle et al. 2017); thus it ranked ***high*** in sensitivity at the *estuary stage*.

Although Central Valley spring-run Chinook scored relatively ***low*** in sensitivity for *early life history* (egg incubation/early fry stage), there is evidence that temperatures during incubation (September-March) sometimes exceed the critical limits for 100% mortality (CDFG 1998). Rising temperatures could also affect juvenile growth and development rates, especially for yearlings that may hold over summer in freshwater tributaries.

Climate predictors associated with marine survival or behavior are not known for Central Valley spring-run Chinook specifically, but for Pacific salmon in general, growth and survival have been correlated with large-scale indicators, such as the El Niño Southern and Pacific Decadal Oscillations. Moreover, a strong correlation has been shown between early marine survival and upwelling/sea surface temperature. Early marine links between climate indicators and survival are better understood than later marine links (Wells et al. 2007; Petrosky and Schaller 2010). Overall, Central Valley spring-run Chinook ranked ***moderate*** in sensitivity at the *marine stage*, but ***high*** in exposure to *sea surface temperature* and *upwelling*.

Extrinsic Factors

The California Central Valley and Sacramento-San Joaquin Delta are highly altered systems, with impassable dams on nearly every major river and a very high concentration of invasive predators. These anthropogenic effects prevent the species from using high-elevation streams in the Sierra Nevada, where fish would have more opportunities for refuge from predicted effects of climate change such as higher stream temperatures, and lower snowpack. The few remaining populations of Central Valley spring-run Chinook salmon are quite vulnerable to all three extrinsic factors. These spring-run adults need to hold over summer in headwaters of the natal river, yet all populations are relegated to relatively low-elevation streams. For these reasons, Central Valley spring-run Chinook ranked ***very high*** in sensitivity to *other stressors*. This DPS also ranked ***very high*** in sensitivity to *hatchery influence* due to its reliance on the Feather River Hatchery program.

Central Valley spring-run Chinook is listed as threatened under the U.S. Endangered Species Act. Spatial structure in this DPS was considered especially vulnerable to climate change, and it ranked ***very high*** in sensitivity for *population viability*.

Adaptive Capacity

Central Valley spring-run Chinook ranked ***low*** in adaptive capacity. This was in large part due to highly altered freshwater and estuary ecosystems, which have reduced the number of viable life histories and created a situation where the species is highly dependent on human management of resources (such as design flows to queue migrating adults and keep temperatures cool).

Literature Cited

Brandes, P. L. and J. S. McLain. 2001. Juvenile Chinook salmon abundance, distribution and survival in the Sacramento San Joaquin estuary. Calif. Dep.Fish Game Fish Bull.179:39-138.

CDFG (California Department of Fish and Game). 1998. A status review of the spring run Chinook salmon (*Oncorhyncus tshawytscha*) in the Sacramento River drainage. Candidate Species Status Report 98-01, Sacramento, CA.

CDFG (California Department of Fish and Game). 2003-2012. Butte creek spring run Chinook salmon, *Oncorhynchus tshawytscha* pre spawn mortality evaluation. Inland fisheries administrative reports (10 reports), Sacramento, CA.

Grover, A., A. Lowe, P. Ward, J. Smith, M. Mohr, D. Vierle, and C. Tracy. 2004. Recommendations for developing fishery management plan conservation objectives for Sacramento River winter Chinook and Sacramento River spring Chinook. Report to the Pacific Fishery Management Council, Portland, OR.

Harvey, B. N., D. P. Jacobson, and M. A. Banks. 2014. Quantifying the uncertainty of a juvenile Chinook salmon race identification method for a mixed-race stock. N. Am. J. Fish. Manage. 34:1177-1186.

Herbold, B., S. M. Carlson, and R. Henery. 2018. Managing for Salmon Resilience in California’s Variable and Changing Climate. San Francisco Est. Water Sci. 16.

Johnson, R. C. and S. T. Lindley. 2016. Isotopes and genes reveal freshwater origins of Chinook salmon (*Oncohynchus tshawytscha*) aggregations in California’s coastal ocean. U.S. Dep. Commerce NOAA Tech. Memo. NMFS-SWFSC-564.

Lindley, S., R. Schick, E. Mora, P. Adams, J. Anderson, S. Greene, C. Hanson, B. May, D. McEwan, R. MacFarlane, C. Swanson, and J. Williams. 2007. Framework for assessing viability of threatened and endangered Chinook salmon and steelhead in the Sacramento San Joaquin Basin. San Francisco Est. Water Sci. 5.

Moyle, P. B., R. Lusardi, P. Samuel, and J. Katz. 2017. State of the salmonids: Status of California’s emblematic fishes 2017. Center for Watershed Sciences, University of California, Davis and California Trout, San Francisco, CA. Available at http://caltrout.org/sos/.

Petrosky, C. E. and H. A. Schaller. 2010. Influence of river conditions during seaward migration and ocean conditions on survival rates of Snake River Chinook salmon and steelhead. Ecol. Freshw. Fish. 19:520-536.

Satterthwaite, W. H., J. Ciancio, E. Crandall, M. L. Palmer-Zwahlen, A. M. Grover, M. R. O'Farrell, E. C. Anderson, M. S. Mohr, and J. C. Garza. 2015. Stock composition and ocean spatial distribution inference from California recreational Chinook salmon fisheries using genetic stock identification. Fish. Res. 170:166-178.

Wells, B. K., C. B. Grimes, and J. B. Waldvogel. 2007. Quantifying the effects of wind, upwelling, curl, sea surface temperature and sea level height on growth and maturation of a California Chinook salmon (*Oncorhynchus tshawytscha*) population. Fish. Oceanogr. 16:363-382.

Whipple, A., R. M. Grossinger, D. Rankini, B. Stanford, and R. A. Askevold. 2012. Sacramento San Joaquin Delta historical ecology investigation: Exploring pattern and process. Report of the San Francisco Estuary Institute, Richmond, CA.

Williams, T. H., S. T. Lindley, B. C. Spence, and D. A. Boughton. 2011. Status review update for Pacific salmon and steelhead listed under the endangered species act: Southwest. Updated report 20 May 2011, U.S. Dep. Commerce, NOAA Fisheries, Santa Cruz, CA.

Central Valley fall/late fall-run Chinook

Overall vulnerability—Very high (21% High, 79% Very high)
Biological sensitivity—Very high (24% High, 76% Very high)
Climate exposure—High (87% High, 13% Very high)
Adaptive capacity—Low (1.5)
Data quality—68% of scores ≥ 2

	Central Valley fall/late fall-run Chinook	Expert Scores	Data Quality	Expert Scores Plots (Portion by Category)
Sensitivity attributes	Early life history	2.3	2.5	
	Juvenile freshwater stage	2.9	2.8	
	Estuary stage	3.4	2.3	
	Marine stage	3.3	2.5	
	Adult freshwater stage	2.8	2.8	
	Cumulative life-cycle effects	3.6	2	
	Hatchery influence	3.9	3	
	Other stressors	3.6	2.5	
	Population viability	2.6	3	
	Ocean acidification sensitivity	1.8	1.5	
	Sensitivity Score	Very High		
Exposure variables	Stream temperature	2.8	2	
	Summer water deficit	3.1	1.8	
	Flooding	3.2	1.3	
	Hydrologic regime	2.7	1.8	
	Sea level rise	3.4	2.3	
	Sea surface temperature	3.4	2	
	Ocean acidification exposure	4	2.5	
	Upwelling	3.3	1	
	Ocean currents	1.9	1	
	Exposure Score	High		
Overall Vulnerability Rank		Very High		

Low
Moderate
High
Very High

Life History Synopsis

Adults of the Central Valley fall/late fall-run Chinook DPS enter rivers fully mature and move quickly to spawning grounds (Williams 2006; Moyle et al. 2017). Peak spawning typically occurs during October-November but can continue into early January. Naturally spawned juveniles emerge from the gravel from December through March and rear in fresh water for 1-7 months, moving downstream into large rivers after only a few weeks, with most movement occurring at night.

This Chinook DPS has a life history that minimizes time spent in fresh water. Juveniles migrate to the ocean in spring before water temperatures become too warm. Thus these fish can exploit the lower-elevation reaches of Central Valley rivers prior to

being exposed to summer temperatures that exceed thermal tolerances for salmon. These characteristics also make them the preferred broodstock for use in hatcheries.

Turbidity has a strong positive relationship with survival during juvenile migration, in part through its association with high flows and cooler temperatures (CDWR 2016; Johnson et al. 2017). Historically, these juveniles likely foraged for 1-2 months on floodplains, where growth is demonstrably much faster than in rivers (Sommer et al. 2001). Today, most river-reared fish enter the estuary at a relatively small size and suffer high mortality as a consequence. Hatchery fish are typically released to rivers at a larger size than their naturally spawned cohorts (Huber and Carlson 2015), but naive fish released into clear, shallow water are subjected to heavy predation.

From the estuary, Central Valley fall/late fall-run Chinook juveniles move into the Gulf of the Farallones, a food-rich region in most years due to wind-driven upwelling associated with the California Current (Wells et al. 2012). Fish from this DPS spend 2-5 years at sea, where they feed on fish and shrimp. For juveniles, the early ocean distribution is related to shallower waters with high chlorophyll concentrations and proximity to the natal river (Hassrick et al. 2016). As adults, most of these fish remain off the California coast between Point Sur and Point Arena, but some move into coastal waters off Oregon (Palmer-Zwahlen and Jormos 2015; Satterthwaite et al. 2015).

Climate Effects on Abundance and Distribution

Central Valley fall-/late fall-run Chinook is sensitive to changing conditions at all life stages but is particularly sensitive to conditions in the ocean and in estuaries, where individuals spend most of their lives (Moyle et al. 2017; Herbold et al. 2018). This DPS ranked ***moderate*** in sensitivity at the *juvenile* and *adult freshwater stages* and ***high*** at the *estuary* and *marine stages*. Due to the dominance of hatchery fish, variation in estuary arrival timing is greatly reduced, making the DPS as a whole much more vulnerable to cohort collapse (Lindley et al. 2009). More generally, the highly altered freshwater habitat and severe reduction life history viability of this DPS (Sturrock et al. 2015) contributed to its rank of ***very high*** in *cumulative life-cycle effects*.

Central Valley fall/late fall-run Chinook ranked high in overall exposure attributes, with nearly every score in the high category. This DPS was ranked ***high*** in *flooding* due to expected dramatic increases in atmospheric river intensity and frequency. Adults migrate in fall, often during the first large seasonal storms, and thus are affected by storm timing. Exposure to *hydrological regime* shift was ranked ***moderate*** because of the role played by snowpack in water management over the larger region. Although the actual spawning area of this DPS is already rain-dominated, snowfall in the Sierra Nevada Mountains still plays an important role. The Sacramento River is heavily managed, and the potential for reservoir management to limit future impacts from flooding and loss of snowfall was uncertain.

As noted above, juveniles of this DPS emerge in winter and move quickly downstream to the estuary in late winter through spring. However, they are vulnerable to stranding during low flows or as a result of regional water management practices. Thus, this DPS ranked ***high*** in exposure to *sea surface temperature*, *upwelling*, and *sea level rise*.

Extrinsic Factors

The California Central Valley and San Francisco Bay-Delta are highly altered systems, with impassable dams on nearly every major river and with high concentrations of predators. Salmon runs are largely maintained by hatchery fish that exhibit high stray rates, so the overall influence of domestication selection is high, with genetic homogenization occurring among populations (Williamson and May 2005; Johnson et al. 2012). For fall/late fall-run juvenile salmon, survival rates are variable among years but generally low (Perry et al. 2010; Michel et al. 2015). Thus sensitivity to *other stressors* for this DPS was ranked ***very high***. Moyle et al. (2017) rated wild components of both the late fall and fall run as "of high concern" and on a pathway to extinction.

This DPS is regarded as a species of concern by the National Marine Fisheries Service, and was ranked ***moderate*** for *population viability*. Nonetheless, concern is very high for the late-fall life history and wild components of this DPS (Moyle et al. 2017). The DPS is now dominated by hatchery fish and comprised of a single, genetically uniform population under artificial selection (Williams 2006; Lindley et al. 2009), driving its *hatchery influence* score to 3.9, the highest of all DPSs for this attribute.

Adaptive Capacity

Central Valley fall/late fall-run Chinook ranked ***low*** in adaptive capacity, largely due to highly altered freshwater and estuary ecosystems, which have reduced the number of viable life histories. As a result of these alterations, the DPS is highly dependent on human management of resources, such as released flows from reservoirs to cue migrating adults and keep temperatures cool.

Literature Cited

Life history information in this account is derived from Moyle (2002), Williams (2006), and Moyle et al. (2017), which should be consulted for additional references.

CDWR (California Department of Water Resources). 2016. Final report: 2014 Georgiana Slough floating fish guidance structure performance evaluation project report. Report of the CDWR, Bay-Delta Office, Sacramento, CA.

Hassrick, J. L., M. J. Henderson, D. D. Huff, W. J. Sydeman, M. C. Sabal, J. A. Harding, A. J. Ammann, E. D. Crandall, E. P. Bjorkstedt, J. C. Garza, and S. A. Hayes. 2016. Early ocean distribution of juvenile Chinook salmon in an upwelling ecosystem. Fish Oceanogr **25**:133-146.

Herbold, B., S. M. Carlson, and R. Henery. 2018. Managing for Salmon Resilience in California's Variable and Changing Climate. San Francisco Est. Water Sci. **16**.

Huber, E. R., and S. M. Carlson. 2015. Temporal trends in hatchery releases of fall-run Chinook salmon in California's Central Valley. San Francisco Est. Water Sci. **13**:23pp.

Johnson, R., S. Windell, P. Brandes, J. Conrad, J. Ferguson, P. Goertler, B. Harvey, J. Heublein, J. Israel, D. Kratville , J. Kirsch, R. Perry, J. Pisciotto, W. Poytress, K. Reece, and B. Swart. 2017. Science advancements key to increasing management value of life stage monitoring networks for endangered Sacramento River Winter run Chinook salmon in California. San Francisco Est. Water Sci. **15**.

Johnson, R. C., P. K. Weber, J. D. Wikert, M. L. Workman, R. B. MacFarlane, M. J. Grove, and A. K. Schmitt. 2012. Managed metapopulations: do salmon hatchery 'sources' lead to in-river 'sinks' in conservation? PLoS ONE **7**:e28880.

Lindley, S. T., C. B. Grimes, M. S. Mohr, W. Peterson, J. Stein, J. T. Anderson, L. W. Botsford, D. L. Bottom, C. A. Busack, T. K. Collier, J. Ferguson, J. C. Garza, A. M. Grover, D. G. Hankin, R. G. Kope, P. W. Lawson, A. Low, R. B. MacFarlane, K. Moore, M. Palmer-Zwahlen, F. B. Schwing, J. Smith, C. Tracy, R. Webb, B. K. Wells, and T. H. Williams. 2009. What caused the Sacramento River fall Chinook stock collapse? NOAA-TM-NMFS-SWFSC-447, U.S. Bur Commerce NOAA-TM-NMFS-SWFSC-447, Santa Cruz, CA.

Michel, C. J., A. J. Ammann, S. T. Lindley, P. T. Sandstrom, E. D. Chapman, M. J. Thomas, G. P. Singer, A. P. Klimley, R. B. MacFarlane, and M. Bradford. 2015. Chinook salmon outmigration survival in wet and dry years in California's Sacramento River. Can. J. Fish. Aquat. Sci. **72**:1749-1759.

Moyle, P. B. 2002. Inland fishes of California. University of California Press, Berkeley, CA.

Moyle, P. B., R. Lusardi, P. Samuel, and J. Katz. 2017. State of the salmonids: Status of California's emblematic fishes 2017. Center for Watershed Sciences, University of California, Davis and California Trout, San Francisco, CA. Available at http://caltrout.org/sos/.

Palmer-Zwahlen, M. and B. Jormos. 2015. Recovery of Coded-Wire Tags from Chinook Salmon in California's Central Valley Escapement, Inland Harvest, and Ocean Harvest in 2012. California Department of Fish and Wildlife, Santa Rosa.

Perry, R. W., J. R. Skalski, P. L. Brandes, P. T. Sandstrom, A. P. Klimley, A. Ammann, and B. MacFarlane. 2010. Estimating survival and migration route probabilities of juvenile Chinook salmon in the Sacramento–San Joaquin River Delta. N. Am. J. Fish. Manage. **30**:142-156.

Satterthwaite, W. H., J. Ciancio, E. Crandall, M. L. Palmer-Zwahlen, A. M. Grover, M. R. O'Farrell, E. C. Anderson, M. S. Mohr, and J. C. Garza. 2015. Stock composition and ocean spatial distribution inference from California recreational Chinook salmon fisheries using genetic stock identification. Fish Res **170**:166-178.

Sommer, T., M. Nobriga, W. Harell, W. Batham, and W. Kimmerer. 2001. Floodplain rearing of juvenile Chinook salmon: evidence of enhanced growth and survival. Can. J. Fish. Aquat. Sci. 58:325-333.

Sturrock, A. M., J. D. Wikert, T. Heyne, C. Mesick, A. E. Hubbard, T. M. Hinkelman, P. K. Weber, G. E. Whitman, J. J. Glessner, and R. C. Johnson. 2015. Reconstructing the migratory behavior and long-term survivorship of juvenile Chinook salmon under contrasting hydrologic regimes. PLoS ONE **10**:e0122380.

Wells, B. K., J. A. Santora, J. C. Field, R. B. MacFarlane, B. B. Marinovic, and W. J. Sydeman. 2012. Population dynamics of Chinook salmon *Oncorhynchus tshawytscha* relative to prey availability in the central California coastal region. Mar. Ecol. Prog. Ser. **457**:125-137.

Williams, J. G. 2006. Central Valley salmon: A perspective on Chinook and steelhead in the Central Valley of California. San Francisco Est. Water Sci. **4**.

Williamson, K. S., and B. May. 2005. Homogenization of fall-run Chinook salmon gene pools in the Central Valley of California, USA. N. Am. J. Fish. Manage. **25**:993-1009.

California Coastal Chinook

Overall vulnerability—High (2% Moderate, 86% High, 12% Very high)
Biological Sensitivity—High (3% Moderate, 97% High)
Climate exposure—High (88% High, 12% Very high)
Adaptive capacity—Low (1.56)
Data quality—42% of scores ≥ 2

	California Coastal Chinook	Expert Scores	Data Quality	Expert Scores Plots (Portion by Category)
Sensitivity attributes	Early life history	1.7	1.5	
	Juvenile freshwater stage	2.3	1.8	
	Estuary stage	2.8	1.8	
	Marine stage	2.6	1.8	
	Adult freshwater stage	2.5	2.8	
	Cumulative life-cycle effects	3.3	1.8	
	Hatchery influence	1.1	3	
	Other stressors	3.6	1.8	
	Population viability	3	3	
	Ocean acidification sensitivity	1.8	1.5	
	Sensitivity Score	High		
Exposure variables	Stream temperature	3.3	2	
	Summer water deficit	2.4	1.8	
	Flooding	3.5	1.3	
	Hydrologic regime	1	2.5	
	Sea level rise	3.3	2	
	Sea surface temperature	3.3	2	
	Ocean acidification exposure	4	2.5	
	Upwelling	3.3	1	
	Ocean currents	1.9	1	
	Exposure Score	High		
Overall Vulnerability Rank		High		

Legend: Low, Moderate, High, Very High

Life History Synopsis

In large rivers that remain open to the ocean all summer, fall-run adults of the California Coastal Chinook DPS migrate from September through December or January. In streams with bar-built estuaries, adult freshwater entry may be delayed until the onset of fall rains (Myers et al. 1998; S. Harris, CDFW, personal communication). Spawning generally occurs from October to December (Fukushima and Lesh 1998; Myers et al. 1998; M. Sparkman, CDFW, personal communication), and egg incubation from November into April.

Fall-run Chinook salmon produce primarily ocean-type juveniles that reside for less than one year in fresh water. These ocean-type juveniles migrate to estuaries or the

ocean between March and August of their first year (Gallagher 2001, 2003, 2004; Chase et al. 2002, 2004, 2005). Early migrants are often young fry smaller than 50 mm, whereas June and July migrants tend to be larger (~70 mm). A small fraction of juveniles reside for a full year in fresh water and migrate the following spring (Sparkman et al. 2016), probably due to variation in individual growth rates rather than to a genetically distinct life-history type.

Juvenile use of estuaries likely varies among populations of this DPS. Juvenile Chinook have been reported in Humboldt Bay throughout summer, suggesting several months of estuarine residence (Wallace and Allen 2009). Many stream systems have bar-built estuaries, with sand bars that recurrently close and open the marine connection, creating seasonal freshwater lagoons. The majority of juveniles probably enter the ocean before bar formation occurs, typically by late July, but some remain in the lagoon until connectivity to the ocean is reestablished upon return of the wet season in October or November (Madej et al. 2012).

In the ocean, California coastal Chinook are distributed primarily between Pt. Reyes and southern Oregon, with highest abundances in the Fort Bragg and Klamath subareas (Satterthwaite et al. 2014b, 2015; Bellinger et al. 2015). Most fish appear to mature at age 3 or 4, with age-2 jacks comprising 5-11 % of returns (Myers et al. 1998). Interannual variation in the percentage of each life-history type can be substantial.

Climate Effects on Abundance and Distribution

In fresh water, the interaction between changes in precipitation and warming will likely have effects that propagate through the life cycle of California coastal Chinook. Sensitivity to *cumulative life-cycle effects* was ranked ***high*** for this DPS. Changes in the timing and magnitude of fall storms could adversely affect the ability of migrating adults to enter streams and navigate to spawning areas. These conditions merited ranks of ***high*** exposure to *flooding* and ***moderate*** sensitivity at the *adult freshwater stage*.

For the *early life history* (egg stage), likely climatic threats include redd scour during high flows (exacerbated by habitat degradation) and deposition of fine sediments in spawning gravels, both of which will be affected by rainfall intensity and frequency (NMFS 2015). Additionally, incubation rates are dependent on water temperature (Murray and McPhail 1988); thus, accelerated incubation and early emergence will occur in a warmer climate. *Stream temperature* and *hydrologic regime* shift could be limiting for the fraction of juveniles that migrate downstream in summer or spend a full year in fresh water. This DPS ranked ***high*** in exposure to *stream temperature*. Climate change could also affect estuarine habitat quality, especially in the summer dry season; thus, California coastal Chinook ranked ***moderate*** in sensitivity at the *estuarine stage*.

The first spring and summer at sea is considered a critical period when most ocean mortality occurs (Beamish and Mahnken 2001; Wells et al. 2012), although factors

affecting ocean survival are complex and multi-scale. The state of the North Pacific High (NPH) during winter influences subsequent productivity (Schroeder et al. 2009, 2013; Black et al. 2011; Thompson et al. 2012), with a strong NPH maintaining both nutrient levels and an abundant and diverse diet portfolio (Thayer et al. 2014). Timing of the spring transition, and the intensity and consistency of upwelling (also affected by the NPH) control the development, maintenance, and retention of krill populations and forage fish, as well as affecting potential predators of salmon (Fiechter et al. 2015).

Because of the complexity of interactions operating at different scales, attempts to directly correlate survival to individual environmental variables have not been especially successful. However, the timing, intensity, and duration of upwelling are clearly key factors likely to be affected by changing climate (Satterthwaite et al. 2014a). California coastal Chinook ranked ***high*** in exposure to both mean sea *surface temperature* and *upwelling* and ***moderate*** in sensitivity at the *marine stage*.

Extrinsic Factors

Historically, the California Coastal Chinook DPS included both spring- and fall-run stocks, but the spring-run has been extirpated. This DPS is listed as threatened under the U.S. Endangered Species Act (Spence et al. 2008; Spence 2016). Sensitivity to *population viability* was ranked ***moderate*** to ***high*** for California Coastal Chinook with sensitivity to *other stressors* ranked ***high***. Threats include incidental harvest and dams that block access to habitat on the Eel and Russian Rivers. Incidental harvest is regulated, but its impacts remain highly uncertain (O'Farrell et al. 2012, 2015), and indirect mortality from catch-and-release of undersized fish, as well as bycatch in non-salmon fisheries, have been identified as concerns (NMFS 2015).

In fresh water, warm-adapted invasive species are likely to gain advantage with climate change and prey on juvenile Chinook salmon or limit their use of preferred habitat. Such species include non-native smallmouth bass, striped bass, channel catfish (NMFS 2015), and Sacramento pikeminnow. Migrating adult Chinook salmon are commonly intercepted by recreational fishing in the Eel and Russian Rivers (NMFS 2015). Although caught fish must be released, capture and handling occurs during periods of thermal stress and has an impact that is likely to increase with climate change.

Adaptive Capacity

Overall, California Coastal Chinook ranked ***low*** in adaptive capacity. As conditions in summer holding pools become less favorable, increased summer warming will likely reduce opportunities for re-expression of the presently extirpated spring-run life history.

Literature Cited

Beamish, R. J. and C. Mahnken. 2001. A critical size and period hypothesis to explain natural regulation of salmon abundance and the linkage to climate and climate change. Oceanography 49:423-435.

Bellinger, M. R., M. A. Banks, S. J. Bates, E. D. Crandall, J. C. Garza, G. Sylvia, and P. W. Lawson. 2015. Geo-referenced, abundance calibrated ocean distribution of Chinook salmon (*Oncorhynchus tshawytscha*) stocks across the West Coast of North America. PLoS ONE 10:e0131276.

Black, B. A., I. D. Schroeder, W. J. Sydemans, S. J. Bograd, B. K. Wells, and F. B. Schwing. 2011. Winter and summer upwelling modes and their biological importance in the California Current Ecosystem. Global Change Biol. 17:2536-2545.

Chase, S., R. Benkert, D. Manning, and S. White. 2002. Sonoma County Water Agency’s Mirabel Rubber Dam/Wohler Pool fish sampling program: year 2 results, 2001. Sonoma County Water Agency, Santa Rosa, CA

Chase, S., R. Benkert, D. Manning, and S. White. 2004. Sonoma County Water Agency’s Mirabel Rubber Dam/Wohler Pool fish sampling program: year 4 results, 2003. Sonoma County Water Agency, Santa Rosa, CA

Chase, S., R. Benkert, D. Manning, and S. White. 2005. Sonoma County Water Agency’s Mirabel Rubber Dam/Wohler Pool fish sampling program: year 5 results, 2004. Sonoma County Water Agency, Santa Rosa, CA

Fiechter, J., D. D. Huff, B. T. Martin, D. W. Jackson, C. A. Edwards, K. A. Rose, E. N. Curchitser, K. S. Hedstrom, S. T. Lindley, and B. K. Wells. 2015. Environmental conditions impacting juvenile Chinook salmon growth off central California: an ecosystem model analysis. Geophys. Res. Letters 42:2910-2917.

Fukushima, L. and E. W. Lesh. 1998. Adult and juvenile anadromous salmonid migration timing in California streams. Calif. Fish Game 84:133-145.

Gallagher, S. P. 2001. Steelhead (*Oncorhynchus mykiss*) and coho salmon (*Oncorhynchus kisutch*) juvenile population estimations and predictions in the Noyo River, California during spring and summer 2001. Report of the California Department of Fish and Game, North Coast Region Anadromous Fisheries Resource Assessment and Monitoring Program

Gallagher, S. P. 2003. Juveniles salmonid abundance estimation in the upper Noyo River, California during spring and summer 2002, Project 2a2. California Department of Fish and Game, North Coast Region, Steelhead Research and Monitoring Program.

Gallagher, S. P. 2004. Juvenile salmonid abundance estimation in the upper Noyo River, California during spring 2003, Project 2a2. Report of the California Dep.Fish and Game, North Coast Region, Anadromous Fisheries Resource Assessment and Monitoring Program.

Madej, M. A., A. Torregrosa, and A. Woodward. 2012. Linking physical monitoring to coho and Chinook salmon populations in the Redwood Creek watershed, California—Summary of May 304, 2012 Workshop. U.S. Geological Survey Open-File Report 2012-1245.

Murray, C. B. and J. D. McPhail. 1988. Effect of incubation temperature on the development of five species of Pacific salmon (*Oncorhynchus*) embryos and alevins. Can. J. Zool. 66:266-273.

Myers, J. M., R. G. Kope, G. J. Bryant, D. Teel, L. J. Lierheimer, T. C. Wainwright, S. W. Grant, W. F. Waknitz, K. Neely, S. T. Lindley, and R. S. Waples. 1998. Status review of Chinook salmon from Washington, Idaho, Oregon, and California. U.S. Dep. Commerce NOAA Tech. Memo. NMFS NWFSC-35.

NMFS (National Marine Fisheries Service). 2015. Coastal multi species recovery plan: California Coastal Chinook Salmon ESU, Northern California Steelhead ESU and Central California Coast Steelhead ESU. Volume III: Northern California Steelhead. U.S. Dep. Commerce, NOAA Fisheries West Coast Region.

O'Farrell, M. R., S. Allen-Moran, K. A. Atkinson, P. Dygert, S. Gallagher, A. Grover, B. Kormos, M. Lacy, E. Larson, M. Mohr, S. Ricker, W. Satterthwaite, and B. C. Spence. 2015. California coastal Chinook salmon fishery management: future prospects. U.S. Dep. Commerce NOAA Tech. Memo. NMFS-SWFSC-542.

O'Farrell, M. R., W. Satterthwaite, and B. C. Spence. 2012. California Coastal Chinook salmon: status, data, and feasibility of alternative fishery management strategies. U.S. Dep. Commerce NOAA Tech. Memo. NMFS SWFSC 494, Santa Cruz.

Satterthwaite, W. H., S. M. Carlson, S. D. Allen-Moran, S. Vincenzi, S. J. Bograd, and B. K. Wells. 2014a. Match-mismatch dynamics and the relationship between ocean-entry timing and relative ocean recoveries of Central Valley fall run Chinook salmon. Mar. Ecol. Prog. Ser. 511:237-248.

Satterthwaite, W. H., J. Ciancio, E. Crandall, M. L. Palmer-Zwahlen, A. M. Grover, M. R. O'Farrell, E. C. Anderson, M. S. Mohr, and J. C. Garza. 2015. Stock composition and ocean spatial distribution inference from California recreational Chinook salmon fisheries using genetic stock identification. Fish. Res. 170:166-178.

Satterthwaite, W. H., M. S. Mohr, M. R. O'Farrell, E. C. Anderson, M. A. Banks, S. J. Bates, M. R. Bellinger, L. A. Borgerson, E. D. Crandall, J. C. Garza, B. J. Kormos, P. W. Lawson, and M. L. Palmer-Zwahlen. 2014b. Use of genetic stock identification data for comparison of the ocean spatial distribution, size at age, and fishery exposure of an untagged stock and its indicator: California Coastal versus Klamath River Chinook salmon. Trans. Am. Fish. Soc. 143:117-133.

Schroeder, I. D., B. A. Black, W. J. Sydeman, S. J. Bograd, E. L. Hazen, J. A. Santora, and B. K. Wells. 2013. The North Pacific High and wintertime pre-conditioning of California current productivity. Geophys Res Lett 40:541-546.

Schroeder, I. D., W. J. Sydeman, N. Sarkar, S. A. Thompson, S. J. Bograd, and F. B. Schwing. 2009. Winter pre-conditioning of seabird phenology in the California Current. Mar. Ecol. Prog. Ser. 393:211-223.

Sparkman, M. D., R. Park, L. Osborn, S. Holt, and M. A. Wilzbach. 2016. Lower Redwood Creek juvenile salmonid (smolt) abundance project, 2004-2015 seasons. California Department of Fish and Game, Fisheries Restoration Grants Program project P1210322, Sacramento, CA.

Spence, B. C. 2016. North-Central California Coast Recovery Domain.*in* B. C. Williams, D. A. Spence, D. Boughton, R. C. Johnson, N. Crozier, M. Mantua, M. R. O'Farrell, and S. Lindley, editors. Viability assessment for Pacific salmon and steelhead listed under the Endangered Species Act: Southwest. U.S. Dep. Commerce NOAA Tech. Memo. NMFS-SWFSC-564.

Spence, B. C., E. Bjorkstedt, J. C. Garza, J. J. Smith, D. G. Hankin, D. Fuller, W. E. Jones, R. Macedo, T. H. Williams, and E. Mora. 2008. A framework for assessing the viability of threatened and endangered salmon and steelhead in the North Central California Coast Recovery Domain. U.S. Dep. Commerce NOAA Tech. Memo. NMFS-SWFSC-423.

Thayer, J. A., J. C. Field, and W. J. Sydeman. 2014. Changes in California Chinook salmon diet over the past 50 years: relevance to the recent population crash. Mar. Ecol. Prog. Ser. 498:249-261.

Thompson, S. A., W. J. Sydeman, J. A. Santora, B. A. Black, R. M. Suryan, J. Calambokidis, W. T. Peterson, and S. J. Bograd. 2012. Linking predators to seasonality of upwelling: Using food web indicators and path analysis to infer trophic connections. Prog Oceanogr 101:106-120.

Wallace, M. and S. Allen. 2009. Juvenile salmonid use of the tidal portions of selected tributaries to Humboldt Bay, California, 2007–2009. Final Report for Contract P0610522, California Dep.Fish and Wildlife Final Report for Contract P0610522.

Wells, B. K., J. A. Santora, J. C. Field, R. B. MacFarlane, B. B. Marinovic, and W. J. Sydeman. 2012. Population dynamics of Chinook salmon *Oncorhynchus tshawytscha* relative to prey availability in the central California coastal region. Mar. Ecol. Prog. Ser. 457:125-137.

Upper Willamette River Chinook

Overall vulnerability—Very high (15% High, 85% Very high)
Biological sensitivity—Very high (20% High, 80% Very high)
Climate exposure—High (78% High, 22% Very high)
Adaptive capacity—Moderate (1.6)
Data quality—74% of scores ≥ 2

	**Upper Willamette River Chinook**	Expert Scores	Data Quality	Expert Scores Plots (Portion by Category)
Sensitivity attributes	Early life history	1.4	3	
	Juvenile freshwater stage	2.9	3	
	Estuary stage	1.9	2.3	
	Marine stage	2.3	1.8	
	Adult freshwater stage	3.6	2.8	
	Cumulative life-cycle effects	3.3	2	
	Hatchery influence	3.8	2.5	
	Other stressors	3.5	3	
	Population viability	3.6	2.8	
	Ocean acidification sensitivity	1.9	1.8	
	Sensitivity Score	Very High		
Exposure variables	Stream temperature	3.6	2.3	
	Summer water deficit	2.4	1.8	
	Flooding	2.8	2	
	Hydrologic regime	3.2	2.5	
	Sea level rise	2	2.3	
	Sea surface temperature	3.4	2	
	Ocean acidification exposure	3.8	2.5	
	Upwelling	2.3	1.8	
	Ocean currents	1.8	1.3	
	Exposure Score	High		
Overall Vulnerability Rank		Very High		

Low
Moderate
High
Very High

Life History Synopsis

Upper Willamette River Chinook adults arrive in fresh water from late winter through early summer, hold in the river system, and mature during the warmest temperatures of the year. Spawning occurs from late summer through much of fall, with eggs incubating over winter. Juvenile life histories can be diverse (Schroeder et al. 2016). Fry emerge over a protracted period from early winter through mid-spring, with juveniles rearing in fresh water for 8-16 months and exhibiting diverse patterns of habitat use. Some juveniles migrate as subyearlings, with migration peaks in both spring and fall. However, the majority remain in fresh water for a full year prior to migration.

Estuarine use by subyearlings from this DPS can be extensive (Teel et al. 2014; Rose 2015), while yearlings migrate rapidly through the estuary 1-2 months earlier than other yearling Chinook smolts in the Columbia River (Weitkamp et al. 2015). Marine distribution of subyearlings is poorly understood, but yearlings move rapidly northward along the continental shelf during their first summer, and then likely rear in the central Gulf of Alaska (Fisher et al. 2014; Teel et al. 2014; Riddell et al. 2018). As adults, Upper Willamette River Chinook are widely dispersed in marine waters, from southeast Alaska to the Oregon coast (Weitkamp 2010). Most adults return after 2-3 years at sea. Because both juveniles and adults use habitats throughout the Willamette River year-round, they are exposed to climate risks in all seasons.

Climate Effects on Abundance and Distribution

Exposure attributes for Upper Willamette River Chinook salmon were ranked high overall, due to ***very high*** scores for *ocean acidification* and *stream temperature*. Mean August temperature was projected to increase 1.4°C by the 2040s, and 2.4°C by the 2080s. Other ***high*** exposure attributes included *sea surface temperature* and *hydrologic regime* shift. Although approximately 90% of the basin is already rain-dominated, the remaining 10% is very likely to change to rain-dominated by the 2040s. Scores for ocean acidification and sea surface temperature were similar to those of most DPSs.

Sensitivity attributes for this DPS were ranked very high due to a host of factors, including its ***very high*** vulnerability in the *adult freshwater stage* and ***very high*** *cumulative life-cycle effects* reflecting threats to the species' entire life cycle and to its life history diversity.

Extrinsic Factors

Upper Willamette River Chinook scored ***high*** or ***very high*** in all three extrinsic factor categories. Keefer and Caudill (2010) and Myers et al. (2018) highlighted the factors posing potential or demonstrated threats to Willamette River salmonids. Upper Willamette River Chinook is listed as threatened under the U.S. Endangered Species Act. This DPS has low numbers of wild adults, with the majority of production hatchery origin. Thus, Upper Willamette River Chinook was ranked ***very high*** for *population viability* and *hatchery influence*. A ***high*** ranking for *other stressors* reflected the following pressures on this DPS:

1) Loss of hundreds of kilometers of historical habitat due to impoundment by major dams
2) Low survival of fish transported above dams
3) High pre-spawn mortality of adults linked to impaired temperature and flow regime below dams
4) Widespread invasion of non-native species

5) A host of contaminants, ranging from the traditionally important, such as heavy metals, to the newer and more poorly understood, including nanoparticles, personal care products, and human and animal pharmaceuticals (Yeakley et al. 2014).

These and other fundamental transformations of the Willamette River will be difficult to reverse, given constraints imposed by human resource needs in a heavily populated watershed along with projected climate change impacts (Moore 2015).

Adaptive Capacity

Modified environments available to Chinook salmon in the Willamette River have exerted powerful selection pressures, such that the DPS itself may be fundamentally transforming. For example, in the Green Peter Reservoir, individuals have been collected that appear to have completed their entire life cycle in fresh water as the offspring of adfluvial parents rather than as hatchery releases (Romer and Monzyk 2014). Use of reservoirs may be under-reported, along with other juvenile life history patterns (Bourret et al. 2014). However, the extent to which alternate rearing patterns represent viable strategies vs. ecological traps is unknown (Bourret et al. 2014). Nonetheless, modified reservoir operations to benefit juvenile production are being considered (Johnson and Friesen 2014), despite uncertain outcomes (C. Murphy, Oregon State University, personal communication).

Upper Willamette River Chinook exhibits a remarkable ability to survive in such a highly altered system. However, given the elevated extrinsic pressure and depressed natural production of this DPS, its capacity for further adaption is unclear. Thus Upper Willamette River Chinook scored ***moderate*** for *adaptive capacity*.

Literature Cited

Information in this narrative was summarized from literature reviewed by Keefer and Caudil (2010), unless otherwise noted.

Bourret, S. L., B. P. Kennedy, C. C. Caudill, and P. M. DChittaro. 2014. Using otolith chemical and structural analysis to investigate reservoir habitat use by juvenile Chinook salmon *Oncorhynchus tshawytscha*. J. Fish. Biol. 85:1507-1525.

Fisher, J. P., L. A. Weitkamp, D. J. Teel, S. A. Hinton, J. A. Orsi, E. V. Farley, J. F. T. Morris, M. E. Thiess, R. M. Sweeting, and M. Trudel. 2014. Early ocean dispersal patterns of Columbia River Chinook and coho salmon. Trans. Am. Fish. Soc. 143:252-272.

Johnson, M. A. and T. A. Friesen. 2014. Genetic diversity and population structure of spring Chinook salmon from the upper Willamette River, Oregon. N. Am. J. Fish. Manage. 34:853-862.

Keefer, M. L. and C. C. Caudil. 2010. A review of adult salmon and steelhead life history and behavior in the Willamette River basin: identification of knowledge gaps and research needs. University of Idaho College of Natural Resoures, Moscow, ID. Available from http://www.webpages.uidaho.edu/uiferl/pdf%20reports/2010-08_Keefer-Caudill%20WIL%20lit%20review%20(FINAL).pdf.

Moore, K. M. 2015. Optimizing reservoir operations to adapt to 21st Century expectations of climate and social change in the Willamette River Basin, Oregon. PhD thesis, Oregon State University, Corvallis, OR. Available from https://ir.library.oregonstate.edu/concern/graduate_thesis_or_dissertations/jh343z011?locale=en.

Myers, J., J. Jorgensen, M. Sorel, M. Bond, T. Nodine, and R. Zabel. 2018. Upper Willamette River life cycle modeling and the potential effects of climate change. Report of the National Marine Fisheries Service to the U.S. Army Corps of Engineers, Portland, OR.

Riddell, B. E., R. D. Brodeur, Alexander V. Bugaev , P. Moran, J. s. M. Murphy, J. h. A. Orsi, M. Trudel, L. A. Weitkamp, B. K. Wells, and A. C. Wertheimer. 2018. The ocean ecology of Chinook salmon Pages 555-696 *in* R. J. Beamish, editor. The ocean ecology of Pacific salmon and trout. American Fisheries Society, Bethesda.

Romer, J. D. and F. R. Monzyk. 2014. Adfluvial life history in spring Chinook salmon from Quartzville Creek, Oregon. N. Am. J. Fish. Manage. 34:885-891.

Rose, G. W. 2015. Connecting tidal-fluvial life histories to survival of McKenzie River spring Chinook salmon (*Oncorhynchus tshawytscha*). M.S. Thesis, Oregon State University, Corvallis, OR. Available from: https://ir.library.oregonstate.edu/concern/graduate_thesis_or_dissertations/zc77st35f.

Schroeder, R. K., L. D. Whitman, B. Cannon, and P. Olmsted. 2016. Juvenile life-history diversity and population stability of spring Chinook salmon in the Willamette River basin, Oregon. Can. J. Fish. Aquat. Sci. 73:921-934.

Teel, D. J., D. L. Bottom, S. A. Hinton, D. R. Kuligowski, G. T. McCabe, R. McNatt, G. C. Roegner, L. A. Stamatiou, and C. A. Simenstad. 2014. Genetic identification of Chinook salmon in the Columbia River Estuary: Stock-specific distributions of juveniles in shallow tidal freshwater habitats. N. Am. J. Fish. Manage. 34:621-641.

Weitkamp, L. A. 2010. Marine distributions of Chinook salmon from the West Coast of North America determined by coded wire tag recoveries. Trans. Am. Fish. Soc. 139:147-170.

Weitkamp, L. A., D. J. Teel, M. Liermann, S. A. Hinton, D. M. Van Doornik, and P. J. Bentley. 2015. Stock-specific size and timing at ocean entry of Columbia River juvenile Chinook salmon and steelhead: Implications for early ocean growth. Mar. Coast. Fish. 7:370-392.

Yeakley, J. A., K. G. Maas-Hebner, and R. M. Hughes. 2014. Wild salmonids in the urbanizing Pacific Northwest. Springer, New York, NY.

Middle Columbia River spring-run Chinook

Overall vulnerability—High (48% High, 53% Very high)
Biological sensitivity—High (93% High, 0.07% Very high)
Climate exposure—High (52% High, 48% Very high)
Adaptive capacity—Moderate (2)
Data quality—79% of scores ≥ 2

	Middle Columbia River spring-run Chinook	Expert Scores	Data Quality	Expert Scores Plots (Portion by Category)
Sensitivity attributes	Early life history	1.3	2.5	
	Juvenile freshwater stage	3.5	2.5	
	Estuary stage	1.5	2.3	
	Marine stage	2.7	2	
	Adult freshwater stage	3.7	3	
	Cumulative life-cycle effects	3.2	2.5	
	Hatchery influence	2.7	2.5	
	Other stressors	3.3	3	
	Population viability	2.4	2.5	
	Ocean acidification sensitivity	1.9	1.8	
	Sensitivity Score	High		
Exposure variables	Stream temperature	3.4	2.3	
	Summer water deficit	3	1.8	
	Flooding	2.4	2	
	Hydrologic regime	3.7	2.5	
	Sea level rise	1.9	2.3	
	Sea surface temperature	3.4	2	
	Ocean acidification exposure	3.8	2.5	
	Upwelling	2.3	1.8	
	Ocean currents	1.8	1.3	
	Exposure Score	High		
Overall Vulnerability Rank		High		

Life History Synopsis

Middle Columbia River spring-run Chinook adults migrate to natal tributaries in April and May. Adults generally range from age-3 to -5, with age-4 dominating in most years (Myers et al. 1998). Several large tributaries to the Columbia support production of this DPS including the Deschutes, Klickitat, John Day and Yakima Rivers. These tributaries enter the Columbia upstream from its mainstem dams, with the number of dams in the migration corridor ranging from one for Klickitat River to four for Yakima River populations. Adults migrate directly to natal tributaries and then hold over summer for several weeks prior to spawning. Spawning occurs from late August through late September depending on flow and temperature in specific watersheds. Emergence occurs

in early spring, from February to May, with most populations peaking in March, again depending on watershed-specific temperature and flow.

Middle Columbia River spring-run Chinook exhibits a yearling life history, spending the first summer and winter in fresh water before the juvenile migration. Following the pattern seen in other interior Columbia spring runs, smolts move rapidly through the estuary (Weitkamp et al. 2015). After ocean entry, juveniles swim rapidly northward along the continental shelf (Fisher et al. 2014; Teel et al. 2015). Like other stream-type Chinook, the Middle Columbia River spring-run Chinook DPS is thought to have an oceanic marine distribution, and individuals are rarely caught in coastal fisheries (Healey 1983; Myers et al. 1998; Riddell et al. 2018).

This DPS is not listed under the ESA (Myers et al. 1998), but is identified as a sensitive species by the State of Oregon (ODFW 2016). The DPS includes populations existing under a range of habitat conditions within several major tributaries to the Columbia River (Lindsay et al. 1989; Bare et al. 2014). Populations in the lower elevation reaches of those tributaries are subject to higher temperatures during holding, spawning, and juvenile rearing and are also most impacted by degraded habitats. In some cases, impassable dams block access to higher-elevation habitats with colder temperatures that could support production under projected future climate change. Extirpated natural production areas in the Upper Deschutes Basin are the focus of ongoing restoration efforts (Lindsay et al. 1989).

Climate Effects on Abundance and Distribution

Across the Middle Columbia River spring-run Chinook DPS, the majority of populations are in watersheds transitioning from snow- to rain-driven flow patterns (Ruesch et al. 2012; Dittmer 2013; Clifton et al. 2018). There is considerable variation in flow and temperature regime within and across drainages used by this DPS. Summer rearing conditions can be limited by temperature and flow, either overall or for large sections of a majority of populations (Hatten et al. 2014). In upper-reach production areas, extant populations are generally intact, but in lower tributary production areas, declines in habitat quality have resulted in general losses of productive capacity. Most populations are subject to peak summer temperatures during the prolonged pre-spawn holding phase (Myers et al. 1998, Bare et al. 2014). The presence of tributary dams or irrigation diversions in most drainages may exacerbate exposure to high temperatures.

A ***high*** overall sensitivity rank for this DPS stemmed from the combination of its migration, adult holding, and juvenile rearing patterns. Negative effects of higher temperatures during the *adult* and *juvenile freshwater stages* have been documented (Ruesch et al. 2012; Tattam et al. 2015). Timing of the adult migration puts spawners at risk of exposure to increasing late spring/early summer temperatures (Keefer et al. 2008) through the Columbia River mainstem as well as the mainstems of large tributaries such as the Yakima, John Day, and Deschutes River. In addition, adults hold over summer in

upstream reaches prior to spawning in early fall. High pre-spawn mortality during this phase has been observed and is believed to be associated with increases in stream temperature and decreases in pool habitat (Bare et al. 2014). This DPS ranked ***very high*** for the *adult freshwater stage*.

Spring-run juveniles spend a full year in fresh water and experience negative effects on survival from high summer temperatures and low flows (Hatten et al. 2014; Tattam et al. 2015). This DPS ranked ***high*** in sensitivity at the *juvenile freshwater stage* and ***very high*** in exposure to *hydrologic regime* shift and *stream temperature*. Because the majority of populations exhibit the yearling life history, loss of this rearing strategy would mean significant loss of a characteristic of this DPS. Vulnerability at multiple life stages increases the risk for c*umulative life-cycle effects*. For example, changes in temperature and flow affect rearing, smolt survival, and migration timing, which in turn may affect early marine survival (Crozier et al. 2008).

The hydrology of most river basins supporting Middle-Columbia spring-run Chinook production are classified as transitional between rain-and snow-dominated flow patterns. Climate models project that these basins will shift towards rain-dominated flow regimes with earlier runoff peak flows and lower summer base flows (Dittmer 2013; Clifton et al. 2018), thus exposure to *hydrological regime* shift was ranked ***high*** for this DPS. These shifts would detrimentally affect juvenile rearing and adult pre-spawn life stages. Stream temperatures in the Mid-Columbia River tributaries used by this DPS for spawning and extended juvenile rearing are also warming rapidly, so exposure to *stream temperature* was scored ***high***.

Middle-Columbia spring-run Chinook exhibit ocean entry and return patterns similar to those of Snake River spring/summer Chinook. Although estimated smolt-to-adult return rates for this DPS are generally higher than those of Snake River conspecifics, they are strongly correlated with estimates for Snake River spring/summer Chinook. Based on these similarities, the Mid-Columbia spring Chinook DPS also ranked ***high*** in sensitivity at the *marine stage*. Marine survival is lower for this DPS during warm phases of the Pacific Decadal Oscillation, and rising *sea surface temperature* will likely have impacts similar to the warm ocean conditions related to both warm-phase PDO and low survival.

For Middle-Columbia spring Chinook, a longer juvenile freshwater residence period resulted in a ***high*** rank for sensitivity at the *juvenile freshwater stage*. This DPS scored ***low*** at the *estuary stage* due to its relatively short residence time in the estuary (Teel et al. 2015; Weitkamp et al. 2015).

Of primary concern in cumulative life-cycle effects is the loss of unique life history types historically enabled by the availability of a diverse set of habitats. Functional losses of downstream rearing habitat due to degraded habitat quality have already reduced or eliminated historical life history patterns in many populations (Ruesch

et al. 2012; Tattam et al. 2015). Cumulative effects from shifts in successive life stages may also reduce survival in subsequent life stages. For example, earlier migration timing at the juvenile freshwater stage may mean fish are smaller at ocean entry, which in turn may reduce survival at the early ocean stage. Thus, sensitivity of this DPS was considered ***high*** for *cumulative life-cycle effects*.

Extrinsic Factors

Freshwater habitat loss and reduction in spatial structure are important concerns for this DPS compared with others in this assessment. While some tributary spawning and rearing habitats are in relatively pristine wilderness areas, most production areas for this DPS are highly impacted by anthropogenic factors associated with agriculture, roads and land development. Smallmouth bass, an introduced warm-water species and predator on young-of-the-year Chinook, are present in several tributaries that support juvenile rearing. Increasing temperatures are allowing bass to extend further upstream, potentially overlapping juvenile spring Chinook rearing areas (Lawrence et al. 2012). Tributaries that support spawning and rearing are located upstream from between one and four dams on the mainstem Columbia River. As a result, dam passage impacts in warm years can potentially vary in magnitude across populations. All of these threats contributed to a ***high*** rank for this DPS in sensitivity to *other stressors*.

Adaptive Capacity

Middle-Columbia River spring-run Chinook salmon DPS was ranked ***moderate*** in adaptive capacity overall. Some populations within this DPS may have sufficient adaptive capacity to reduce juvenile freshwater residence time, either by migrating earlier in the year or by increasing the proportion of subyearling migrants. Adults may have the potential to shift migration timing earlier to avoid high stream temperatures in the migration corridor. However, early migrant adults in this DPS will still need to hold for extended periods until temperatures cool in the fall, and this will increase exposure to high *stream temperature*. Increased energetic costs during the holding period could limit adaptive capacity in the adult stage.

Literature Cited

Bare, C. M., J. L. Latshaw, I. A. Tattam, J. R. Ruzycki, and R. W. Carmichael. 2014. Chinook Salmon productivity and escapement monitoring in the John Day River basin. Oregon Dep. Fish Wildl. Tech. Rep. OWEB Contr. 212-909.

Clifton, C. C., K. T. Dary, C. H. Luce, G. E. Grant, M. Safeeq, J. E. Halofsky, and B. P. Staub. 2018. Effects of climate change on hydrology and water resources in the Blue Mountains, Oregon, USA. Climate Serv. **10**:9-19.

Crozier, L. G., A. P. Hendry, P. W. Lawson, T. P. Quinn, N. J. Mantua, J. Battin, R. G. Shaw, and R. B. Huey. 2008. Potential responses to climate change in organisms with complex life histories: evolution and plasticity in Pacific salmon. Evol. Appl. **1**:252-270.

Dittmer, K. 2013. Changing streamflow on Columbia basin tribal lands—climate change and salmon. Climate Change **120**:627–641.

Fisher, J. P., L. A. Weitkamp, D. J. Teel, S. A. Hinton, J. A. Orsi, E. V. Farley, J. F. T. Morris, M. E. Thiess, R. M. Sweeting, and M. Trudel. 2014. Early ocean dispersal patterns of Columbia River Chinook and coho salmon. Trans. Am. Fish. Soc. **143**:252-272.

Hatten, J. R., T. R. Batt, P. J. Connolly, and A. W. Maule. 2014. Modeling effects of climate change on Yakima River salmonid habitats. Climatic Change **124**:427-439.

Healey, M. C. 1983. Coastwide distribution and ocean migration patterns of stream- and ocean-type Chinook salmon, *Oncorhynchus tshawytscha*. Can. Field-Nat. **97**.

Keefer, M. L., C. A. Peery, and C. C. Caudill. 2008. Migration timing of Columbia River spring Chinook salmon: effects of temperature, river discharge, and ocean environment. Trans. Am. Fish. Soc. **137**:1120-1133.

Lawrence, D. J., J. D. Olden, and C. E. Torgensen. 2012. Spatiotemporal patterns and habitat associations of smallmouth bass (*Micropterus dolomieu*) invading salmon-rearing habitat. Freshw. Biol. **57**:1929-1946.

Lindsay, R. B., B. C. Jonasson, S. R.K., and B. C. Cates. 1989. Spring Chinook Salmon in the Deschutes River, Oregon. Report of the Oregon Dep. Fish Wildl.

Myers, J. M., R. G. Kope, G. J. Bryant, D. Teel, L. J. Lierheimer, T. C. Wainwright, S. W. Grant, W. F. Waknitz, K. Neely, S. T. Lindley, and R. S. Waples. 1998. Status review of Chinook salmon from Washington, Idaho, Oregon, and California. U.S. Dep. Commerce NOAA Tech. Memo. NMFS-NWFSC-35.

ODFW (Oregon Department of Fish and Wildlife). 2016 Sensitive Species List. Oregon Department of Fish and Wildlife. Avaialble from www.dfw.state.or.us/wildlife/diversity/species/docs/2017_Sensitive_Species_List.pdf.

Riddell, B. E., R. D. Brodeur, Alexander V. Bugaev , P. Moran, J. s. M. Murphy, J. h. A. Orsi, M. Trudel, L. A. Weitkamp, B. K. Wells, and A. C. Wertheimer. 2018. The ocean ecology of Chinook salmon Pages 555-696 *in* R. J. Beamish, editor. The ocean ecology of Pacific salmon and trout. American Fisheries Society, Bethesda, MD.

Ruesch, A. A., C. E. Torgersen, J. J. Lawler, J. D. Olden, E. E. Peterson, C. J. Volk, and D. J. Lawrence. 2012. Projected climate-induced habitat loss for salmonids in the John Day River network, Oregon, U.S.A. Trans. Am. Fish. Soc. **144**:1237-1248.

Tattam, I. A., J. R. Ruzycki, J. L. McCormick, and R. W. Carmichael. 2015. Length and condition of wild Chinook salmon smolts influence age at maturity. Trans. Am. Fish. Soc. **145**:1237-1248.

Teel, D. J., B. J. Burke, D. R. Kuligowski, C. A. Morgan, and D. M. Van Doornik. 2015. Genetic identification of Chinook salmon: Stock-specific distributions of juveniles along the Washington and Oregon Coasts. Mar. Coast. Fish. **7**:274-300.

Weitkamp, L. A., D. J. Teel, M. Liermann, S. A. Hinton, D. M. Van Doornik, and P. J. Bentley. 2015. Stock-specific size and timing at ocean entry of Columbia River

juvenile Chinook salmon and steelhead: Implications for early ocean growth. Mar. Coast. Fish. **7**:370-392.

Snake River spring/summer-run Chinook

Overall vulnerability—High (54% High, 46% Very high)
Biological sensitivity—High (97% High, 3% Very high)
Climate exposure—Very high (55% High, 45% Very high)
Adaptive capacity—High (2.1)
Data quality—79% of scores ≥ 2

	Snake River spring/summer-run Chinook	Expert Scores	Data Quality	Expert Scores Plots (Portion by Category)
Sensitivity attributes	Early life history	1.5	2.8	
	Juvenile freshwater stage	3.5	3	
	Estuary stage	1.5	2.8	
	Marine stage	2.9	2.3	
	Adult freshwater stage	3.5	3	
	Cumulative life-cycle effects	3.1	2.5	
	Hatchery influence	2.9	2.5	
	Other stressors	3.1	2.8	
	Population viability	3.3	2.8	
	Ocean acidification sensitivity	1.9	1.8	
	Sensitivity Score	High		
Exposure variables	Stream temperature	3.5	2.3	
	Summer water deficit	2.7	1.8	
	Flooding	2.3	2	
	Hydrologic regime	3.5	2.5	
	Sea level rise	1.8	2.3	
	Sea surface temperature	3.4	2	
	Ocean acidification exposure	3.8	2.5	
	Upwelling	2.3	1.8	
	Ocean currents	2	1.3	
	Exposure Score	Very High		
Overall Vulnerability Rank		Very High		

Low
Moderate
High
Very High

Life History Synopsis

Snake River spring/summer Chinook adults enter the Columbia River from April through June. Within about 2-3 weeks, most adults in this DPS pass eight major dams spanning 462 km of the Columbia and Snake River-s. The Tucannon River population leaves the Snake River downstream from Lower Granite Dam, and the Grande Ronde populations leave the Snake River downstream from its confluence with the Salmon River. Salmon River populations continue another 800-1500 km to elevations as high as 2000 m, with a full migration that usually spans several months.

Adults in this DPS typically arrive in tributaries at least one month before spawning, and hold over the summer in deep pools before spawning in August or

September. Populations at higher elevation streams spawn earlier than those at lower elevations. Eggs incubate during winter and hatch in spring. Parr rear near spawning areas or in lower elevation reaches, such as the Salmon and Grand Ronde Rivers. However, little is known about specific overwintering habitat usage or impacts of overwintering location on survival. The majority of juveniles from these populations spend a whole year in freshwater before the smolt transition in April and May.

Most fish spend little time in estuary habitats and exhibit a rapid, directed juvenile migration. Once they enter coastal waters, these fish move north along the continental shelf (Fisher et al. 2014; Teel et al. 2014; Weitkamp et al. 2015). Like other stream-type Chinook salmon, Snake River spring/summer Chinook are thought to have oceanic marine distributions and are rarely caught by coastal fisheries (Healey 1983; Myers et al. 1998; Riddell et al. 2018). Most adults return after 2-3 years at sea, although some, mostly hatchery fish and males, return after 1 year.

Climate Effects on Abundance and Distribution

Snake River spring/summer Chinook salmon has been closely studied as a threatened and indicator species and is the subject of life-cycle modeling under climate change conditions. The ***high*** overall sensitivity rank of this DPS stemmed largely from characteristics of its migration. Negative effects of high temperatures encountered during the adult and juvenile freshwater stages have been documented (Crozier and Zabel 2006; Crozier et al. 2017a,b). Populations within this DPS that migrate later are called summer-run fish. Examples are the Pahsimeroi and South Fork Salmon River populations, which encounter stressful temperatures during the adult migration. However, both spring- and summer-run populations are at risk for pre-spawn mortality while holding in tributary habitats during peak summer temperatures (Bowerman et al. 2016). This DPS was ranked ***very high*** for the adult freshwater stage.

Because juveniles spend a full year in fresh water, they can experience negative effects on survival from warm summer temperatures and low flows (Crozier and Zabel 2006; Crozier et al. 2008b). Juvenile survival during the smolt migration depends strongly on rapid flows from snowmelt (Zabel et al. 2008; Faulkner et al. 2018). Thus, sensitivity in the *juvenile freshwater stage* was ranked ***high***. The Interior Columbia recovery domain is likely to lose a substantial portion of snowpack, so this DPS was ranked ***very high*** for *hydrologic regime* shift. Snake River spring/summer-run Chinook also ranked ***very high*** in exposure to *stream temperature* change, elevating its vulnerability to ***very high*** in both the *juvenile* and *adult freshwater stages*.

A vast majority of populations in this DPS exhibit the yearling life history strategy. Therefore, loss of this rearing strategy would mean loss of a significant characteristic of this DPS, a threat reflected in the ***high*** score for *cumulative life-cycle effects*. Carryover effects between life stages also increased the *cumulative life-cycle effects* risk, as discussed below.

Snake River spring/summer Chinook sensitivity was ranked ***moderate*** at the *marine stage*, although some scorers considered the marine mortality risk to be high. Marine survival for this DPS is lower during warm phases of the Pacific Decadal Oscillation, and rising *sea surface temperature* will likely have impacts similar to the warm ocean conditions related with both warm phases of the PDO and low adult survival (Zabel et al. 2006; Crozier et al. 2008b). On the other hand, while the smolt migration is slower in low snowpack years, earlier smolt migration timing might benefit this DPS in relation to ocean upwelling. At present, much of the population enters the ocean later than the optimal period for survival (Scheuerell et al. 2009).

Snake River spring/summer Chinook juveniles have a relatively short estuary rearing period (Weitkamp et al. 2012, 2015), which resulted in its ***low*** rank in sensitivity at the *estuary stage*. Longer freshwater rearing is generally observed to produce larger smolts, which then typically spend less time in the estuary.

Of primary concern in the *cumulative life-cycle effects* attribute was a loss of unique life history types, including the spring/summer adult run type and the yearling juvenile life history type. Cumulative effects from shifts in successive life stages may reduce survival in subsequent life stages. For example, earlier migration timing at the juvenile freshwater stage may mean fish are smaller at ocean entry and less likely to encounter favorable ocean feeding conditions. Such a timing alteration could in turn reduce early marine survival (Crozier et al. 2008a). Thus, this DPS ranked ***high*** in sensitivity to *cumulative life-cycle effects.*

Extrinsic Factors

Snake River spring/summer Chinook is listed as threatened, with most populations at high risk (Ford et al. 2011), especially in the upper Grande Ronde and upper Salmon Rivers. Estimated extinction risk under climate change scenarios is significantly higher than under the historical climate regime (Crozier and Zabel 2013). At present, abundance is low for this DPS, and it is considered highly sensitive to marine conditions, owing to rapid declines in abundance during poor ocean years (Zabel et al. 2006). Early returning runs are susceptible to increased marine mammal predation in the lower Columbia River (Sorel et al. 2017). Hatchery production of fish in this DPS is substantial; among yearling Chinook passing Lower Granite Dam in 2017, 86% were estimated to be of hatchery origin (Faulkner et al. 2018). Nonetheless, some hatcheries do have supplementation control rules in place that prioritize conservation objectives. Therefore, this DPS scored ***moderate*** in sensitivity hatchery influence.

Habitat loss and reduction in spatial structure are lesser concerns for much of this DPS compared with others in this assessment because a relatively large proportion of its spawning habitat is in protected wilderness area. Nonetheless, some populations are highly impacted by anthropogenic factors (e.g., Upper Grand Ronde and Lower Snake), especially land development and agriculture. Major dams throughout the migratory

corridor can hinder passage of both juveniles and adults, especially in warm years. Therefore, this DPS ranked ***high*** in sensitivity to *other stressors*.

Adaptive Capacity

Snake River spring/summer Chinook salmon may have sufficient adaptive capacity to increase the production of subyearling smolts, or for yearling smolts to migrate earlier in spring. Adults may have some flexibility in migration timing to avoid high stream temperatures in the migration corridor. However, it is not clear whether subyearling smolts will be viable in the future because they currently have very low adult return rates. Furthermore, early migrating adults in this DPS still need to hold for extended periods until temperatures cool in fall, and such holding will increase exposure to high stream temperatures and risk of harvest. Increased energetic cost during the holding period might also limit adaptive capacity in the adult stage. Thus behavioral changes could ultimately reduce abundance in the basin. Nonetheless, this DPS ranked ***high*** in *adaptive capacity*.

Literature Cited

Bowerman, T., M. L. Keefer, and C. C. Caudill. 2016. Pacific salmon prespawn mortality: patterns, methods, and study design considerations. Fisheries 41:738-749.

Crozier, L., L. Wiesebron, E. Dorfmeier, and B. Burke. 2017a. River conditions, fisheries and fish history drive variaion in upstream survival and fallback for Upper Columbia River spring and Snake River spring/summer Chinook salmon. Report of the National Marine Fisheries Service.

Crozier, L., and R. W. Zabel. 2006. Climate impacts at multiple scales: evidence for differential population responses in juvenile Chinook salmon. J. Anim. Ecol. 75:1100-1109.

Crozier, L. G., T. E. Bowerman, B. J. Burke, M. L. Keefer, and C. C. Caudill. 2017b. High-stakes steeplechase: a behavior-based model to predict individual travel times through diverse migration segments. Ecosphere 8:e01965-n/a.

Crozier, L. G., A. P. Hendry, P. W. Lawson, T. P. Quinn, N. J. Mantua, J. Battin, R. G. Shaw, and R. B. Huey. 2008a. Potential responses to climate change in organisms with complex life histories: evolution and plasticity in Pacific salmon. Evol. Appl. 1:252-270.

Crozier, L. G., and R. W. Zabel. 2013. Population responses of spring/summer Chinook salmon to projected changes in stream flow and temperature in the Salmon River Basin, Idaho.*in* R. W. Zabel, T. D. Cooney, and C. E. Jordan, editors. Life cycle models of interior Columbia River populations. Report of the National Marine Fisheries Service.

Crozier, L. G., R. W. Zabel, and A. F. Hamlett. 2008b. Predicting differential effects of climate change at the population level with life-cycle models of spring Chinook salmon. Global Change Biol. 14:236-249.

Faulkner, J. R., D. L. Widener, S. G. Smith, T. M. Marsh, and R. W. Zabel. 2018. Survival estimates for the passage of spring migrating juvenile salmonids through Snake and Columbia River dams and reservoirs, 2017. Report of the National Marine Fisheries Service to the Bonneville Power Administration, Portland, OR.

Fisher, J. P., L. A. Weitkamp, D. J. Teel, S. A. Hinton, J. A. Orsi, E. V. Farley, J. F. T. Morris, M. E. Thiess, R. M. Sweeting, and M. Trudel. 2014. Early ocean dispersal patterns of Columbia River Chinook and coho salmon. Trans. Am. Fish. Soc. 143:252-272.

Ford, M. J., A. Albaugh, K. Barnas, T. Cooney, J. Cowen, J. J. Hard, R. G. Kope, M. M. McClure, P. McElhany, J. M. Myers, N. J. Sands, D. J. Teel, and L. A. Weitkamp. 2011. Status review update for Pacific salmon and steelhead listed under the Endangered Species Act: Pacific Northwest. U.S. Dep. Commerce NMFS-NWFSC-113.

Healey, M. C. 1983. Coastwide distribution and ocean migration patterns of stream- and ocean-type Chinook salmon, *Oncorhynchus tshawytscha*. Can. Field-Nat. 97.

Myers, J. M., R. G. Kope, G. J. Bryant, D. Teel, L. J. Lierheimer, T. C. Wainwright, S. W. Grant, W. F. Waknitz, K. Neely, S. T. Lindley, and R. S. Waples. 1998. Status review of Chinook salmon from Washington, Idaho, Oregon, and California. U.S. Dep. Commerce NOAA Tech. Memo. NMFS-NWFSC-35.

Riddell, B. E., R. D. Brodeur, Alexander V. Bugaev , P. Moran, J. s. M. Murphy, J. h. A. Orsi, M. Trudel, L. A. Weitkamp, B. K. Wells, and A. C. Wertheimer. 2018. The ocean ecology of Chinook salmon Pages 555-696 *in* R. J. Beamish, editor. The ocean ecology of Pacific salmon and trout. American Fisheries Society, Bethesda, MD.

Scheuerell, M. D., R. W. Zabel, and B. P. Sandford. 2009. Relating juvenile migration timing and survival to adulthood in two species of threatened Pacific salmon (*Oncorhynchus* spp.). J. Appl. Ecol. 46:983–990.

Sorel, M. H., A. M. W. Rub, and R. W. Zabel. 2017. Population-specific migration timing affects en route survival of adult Chinook salmon through the Lower Columbia River. Chapter 6.a in Interior Columbia Basin Life-Cycle Modeling. Report of the National Marine Fisheries Service.

Teel, D. J., D. L. Bottom, S. A. Hinton, D. R. Kuligowski, G. T. McCabe, R. McNatt, G. C. Roegner, L. A. Stamatiou, and C. A. Simenstad. 2014. Genetic identification of Chinook salmon in the Columbia River Estuary: Stock-specific distributions of juveniles in shallow tidal freshwater habitats. N. Am. J. Fish. Manage. 34:621-641.

Weitkamp, L. A., P. J. Bentley, and M. N. C. Litz. 2012. Seasonal and interannual variation in juvenile salmonids and associated fish assemblage in open waters of the lower Columbia River estuary. Fish. Bull. U.S. 110:426-450.

Weitkamp, L. A., D. J. Teel, M. Liermann, S. A. Hinton, D. M. Van Doornik, and P. J. Bentley. 2015. Stock-specific size and timing at ocean entry of Columbia River juvenile Chinook salmon and steelhead: Implications for early ocean growth. Mar. Coast. Fish. 7:370-392.

Zabel, R. W., J. Faulkner, S. G. Smith, J. J. Anderson, C. Van Holmes, N. Beer, S. Iltis, J. Krinke, G. Fredricks, B. Bellerud, J. Sweet, and A. Giorgi. 2008. Comprehensive passage (COMPASS) model: a model of downstream migration and survival of juvenile salmonids through a hydropower system. Hydrobiologia 609:289-300.

Zabel, R. W., M. D. Scheuerell, M. M. McClure, and J. G. Williams. 2006. The interplay between climate variability and density dependence in the population viability of Chinook salmon. Conserv. Biol. 20:190-200.

Snake River fall-run Chinook

Overall vulnerability—High (35% Moderate, 65% High)
Biological sensitivity—High (35% Moderate, 65% High)
Climate exposure—High (100% High)
Adaptive capacity—High (2.3)

	Snake River fall-run Chinook	Expert Scores	Data Quality	Expert Scores Plots (Portion by Category)
Sensitivity attributes	Early life history	1.3	3	
	Juvenile freshwater stage	1.5	3	
	Estuary stage	2.1	2	
	Marine stage	2.6	2	
	Adult freshwater stage	2.9	2.8	
	Cumulative life-cycle effects	2.5	2.3	
	Hatchery influence	3.7	2.8	
	Other stressors	3	3	
	Population viability	2.3	2.8	
	Ocean acidification sensitivity	2	1.8	
	Sensitivity Score	High		
Exposure variables	Stream temperature	3.5	2.3	
	Summer water deficit	2	1.8	
	Flooding	2.4	2	
	Hydrologic regime	2.1	2.5	
	Sea level rise	2.4	2.3	
	Sea surface temperature	3	2	
	Ocean acidification exposure	3.8	2.5	
	Upwelling	2.3	1.8	
	Ocean currents	1.9	1.3	
	Exposure Score	High		
Overall Vulnerability Rank		High		

Low
Moderate
High
Very High

Life History Synopsis

Snake River fall-run Chinook adults enter the lower Columbia River from late summer through early fall (August-October). Females are predominately age 4-5, with small proportions of age-3 and age-6 spawners, whereas adult males are predominately age 3-5. Hatchery adults are skewed toward younger ages (~3 years), particularly among males. After entering the Columbia River, the majority of adults migrate over 400 km to pass Lower Granite Dam typically within 2-3 weeks. Because this DPS is close to maturity upon freshwater entry, individuals spawn a relatively short time after entering fresh water, from late October to early November (Connor et al. 2003b). Eggs incubate in stream gravel through winter, and fry emerge from early April in the Hells Canyon reach of the Snake River (where most individuals return to spawn) to late May in the Grande Ronde and Imnaha Rivers (Connor et al. 2003b). Low winter temperatures may

extend incubation times or limit the suitability of egg incubation in some locations (Connor et al. 2003b).

Warm temperatures in summer may limit the time that juveniles spend near natal locations, except in the cooler Clearwater River (Connor et al. 2005). Most juveniles below the Clearwater River migrate as subyearlings, whereas within the Clearwater, a small proportion migrates as yearlings. Hatchery releases are a mixture of subyearling and yearling juveniles. All upper Snake and Clearwater rearing locations produce some juveniles that overwinter in the lower Snake and Columbia Rivers. The extent of estuary use and residence by these juveniles is not well known, but individuals are present in the estuary during summer and early fall (Teel et al. 2014). During the first summer of ocean residency, both subyearlings and yearlings remain in waters near the Columbia River mouth, ranging south off Oregon and north of the West Coast of Vancouver Island (Fisher et al. 2014, Teel et al. 2015). Adults are captured in coastal fisheries from Southeast Alaska to Oregon (Weitkamp 2010).

Climate Effects on Abundance and Distribution

For Snake River fall-run Chinook, the upstream migration and pre-spawn holding period extends from mid-August through October (Connor et al. 2019). Returning adults are exposed to temperatures exceeding 20°C, with cumulative exposures highest for early-returning adults (Keefer and Caudill 2015). This DPS ranked ***high*** for exposure to *stream temperature* in the Snake River Basin, and models suggest that future migrants may experience lower migration and spawning success due to rising temperatures (Connor et al. 2018). Nonetheless, vulnerability of this DPS during the *adult freshwater stage* was ranked ***moderate*** because most adults migrate after temperatures have peaked and spawn after temperatures have declined in the fall.

Egg development is constrained by cold winter temperatures (Connor et al. 2003b), so rising stream temperature during incubation is of less concern for this DPS. This was reflected in the ***low*** sensitivity score for *early life history*. Juvenile emergence, growth, and migration occur earlier in warmer than in cooler spawning and rearing areas, and certain areas not used as spawning habitat appear to be too cold (Connor and Burge 2003, Connor et al. 2003a,b). Juveniles have been observed to tolerate constant temperatures of 22°C and fluctuating temperatures up to 27°C (Geist et al. 2010) and to grow well in these conditions, even at reduced rations (Geist et al. 2011).

During the predominantly subyearling migration of this DPS, rapid growth and thermoregulatory behavior (Tiffan et al. 2009) allow fish to avoid thermal stress, despite generally warm summer temperatures in the lower Snake River (Connor and Burge 2003). Juveniles rearing in cooler reaches of the Clearwater River have shown a yearling life history, and hence adaptability to a wide temperature range. Thus Snake River fall-run Chinooke ranked ***low*** in sensitivity at the *juvenile freshwater stage*.

Snake River fall-run Chinook exhibits a wide distribution across the eastern Pacific Ocean, ranging from coastal British Columbia to California and Oregon. A moderate relationship has been reported between survival of subyearlings and the Pacific Decadal Oscillation, and between the northern copepod anomaly index and survival of Columbia River fall chinook (Peterson et al. 2014). These findings were reflected in a ***moderate*** score for *marine stage*.

Extrinsic Factors

Snake River fall-run Chinook salmon production is dominated by hatcheries, and the DPS received a ***very high*** score for hatchery influence on climate resilience. Extrinsic factors in lower reaches of the Snake and Columbia River range from an increasing proliferation of non-native species (Sanderson et al. 2009) to a growing list of contaminants (Yeakley et al. 2014). Mainstem dams on the Snake and Columbia present challenges for both juvenile and adult migration (Smith et al. 2003; Keefer and Caudill 2015). These factors were reflected in a ***high*** sensitivity score for *other stressors*.

Adaptive Capacity

Despite considerable hatchery influence, the Snake River fall-run Chinook DPS has extensive opportunity for habitat shift and flexibility in age at juvenile migration, resulting in an overall ***high*** rank for adaptive capacity.

Literature Cited

Connor, W. P., and H. L. Burge. 2003. Growth of wild subyearling fall Chinook salmon in the Snake River. N. Am. J. Fish. Manage. 23:594–599.

Connor, W. P., H. L. Burge, J. R. Yearsley, and T. C. Bjornn. 2003a. Influence of flow and temperature on survival of wild subyearling fall Chinook salmon in the Snake River. N. Am. J. Fish. Manage. 23:362–375.

Connor, W. P., C. E. Piston, and A. P. Garcia. 2003b. Temperature during incubation as one factor affecting the distribution of Snake River fall Chinook salmon spawning areas. Trans. Am. Fish. Soc. 132:1236-1243.

Connor, W. P., J. G. Sneva, K. F. Tiffan, R. K. Steinhorst, and D. Ross. 2005. Two alternative juvenile life history types for fall Chinook salmon in the Snake River Basin. Trans. Am. Fish. Soc. 134:291-304.

Connor, W. P., K. F. Tiffan, J. A. Chandler, D. W. Rondorf, B. D. Arnsberg, and K. C. Anderson. 2019. Upstream migration and spawning success of Chinook salmon in a highly developed, seasonally warm river system. Rev. Fish. Sci. Aquacult. 27(1):1-50 https://doi.org/10.1080/23308249.2018.1477736.

Fisher, J. P., L. A. Weitkamp, D. J. Teel, S. A. Hinton, J. A. Orsi, E. V. Farley, J. F. T. Morris, M. E. Thiess, R. M. Sweeting, and M. Trudel. 2014. Early ocean dispersal patterns of Columbia River Chinook and coho salmon. Trans. Am. Fish. Soc. 143:252-272.

Geist, D. R., Z. Deng, R. P. Mueller, S. R. Brink, and J. A. Chandler. 2010. Survival and growth of juvenile Snake River fall Chinook salmon exposed to constant and fluctuating temperatures. Trans. Am. Fish. Soc. 139:92-107.

Geist, D. R., Z. Deng, R. P. Mueller, V. Cullinan, S. Brink, and J. A. Chandler. 2011. The effect of fluctuating temperature and ration levels on the growth of juvenile Snake River fall Chinook salmon. Trans. Am. Fish. Soc. 142:1299-1307.

Keefer, M. L., and C. C. Caudill. 2015. Estimating thermal exposure of adult summer steelhead and fall Chinook salmon migrating in a warm impounded river. Ecol. Freshw. Fish. 25:599-611.

Peterson, W. T., J. L. Fisher, J. O. Peterson, C. A. Morgan, B. J. Burke, and K. L. Fresh. 2014. Applied fisheries oceanography: ecosystem indicators of ocean conditions inform fisheries management in the California Current. Oceanography 27:80-89.

Sanderson, B. L., K. A. Barnas, and A. M. W. Rub. 2009. Nonindigenous species of the Pacific Northwest: an overlooked risk to endangered salmon? BioScience 59:245-256.

Smith, S. G., W. D. Muir, E. E. Hockersmith, R. W. Zabel, R. J. Graves, C. V. Ross, and W. P. Connor. 2003. Influence of river conditions on survival and travel time of Snake River subyearling fall Chinook Salmon. N. Am. J. Fish. Manage. 23:939–961.

Teel, D. J., D. L. Bottom, S. A. Hinton, D. R. Kuligowski, G. T. McCabe, R. McNatt, G. C. Roegner, L. A. Stamatiou, and C. A. Simenstad. 2014. Genetic identification of Chinook salmon in the Columbia River Estuary: Stock-specific distributions of juveniles in shallow tidal freshwater habitats. N. Am. J. Fish. Manage. 34:621-641.

Teel, D. J., B. J. Burke, D. R. Kuligowski, C. A. Morgan, and D. M. Van Doornik. 2015. Genetic identification of Chinook salmon: Stock-specific distributions of juveniles along the Washington and Oregon Coasts. Mar. Coast. Fish. 7:274-300.

Tiffan, K. F., T. J. Kock, W. P. Connor, R. K. Steinhorst, and D. W. Rondorf. 2009. Behavioural thermoregulation by subyearling fall (autumn) Chinook salmon *Oncorhynchus tshawytscha* in a reservoir. J. Fish. Biol. 74:1562-1579.

Weitkamp, L. A. 2010. Marine distributions of Chinook salmon from the West Coast of North America determined by coded wire tag recoveries. Trans. Am. Fish. Soc. 139:147-170.

Yeakley, J. A., K. G. Maas-Hebner, and R. M. Hughes. 2014. Wild salmonids in the urbanizing Pacific Northwest. Springer, New York.

Upper Columbia River spring-run Chinook

Overall vulnerability—High (73% High, 27% Very high)
Biological sensitivity—High (84% High, 16% Very high)
Climate exposure—High (86% High, 14% Very high)
Adaptive capacity—Moderate (1.8)
Data quality—79% of scores ≥ 2

	Upper Columbia River spring-run Chinook	Expert Scores	Data Quality	Expert Scores Plots (Portion by Category)
Sensitivity attributes	Early life history	1.5	2.8	
	Juvenile freshwater stage	3.5	3	
	Estuary stage	1.5	2.8	
	Marine stage	2.9	2.3	
	Adult freshwater stage	3.5	3	
	Cumulative life-cycle effects	2.8	2.3	
	Hatchery influence	3.1	2.5	
	Other stressors	3.1	3	
	Population viability	3.5	2.8	
	Ocean acidification sensitivity	1.9	1.8	
	Sensitivity Score	High		
Exposure variables	Stream temperature	3.2	2.3	
	Summer water deficit	2.9	1.8	
	Flooding	2.4	2	
	Hydrologic regime	3.5	2.5	
	Sea level rise	1.8	2.3	
	Sea surface temperature	3.4	2	
	Ocean acidification exposure	3.8	2.5	
	Upwelling	2.3	1.8	
	Ocean currents	1.8	1.3	
	Exposure Score	High		
Overall Vulnerability Rank		High		

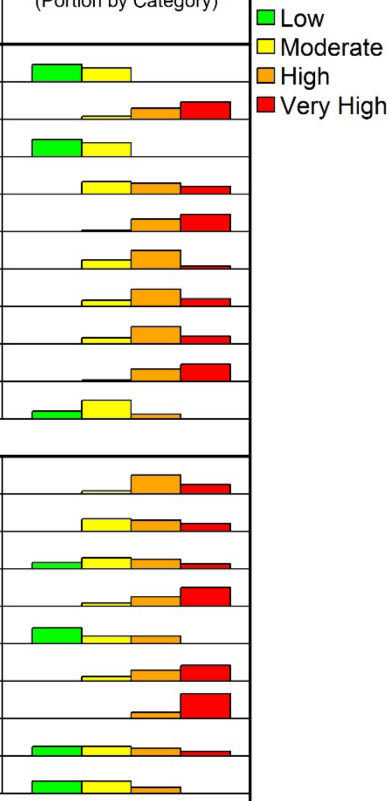

Life History Synopsis

Upper Columbia River spring-run adults enter the Columbia River as an early part of the aggregate run of spring/summer Chinook salmon, passing Bonneville Dam in April and May (Keefer et al. 2008; Crozier et al. 2016). These fish migrate to three major spawning areas and hold over summer in deep, cool pools before spawning in August or September. Eggs incubate over winter and hatch in spring. The majority of juveniles from these populations spend a full year in fresh water before the downstream migration in April and May. Like other yearling Chinook migrants, juveniles from these populations have a relatively brief estuary residency (Teel et al. 2014; Weitkamp et al. 2015). During their first summer in marine waters, they rapidly move northward along the continental shelf (Fisher et al. 2014; Teel et al. 2015). Also like other stream-type

Chinook, the Upper Columbia spring Chinook salmon DPS is thought to have an oceanic marine distribution, and individuals are rarely caught by coastal fisheries (Healey 1991; Myers et al. 1998; Riddell et al. 2018). Although some fish return after one year in the ocean (mostly males and more hatchery than wild fish), most return after 2-3 years at sea.

Climate Effects on Abundance and Distribution

For Upper Columbia River spring-run Chinook, high overall sensitivity scores stemmed largely from migration characteristics. Multiple studies have examined the effect of climate change on water temperature and potential viability of populations in this DPS (Cristea and Burges 2010; Honea et al. 2016). High pre-spawn mortality is associated with adult holding in some lower tributaries. However, these lower-river holding areas appears to be utilized mainly by hatchery production fish. Thus, wild stocks do not appear directly threatened by high stream temperatures in holding tributaries.

Increasing fines in sediment due to increased winter flooding has been highlighted as a potential risk (Honea et al. 2016), although flooding was considered a lower risk for this Chinook DPS than for others considered in this assessment. Upper Columbia River spring-run Chinook ranked ***very high*** in sensitivity at the *adult freshwater stage* and ***very high*** in exposure to *hydrologic regime* shift. Most of this DPS inhabits streams with temperatures that are presently below optimal for growth, so short-term warming does not pose an imminent threat to juvenile survival. Nonetheless, Upper Columbia spring-run juveniles characteristically spend a full year in fresh water, and smolt survival depends on high spring flows. Therefore, this DPS ranked ***very high*** in sensitivity to climate change at the *juvenile freshwater stage*.

Long migrations contribute to climate risk for all upper Columbia River populations; however, the spawning and rearing habitat of this DPS is of relatively high quality compared to those of many other DPSs. Eggs incubate over winter, with relatively low risk of warming, and this was reflected in a ***low*** mean sensitivity attribute score. Flow regime in the Columbia Basin is strongly driven by snowmelt; therefore, loss of snowpack and subsequent reduction in the spring freshet will affect juvenile rearing and smolt migration. Although the spring smolt migration is slower in low snowpack years, earlier migration timing might benefit this DPS because at present, much of the population enters the ocean later than the optimal period for survival (Scheuerell et al. 2009). This was reflected in a ***moderate*** rank for sensitivity at the *marine stage*. The longer juvenile residence period translates to a higher risk of freshwater mortality but allows smolts to spend very little time in the estuary, lowering exposure to *sea level rise*. Thus, Upper Columbia River spring-run Chinook ranked ***low*** in sensitivity at the *estuary stage*.

The primary concern in terms of *cumulative life-cycle effects* was loss of the unique life histories of spring-run adults and yearling juvenile migrants. Upper Columbia

River spring-run Chinook is at less risk than Snake River spring/summer Chinook, largely because of its earlier run timing, which largely avoids high temperature stress during adult migration, prespawn holding, and juvenile rearing. *Cumulative life-cycle effects* was ranked ***moderate***.

Extrinsic Factors

Upper Columbia River spring-run Chinook is listed as threatened under the U.S. Endangered Species Act, with most populations at high risk of extinction due to low abundance and productivity. Spatial structure is also greatly depleted in this DPS because much of the original spawning habitat is blocked by impassable dams. Sensitivity to population viability was therefore ranked ***very high***. This DPS also ranked ***high*** in sensitivity to *hatchery influence* because it is heavily supplemented, and natural production is low. Its location in an agricultural region contributed to a ***high*** rank for *other stressors*, particularly water diversion and habitat loss.

Adaptive Capacity

Upper Columbia River spring-run Chinook may have sufficient adaptive capacity to shorten the juvenile freshwater residence period, but the consequences of such a shift for population viability are unknown. This DPS was deemed unlikely to shift upstream migration timing substantially, and its overall rank for adaptive capacity was ***moderate***.

Literature Cited

Cristea, N. C., and S. J. Burges. 2010. An assessment of the current and future thermal regimes of three streams located in the Wenatchee River basin, Washington State: some implications for regional river basin systems. Climatic Change **102**:493-520.

Crozier, L., E. Dorfmeier, T. Marsh, B. Sandford, and D. Widener. 2016. Refining our understanding of early and late migration of adult Upper Columbia spring and Snake River spring/summer Chinook salmon: passage timing, travel time, fallback and survival. Report of the National Marine Fisheries Service. Available from https://www.nwfsc.noaa.gov/publications/scipubs/searchdoc.cfm.

Fisher, J. P., L. A. Weitkamp, D. J. Teel, S. A. Hinton, J. A. Orsi, E. V. Farley, J. F. T. Morris, M. E. Thiess, R. M. Sweeting, and M. Trudel. 2014. Early ocean dispersal patterns of Columbia River Chinook and coho salmon. Trans. Am. Fish. Soc. **143**:252-272.

Healey, M. C. 1991. Life history of chinook salmon (*Oncorhynchus tshawytscha*). Page 564 *in* C. Groot and L. Margolis, editors. Pacific Salmon Life Histories. UCB Press, Vancouver.

Honea, J. M., M. M. McClure, J. C. Jorgensen, and M. D. Scheuerell. 2016. Assessing freshwater life-stage vulnerability of an endangered Chinook salmon population to climate change influences on stream habitat. Clim Res **71**:127-137.

Keefer, M. L., C. A. Peery, and C. C. Caudill. 2008. Migration timing of Columbia River spring Chinook salmon: effects of temperature, river discharge, and ocean environment. Trans. Am. Fish. Soc. **137**:1120-1133.

Myers, J. M., R. G. Kope, G. J. Bryant, D. Teel, L. J. Lierheimer, T. C. Wainwright, S. W. Grant, W. F. Waknitz, K. Neely, S. T. Lindley, and R. S. Waples. 1998. Status review of Chinook salmon from Washington, Idaho, Oregon, and California. U.S. Dep. Commerce NOAA Tech. Memo. NMFS-NWFSC-35.

Riddell, B. E., R. D. Brodeur, Alexander V. Bugaev , P. Moran, J. s. M. Murphy, J. h. A. Orsi, M. Trudel, L. A. Weitkamp, B. K. Wells, and A. C. Wertheimer. 2018. The ocean ecology of Chinook salmon Pages 555-696 *in* R. J. Beamish, editor. The ocean ecology of Pacific salmon and trout. American Fisheries Society, Bethesda.

Scheuerell, M. D., R. W. Zabel, and B. P. Sandford. 2009. Relating juvenile migration timing and survival to adulthood in two species of threatened Pacific salmon (*Oncorhynchus* spp.). J. Appl. Ecol. **46**:983–990.

Teel, D. J., D. L. Bottom, S. A. Hinton, D. R. Kuligowski, G. T. McCabe, R. McNatt, G. C. Roegner, L. A. Stamatiou, and C. A. Simenstad. 2014. Genetic identification of Chinook salmon in the Columbia River Estuary: Stock-specific distributions of juveniles in shallow tidal freshwater habitats. N. Am. J. Fish. Manage. **34**:621-641.

Teel, D. J., B. J. Burke, D. R. Kuligowski, C. A. Morgan, and D. M. Van Doornik. 2015. Genetic identification of Chinook salmon: Stock-specific distributions of juveniles along the Washington and Oregon Coasts. Mar. Coast. Fish **7**:274-300.

Weitkamp, L. A., D. J. Teel, M. Liermann, S. A. Hinton, D. M. Van Doornik, and P. J. Bentley. 2015. Stock-specific size and timing at ocean entry of Columbia River juvenile Chinook salmon and steelhead: Implications for early ocean growth. Mar. Coast. Fish **7**:370-392.

Lower Columbia River Chinook

Overall vulnerability—Moderate (1% Low, 87% Moderate, 12% High)
Biological sensitivity—Moderate (1% Low, 94% Moderate, 5% High)
Climate exposure—High (93% High, 7% Very high)
Adaptive capacity—High (2.4)
Data quality—74% of scores ≥ 2

	Lower Columbia River Chinook	Expert Scores	Data Quality	Expert Scores Plots (Portion by Category)
Sensitivity attributes	Early life history	1.3	3	
	Juvenile freshwater stage	1.5	3	
	Estuary stage	2.2	2.3	
	Marine stage	2.8	2.5	
	Adult freshwater stage	1.6	3	
	Cumulative life-cycle effects	1.3	1.8	
	Hatchery influence	3.3	2.5	
	Other stressors	2.4	2.3	
	Population viability	2.5	2.5	
	Ocean acidification sensitivity	1.9	1.8	
	Sensitivity Score	Moderate		
Exposure variables	Stream temperature	3.4	2.3	
	Summer water deficit	2.3	1.8	
	Flooding	2	2	
	Hydrologic regime	2.4	2.5	
	Sea level rise	2.1	2.3	
	Sea surface temperature	3.4	2	
	Ocean acidification exposure	3.8	2.5	
	Upwelling	2.3	1.8	
	Ocean currents	2	1.3	
	Exposure Score	High		
	Overall Vulnerability Rank	Moderate		

Low
Moderate
High
Very High

Life History Synopsis

Lower Columbia River Chinook exhibit two major life history types (Myers et al. 2006): a stream-maturing spring-run and an ocean-maturing fall-run. Each type represents a coordinated suite of juvenile and adult life-history traits. For example, spring-run adults enter fresh water in spring with a rising thermograph; these fish historically return to the upper reaches of larger watersheds, which are not readily accessible except during high flow (snowmelt) periods. Spring-run Chinook hold in these headwater areas through summer and into early fall, when they spawn.

Alternatively, fall-run adults return to natal streams with a falling thermograph, entering freshwater in September and October at the onset of seasonal rains. At freshwater entry, adults are at an advanced stage of maturity. Fall-run fish tend to spawn in the lower reaches of most streams, although in some rivers they may migrate a considerable distance (e.g. Cowlitz River). A variant of the fall-run is the late-fall run; these fish enter the Columbia River at an early stage of maturity, retaining much of their ocean silvering, and for this reason are called upriver brights. Their spawning begins in November and extends into the winter months (Marshall et al. 1995).

In general, juvenile Chinook can move downstream as fry in late winter, but may migrate throughout the year and into their second spring, depending on incubation and rearing conditions. In headwater areas, where spring-run Chinook spawn, water temperatures are colder, incubation and growth are slower, and juveniles may migrate in autumn as subyearlings or in the subsequent spring as yearlings. Both fall and late-fall juveniles migrate to the ocean primarily during their first year, with relatively few yearling migrants observed (Rich 1920; Groot and Margolis 1991; Healey 1991). Subyearling migrants from this DPS make extensive use of estuary habitats, while yearlings are less estuarine dependent (Fresh et al. 2005; Teel et al. 2014).

After ocean entry, subyearlings will spend some time in the Columbia River plume before migrating northward, commonly to coastal areas off British Columbia, but at times as far as the southeast Alaskan panhandle (Fisher et al. 2014; Teel et al. 2015). Yearling migrants, predominantly spring-run fish, move more rapidly through the estuary, but are ultimately captured in the same coastal fisheries as subyearlings.

Both life history types have evolved freshwater traits that respond to changes in temperature and precipitation, and are therefore susceptible to climate effects (Brannon et al. 2004). These fish will spend 1-5 years in the ocean, maturing predominantly at age 4 or 4, although early maturing (age-2) jacks are common (Myers et al. 2006). While spring and fall-run Chinook have distinct life history traits, both exhibit considerable plasticity in trait expression.

Climate Effects on Abundance and Distribution

Lower Columbia River Chinook had a ***high*** exposure score for summer *stream temperature*. If spring-run adults or yearling juveniles are restricted to lower river reaches due to lower flows, summer temperatures might become limiting. This DPS scored ***moderate*** for *hydrologic regime* shift, indicating that reduced snowmelt and higher winter flows may affect these fish in some areas. To access headwater areas, spring-run Chinook rely upon high flows from snowmelt during April-June; thus a reduced spring freshet might require earlier migration.

Timing of river entry for the spring run is triggered by a rising thermograph (Keefer et al. 2008). If spring temperatures are higher and spring flows lower, adults may

move into headwater reaches sooner than normal. It is conceivable that their energy stores might be insufficient to sustain them from summer to the early fall spawning period, when temperatures decline. Higher-resolution study of specific habitats is needed to clarify the extent of this risk.

Fall-run adults return to fresh water at an advanced state of maturation during September-October. For these fish, river entry is triggered in part by a falling thermograph, so warmer temperatures may delay arrival at spawning grounds or require fish to hold and spawn in waters at lethal or sublethal temperatures, resulting in direct or indirect mortality (Schreck et al. 2013; Keefer et al. 2018). There is some indication that holding in sublethal temperatures can degrade the quality of both male and female gametes (McCullough et al. 2001; Lahnsteiner and Kletzl 2012). Late-fall adults from this DPS may be less subject to deleterious temperatures given the November timing of their freshwater entry. Timing of maturation and spawning strongly influences the susceptibility of different run types to climate change.

As for nearly all Chinook DPSs, warmer winter temperatures will likely accelerate embryonic development and emergence timing. Delayed spawning might reduce temperature effects on emergence timing. However, warmer developmental temperatures can still lead to degraded condition in alevins (Fuhrman et al. 2018), which may have less yolk to tide them over until external food sources are available.

At present, we lack sufficient information on how stream productivity changes with warming temperature to determine whether bioenergetic constraints will be detrimental to salmon. Nevertheless, downstream migration is triggered by flow and facilitated by snowmelt in spring. Whether directly or indirectly, Lower Columbia River Chinook salmon juveniles will be affected by warmer stream temperatures, as well as by changing estuary and coastal ocean conditions (Daly and Brodeur 2015).

Extrinsic Factors

Lower Columbia River Chinook ranked ***high*** in sensitivity to the role of hatcheries, with diversity affected by major hatchery programs. This DPS is listed as threatened under the U.S. Endangered Species Act, and was scored ***moderate*** for population viability. Spatial structure is compromised in this DPS because major dams limit or exclude access to historically important spawning habitat. Development in lowland areas and diking has also diminished freshwater and estuary habitats. Adaptive Capacity

Lower Columbia River Chinook ranked ***high*** in adaptive capacity overall, largely because of high diversity in both juvenile and adult run timing across the DPS as a whole. This rank does not imply that specific populations might not be at higher risk, or that diversity within the DPS will not diminish in the future.

Literature Cited

Brannon, E. L., M. S. Powell, T. P. Quinn, and A. Talbot. 2004. Population structure of Columbia River Basin Chinook salmon and steelhead trout. Rev. Fish. Sci. 12:99-232.

Daly, E. A., and R. D. Brodeur. 2015. Warming ocean conditions relate to increased trophic requirement of threatened and endangered salmon. PLoS ONE 10:e0144066.

Fisher, J. P., L. A. Weitkamp, D. J. Teel, S. A. Hinton, J. A. Orsi, E. V. Farley, J. F. T. Morris, M. E. Thiess, R. M. Sweeting, and M. Trudel. 2014. Early ocean dispersal patterns of Columbia River Chinook and coho salmon. Trans. Am. Fish. Soc. 143:252-272.

Fresh, K. L., L. L. Casillas, L. L. Johnson, and D. L. Bottom. 2005. Role of the estuary in the recovery of Columbia River basin salmon and steelhead: an evaluation of the effects of selected factors on salmonid population viability. U.S. Dep. Commerce NOAA Tech. Memo. NMFS-NWFSC-69.

Fuhrman, A. E., D. A. Larsen, E. A. Steel, G. Young, and B. R. Beckman. 2018. Chinook salmon emergence phenotypes: Describing the relationships between temperature, emergence timing and condition factor in a reaction norm framework. Ecol. Freshw. Fish. 27:350-362.

Groot, C., and L. Margolis. 1991. Pacific Salmon Life Histories. Columbia Press, Vancouver, B.C., Canada.

Healey, M. C. 1991. Life history of chinook salmon (*Oncorhynchus tshawytscha*). Page 564 *in* C. Groot and L. Margolis, editors. Pacific Salmon Life Histories. UCB Press, Vancouver, B.C., Canada.

Keefer, M. L., T. S. Clabough, M. A. Jepson, E. L. Johnson, C. A. Peery, and C. C. Caudill. 2018. Thermal exposure of adult Chinook salmon and steelhead: Diverse behavioral strategies in a large and warming river system. PLoS ONE 13:e0204274.

Keefer, M. L., C. A. Peery, and C. C. Caudill. 2008. Migration timing of Columbia River spring Chinook salmon: effects of temperature, river discharge, and ocean environment. Trans. Am. Fish. Soc. 137:1120-1133.

Lahnsteiner, F., and M. Kletzl. 2012. The effect of water temperature on gamete maturation and gamete quality in the European grayling (*Thymalus thymallus*) based on experimental data and on data from wild populations. Fish. Physiol. 38:455-467.

Marshall, A. R., C. Smith, R. Brix, W. Dammers, J. Hymer, and L. Lavoy. 1995. Genetic diversity units and major ancestral lineages for Chinook salmon in Washington. WA Dep. Fish Wildl. Tech. Rep. 95 02.

McCullough, D., S. Spalding, D. Sturdevant, and M. hicks. 2001. Issue Paper 5. Summary of technical literature examining the physiological effects of temperature. Region 10 temperture water quality criteria guidance development project. U.S. Environmental Protection Agency doc 910 D 01 005.

Myers, J., C. Busack, D. Rawding, A. Marshall, D. J. Teel, D. M. Van Doornik, and M. Maher. 2006. Historical population structure of Pacific salmonids in the Willamette River and Lower Columbia River Basins. U.S. Dep. Commerce NOAA Tech. Memo. NMFS-NWFSC-73.

Rich, W. H. 1920. Early life history and seaward migration of Chinook salmon in the Columbia and Sacramento Rivers. Fish. Bull. U.S. 37(1).

Schreck, C. B., M. L. Jent, M. E. Colvin, S. Benda, C. Sharpe, J. T. Peterson, and B. Dolan. 2013. Potential causes and management of prespawn mortality in adult Upper Willamette River spring Chinook. Report to the U.S. Army Corps of Engineers, Portland, OR.

Teel, D. J., D. L. Bottom, S. A. Hinton, D. R. Kuligowski, G. T. McCabe, R. McNatt, G. C. Roegner, L. A. Stamatiou, and C. A. Simenstad. 2014. Genetic identification of Chinook salmon in the Columbia River Estuary: Stock-specific distributions of juveniles in shallow tidal freshwater habitats. N. Am. J. Fish. Manage. 34:621-641.

Teel, D. J., B. J. Burke, D. R. Kuligowski, C. A. Morgan, and D. M. Van Doornik. 2015. Genetic identification of Chinook salmon: Stock-specific distributions of juveniles along the Washington and Oregon Coasts. Mar. Coast. Fish 7:274-300.

Puget Sound Chinook

Overall vulnerability—High (96% High, 4% Very high)
Biological sensitivity—High (100% High)
Climate exposure—High (96% High, 4% Very high)
Adaptive capacity—High (2.3)
Data quality—79% of scores ≥ 2

	Puget Sound Chinook	Expert Scores	Data Quality	Expert Scores Plots (Portion by Category)
Sensitivity attributes	Early life history	3.5	2.5	
	Juvenile freshwater stage	2.6	2	
	Estuary stage	2.7	2.5	
	Marine stage	2.8	2.5	
	Adult freshwater stage	2.9	3	
	Cumulative life-cycle effects	2.3	1.8	
	Hatchery influence	2.7	2.3	
	Other stressors	3.6	2.8	
	Population viability	2.9	3	
	Ocean acidification sensitivity	2	2.3	
	Sensitivity Score	High		
Exposure variables	Stream temperature	3.4	2.3	
	Summer water deficit	2.3	1.8	
	Flooding	2.5	2	
	Hydrologic regime	3.4	2.5	
	Sea level rise	2.2	2.3	
	Sea surface temperature	3.1	2	
	Ocean acidification exposure	3.8	2.5	
	Upwelling	2.3	1.8	
	Ocean currents	2	1.3	
	Exposure Score	High		
Overall Vulnerability Rank		High		

Low
Moderate
High
Very High

Life History Synopsis

Puget Sound Chinook salmon is notable for its large adult size and great life history variation. Adults migrate into Puget Sound rivers in two to three seasonal runs. The spring run returns during May-July with snowmelt from the Cascade and Olympic Mountains and spawns primarily in higher-elevation tributaries. The summer/fall-run returns from July to October during low-flow periods and spawns in lower-elevation mainstem and large tributary reaches. There are 19 Chinook spawning populations in Puget Sound, with population group spanning both the Olympic and Cascade watersheds and ensuring broad spatial diversity across the region for this DPS. Migration corridors in these watersheds are no greater than 100 miles from river mouth to spawning grounds, so upstream migration can be quite rapid. These migrations can be delayed by stream

temperatures, which vary dramatically across the Puget Sound region in part due to the presence or absence of headwater glaciers. Adults spawn in large gravel beds, depositing 2500-7500 eggs. Eggs hatch in 4-7 months, and length of incubation is temperature-dependent.

After emergence from redds, Puget Sound Chinook exhibits highly variable juvenile life history strategies. Most migrate to Puget Sound as subyearling fry that rear extensively in natal or nearby non-natal estuaries, coastal creek mouths, or other coastal habitats for up to 4 months (Healey 1991; Beamer et al. 2005). Accordingly, Puget Sound Chinook salmon may be regarded as highly dependent upon estuary environments during the early life history. Nevertheless, other subyearlings develop as parr that rear an equivalent time in riverine habitats before migrating. A smaller proportion of individuals migrate as yearlings, but this life history type has decreased in recent years (Beechie et al. 2006).

After migration into Puget Sound in spring or summer, individuals may exhibit extended residence within the Salish Sea before migrating to the Pacific Ocean, and some individuals (termed blackmouth) remain for the rest of their lives as residents within the Salish Sea. Ocean-going individuals tend to remain near the continental shelf, residing from coastal British Columbia to southeast Alaska (Myers et al. 1998). Most Puget Sound Chinook adults return to spawn after 3-6 years in marine waters (the dominant year class is age 4), although a minority may return as age-2 jacks.

Climate Effects on Abundance and Distribution

Puget Sound Chinook salmon is subject to a wide variety of climate impacts. The greatest risks likely occur during incubation, when eggs are vulnerable to high mortality due to increased flooding and variability in seasonal flow (Ward et al. 2015). This was reflected in a ***high*** sensitivity score for *early life history* (egg incubation). A ***high*** exposure score for *hydrologic regime* resulted from the expectation from our analysis that 8% of spawning habitat will change from snow-dominated to transitional, and 16% will change from transitional to rain-dominated (Appendix S2). These projections suggest that winter flooding will become more common, directly affecting incubating eggs. *Stream temperature* was ranked ***high*** in the extent of change expected, which could increase pre-spawn mortality in low-elevation tributaries (Cristea and Burges 2010).

Rising temperatures during late spring and summer may also impact Chinook juveniles in estuary and riverine habitats. Most Puget Sound estuaries already surpass optimal rearing temperatures in summer, and the expectation of additional habitat warming for this DPS was reflected in its ***high*** exposure score to *sea surface temperature*. Thus Puget Sound Chinook is likely to face increased vulnerability, despite its ***moderate*** score for *estuary stage*. Estuary rearing habitat in Puget Sound has already been greatly impaired due to agriculture and urbanization and may be further degraded as a consequence of *sea level rise*. Nevertheless, this DPS ranked ***moderate*** in exposure to

sea level rise because Puget Sound is generally expected to experience less sea level rise than recovery domains to the south.

Chinook salmon is a notable predator of crab larvae, including Dungeness crab, which is thought to be sensitive to direct (Busch and McElhany 2016) as well as indirect effects of *ocean acidification* on the benthic food web (Marshall et al. 2017; Hodgson et al. 2018). However, such food web effects remain highly uncertain (Busch et al. 2013). Chinook salmon appears sensitive to a variety of ocean conditions, so *upwelling* and *ocean currents* have potential to impact abundance of adult returns. Compared to other DPSs, Puget Sound Chinook will likely have lower exposure to adverse temperature impacts during upstream migration, holding, and spawning, although *stream temperature* effects are more likely for summer/fall-run adult migrants.

Extrinsic Factors

Hatcheries play a large role in Puget Sound, with migrants through the sound surpassing 70% hatchery origin in some areas (Rice et al. 2011). Rates of disease prevalence and intensity are correlated with local abundance of hatchery fish (Rhodes et al. 2011). Many hatcheries within the Puget Sound recovery domain are production hatcheries. Genetic diversity of the Puget Sound Chinook DPS has been greatly impacted by fish from the Green River, which has historically dominated the broodstock of all regional hatcheries (Hard et al. 2015; Myers et al. 2015). Nonetheless, this DPS scored ***moderate*** in vulnerability to *hatchery influence*, reflecting the perception by scorers that other DPSs experience relatively higher impacts from hatcheries.

Chinook salmon life-history diversity has likely been reduced by hydropower facilities that block high-elevation spawning areas, thereby reducing abundance of the yearling life history type (Beechie et al. 2006). However, all Chinook life history types have been impacted by losses of mainstem, floodplain, and estuary rearing habitats as a consequence of forestry, agriculture, and urbanization (Simenstad et al. 2011). These activities have also exposed spawning populations to contaminants correlated with lower survival (Meador 2014). These impacts combined to produce a ***very high*** score for *other stressors*.

Puget Sound Chinook is listed as threatened under the U.S. Endangered Species Act, and *population viability* was ranked as ***moderate***. Many populations show a continued decline in adult returns (Ford et al. 2015). Marine survival is highly variable among populations within Puget Sound (Ruff et al. 2017).

Adaptive Capacity

Puget Sound Chinook was ranked ***high*** in adaptive capacity because of its high expression of life history variation. This DPS is expected to be somewhat resilient to temperature extremes and to high mortality events associated with changes in flow. Subyearlings exhibit great flexibility in residence among freshwater, estuarine, and

marine habitats. As a consequence of change in both temperature and rearing habitat capacity, relative habitat usage was considered likely to change based on local conditions and growth opportunities. Furthermore, some large rivers within Puget Sound are expected to increase in growth opportunity for salmon as a consequence of warming (Beer and Anderson 2011); thus, not all climate change effects need result in negative impacts to salmon populations.

Literature Cited

Beamer, E., A. M. McBride, C. Greene, R. Henderson, W. G. Hood, K. Wolf, K. Larsen, C. A. Rice, and K. Fresh. 2005. Delta and nearshore restoration for the recovery of wild Skagit River Chinook salmon: Linking estuary restoration to wild Chinook salmon populations. Skagit River System Cooperative, LaConner, WA. Available from http://skagitcoop.org/wp-content/uploads/Appendix-D-Estuary1.pdf.

Beechie, T., E. Buhle, M. Ruckelshaus, A. Fullerton, and L. Holsinger. 2006. Hydrologic regime and the conservation of salmon life history diversity. Biol. Conserv. 130:560-572.

Beer, W. N. and J. J. Anderson. 2011. Sensitivity of juvenile salmonid growth to future climate trends. River. Res. Appl. 27:663-669.

Busch, D. S., C. J. Harvey, and P. McElhany. 2013. Potential impacts of ocean acidification on the Puget Sound food web. ICES J. Mar. Sci. 70:823-833.

Busch, D. S. and P. McElhany. 2016. Estimates of the direct effect of seawater pH on the survival rate of species groups in the California Current ecosystem. PLoS ONE 11:e0160669.

Cristea, N. C. and S. J. Burges. 2010. An assessment of the current and future thermal regimes of three streams located in the Wenatchee River basin, Washington State: some implications for regional river basin systems. Climatic Change 102:493-520.

Ford, M. J., K. Barnas, T. Cooney, L. G. Crozier, M. Diaz, J. J. Hard, E. E. Holmes, D. M. Holzer, R. G. Kope, P. W. Lawson, M. Liermann, J. M. Myers, M. Rowse, D. J. Teel, D. M. V. Doornik, T. C. Wainwright, L. A. Weitkamp, and M. Williams. 2015. Status review update for Pacific salmon and steelhead listed under the Endangered Species Act: Pacific Northwest. U.S. Dep. Commerce, Seattle, WA. Available from www.nwfsc.noaa.gov/publications/scipubs/searchdoc.cfm.

Hard, J. J., J. M. Myers, E. J. Conner, R. A. Hayman, R. G. Kope, G. Lucchetti, A. R. Marshall, G. R. Pess, and B. E. Thomspson. 2015. Viability criteria for steelhead within the Puget Sound distinct population segment. U.S. Dep. Commerce NOAA Tech. Memo. NMFS-NWFSC-129.

Healey, M. C. 1991. Life history of chinook salmon (*Oncorhynchus tshawytscha*). Page 564 *in* C. Groot and L. Margolis, editors. Pacific Salmon Life Histories. UCB Press, Vancouver, B.C., Canada.

Hodgson, E. E., I. C. Kaplan, K. N. Marshall, J. Leonard, T. E. Essington, D. S. Busch, E. A. Fulton, C. J. Harvey, A. Hermann, and P. McElhany. 2018. Consequences of spatially variable ocean acidification in the California Current: Lower pH drives strongest declines in benthic species in southern regions while greatest economic impacts occur in northern regions. Ecol. Model. 383:106-117.

Marshall, K. N., I. Kaplan, E. E. Hodgson, A. Hermann, D. Shallin Busch, P. McElhany, T. Essington, C. J. Harvey, and E. A. Fulton. 2017. Risks of ocean acidification in the California Current food web and fisheries: Ecosystem model projections. Global Change Biol. 23:1525-1539.

Meador, J. P. 2014. Do chemically contaminated river estuaries in Puget Sound (Washington, USA) affect the survival rate of hatchery-reared Chinook salmon? Can. J. Fish. Aquat. Sci. 71:162-180.

Myers, J. M., J. J. Hard, E. J. Connor, R. A. Hayman, R. G. Kope, A. Luchetti, A. R. Marshall, G. Pess, and B. E. Thompson. 2015. Identifying historical populations of steelhead within the Puget Sound distinct population segment. U.S. Dep. Commerce NOAA Tech. Memo. NMFS-NWFSC-128.

Myers, J. M., R. G. Kope, G. J. Bryant, D. Teel, L. J. Lierheimer, T. C. Wainwright, S. W. Grant, W. F. Waknitz, K. Neely, S. T. Lindley, and R. S. Waples. 1998. Status review of Chinook salmon from Washington, Idaho, Oregon, and California. U.S. Dep. Commerce NOAA Tech. Memo. NMFS-NWFSC-35.

Rhodes, L. D., C. A. Rice, C. M. Greene, D. J. Teel, S. L. Nance, P. Moran, C. A. Durkin, and S. B. Gezhegne. 2011. Nearshore ecosystem predictors of a bacterial infection in juvenile Chinook salmon. Mar. Ecol. Prog. Ser. 432:161-172.

Rice, C. A., C. M. Greene, P. Moran, D. J. Teel, D. R. Kuligowski, R. R. Reisenbichler, E. M. Beamer, J. R. Karr, and K. L. Fresh. 2011. Abundance, stock origin, and length of marked and unmarked juvenile Chinook salmon in the surface waters of greater Puget Sound. Trans. Am. Fish. Soc. 140:170-189.

Ruff, C. P., J. H. Anderson, I. M. Kemp, N. W. Kendall, P. A. McHugh, A. Velez-Espino, C. M. Greene, M. Trudel, C. A. Holt, K. E. Ryding, and K. Rawson. 2017. Salish Sea Chinook salmon exhibit weaker coherence in early marine survival trends than coastal populations. Fish. Oceanogr. 26:625-637.

Simenstad, C. A., M. Ramirez, J. Burke, M. Logsdon, H. Shipman, C. Tanner, J. Toft, B. Craig, C. Davis, J. Fung, P. Bloch, K. Fresh, S. Campbell, D. Myers, E. Iverson, B. A., P. Schlenger, C. Kiblinger, P. Myre, W. I. Gertsel, and A. MacLennan. 2011. Historical change and impairment of Puget Sound shorelines: Atlas and interpretation of Puget Sound nearshore ecosystem restoration project change analysis. Puget Sound Nearshore Ecosystem Restoration Project Rep 2011-01; 2011. Available from http://www.pugetsoundnearshore.org/technical_papers/change_analysis.pdf.

Ward, E. J., J. H. Anderson, T. J. Beechie, G. R. Pess, and M. J. Ford. 2015. Increasing hydrologic variability threatens depleted anadromous fish populations. Global Change Biol. 21:2500-2509.

Coho Salmon (*Oncorhynchus kisutch*)

Central California Coast coho

Overall vulnerability—Very high (100% Very high)
Biological sensitivity—Very high (100% Very high)
Climate exposure—High (86% High, 14% Very high)
Adaptive capacity—Low (1.3)
Data quality—79% of scores ≥ 2

	Central California Coast Coho	Expert Scores	Data Quality	Expert Scores Plots (Portion by Category)
Sensitivity attributes	Early life history	3	1.5	
	Juvenile freshwater stage	3.8	2.8	
	Estuary stage	3.2	1.5	
	Marine stage	3.4	2.5	
	Adult freshwater stage	1.9	2.8	
	Cumulative life-cycle effects	3.6	1.5	
	Hatchery influence	2.1	2.8	
	Other stressors	3	2	
	Population viability	3.7	3	
	Ocean acidification sensitivity	1.8	1.8	
	Sensitivity Score	Very High		
Exposure variables	Stream temperature	3.2	2	
	Summer water deficit	2.4	1.8	
	Flooding	3.7	1.3	
	Hydrologic regime	1.1	2.5	
	Sea level rise	3.3	2	
	Sea surface temperature	3.4	2	
	Ocean acidification exposure	4	2.5	
	Upwelling	2.9	1	
	Ocean currents	1.8	1	
	Exposure Score	High		
Overall Vulnerability Rank		Very High		

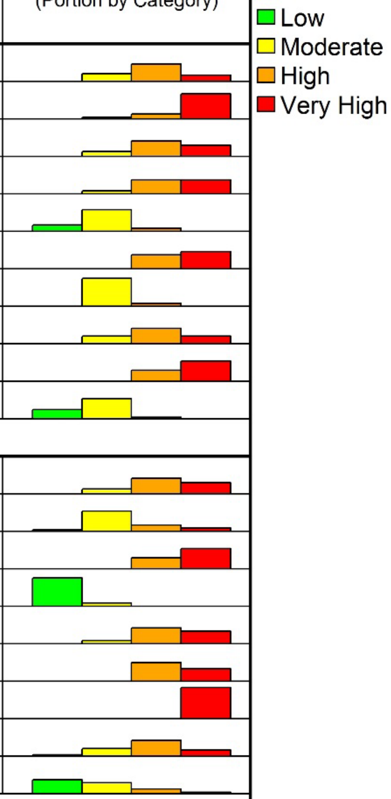

Life History Synopsis

Central California Coast coho adults typically enter rivers from November through mid February (Shapovalov and Taft 1954; Weitkamp et al. 1995). Because many central California estuaries are seasonally closed by sandbars, migration timing can depend on bar-breaching hydrodynamics. Freshwater migration distances range from a few to 50 km or more in larger watersheds such as the Russian River. Egg incubation generally takes place between December and late April or early May (Shapovalov and Taft 1954; Weitkamp et al. 1995).

Juveniles exhibit a range of behaviors, life histories, and habitat associations, with the majority residing about one year in fresh water before migrating to sea. In estuaries seasonally closed due to sandbar dynamics, some age-0 juveniles will migrate only as far as the ecotone between the stream and estuary. These juveniles reside in estuaries for variable periods depending on temperature, dissolved oxygen, and salinity levels, but they subsequently move back upstream without entering seawater (Miller and Sadro 2003; Koski 2009, Jones et al. 2014; Wallace et al. 2015). A small-to-modest fraction may spend a second year in fresh water before migrating to sea (Bell 2001; Gallagher et al. 2014). Downstream migrants typically enter the ocean between April and May (Spence and Hall 2010).

Marine movements of Central California Coast coho are unclear, but tag recovery data from hatchery populations to the north suggest that these fish inhabit marine regions fairly close to their natal rivers (Pearcy and Fisher 1988; Weitkamp and Neely 2002; Quinn and Myers 2005). The majority of adults return to spawn at age 3, though age-2 jacks can comprise a significant proportion of the run (Shapovalov and Taft 1954; Weitkamp et al. 1995). Fish residing for 2 years in fresh water appear to return for spawning at age 3 or 4 (E. Ettlinger, Marin Municipal Water District, pers. comm.).

Climate Effects on Abundance and Distribution

The Central California Coast coho DPS comprises the southern distributional limit of its species, and thus already faces numerous limiting factors stemming from climate effects. No formal studies were available on climate impacts to Central California Coast coho, but the vulnerability assessment suggested four important threats:

1) Estuary breaching dynamics and water-quality are important for coho ecology; thus coho populations are likely sensitive to rising sea-level, rising air temperatures, and changes in stream flow. To reflect this threat, sensitivity in the *estuary stage* was ranked ***high*** for Central California Coast coho, as was exposure to *sea level rise* and *stream temperature*. Based on these scores, the *estuary stage* was considered a ***highly vulnerable life stage*** for this DPS, and its exposure to *flooding* was also ranked ***very high***. It is important to note that flooding can have positive effects on bar-breaching, despite negative effects on eggs in the gravel.

   During dry periods, California estuaries naturally develop sand bars that close the estuary, block fish passage, and alter hydrological functioning. In many streams, recent drought has delayed and sometimes prevented the winter breaching of sand bars that allows migrating adults to enter fresh water. Some juveniles use estuaries during the closed phase, but these fish are impacted, and can even be killed, by poor water quality in closed estuaries, primarily due to high temperatures, low levels of dissolved oxygen, and high salinity (Smith 2009). In closed estuaries, water quality depends on complex interactions between stream flow, over-bar exchange of fresh and marine waters, breaching dynamics of sand bars, wind, and other factors. Changes in sea-level, air temperature, and the amount and timing of precipitation are likely to alter these interactions in ways that further impact coho.

2) For coho eggs and juveniles, the interaction between hydrologic change and warming will likely have sub-lethal effects that propagate through the life cycle. Warmer water temperatures accelerate development and hatching of eggs (Murray and McPhail 1988), as well as affecting metabolism, growth, and development of juveniles (Spence 1995). Streambed scour may increase due to more extreme winter flows, which may in turn increase the deposition of fine sediments, thereby increasing egg mortality (Shapovalov and Taft 1954; Stillwater Sciences and EA Engineering 1997). Deposition of fine sediments may also obstruct the emergence of alevins, although this relationship may not be entirely predictable (Ward et al. 2015). Sensitivity in the *early life history* stage was ranked ***moderate***.

3) Fog dynamics in summer are associated with coastal upwelling in the nearshore ocean and can have a significant influence on maximum temperature and evaporation in local streams. Central California Coast coho ranked ***moderate*** in exposure to *upwelling* but ***high*** in exposure to *sea surface temperature* and *stream temperature*. For juveniles, summer and fall are high-stress periods characterized by low flows, diminished food production, and high metabolic demands caused by warm temperatures. Sensitivity in the *juvenile freshwater stage* was thus ranked ***very high*** for this DPS. Changes in the fog regime would further directly affect the juvenile freshwater stage, which is already a stressful period. However, the future fog regime is highly uncertain: fog may intensify, diminish, or change in seasonality or year-to-year consistency (Johnstone and Dawson 2010).

4) Depending on the balance of these factors in the adult, early life history and juvenile freshwater stages, fish may or may not transition to the smolt stage during the historical juvenile migration period. A shift in smolt timing in turn may have fitness consequences due to a potential mismatch between ocean entry timing and favorable ocean conditions or due to closure of migration corridors by sandbar formation (Osterback et al. 2018). Owing to the combination of ***very high*** scores for *adult* and *juvenile freshwater stages* and the potential for lost access between freshwater and ocean habitats, this DPS ranked ***very high*** in *cumulative life-cycle effects*.

Broadly, marine survival of coho populations across Oregon and California appears sensitive to specific aspects the Pacific Decadal Oscillation, such as timing of the spring upwelling transition, strength of upwelling in spring, and sea-surface temperatures, although relationships are complex (Lawson 1997; Mantua et al. 1997; Ryding and Skalski 1999; Beamish et al. 2000; Koslow et al. 2002; Logerwell et al. 2003). Movement of coho salmon in response to ocean regime is not well understood, but the first few months at sea appear to be critical to marine survival (Pearcy 1992). Thus, overall sensitivity in the *marine stage* was ranked ***moderate***.

Extrinsic Factors

The Central California Coast coho DPS is listed as endangered under the U.S. Endangered Species Act and ranked ***very high*** in sensitivity to *population viability*. In the most recent viability assessment, 8 of 12 independent populations were extant in this DPS, but none were considered at low risk of extinction (Spence 2016). Assessing the

viability of Central California Coast coho DPS is challenging due to the scarcity of long-term datasets for most populations. Available data indicated that all independent and dependent populations were well below recovery targets, and in some cases, exceeded high-risk thresholds. An area of particular concern was the downward trend in abundance of virtually all dependent populations throughout the DPS. Downward trends in dependent populations may indicate that they are less able to maintain connectivity or act as buffers against declines in neighboring independent populations. Independent populations might therefore become more isolated with time (Spence 2016).

Stressors that limit viability in this DPS include pollution and poor water quality, especially in areas of heavy agricultural and urban development (NMFS 2012), as well as modest bycatch in Chinook and coho fisheries off California and Oregon (Williams et al. 2016). Many stream and estuarine habitats used by this DPS have been significantly reduced in extent, quality, or complexity due to flood-plain alteration, lack of large woody debris, water diversions, and other stressors. These historic habitat alterations will likely reduce the resilience of coho to climate change, but an ongoing, long-term effort to improve habitat conditions could aid resilience over time. For these reasons, this DPS ranked ***high*** in sensitivity to *other stressors*.

For Central California Coast coho populations, persistence is supported by two conservation hatcheries involving captive rearing of adults. Conservation hatcheries scored lower in this risk assessment than production hatcheries, so overall sensitivity to *hatchery influence* was ranked ***low*** for this DPS. However, hatchery risks include loss of genetic diversity (Satterthwaite et al. 2015) and possible inbreeding depression, in addition to fungal infection at the southernmost hatchery. Marine survival of conservation hatchery fish has generally been poor compared to that of fish from other hatcheries.

Adaptive Capacity

Central California Coast coho ranked ***low*** for *adaptive capacity*. This DPS comprises the southern distributional limit of the species, apparently having maximized its ability to modify its life history.

Literature Cited

Beamish, R. J., D. J. Noakes, G. A. McFarlane, W. Pinnix, R. Sweeting, and J. King. 2000. Trends in coho marine survival in relation to the regime concept. Fish. Oceanogr. 9:114-119.

Bell, E. 2001. Survival, growth and movement of juvenile coho salmon (*Oncorhynchus kisutch*) over wintering in alcoves, backwaters, and main channel pools in Prairie Creek, California. M.S. Thesis, Humboldt State University, Arcata, CA.

Gallagher, S. P., S. A. Thompson, and D. W. Wright. 2014. Coastal Mendocino County salmonid life cycle and regional monitoring: monitoring status and trends 2013. Calif. Dep. Fish Wildl., Fort Bragg.

Johnstone, J. A. and T. E. Dawson. 2010. Climatic context and ecological implications of summer fog decline in the coast redwood region. Proc. Nat. Acad. Sci. 107:4533-4538.

Jones, K. K., T. J. Cornwell, D. L. Bottom, L. A. Campbell, and S. Stein. 2014. The contribution of estuary-resident life histories to the return of adult *Oncorhynchus kisutch*. J. Fish. Biol. 85:52-80.

Koski, K. 2009. The fate of coho salmon nomads: the story of estuarine rearing strategy promoting resilience. Ecol. Soc. 14:4.

Koslow, J. A., A. J. Hobday, and G. W. Boehlert. 2002. Climate variability and marine survival of coho salmon (*Oncorhynchus kisutch*) in the Oregon production area. Fish. Oceanogr. 11:65-77.

Lawson, P. W. 1997. Interannual variability in growth and survival of Chinook and coho salmon. Oregon Dep. Fish Wildl., Newport.

Logerwell, E. A., N. Mantua, P. W. Lawson, R. C. Francis, and V. N. Agostini. 2003. Tracking environmental processes in the coastal zone for understanding and predicting Oregon coho (*Oncorhynchus kisutch*) marine survival. Fish. Oceanogr. 12:554-568.

Mantua, N. J., S. R. Hare, Y. Zhang, J. M. Wallace, and R. C. Francis. 1997. A Pacific interdecadal climate oscillation with impacts on salmon production. Bull. Am. Meteorol. Soc. 78:1069-1079.

Miller, B. A. and S. Sadro. 2003. Residence time and seasonal movements of juvenile coho salmon in the ecotone and lower estuary of Winchester Creek, South Slough, Oregon. Trans. Am. Fish. Soc. 132:546-559.

Murray, C. B..and J. D. McPhail. 1988. Effect of incubation temperature on the development of five species of Pacific salmon (*Oncorhynchus*) embryos and alevins. Can. J. Zool. 66:266-273.

NMFS (National Marine Fisheries Service). 2012. Final recovery plan for Central California Coast coho salmon evolutionarily significant Unit. U.S. Dep. Commerce, NMFS Southwest Region, Santa Rosa, CA.

Osterback, A.M. K., C. H. Kern, E. A. Kanawi, J. M. Perez, and J. D. Kiernan. 2018. The effects of early sandbar formation on the abundance and ecology of coho salmon (*Oncorhynchus kisutch*) and steelhead trout (*Oncorhynchus mykiss*) in a central California coastal lagoon. Can. J. Fish. Aquat. Sci. 75:2184-2197.

Pearcy, W. G. 1992. Ocean ecology of North Pacific salmonids. University of Washington Press, Seattle.

Pearcy, W. G. and J. P. Fisher. 1988. Migrations of coho salmon, *Oncorhynchus kisutch*, during their first summer in the ocean. Fish. Bull. U.S. 86:173-195.

Quinn, T. P. and K. W. Myers. 2005. Anadromy and the marine migrations of Pacific salmon and trout: Rounsefell revisited. Rev. Fish. Biol. Fish. 14:421-442.

Ryding, K. E. and J. R. Skalski. 1999. Multivariate regression relationships between ocean conditions and early marine survival of coho salmon (*Oncorhynchus kisutch*). Can. J. Fish. Aquat. Sci. 56:2374-2384.

Satterthwaite, W. H., S. M. Carlson, and I. Bradbury. 2015. Weakening portfolio effect strength in a hatchery-supplemented Chinook salmon population complex. Can. J. Fish. Aquat. Sci. 72:1860-1875.

Shapovalov, L. and A. C. Taft. 1954. The life histories of the steelhead rainbow trout (*Salmo gairdneri gairdneri*) and silver salmon (*Oncorhynchus kisutch*) with special reference to Waddell Creek, California and recommendations regarding their management. Calif. Dep. Fish Game Fish. Bull. 98:275 pp.

Smith, K. A. 2009. Inorganic chemical oxygen demand of re-suspended sediments in a bar bound lagoon. M.S. Thesis, San Jose University, San Jose, CA.

Spence, B. C. 1995. Spatiotemporal patterns in migration timing of coho salmon (*Oncorhynchus kisutch*) smolts in North America. Oregon State University, Corvallis.

Spence, B. C. 2016. North-Central California Coast Recovery Domain.*in* B. C. Williams, D. A. Spence, D. Boughton, R. C. Johnson, N. Crozier, M. Mantua, M. R. O'Farrell, and S. Lindley, editors. Viability assessment for Pacific salmon and steelhead listed under the Endangered Species Act: Southwest. U.S. Dep. Commerce NOAA Tech. Memo. NMFS-SWFSC-564.

Spence, B. C. and J. D. Hall. 2010. Spatiotemporal patterns in migration timing of coho salmon (*Oncorhynchus kisutch*) smolts in North America. Can. J. Fish. Aquat. Sci. 67:1316-1334.

Stillwater Sciences and EA Engineering. 1997. A review of coho salmon life history to assess potential limiting factors and the implications of historic removal of large woody debris in coastal Mendocino County. Preliminary draft report. Prepared by Stillwater Sciences, Berkeley, California and EA Engineering, Science, and Technology, Lafayette, California for Louisiana-Pacific Corporation, Trinidad, California.

Wallace, M., S. Ricker, A. Garwood, A. Frimodig, and S. Allen. 2015. Importance of the stream estuary ecotone to juvenile coho salmon (*Oncorhynchus kisutch*) in Humboldt Bay, California. Calif. Fish Game 101:241-266.

Ward, E. J., J. H. Anderson, T. J. Beechie, G. R. Pess, and M. J. Ford. 2015. Increasing hydrologic variability threatens depleted anadromous fish populations. Global Change Biol. 21:2500-2509.

Weitkamp, L. A. and K. Neely. 2002. Coho salmon (*Oncorhynchus kisutch*) ocean migration patterns: insight from marine coded wire tag recoveries. Can. J. Fish. Aquat. Sci. 59:1100-1115.

Weitkamp, L. A., T. C. Wainwright, G. J. Bryant, G. B. Milner, D. J. Teel, R. G. Kope, and R. S. Waples. 1995. Status review of coho salmon from Washington, Oregon, and California. U.S. Dep. Commerce NOAA Tech. Memo. NMFS-NWFSC-24.

Williams, T. H., B. C. Spence, D. A. Boughton, R. C. Johnson, L. G. Crozier, N. J. Mantua, M. R. O'Farrell, and S. T. Lindley. 2016. Viability assessment for Pacific salmon and steelhead listed under the Endangered Species Act: Southwest U.S. Dep. Commerce NOAA Tech. Memo. NMFS-SWFSC-564.

Southern Oregon/Northern California Coast coho

Overall vulnerability—Very high (28% High, 72% Very high)
Biological sensitivity—Very high (44% High, 56% Very high)
Climate exposure—High (66% High, 34% Very high)
Adaptive capacity—Low (1.56)
Data quality—58% of scores ≥ 2

Southern Oregon/Northern California Coast Coho		Expert Scores	Data Quality	Expert Scores Plots (Portion by Category)
Sensitivity attributes	Early life history	2.5	1.8	
	Juvenile freshwater stage	3.7	2.8	
	Estuary stage	3.2	1.5	
	Marine stage	3.4	2.8	
	Adult freshwater stage	1.6	2.5	
	Cumulative life-cycle effects	3.3	1.5	
	Hatchery influence	2.2	2.8	
	Other stressors	3.6	2.3	
	Population viability	3.5	3	
	Ocean acidification sensitivity	1.8	1.8	
	Sensitivity Score	High		
Exposure variables	Stream temperature	3.7	2	
	Summer water deficit	2.7	1.8	
	Flooding	3.4	1.3	
	Hydrologic regime	1.4	2.3	
	Sea level rise	3.3	2	
	Sea surface temperature	3.3	2	
	Ocean acidification exposure	4	2.5	
	Upwelling	2.9	1	
	Ocean currents	1.9	1	
	Exposure Score	High		
Overall Vulnerability Rank		High		

Low · Moderate · High · Very High

Life History Synopsis

In general, Southern Oregon/Northern California Coast coho salmon exhibit a 3-year life cycle. Adults enter natal streams and rivers from mid-November to January, coincident with the onset of rain-induced freshets in fall or early winter. However, entry into the Rogue River can occur as early as late August or early September (Weitkamp et al. 2015). Spawning may take place in small-to-moderate coastal streams and tributaries or larger rivers and usually occurs within a few days to a few weeks of freshwater entry. Depending upon water temperature, eggs incubate for approximately 8-12 weeks before hatching, after which alevins continue to reside in the gravel for an additional 2-8 weeks (Sandercock 1991). Fry emerge in early spring.

Juveniles exhibit a range of behaviors, life histories, and habitat associations. The majority reside about one year in fresh water before migrating to sea. Some juveniles may reside in estuaries for variable periods depending on temperature, dissolved oxygen, and salinity levels, but they subsequently move back upstream without entering the ocean (Miller and Sadro 2003, Koski 2009; Jones et al. 2014; Wallace et al. 2015). Wallace et al. (2015) found that the estuaries of stream tributaries to Humboldt Bay provided major rearing habitat for coho juveniles, including the estuaries of small streams in which no coho spawn.

In more northern latitudes, a significant proportion of juvenile coho may spend a second or even third full year in fresh water (Sandercock 1991). This life-history pattern has also been documented in the California portion of this Southern Oregon/Northern California Coast coho DPS (Bell and Duffy 2007) and in the Central California Coast coho DPS to the south (Gallagher et al. 2014). However, only a small-to-modest fraction of these fish may spend a second year in fresh water before migrating to sea.

During winter, juvenile coho need refuge from high, turbid flows. Typically, these refuges are side-channels (Bell 2001), complex masses of large woody debris, and small tributaries (Ebersole et al. 2006).

For Southern Oregon/Northern California Coast coho, the smolt migration occurs in spring, generally peaking in April or May (Weitkamp et al. 1995). Migratory behavior can be triggered by a range of environmental stimuli (Spence and Dick 2014), reflecting adaptation to differences in the timing and predictability of favorable ocean conditions (Spence and Hall 2010). Characteristics that have been recognized as influencing migratory behavior include stream flow, fish size, day length, water temperature, and food density.

The ocean phase of coho salmon typically lasts about 1.5 years; however, a proportion of male fish return after only 6 months at sea as precocious males, or jacks, which are substantially smaller than adults returning after a full 18 months (Weitkamp et al. 1995). The proportion of precocious males in the escapement population can be highly variable (Sandercock 1991).

After entering the ocean, juvenile coho initially remain in nearshore waters close to the natal stream, as suggested by tag recovery data from hatchery populations north of this DPS (Beamish et al. 2018). Coho salmon can range widely in the North Pacific Ocean, but specific ocean movements of Southern Oregon/Northern California Coast coho are poorly understood. Beamish et al. (2018) provide a detailed overview of coho marine diets. In general, they summarize coho salmon off the coast of North America as opportunistic visual predators. Typical prey items found in fish collected from the California Current, included larval or juvenile fishes, decapod larvae, euphausiids, amphipods, juvenile squid, and terrestrial insects.

Climate Effects on Abundance and Distribution

An understanding of the geographical make-up of the Southern Oregon/Northern California Coast coho DPS is critical to understanding climate effects on its abundance and distribution. The geographic setting of this DPS includes three large basins and numerous smaller basins across a diverse landscape. The Rogue and Klamath Basins extend beyond the Coast Range and include the Cascade Mountains. The Eel River Basin also extends well inland, including portions at relatively high elevation and portions that experience dryer and warmer summer temperatures. Numerous moderate and smaller coastal basins of the DPS experience relatively wet, cool, and temperate conditions. These contrast with conditions in the Rogue, Klamath, and Eel interior sub-basins, which range from snowmelt-driven hydrographs to hot, dry summers to cold winters. In terms of environmental conditions, the lower-elevation portions of these large basins are more similar to the smaller coastal basins than they are to the interior sub-basins (Williams et al. 2006).

Predicted problems faced by Southern Oregon/Northern California Coast coho include increases in *stream temperature*, for which sensitivity was rated ***high***, as well as increased variability in flows, reflected in a ***high*** score for *flooding*. This DPS may also be affected by changed ocean conditions, although the nature of such impacts remains highly uncertain.

Overall sensitivity attributes were ranked ***high***, although in bootstrap resampling, a majority of resamples produced a ***very high*** score. This DPS was borderline between ***high*** and ***very high***, indicating that many individual attributes included multiple very high tallies. Nearly all life stages contributed to this result, with sensitivity ranking ***very high*** at the *juvenile freshwater stage*, ***high*** at the *estuary* and *marine stages*, and ***high*** for *cumulative life-cycle effects*.

This DPS also ranked ***high*** for exposure overall. Contributing to this overall score were ***high*** rankings for *flooding*, *sea surface temperature*, and *sea level rise*. Because juvenile coho rear in rivers over the summer, they are highly sensitive to stream temperature and thus highly vulnerable in the juvenile freshwater life stage. Thus Southern Oregon/Northern California Coast coho ranked ***very high*** in exposure to *stream temperature*. The high exposure rank of this DPS for *flooding* reflected potential effects of flood risk on the *early life history* and *adult freshwater stage*, because winter storms overlap both adult migrations and spawning. In the future, the combination of changes in storm dynamics and sea level rise may impact lagoon habitat in ways that have not been fully explored. Nonetheless, this DPS is especially diverse in its dependence on lagoon habitat. Specifically, components from the three large inland basins could contribute to greater resilience to sea level rise for this DPS than for the strictly coastal ESUs further south.

Extrinsic Factors

Climate change impacts to Southern Oregon/Northern California Coast coho are superimposed on stressors such as dams, water diversions, and erosion from logging. Thus sensitivity to *other stressors* was ranked ***very high***. These threats increase the likelihood of rapid extirpation as time passes without dramatic action to protect and enhance habitats. Such dramatic action is planned with the removal of four dams on the Klamath River, which is scheduled to occur in 2021. In addition, genetic management plans are in place for all three hatcheries influencing this DPS, and multiple dams have been removed on the Rogue River.

Southern Oregon/Northern California Coast coho is listed as threatened under the U.S. Endangered Species Act, and sensitivity for *population viability* was ranked ***high***. Although long-term data on abundance in this DPS are scarce, the last two viability assessments indicate little change in abundance trends (Williams et al. 2016). All populations fail to meet viability criteria, although all are extant, unlike the Central California Coast coho salmon DPS immediately to the south.

Adaptive Capacity

Because this DPS is near the southern range limit for coho, it appears to have little capacity to adapt to warmer streams, and adaptive capacity was ranked ***low*** for Southern Oregon/Northern California Coast coho salmon. However, habitat is particularly diverse within this DPS, and this diversity will likely cause greater differentiation in response to climate impacts and possibly a wider range of responses than possible for the more southerly Central California Coast coho salmon DPS. The three large basins that penetrate coastal mountain ranges include snowmelt-driven hydrographs, hot dry summers, and cold winters, while the numerous moderate and smaller coastal basins inhabited by this DPS experience relatively wet, cool, and temperate conditions. The contrast between coastal and interior sub-basins provides a range of environmental conditions that will most likely be impacted differently by climate-driven changes.

Literature Cited

Beamish, R. J., L. A. Weitkamp, L. D. Shaul, and V. I. Radchenko. 2018. Ocean ecology of Coho Salmon. Pages 391-554 *in* R. J. Beamish, editor. The ocean ecology of Pacific salmon and trout. American Fisheries Society, Bethesda.

Bell, E. 2001. Survival, growth and movement of juvenile coho salmon (*Oncorhynchus kisutch*) over wintering in alcoves, backwaters, and main channel pools in Prairie Creek, California. M.S. Thesis, Humboldt State University, Arcata, CA.

Bell, E. and W. G. Duffy. 2007. Previously undocumented two-year freshwater residency of juvenile coho salmon in Prairie Creek, California. Trans. Am. Fish. Soc. 136:966-970.

Ebersole, J. L., P. J. Wigington, J. P. Baker, M. A. Cairns, M. R. Church, B. P. Hansen, B. A. Miller, H. R. LaVigne, J. E. Compton, and S. G. Leibowitz. 2006. Juvenile Coho salmon growth and survival across stream network seasonal habitats. Trans. Am. Fish. Soc. 135:1681-1697.

Gallagher, S. P., S. A. Thompson, and D. W. Wright. 2014. Coastal Mendocino County salmonid life cycle and regional monitoring: monitoring status and trends 2013. California Department of Fish and Wildlife, Fort Bragg, CA.

Jones, K. K., T. J. Cornwell, D. L. Bottom, L. A. Campbell, and S. Stein. 2014. The contribution of estuary-resident life histories to the return of adult *Oncorhynchus kisutch*. J. Fish. Biol. 85:52-80.

Koski, K. 2009. The fate of coho salmon nomads: the story of estuarine rearing strategy promoting resilience. Ecol. Soc. 14:4.

Miller, B. A. and S. Sadro. 2003. Residence time and seasonal movements of juvenile coho salmon in the ecotone and lower estuary of Winchester Creek, South Slough, Oregon. Trans. Am. Fish. Soc. 132:546-559.

Sandercock, F. K. 1991. Life history of coho salmon (*Oncorhynchus kisutch*).*in* C. Groot and L. Margolis, editors. Pacific Salmon Life Histories. UBC, Press, Vancouver, BC, Canada.

Spence, B. C. and E. J. Dick. 2014. Geographic variation in environmental factors regulating outmigration timing of coho salmon (*Oncorhynchus kisutch*) smolts. Can. J. Fish. Aquat. Sci. 71:56-69.

Spence, B. C. and J. D. Hall. 2010. Spatiotemporal patterns in migration timing of coho salmon (*Oncorhynchus kisutch*) smolts in North America. Can. J. Fish. Aquat. Sci. 67:1316-1334.

Wallace, M., S. Ricker, A. Garwood, A. Frimodig, and S. Allen. 2015. Importance of the stream estuary ecotone to juvenile coho salmon (*Oncorhynchus kisutch*) in Humboldt Bay, California. Calif. Fish Game 101:241-266.

Weitkamp, L. A., D. J. Teel, M. Liermann, S. A. Hinton, D. M. Van Doornik, and P. J. Bentley. 2015. Stock-specific size and timing at ocean entry of Columbia River juvenile Chinook salmon and steelhead: Implications for early ocean growth. Mar. Coast. Fish. 7:370-392.

Weitkamp, L. A., T. C. Wainwright, G. J. Bryant, G. B. Milner, D. J. Teel, R. G. Kope, and R. S. Waples. 1995. Status review of coho salmon from Washington, Oregon, and California. U.S. Dep. Commerce NOAA Tech. Memo. NMFS-NWFSC-24.

Williams, T. H., E. P. Bjorkstedt, W. Duffy, D. Hillemeier, G. Kautsky, T. Lisle, M. McCain, M. Rode, R. G. Szerlong, R. Schick, M. Goslin, and A. Agrawal. 2006. Historical population structure of coho salmon in the Southern Oregon/Northern California Coasts Evolutionarily Significant Unit National Marine Fisheries Service, Southwest Fisheries Science Center, Santa Cruz, CA.

Williams, T. H., B. C. Spence, D. A. Boughton, R. C. Johnson, L. G. Crozier, N. J. Mantua, M. R. O'Farrell, and S. T. Lindley. 2016. Viability assessment for Pacific salmon and steelhead listed under the Endangered Species Act: Southwest U.S. Dep. Commerce NOAA Tech. Memo. NMFS-SWFSC-564.

Oregon Coast coho

Overall vulnerability—Moderate (60% Moderate, 40% High)
Biological sensitivity—Moderate (58% Moderate, 42% High)
Climate exposure—High (6% Moderate, 94% High)
Adaptive capacity—Moderate (2.0)
Data quality—89% of scores ≥ 2

	Oregon Coast Coho	Expert Scores	Data Quality	Expert Scores Plots (Portion by Category)
Sensitivity attributes	Early life history	1.8	2.8	
	Juvenile freshwater stage	3.1	2.8	
	Estuary stage	2.3	2.8	
	Marine stage	3	2.3	
	Adult freshwater stage	1.6	3	
	Cumulative life-cycle effects	2.1	2.3	
	Hatchery influence	1.3	2.8	
	Other stressors	2.6	2.8	
	Population viability	2.2	3	
	Ocean acidification sensitivity	1.9	1	
	Sensitivity Score	High		
Exposure variables	Stream temperature	3.2	2.8	
	Summer water deficit	2.7	2	
	Flooding	1.6	2	
	Hydrologic regime	1.3	3	
	Sea level rise	2	2	
	Sea surface temperature	2.8	2.5	
	Ocean acidification exposure	3.9	3	
	Upwelling	1.7	2	
	Ocean currents	1.9	1	
	Exposure Score	High		
Overall Vulnerability Rank		High		

Low / Moderate / High / Very High

Life History Synopsis

Oregon Coast coho salmon displays an ocean-maturing phenotype, with adults generally returning to spawn in freshwater tributaries from late September through January. This late run timing is especially characteristic of populations inhabiting large coastal lakes in the central coast (Ford et al. 2004). Adults tend to enter larger rivers earlier and may delay migration into smaller tributaries until precipitation and stream discharge is sufficient (Clark et al. 2014). Information on coho age at reproduction is summarized by Weitkamp et al. (1995). Females return predominately at age 3, after 18 months at sea, whereas males may return at age 3 or ~age 2, after only 6 months at sea.

Eggs of Oregon Coast coho salmon incubate from October through March, and juveniles emerge throughout late winter and spring. Like other coho salmon in this region, most juveniles enter the ocean at age 1 (Weitkamp et al. 1995). Juveniles have been shown to make extensive use of estuary habitats when they are available (Wainwright and Weitkamp 2013). Females of this DPS typically spend about 16 months at sea (Sandercock 1991), while male ocean residence time varies as indicated above.

Oregon coast coho is widely distributed in marine waters. During the first summer, some individuals remain in local waters off the Oregon and Washington coasts, while others move rapidly northward along the continental shelf off Alaska (Morris et al. 2007; Van Doornik et al. 2007; Beamish et al. 2018). As maturing adults, fish from this DPS are caught in coastal fisheries from Vancouver Island to Monterrey Bay (Weitkamp and Neely 2002).

Climate Effects on Abundance and Distribution

In September, early returning adults may encounter seasonally warm temperatures or low flows that delay entry into spawning tributaries. However, Oregon Coast coho adults will typically hold in estuaries or larger rivers and rapidly ascend tributaries to spawn when conditions become suitable (Clark et al. 2014). Autumnal drops in stream temperature and increases in stream discharge improve conditions for adult migration as well as egg incubation. Thus, incubating eggs are unlikely to be exposed to excessively warm temperatures or desiccation.

Because juveniles typically spend at least one year in fresh water, they can be exposed to warm summer conditions or stress from low flows (Ebersole et al. 2009). In winter, exposure to floods may displace juveniles or reduce egg survival (Nickelson et al. 1992). Flood exposure was expected to change somewhat less for this DPS than for those with more southerly populations, which were projected to face larger changes in flooding due to atmospheric rivers. Oregon Coast coho ranked ***high*** in sensitivity at the *juvenile freshwater stage* and in exposure to *stream temperature*; thus the juvenile freshwater stage for this DPS was considered a ***highly vulnerable life stage***. Exposures may vary significantly among major freshwater habitats where coho juveniles rear. These habitats include coastal dune lakes, coastal tributaries, and the Umpqua River—the only habitat occupied by this DPS that includes the Cascade Mountains within its catchment (Wainwright and Weitkamp 2013). The former two habitats frequently warm to levels that pose physiological or survival challenges to coho salmon, and also support a large contingent of non-native warm-water fishes, which may have negative ecological effects (Sanderson et al. 2009). However, declines in snowpack could also have negative consequences for coho inhabiting inland tributaries of the Umpqua River.

Information regarding the potential sensitivity of Oregon Coast coho salmon to climate change is of mixed quality. However, it is certain that exposure to changing marine conditions will occur, for example, with increasing levels of *ocean acidification*.

The importance of marine conditions to productivity (number and size of returning adults) was stressed by Wainwright and Weitkamp (2013), and Oregon Coast coho scored ***high*** in sensitivity at the *marine stage* in this assessment. However, data quality for these threats was limited.

Extrinsic Factors

Although production hatcheries were once more prevalent in the recovery domain of Oregon Coast coho, hatchery propagation has been significantly reduced to only three facilities (NMFS 2011). Accordingly, sensitivity of this DPS to *hatchery influence* was ranked ***low***. Sensitivity to *other stressors* was ranked ***moderate***. Oregon Coast coho salmon is listed as threatened under the U.S. Endangered Species Act. However, it is considered at lower risk of extinction than other coho DPSs and scored ***low*** for *population viability*.

Adaptive Capacity

Oregon Coast coho ranked ***moderate*** for adaptive capacity, as it likely has flexibility in the juvenile rearing period similar to that of other coho salmon. Adults are less constrained in freshwater entry timing than California coho, and thus could potentially respond temporally to changing environmental conditions.

Literature Cited

Much of the information here is summarized from a recent review of climate effects on Oregon Coast coho salmon by Wainwright and Weitkamp (2013).

Beamish, R. J., L. A. Weitkamp, L. D. Shaul, and V. I. Radchenko. 2018. Ocean ecology of coho salmon. Pages 391-554 *in* R. J. Beamish, editor. The ocean ecology of Pacific salmon and trout. American Fisheries Society, Bethesda.

Clark, S. M., J. B. Dunham, J. R. McEnroe, and S. W. Lightcap. 2014. Breeding site selection by coho salmon (*Oncorhynchus kisutch*) in relation to large wood additions and factors that influence reproductive success. Can. J. Fish. Aquat. Sci. 71:1498-1507.

Ebersole, J. L., M. E. Colvin, P. J. Wigington Jr, S. W. Lightcap, J. P. Baker, M. R. Church, J. E. Compton, and M. A. Cairns. 2009. Hierarchical modeling of late summer weight and summer abundance of juvenile coho salmon across a stream network. Trans. Am. Fish. Soc. 138:1138-1156.

Ford, M. J., D. Teel, D. M. Van Doornik, and P. W. Lawson. 2004. Genetic population structure of central Oregon Coast coho salmon (*Oncorhynchus kisutch*). Conserv. Genet. 5:797–812.

Morris, J. F. T., M. Trudel, M. E. Thiess, R. M. Seeting, J. Fisher, S. A. Hinton, E. Ferguson, J. A. Orsi, E. Farley, and D. W. Welch. 2007. Stock-specific migrations of juvenile Coho salmon derived from coded-wire tag recoveries on the continental shelf of western North America. Am. Fish. Soc. Symp. 57:81-104.

Nickelson, T. E. R., J.D., S. L. Johnson, and M. F. Solazzi. 1992. Seasonal changes in habitat use by juvenile coho salmon (*Oncorhynchus kisutch*) in Oregon coastal streams. Can. J. Fish. Aquat. Sci. 49:783-789.

NMFS (National Marine Fisheries Service). 2011. Listing endangered and threatened species: threatened status for the Oregon Coast coho salmon evolutionarily significant unit. U.S. Dep.Commerce Federal Register 50 CFR Part 223 Docket No. 110531311–1310–02, Silver Spring, MD.

Sandercock, F. K. 1991. Life history of coho salmon (*Oncorhynchus kisutch*).*in* C. Groot and L. Margolis, editors. Pacific Salmon Life Histories. UBC, Press, Vancouver, BC, Canada.

Sanderson, B. L., K. A. Barnas, and A. M. W. Rub. 2009. Nonindigenous species of the Pacific Northwest: an overlooked risk to endangered salmon? BioScience 59:245-256.

Van Doornik, D. M., D. J. Teel, D. R. Kuligowski, C. A. Morgan, and E. Casillas. 2007. Genetic analyses provide insight into the early ocean stock distribution and survival of juvenile Coho salmon off the coasts of Washington and Oregon. N. Am. J. Fish. Manage. 27:220-237.

Wainwright, T. C. and L. A. Weitkamp. 2013. Effects of climate change on Oregon coast Coho salmon: Habitat and life-cycle interactions. Northwest Sci. 87:219-242.

Weitkamp, L. and K. Neely. 2002. Coho salmon (*Oncorhynchus kisutch*) ocean migration patterns: insight from marine coded-wire tag recoveries. Can. J. Fish. Aquat. Sci. 59:1100-1115.

Weitkamp, L. A., T. C. Wainwright, G. J. Bryant, G. B. Milner, D. J. Teel, R. G. Kope, and R. S. Waples. 1995. Status review of coho salmon from Washington, Oregon, and California. U.S. Dep. Commerce NOAA Tech. Memo. NMFS-NWFSC-24.

Lower Columbia River coho

Overall vulnerability—High (30% Moderate, 60% High)
Biological sensitivity—High (29% Moderate, 71% High)
Climate exposure—High (2% Moderate, 98% High)
Adaptive capacity—Moderate (2.4)
Data quality—89% of scores ≥ 2

	Lower Columbia River Coho	Expert Scores	Data Quality	Expert Scores Plots (Portion by Category)
Sensitivity attributes	Early life history	1.7	2.8	
	Juvenile freshwater stage	3.2	2.8	
	Estuary stage	1.8	2.8	
	Marine stage	3	2.3	
	Adult freshwater stage	1.7	3	
	Cumulative life-cycle effects	2	2.3	
	Hatchery influence	2.9	2.8	
	Other stressors	2.5	2.5	
	Population viability	2.9	3	
	Ocean acidification sensitivity	1.9	1	
	Sensitivity Score	High		
Exposure variables	Stream temperature	3.3	2.8	
	Summer water deficit	2.7	2	
	Flooding	1.5	2	
	Hydrologic regime	2.2	3	
	Sea level rise	2	2	
	Sea surface temperature	2.8	2.5	
	Ocean acidification exposure	3.9	3	
	Upwelling	1.8	2	
	Ocean currents	1.9	1	
	Exposure Score	High		
Overall Vulnerability Rank		High		

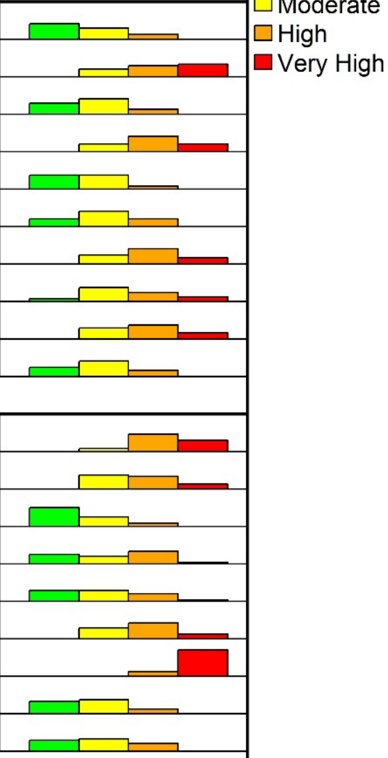

Life History Synopsis

Lower Columbia River coho salmon is an ocean-maturing phenotype typical of its species, with adults returning to spawn in freshwater tributaries of the lower Columbia Basin from late September through November. Fish tend to enter larger rivers earlier, and may delay migration into smaller tributaries until precipitation and stream discharge is sufficient (Clark et al. 2014). Information on age at reproduction indicates that females predominately return at age 3, after 18 months at sea, whereas males may return at age 3 or 2, after only 6 months at sea (Weitkamp et al. 1995).

Eggs of Lower Columbia River coho salmon incubate from October through March, with juveniles emerging throughout late winter and spring. Most juveniles enter

the ocean at age 1 (Weitkamp et al. 1995). Juvenile migrants are believed to move quickly through the Columbia River estuary, although some individuals may make extensive use of estuarine habitats (Weitkamp et al. 2012; Craig et al. 2014). Upon entry into the open ocean, juveniles use nearshore marine habitats with some fish remaining in local waters and others moving northward along the continental shelf to central Alaska (Van Doornik et al. 2007; Fisher et al. 2014; Beamish et al. 2018). Maturing coho salmon are caught in ocean fisheries from Vancouver Island to Monterrey Bay (Weitkamp and Neely 2002). Females typically spend about 16 months at sea (Sandercock 1991), while male residence times are variable, as noted above.

Climate Effects on Abundance and Distribution

In September, early-returning adults may encounter seasonally warm temperatures or low flows that delay entry into spawning tributaries. However, adults will typically hold in estuaries or larger rivers and rapidly ascend tributaries to spawn when conditions become suitable (Clark et al. 2014). Seasonal drops in stream temperature and increases in discharge improve conditions for adult migration as well as egg incubation. Thus, incubating eggs of Lower Columbia River coho salmon are unlikely to be exposed to excessively warm temperatures or desiccation.

Because coho juveniles typically spend at least one year in fresh water, they can be stressed by warm stream conditions or low flows in summer (Ebersole et al. 2009) and by floods that may displace them or reduce available habitat in winter (Nickelson et al. 1992). ***High*** ranks for sensitivity in the *juvenile freshwater stage* and for exposure to *stream temperatures* were reflective of these findings, and resulted in the juvenile freshwater stage rank as a ***highly vulnerable life stage*** for Lower Columbia River coho. Though the quality of information was mixed, sensitivity in the *marine stage* is ranked ***high*** because of the relatively high certainty of exposure to changing marine conditions will occur, namely high levels of *ocean acidification*. However, data quality used to evaluate climate-related threats was limited, and future evidence may alter these rankings.

Extrinsic Factors

Although production hatcheries are present and were historically more prevalent, the latest status review reported that multiple populations consist predominantly of natural spawners (Ford et al. 2015); thus Lower Columbia River coho ranked ***moderate*** in sensitivity to *hatchery influence*. Similarly, the DPS ranked ***moderate*** for the importance of *other stressors,* such as dams that block access to colder spawning tributaries and effects of urbanization. Lower Columbia River coho salmon is listed as threatened under the U.S. Endangered Species Act, and *population viability* was ranked ***moderate*** for this DPS.

Adaptive Capacity

The adaptive capacity of Lower Columbia coho was ranked ***moderate***. This DPS likely has an amount of flexibility in the juvenile rearing period similar to that of other coho. Adults in this DPS are less constrained in freshwater entry timing than California coho, and thus could potentially respond temporally to changing environmental conditions.

Literature Cited

Much information in the section on *Climate Effects on Abundance and Distribution* section was summarized from a recent review by Wainwright and Weitkamp (2013), which focused on Oregon Coast coho salmon. This DPS shares much in common with the Lower Columbia River coho DPS in terms of climate sensitivity and exposure.

Beamish, R. J., L. A. Weitkamp, L. D. Shaul, and V. I. Radchenko. 2018. Ocean ecology of Coho Salmon. Pages 391-554 *in* R. J. Beamish, editor. The ocean ecology of Pacific salmon and trout. American Fisheries Society, Bethesda.

Clark, S. M., J. B. Dunham, J. R. McEnroe, and S. W. Lightcap. 2014. Breeding site selection by coho salmon (*Oncorhynchus kisutch*) in relation to large wood additions and factors that influence reproductive success. Can. J. Fish. Aquat. Sci. 71:1498-1507.

Craig, B. E., C. A. Simenstad, and D. L. Bottom. 2014. Rearing in natural and recovering tidal wetlands enhances growth and life-history diversity of Columbia Estuary tributary coho salmon *Oncorhynchus kisutch* population. J. Fish. Biol. 85:31-51.

Ebersole, J. L., M. E. Colvin, P. J. Wigington Jr, S. W. Lightcap, J. P. Baker, M. R. Church, J. E. Compton, and M. A. Cairns. 2009. Hierarchical modeling of late summer weight and summer abundance of juvenile coho salmon across a stream network. Trans. Am. Fish. Soc. 138:1138-1156.

Fisher, J. P., L. A. Weitkamp, D. J. Teel, S. A. Hinton, J. A. Orsi, E. V. Farley, J. F. T. Morris, M. E. Thiess, R. M. Sweeting, and M. Trudel. 2014. Early ocean dispersal patterns of Columbia River Chinook and coho salmon. Trans. Am. Fish. Soc. 143:252-272.

Ford, M. J., K. Barnas, T. Cooney, L. G. Crozier, M. Diaz, J. J. Hard, E. E. Holmes, D. M. Holzer, R. G. Kope, P. W. Lawson, M. Liermann, J. M. Myers, M. Rowse, D. J. Teel, D. M. V. Doornik, T. C. Wainwright, L. A. Weitkamp, and M. Williams. 2015. Status review update for Pacific salmon and steelhead listed under the Endangered Species Act: Pacific Northwest. Report of the National Marine Fisheries Service, Northwest Fisheries Science Center. Available from www.nwfsc.noaa.gov/publications/scipubs/searchdoc.cfm.

Nickelson, T. E. R., J.D., S. L. Johnson, and M. F. Solazzi. 1992. Seasonal changes in habitat use by juvenile coho salmon (*Oncorhynchus kisutch*) in Oregon coastal streams. Can. J. Fish. Aquat. Sci. 49:783-789.

Sandercock, F. K. 1991. Life history of coho salmon (*Oncorhynchus kisutch*).*in* C. Groot and L. Margolis, editors. Pacific Salmon Life Histories. UBC, Press, Vancouver, BC, Canada.

Van Doornik, D. M., D. J. Teel, D. R. Kuligowski, C. A. Morgan, and E. Casillas. 2007. Genetic analyses provide insight into the early ocean stock distribution and survival of juvenile Coho salmon off the coasts of Washington and Oregon. N. Am. J. Fish. Manage. 27:220-237.

Wainwright, T. C. and L. A. Weitkamp. 2013. Effects of climate change on Oregon coast Coho salmon: Habitat and life-cycle interactions. Northwest Sci. 87:219-242.

Weitkamp, L. A., P. J. Bentley, and M. N. C. Litz. 2012. Seasonal and interannual variation in juvenile salmonids and associated fish assemblage in open waters of the lower Columbia River estuary. Fish. Bull. U.S. 110:426-450.

Weitkamp, L. A. and K. Neely. 2002. Coho salmon (*Oncorhynchus kisutch*) ocean migration patterns: insight from marine coded wire tag recoveries. Can. J. Fish. Aquat. Sci. 59:1100-1115.

Weitkamp, L. A., T. C. Wainwright, G. J. Bryant, G. B. Milner, D. J. Teel, R. G. Kope, and R. S. Waples. 1995. Status review of coho salmon from Washington, Oregon, and California. U.S. Dep. Commerce NOAA Tech. Memo. NMFS-NWFSC-24.

Puget Sound coho

Overall vulnerability—High (8% Moderate, 92% High)
Biological sensitivity—High (7% Moderate, 93% High)
Climate exposure—High (1% Moderate, 99% High)
Adaptive capacity—High (2.1)
Data quality—89% of scores ≥ 2

	Puget Sound Coho	Expert Scores	Data Quality	Expert Scores Plots (Portion by Category)
Sensitivity attributes	Early life history	2.1	2.8	
	Juvenile freshwater stage	3.1	2.8	
	Estuary stage	2	2.8	
	Marine stage	3.1	2.3	
	Adult freshwater stage	1.9	3	
	Cumulative life-cycle effects	1.9	2.3	
	Hatchery influence	2.9	2.8	
	Other stressors	3.3	2.8	
	Population viability	2.1	3	
	Ocean acidification sensitivity	1.9	1	
	Sensitivity Score	High		
Exposure variables	Stream temperature	3.2	2.8	
	Summer water deficit	2.7	2	
	Flooding	1.8	2	
	Hydrologic regime	3.1	2.8	
	Sea level rise	2.5	2	
	Sea surface temperature	2.9	2.5	
	Ocean acidification exposure	3.9	3	
	Upwelling	1.5	2	
	Ocean currents	1.8	1	
	Exposure Score	High		
Overall Vulnerability Rank		High		

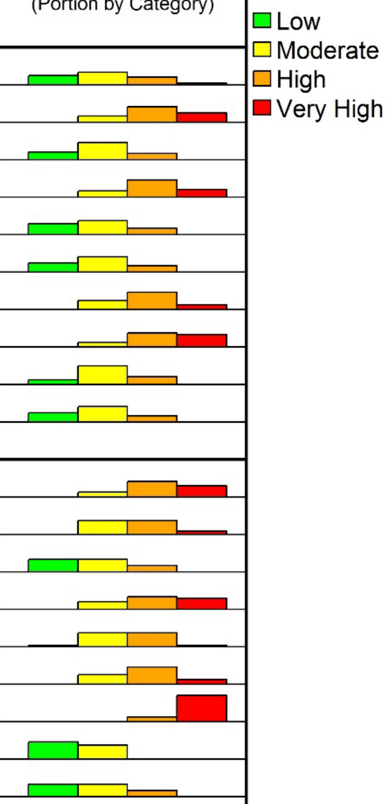

Life History Synopsis

Puget Sound coho adults migrate relatively short distances (<1-150 km) upstream to spawning grounds. Migration begins in late summer or early fall and is often timed with rain events. Coho take advantage of shallower floodplain channels to spawn (Weitkamp et al. 1995). Most coho smolts migrate at age 1+, but a significant proportion may migrate in spring or fall at age 0+. Subyearling coho rear in estuaries, but may employ several strategies. Some rear in the estuary of the natal river and then move to nearshore areas or move back upstream. Others leave as fry and find small, non-natal rearing streams. Average rearing time for age 1+ fish is less than one week, while rearing time is likely more than 4-10 weeks for subyearlings (Beamer et al. 2010; Koski 2009; Jones et al. 2014).

Most Puget Sound coho move directly into deeper waters of Puget Sound and then out to coastal waters from Oregon to northern British Columbia (Morris et al. 2007; Van Doornik et al. 2007). However, some may rear for an extended time within Puget Sound and may be captured in deeper waters of the sound into November. Most fish migrate relatively short distances in the ocean, and maturing fish are caught in fisheries from Vancouver Island to the Washington coast and within the Salish Sea (Weitkamp et al. 1995). Early marine climate signals explain a large proportion of the variation in total return rate (Beamish et al. 2000; Beamish and Mahnken 2001; Zimmerman et al. 2015a). Almost all females return after 2 winters at sea, although males may return as jacks (Weitkamp et al. 1995).

Climate Effects on Abundance and Distribution

Few studies have examined the effect of climate factors on the population productivity of Puget Sound coho. In colder systems, some juveniles migrate at age 2+ (Zimmerman et al. 2015a) Rearing in rivers is likely limited by low flows in summer. High flows in winter may also impact survival, particularly in areas with few floodplains, side channels, or off-channel rearing areas. This was reflected in the ***high*** sensitivity rank for this DPS at the *juvenile freshwater stage*. Summer temperature barriers exist in some lowland river systems, and these conditions are likely to worsen under climate change. Puget Sound coho also ranked ***high*** in exposure to *stream temperature* and *hydrologic regime* shift, causing its *juvenile freshwater stage* to be ranked as a ***highly vulnerable life stage***. Based on our analysis, by 2080, about 10% of Puget Sound coho spawning habitat is expected to shift from transitional to rain-dominated, and about 2% from snow-dominated to transitional (Appendix S2). Yearlings do not rear extensively in estuaries, but use them as refuges during large floods.

Climate change will likely reduce freshwater rearing capacity as a result of summer low flows and high water temperatures, thereby increasing the proportion of estuarine-dependent type juveniles. However, rearing in estuaries is likely already temperature-limited in late spring and summer (Miller and Sadro 2003; Hall et al. 2018). Delayed rainfall can interrupt adult migration and extend exposure of adults to mortality risk within Puget Sound, as well as present spawn timing challenges (Weitkamp et al. 1995). All of these factors could greatly impact marine survival. Early marine climate signals such as temperature, upwelling, or sign of the Pacific Decadal Oscillation explained a large proportion of variation in total return rate for coho, suggesting climate change may have significant impacts (Beamish et al. 2000; Beamish and Mahnken 2001; Zimmerman et al. 2015a). These risks were reflected in a ***high*** sensitivity score for Puget Sound coho at the *marine stage*.

There may be linkages between declining adult body size, egg size, and juvenile size at migration, leading to life-cycle based shifts in total mortality (Holtby et al. 1990). Some of these shifts may be the consequence of marine or incubation thermal regimes

(Quinn et al. 2004). Nonetheless, Puget Sound coho was not considered at imminent risk from these shifts and ranked ***low*** for *cumulative life-cycle effects*.

Sea level rise is affecting the amount of rearing habitat, but these losses may be offset in some places by higher sedimentation rates. This DPS ranked ***moderate*** in exposure to *sea level rise*. Juvenile coho feed on marine arthropods, including crab larvae, krill, and amphipods. Some crabs, including Dungeness crab are thought to be sensitive to direct effects of ocean acidification (Busch and McElhany 2016), as well as to indirect effects of ocean acidification on the benthic food web (Marshall et al. 2017; Hodgson et al. 2018). Fish larvae are also an important food source for coho, and may be physiologically sensitive to ocean acidification (Daly et al. 2009; Sweeting and Beamish 2009). Owing to these potential indirect effects, Puget Sound coho, as all DPSs in this analysis, ranked ***very high*** in exposure to *ocean acidification*.

Extrinsic Factors

Some regions of Puget Sound are experiencing losses in primary production and increases in eutrophication, possibly due to anthropogenic nutrient inputs (Snover et al. 2005). These increases can be exacerbated by higher temperatures and increased stratification, and could impact fish via losses in secondary production and hypoxia (Snover et al. 2005). In addition, when high flow events are preceded by extended dry periods, non-point source pollutants may accumulate rapidly in streams and cause pre-spawn, egg, and juvenile mortality (Scholz et al. 2011). These factors contributed to an overall ranking for *other stressors* as *high*.

Puget Sound coho is not presently protected under the Endangered Species Act and ranked ***low*** in sensitivity to *population viability*. However, populations of this DPS historically have low abundance, particularly those in south Puget Sound. Likely this is a result of lost or degraded spawning habitat in small creeks, as well as loss of floodplains in larger rivers (Pess et al. 2002; Zimmerman et al. 2015b).

Adaptive Capacity

Puget Sound coho ranked ***high*** in *adaptive capacity*. Climate change will likely reduce summer rearing capacity as a result of lower flows and higher temperatures, but coho retain the capacity to shift to a more estuarine-dependent life history type. Historically, coho in Puget Sound had a more protracted spawning period, which extended to March in some rivers. Under higher winter flood scour conditions, later spawn timing might be more successful. (Weitkamp et al. 1995). Adults also display climate-sensitive plasticity in run timing, which might allow them to avoid high stream temperature prior to spawning and subsequent maladaptive early emergence of fry.

Literature Cited

Beamer, E., R. Henderson, and K. Wolf. 2010. Juvenile salmon, estuarine, and freshwater fish utilization of habitat associated with the Fisher Slough Restoration Project, Washington 2009. Report of the Skagit River System Cooperative to the Nature Conservancy. Available http://skagitcoop.org/wp-content/uploads/FisherSlough2009Report_Final.pdf.

Beamish, R. J. and C. Mahnken. 2001. A critical size and period hypothesis to explain natural regulation of salmon abundance and the linkage to climate and climate change. Oceanography 49:423-435.

Beamish, R. J., D. J. Noakes, G. A. McFarlane, W. Pinnix, R. Sweeting, and J. King. 2000. Trends in coho marine survival in relation to the regime concept. Fish. Oceanogr. 9:114-119.

Busch, D. S. and P. McElhany. 2016. Estimates of the direct effect of seawater pH on the survival rate of species groups in the California Current ecosystem. PLoS ONE 11:e0160669.

Daly, E. A., R. D. Brodeur, and L. A. Weitkamp. 2009. Ontogenetic shifts in diets of juvenile and subadult Coho and Chinook salmon in coastal marine waters: Important for marine survival? Trans. Am. Fish. Soc. 138:1420-1438.

Hall, J. E., T. P. Khangaonkar, C. A. Rice, J. Chamberlin, T. Zackey, F. Leonetti, M. Rustay, K. Fresh, A. Kagley, and M. Rowse. 2018. Characterization of salinity and temperature patterns in a large river delta to support tidal wetland habitat restoration. Northwest Sci. 92:36-52.

Hodgson, E. E., I. C. Kaplan, K. N. Marshall, J. Leonard, T. E. Essington, D. S. Busch, E. A. Fulton, C. J. Harvey, A. Hermann, and P. McElhany. 2018. Consequences of spatially variable ocean acidification in the California Current: Lower pH drives strongest declines in benthic species in southern regions while greatest economic impacts occur in northern regions. Ecol. Model. 383:106-117.

Holtby, L. B., B. C. Andersen, and R. Kadowaki. 1990. Importance of smolt size and early ocean growth to interannual variability in marine survival of coho salmon (*Oncorhynchus kisutch*). Can. J. Fish. Aquat. Sci. 47:2181-2194.

Jones, K. K., T. J. Cornwell, D. L. Bottom, L. A. Campbell, and S. Stein. 2014. The contribution of estuary-resident life histories to the return of adult *Oncorhynchus kisutch*. J. Fish. Biol. 85:52-80.

Koski, K. 2009. The fate of coho salmon nomads: the story of estuarine rearing strategy promoting resilience. Ecol. Soc. 14:4.

Marshall, K. N., I. Kaplan, E. E. Hodgson, A. Hermann, D. Shallin Busch, P. McElhany, T. Essington, C. J. Harvey, and E. A. Fulton. 2017. Risks of ocean acidification in the California Current food web and fisheries: Ecosystem model projections. Global Change Biol. 23:1525-1539.

Miller, B. and S. Sadro. 2003. Residence time and seasonal movements of juvenile coho salmon in the ecotone and lower estuary of Winchester Creek, South Slough, Oregon. Trans. Am. Fish. Soc. 132:546–559.

Morris, J. F. T., M. Trudel, M. E. Thiess, R. M. Seeting, J. Fisher, S. A. Hinton, E. Ferguson, J. A. Orsi, E. Farley, and D. W. Welch. 2007. Stock-specific migrations of juvenile Coho salmon derived from coded-wire tag recoveries on the continental shelf of western North America. Am. Fish. Soc. Symp. 57:81-104.

Pess, G. R., D. R. Montgomery, E. A. Steel, R. E. Bilby, B. E. Feist, and H. M. Greenberg. 2002. Landscape characteristics, land use, and coho salmon (*Oncorhynchus kisutch*) abundance, Snohomish River, Wash., U.S.A. Can. J. Fish. Aquat. Sci. 59:613-623.

Quinn, T. P., L. A. Vollestad, J. Peterson, and V. Gallucci. 2004. Influences of freshwater and marine growth on the egg size-egg number tradeoff in Coho and Chinook salmon. Trans. Am. Fish. Soc. 133:55-65.

Scholz, N. L., M. S. Myers, S. G. McCarthy, J. S. Labenia, J. K. McIntyre, G. M. Ylitalo, L. D. Rhodes, C. A. Laetz, C. M. Stehr, B. L. French, B. McMillan, D. Wilson, L. Reed, K. D. Lynch, S. Damm, J. W. Davis, and T. K. Collier. 2011. Recurrent die-offs of adult coho salmon returning to spawn in Puget Sound lowland urban streams. PLoS ONE 6:e28013.

Snover, A. K., P. W. Mote, L. Whitely Binder, A. F. Hamlet, and M. N.J. 2005. Climate change and its effects on Puget Sound. A report for the Puget Sound Action Team by the Climate Impacts Group, Center for Science in the Earth System, Joint Institute for the Study of the Atmosphere and Oceans, University of Washington, Seattle.

Sweeting, R., and R. J. Beamish. 2009. A Comparison of the Diets of Hatchery and Wild Coho Salmon (*Oncorhynchus kisutch*) in the Strait of Georgia from 1997–2007. N. Pac. Anad. Fish Comm. Bull. 5:255-264.

Van Doornik, D. M., D. J. Teel, D. R. Kuligowski, C. A. Morgan, and E. Casillas. 2007. Genetic analyses provide insight into the early ocean stock distribution and survival of juvenile Coho salmon off the coasts of Washington and Oregon. N. Am. J. Fish. Manage. 27:220-237.

Weitkamp, L. A., T. C. Wainwright, G. J. Bryant, G. B. Milner, D. J. Teel, R. G. Kope, and R. S. Waples. 1995. Status review of coho salmon from Washington, Oregon, and California. U.S. Dep. Commerce NOAA Tech. Memo. NMFS-NWFSC-24.

Zimmerman, M. S., J. R. Irvine, M. O'Neill, J. H. Anderson, C. M. Greene, J. Weinheimer, M. Trudel, and K. Rawson. 2015a. Spatial and temporal patterns in smolt survival of wild and hatchery coho salmon in the Salish Sea. Mar. Coast. Fish. 7:116-134.

Zimmerman, M. S., C. Kinsel, E. Beamer, E. J. Connor, and D. E. Pflug. 2015b. Abundance, survival, and life history strategies of juvenile Chinook salmon in the Skagit River, Washington. Trans. Am. Fish. Soc. 144:627-641.

Chum Salmon (*Oncorhynchus keta*)

Columbia River chum

Overall vulnerability—Moderate (3% Low, 97% Moderate)
Biological sensitivity—Moderate (2% Low, 97% Moderate, 1% High)
Climate exposure—Moderate (85% Moderate, 15% High)
Adaptive capacity—Moderate (1.9)
Data quality—84% of scores ≥ 2

	Columbia River Chum	Expert Scores	Data Quality	Expert Scores Plots (Portion by Category)
Sensitivity attributes	Early life history	1.6	2.8	
	Juvenile freshwater stage	1.4	2.8	
	Estuary stage	2.4	2.8	
	Marine stage	2.1	1.8	
	Adult freshwater stage	1.6	3	
	Cumulative life-cycle effects	2.7	2.3	
	Hatchery influence	1.5	2.8	
	Other stressors	2.5	2.3	
	Population viability	3	3	
	Ocean acidification sensitivity	1.9	1	
	Sensitivity Score	Moderate		
Exposure variables	Stream temperature	2.2	2.8	
	Summer water deficit	1.5	2	
	Flooding	1.5	2	
	Hydrologic regime	2.1	2.8	
	Sea level rise	2.7	2	
	Sea surface temperature	2.8	2.5	
	Ocean acidification exposure	3.9	3	
	Upwelling	1.8	2	
	Ocean currents	1.9	1	
	Exposure Score	Moderate		
Overall Vulnerability Rank		Moderate		

Low
Moderate
High
Very High

Life History Synopsis

Columbia River chum salmon adults enter fresh water at an advanced state of maturation and begin spawning in November in tributaries closest to the Columbia River mouth, such as Grays River and Big Creek. Later in the season, adults progress to tributaries farther upstream, such as Hamilton and Hardy Creek, although spawning is restricted to areas below Bonneville Dam (Johnson et al. 1997). Columbia River chum usually spawns in alluvial fans and in the lower reaches of medium and large streams (Salo 1991; Myers et al. 2006). Chum has been observed spawning just above the level

of saltwater intrusion and tidal influence. A remnant summer-run chum salmon population still exists in the Cowlitz River. Although information is limited, these fish enter the river earlier, migrate relatively farther upstream to pass Mayfield Dam (rkm 80), and spawn earlier than other Columbia River chum populations. Chum will also seek out areas of groundwater seeps (Bakkala 1970; Salo 1991). For example, there are a number of spawning areas in the mainstem Columbia River where groundwater percolates through the gravel (Rawding and Hillson 2003). Finally, chum tends to spawn in large aggregations, possibly to improve juvenile survival through predator swamping.

Juveniles migrate to the ocean soon after emergence, and may spend weeks in the estuary and Columbia River plume (Salo 1991; Johnson et al. 1997). Thus, growth and survival during the first year is less dependent on freshwater than on estuarine conditions relative to other salmonids. In the estuary, juvenile chum feed on copepods and gammarid amphipods (Salo 1991). Once in the ocean, juveniles grow quickly and begin moving along the coast in a northerly direction (Weitkamp et al. 2012). Chum salmon typically has a high seas distribution, and fish from Washington rear from the Gulf of Alaska west to the International Date Line and north into the Bering Sea (Urawa et al. 2018). Although there have been relatively few studies, tagged Columbia River chum have been recovered in fisheries off of British Columbia and Southeast Alaska, with the majority of fish returning at age-4- and age-5. In contrast to other salmon, chum salmon spend most of their lives in the ocean and little time in fresh water, either as juveniles or adults.

Climate Effects on Abundance and Distribution

Given the late-autumn return and spawn timing of Columbia River chum, temperatures under climate change scenarios may not be limiting for adult prespawn survival or early life history. Furthermore, the preferential spawning in areas with groundwater seeps provides relatively constant incubation conditions and would moderate somewhat the effect from changes in temperature and precipitation. For chum that spawn in the lowermost reaches of Columbia River tributaries, sea level changes could result in an expansion of areas influenced by saltwater intrusion or tidal (slack water) influence.

Estuary and ocean temperature conditions may change more rapidly than incubation conditions, especially at groundwater seeps, and such changes could leave juvenile migrants “out-of-sync” with nursery conditions. Accordingly, Columbia River chum ranked ***moderate*** in sensitivity to *cumulative life-cycle effects*. The small size of juvenile emergent chum migrating to the estuary makes them especially vulnerable to changing conditions in the lower river and estuary as well. For example, the quantity, type, and timing of zooplankton that juvenile chum feed upon while rearing in the estuary and nearshore environs may be dramatically altered under climate change, especially due to *ocean acidification*. It is during this early ocean entry period that chum salmon are most vulnerable to alterations in their environment.

Extrinsic Factors

Columbia River chum is listed as threatened under the U.S. Endangered Species Act, and this DPS ranked ***moderate*** in sensitivity for *population viability*, but ***low*** in other external sensitivity attributes, such as *other stressors* and *hatchery influence*.

Adaptive Capacity

Columbia River chum ranked ***moderate*** in *adaptive capacity*.

Literature Cited

Bakkala, R. G. 1970. Synopsis of the biological data on chum salmon, *Oncorhynchus kéta* (Walbaum) 1792. U.S. Fish Wildl. Serv. Circ. 315.

Johnson, O. W., S. W. Grant, R. G. Kope, K. Neely, and F. W. Waknitz. 1997. Status review of chum salmon from Washington, Oregon, and California. U.S. Dep. Commerce NOAA Tech. Memo. NMFS-NWFSC-32.

Myers, J., C. Busack, D. Rawding, A. Marshall, D. J. Teel, D. M. Van Doornik, and M. Maher. 2006. Historical population structure of Pacific salmonids in the Willamette River and Lower Columbia River Basins. U.S. Dep. Commerce NOAA Tech. Memo. NMFS-NWFSC-73.

Rawding, D., and T. Hillson. 2003. Population estimates for chum salmon spawning in the mainstem Columbia River. Bonneville Power Administration report DOE/BP-00007373-3, Portland, OR.

Salo, E. O. 1991. Life history of chum salmon (*Oncorhynchus keta*). Page 564 *in* C. Groot and L. Margolis, editors. Pacific salmon life histories. University of British Columbia Press, Vancouver, B.C., Canada.

Urawa, S., T. D. Beacham, M. Fukuwaka, and Kaeriyama. 2018. Ocean ecology of chum salmon. Pages 161-317 *in* R. J. Beamish, editor. The ocean ecology of Pacific salmon and trout. American Fisheries Society, Bethesda.

Weitkamp, L. A., E. Daly, and J. Fisher. 2012. Early marine residence of juvenile pink and chum salmon in the northern California Current: life at the southern end of the range. N. Pac. Anad. Fish Comm. Tech. Rep. 8:54-57

Hood Canal summer-run chum

Overall vulnerability—High (31% Moderate, 69% High)
Biological sensitivity—High (27% Moderate, 73% High)
Climate exposure—High (5% Moderate, 95% High)
Adaptive capacity—Moderate (1.7)
Data quality—84% of scores ≥ 2

	Hood Canal summer-run Chum	Expert Scores	Data Quality	Expert Scores Plots (Portion by Category)
Sensitivity attributes	Early life history	2.8	2.8	
	Juvenile freshwater stage	1.4	2.8	
	Estuary stage	2.5	2.8	
	Marine stage	2.1	1.8	
	Adult freshwater stage	2.8	3	
	Cumulative life-cycle effects	3.3	2.3	
	Hatchery influence	2.2	2	
	Other stressors	2.3	2.5	
	Population viability	3.1	3	
	Ocean acidification sensitivity	1.9	1	
	Sensitivity Score	High		
Exposure variables	Stream temperature	3.1	2.8	
	Summer water deficit	3.1	2	
	Flooding	2	2	
	Hydrologic regime	1.3	2.8	
	Sea level rise	2.8	2	
	Sea surface temperature	2.8	2.5	
	Ocean acidification exposure	3.9	3	
	Upwelling	1.6	2	
	Ocean currents	1.8	1	
	Exposure Score	High		
Overall Vulnerability Rank		High		

Low
Moderate
High
Very High

Life History Synopsis

Adult migration begins in late summer for Hood Canal summer-run chum, with relatively short distances to spawning grounds (<1-60 km). Adults spawn in both large and small river systems, and therefore have access to a broad array of habitats that support spawning. Chum salmon adults typically spawn in the lower reaches of rivers and in side channels and riffles. Eggs incubate in late summer and fall, when temperatures may approach stressful thresholds. High fall river flows increase bed load sediment, which may lead to scouring of redds. Juveniles enter the ocean during the first spring as fry or subyearlings after spending up to one month in fresh water. This short period of freshwater rearing occurs during winter months, when temperature variation is low. In late winter, juvenile chum can spend up to one month in shallow estuarine waters

(all salinity zones) before moving to the ocean. After leaving estuaries, juveniles may exhibit extended residency within Puget Sound before migrating, and may even overwinter in the sound (Salo 1991; Johnson et al. 1997). Juveniles in the ocean move northward along nearshore areas to Alaska.

Chum salmon consume a wider variety of prey than other Pacific salmon species (Davis et al. 2009), and the diet of these fish may confer greater resiliency on this species in confronting impending ecosystem transitions. Chum salmon are micronectivores, zooplanktivores, and piscivores. In general, North American chum populations undergo extensive ocean migrations into the Gulf of Alaska and subarctic North Pacific Ocean (Urawa et al. 2018). Chum has been estimated to tolerate an absolute thermal range of 0-15.6°C in all seasons and frequently observed ranges of 1-13°C during spring to fall and 1.5-10°C during winter (Abdul-Aziz et al. 2011). Due to a lack of extensive marking efforts, the ocean distribution of Hood Canal summer-run chum is not as well known.

Age at maturity is highly variable (age 3, 4, and 5). Adults may aggregate in estuaries near river mouths for up to one month prior to upstream migration and may be stressed by warm temperatures or hypoxia at this time.

Climate Effects on Abundance and Distribution

A relatively small number of studies have examined the effect of climate factors on abundance, distribution, or productivity of West Coast chum salmon. Eaton and Scheller (1996) found a maximum weekly average upper thermal tolerance for chum salmon of 21°C. Early marine climate signals such as coastal sea surface temperature and PDO explain a small proportion of the variation in total productivity for chum salmon off Washington and the west coast of Vancouver Island. Increased productivity is associated with warmer coastal SSTs (and a positive PDO) a few months prior to and during the early marine period (Mueter et al. 2005). Accordingly, this DPS ranked ***low*** in sensitivity at the *marine stage*.

Mantua et al. (2010) suggested that the unique life history of Hood Canal summer-run chum makes this DPS especially vulnerable to the climate change impacts because adults spawn in small shallow streams during late summer, eggs incubate in fall and early winter, and fry migrate to sea in late winter. This DPS ranked ***moderate*** in sensitivity during the *adult freshwater stage* and *early life history*. For the low-elevation Hood Canal streams historically used by summer chum, predicted climate change effects include multiple negative impacts. Such impacts stem from higher water temperatures and reduced streamflow in summer and the potential for increased redd scour from peak flows of greater magnitude in fall and winter. Thus, Hood Canal summer-run chum ranked ***high*** in exposure to both *stream temperature* and *summer water deficit*, largely due to effects on returning adults and hatched fry. Likewise, sensitivity to *cumulative life-cycle effects* was ranked ***high*** for this DPS.

Abdul-Aziz et al. (2011) developed spatially explicit representations of open ocean thermal habitat for chum salmon. They found that under a multimodel ensemble average of climate model outputs using the A1B emissions scenario, summer habitat area for chum salmon declined by 29% for the 2080s, with the largest habitat losses in the eastern half of the Gulf of Alaska. Wintertime habitat area losses were 19%, with reductions at the southern end of the historical range offset somewhat by habitat area gains in the southern Bering Sea. Whether a general northward and westward displacement of the most frequently observed thermal open ocean habitat will have substantial impacts on the life-cycle productivity or spawning distribution of chum salmon is unknown. However, West Coast chum salmon populations are likely vulnerable to the projected displacement of high seas thermal habitat. Sensitivity in the *marine stage* was ranked ***low*** for Hood Canal summer-run chum, but exposure to mean *sea surface temperature* ranked ***moderate***.

Juvenile chum consume crustaceans, including amphipods and copepods, which may be impacted by ocean acidification. However, chum is known to consume jellyfish (Arai et al. 2003), which might be more resilient to OA than crustaceans. Jellyfish become more abundant in warmer years and may offer food sources to juvenile chum that are not taken advantage of by other species. Puget Sound chum salmon appear to compete with pink salmon, so climate effects on pink will indirectly affect chum (Ruggerone and Nielsen 2004; Greene et al. 2015).

Extrinsic Factors

Hood Canal summer-run chum salmon is listed as threatened under the U.S. Endangered Species Act, and a 2011 status review update suggested further decline in some populations (Ford et al. 2011). This DPS ranked ***high*** in sensitivity for *population viability*, but ***low*** in sensitivity to *hatchery influence* and *other stressors*.

Adaptive Capacity

Summer run Hood Canal chum salmon ranked ***moderate*** for adaptive capacity.

Literature Cited

Abdul-Aziz, O. I., N. J. Mantua, and K. W. Myers. 2011. Potential climate change impacts on thermal habitats of Pacific salmon (*Oncorhynchus spp.*) in the North Pacific Ocean and adjacent seas. Can. J. Fish. Aquat. Sci. 68(9):1660-1680.

Arai, M. N., D. W. Welch, A. L. Dunsmuir, M. C. Jacobs, and A. R. Ladouceur. 2003. Digestion of pelagic Ctenophora and Cnidaria by fish. Can. J. Fish. Aquat. Sci. 60:825-829.

Davis, N. D., A. V. Volkov, A. Y. Efimkin, N. A. Kuznetsova, J. L. Armstrong, and O. Sakai. 2009. Review of BASIS salmon food habits studies. N. Pac. Anad. Fish Comm. Bull. 5:197-208.

Eaton, J. and R. Scheller. 1996. Effects of climate warming on fish thermal habitat in streams of the United States. Limnol. Oceanogr. 41:109-115.

Ford, M. J., A. Albaugh, K. Barnas, T. Cooney, J. Cowen, J. J. Hard, R. G. Kope, M. M. McClure, P. McElhany, J. M. Myers, N. J. Sands, D. J. Teel, and L. A. Weitkamp. 2011. Status review update for Pacific salmon and steelhead listed under the Endangered Species Act: Pacific Northwest. U.S. Dep. Commerce NOAA Tech. Memo. NMFS-NWFSC-113.

Greene, C. M., L. Kuehne, C. A. Rice, K. L. Fresh, and D. Penttila. 2015. Forty years of change in forage fish and jellyfish abundance across greater Puget Sound, Washington (USA): anthropogenic and climate associations. Mar. Ecol. Prog. Ser. 525:153-170.

Johnson, O. W., S. W. Grant, R. G. Kope, K. Neely, and F. W. Waknitz. 1997. Status review of chum salmon from Washington, Oregon, and California. U.S. Dep. Commerce NOAA Tech. Memo. NMFS-NWFSC-32.

Mantua, N. J., I. Tohver, and A. F. Hamlet. 2010. Climate change impacts on streamflow extremes and summertime stream temperature Clim. Change 102:187-223.

Mueter, F. J., B. J. Pyper, and R. M. Peterman. 2005. Relationships between coastal ocean conditions and survival rates of northeast Pacific salmon at multiple lags. Trans. Am. Fish. Soc. 134:105-119.

Ruggerone, G. T., and J. L. Nielsen. 2004. Evidence for competitive dominance of Pink salmon (*Oncorhynchus gorbuscha*) over other Salmonids in the North Pacific Ocean. Rev Fish Biol Fish 14:371-390.

Salo, E. O. 1991. Life history of chum salmon (*Oncorhynchus keta*). Page 233 *in* L. Groot and C. Margolis, editors. Pacific salmon life histories. UBC Press, Vancouver, British Columbia, Canada.

Urawa, S., T. D. Beacham, M. Fukuwaka, and Kaeriyama. 2018. Ocean ecology of chum salmon. Pages 161-317 *in* R. J. Beamish, editor. The ocean ecology of Pacific salmon and trout. American Fisheries Society, Bethesda.

Puget Sound chum

Overall vulnerability—Moderate (32% Low, 68% Moderate)
Biological sensitivity—Moderate (32% Low, 68% Moderate)
Climate exposure—Moderate (47% Moderate, 53% High)
Adaptive capacity—Moderate (1.9)
Data quality—84% of scores ≥ 2

	Puget Sound Chum	Expert Scores	Data Quality	Expert Scores Plots (Portion by Category)
Sensitivity attributes	Early life history	1.7	2.8	
	Juvenile freshwater stage	1.4	2.8	
	Estuary stage	2.5	2.8	
	Marine stage	2.2	1.8	
	Adult freshwater stage	1.6	3	
	Cumulative life-cycle effects	2.3	2.3	
	Hatchery influence	1.8	2.3	
	Other stressors	3	2.5	
	Population viability	1.8	3	
	Ocean acidification sensitivity	1.9	1	
	Sensitivity Score	Moderate		
Exposure variables	Stream temperature	2	2.8	
	Summer water deficit	1.6	2	
	Flooding	1.8	2	
	Hydrologic regime	2.9	2.8	
	Sea level rise	2.8	2	
	Sea surface temperature	2.9	2.5	
	Ocean acidification exposure	3.9	3	
	Upwelling	1.5	2	
	Ocean currents	1.8	1	
	Exposure Score	Moderate		
Overall Vulnerability Rank		Moderate		

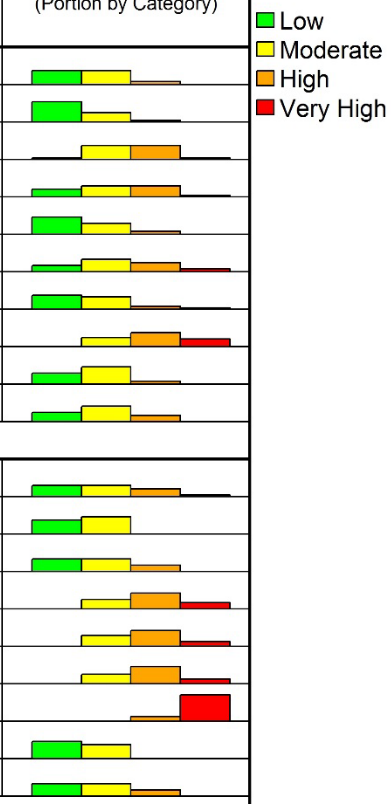

Life History Synopsis

Adult Puget Sound chum migrate relatively short distances to spawning grounds (1-60 km). Migration begins in late summer, when adults may encounter low flows and relatively high stream temperatures. Puget Sound chum spawns in both large and small river systems, and therefore has access to a broad array of habitat areas that support spawning. Chum typically spawns in the lower reaches of rivers and in side-channels and riffles. Incubation occurs in the cool, wet months of fall and winter, when temperatures are generally unlikely to approach stressful thresholds for eggs. Juveniles enter the ocean during the first spring as fry or subyearlings, spending up to one month in fresh water. This short period of freshwater rearing occurs during winter months when temperature variation is low. During late winter, juveniles can spend up to one month in shallow

estuarine waters of all salinity zones before moving to the ocean. After leaving estuaries, juveniles may exhibit extended residency within Puget Sound before migrating and may even overwinter in the sound. In the ocean, juveniles move northward along nearshore areas to Alaska (Johnson et al. 1997). Age at maturity is highly variable (typically age 3, 4, or 5).

In general, West Coast chum salmon undergo extensive ocean migrations into the Gulf of Alaska and subarctic North Pacific Ocean (Urawa et al. 2018). West Coast chum populations are estimated to tolerate an absolute thermal range of 0-15.6°C in all seasons, and a frequently observed range of 1-13°C during spring-fall and 1.5-10°C during winter (Abdul-Aziz et al. 2011). In marine environments juvenile chum consume crustaceans, including amphipods and copepods, which may be impacted by ocean acidification. Chum salmon consumes a greater variety of prey than other salmon species (Davis et al. 2009), including gelatinous zooplankton (Arai et al. 2003).

Climate Effects on Abundance and Distribution

Chum salmon adults may aggregate in estuaries near river mouths for up to one month prior to migration and may be stressed by warm temperatures or hypoxia at this time.

A relatively small number of studies have examined the effects of climate impacts on abundance, distribution, or productivity of West Coast chum salmon. Eaton and Scheller (1996) found that the maximum weekly average upper thermal tolerance for chum salmon was 21°C. Exposure to summer *stream temperature* and *hydrologic regime* shift was a concern, largely hatched fry and adults returning to rivers. Nevertheless, Puget Sound chum ranked ***low*** for both of these exposure attributes. Flow conditions may be highly variable, and are projected to become more extreme with climate change. High fall river flows increase bed load sediment, which may lead to redd scour. Sea level rise and potential storm surge risks pose the greatest climate-related stressors during estuary rearing, although this DPS ranked ***low*** in sensitivity at the *estuary stage*.

Early marine climate signals such as coastal SST and PDO may explain a small proportion of variation in the total productivity of chum salmon off Washington and the west coast of Vancouver Island, with increased productivity associated with warmer coastal SSTs (and positive PDO) a few months prior to and during the early marine period (Mueter et al. 2005).

Abdul-Aziz et al. (2011) developed spatially explicit representations of open ocean thermal habitat for North American chum salmon, using a multimodel ensemble average of climate model outputs under the A1B emissions scenario. They projected a decline in summer habitat area for chum salmon of 29% by the 2080s, with the largest habitat losses in the eastern half of the Gulf of Alaska. Wintertime habitat area losses

were 19%, with reductions at the southern end of the historical range offset somewhat by habitat area gains in the southern Bering Sea.

Whether a general northward and westward displacement of the most frequently observed thermal open ocean habitat will have substantial impacts on life-cycle productivity or spawning distribution for chum salmon is unknown. However, West Coast chum salmon populations would likely be vulnerable to projected displacements of high seas thermal habitat. Puget Sound chum ranked ***moderate*** in exposure to mean *sea surface temperature* and ***low*** in sensitivity at the *marine stage*.

Increasing abundance of jellyfish in warmer years may proffer an advantage to juvenile chum in availability of food sources not usable to other salmon (Arai et al. 2003). Nevertheless, Puget Sound chum appears to compete with pink salmon, so climate impacts to pink salmon could indirectly affect this DPS (Ruggerone and Nielsen 2004; Greene et al. 2015). There may be linkages between declining adult biomass, egg size, and juvenile size at migration, that lead to life-cycle based shifts in total mortality. Size trends are negative over time for many chum populations. These patterns may result in part from thermal regimes during incubation or marine growth (Johnson et al. 1997).

Extrinsic Factors

Some regions of Puget Sound are experiencing losses in primary production and increases in eutrophication, possibly due to nutrient inputs. These can be exacerbated by higher temperatures and increased stratification, and could impact fish via hypoxia and losses in secondary production (Moore et al. 2008). However, sensitivity to *other stressors* (such as pollutants) was ranked ***low*** for Puget Sound chum.

Fall run Puget Sound chum is not presently listed under the U.S. Endangered Species Act. In some areas of the sound, chum run sizes are increasing, while in others they have declined precipitously. This DPS ranked ***moderate*** in *population viability*.

Adaptive Capacity

Puget Sound chum ranked ***moderate*** in adaptive capacity.

Literature Cited

Abdul-Aziz, O. I., N. J. Mantua, and K. W. Myers. 2011. Potential climate change impacts on thermal habitats of Pacific salmon (*Oncorhynchus spp.*) in the North Pacific Ocean and adjacent seas. Can. J. Fish. Aquat. Sci. 68(9)1660-1680.

Arai, M. N., D. W. Welch, A. L. Dunsmuir, M. C. Jacobs, and A. R. Ladouceur. 2003. Digestion of pelagic Ctenophora and Cnidaria by fish. Can. J. Fish. Aquat. Sci. 60:825-829.

Davis, N. D., A. V. Volkov, A. Y. Efimkin, N. A. Kuznetsova, J. L. Armstrong, and O. Sakai. 2009. Review of BASIS salmon food habits studies. N. Pac. Anad. Fish Comm. Bull. 5:197-208.

Eaton, J. and R. Scheller. 1996. Effects of climate warming on fish thermal habitat in streams of the United States. Limnol. Oceanogr. 41:109-115.

Greene, C. M., L. Kuehne, C. A. Rice, K. L. Fresh, and D. Penttila. 2015. Forty years of change in forage fish and jellyfish abundance across greater Puget Sound, Washington (USA): anthropogenic and climate associations. Mar. Ecol. Prog. Ser. 525:153-170.

Johnson, O. W., S. W. Grant, R. G. Kope, K. Neely, and F. W. Waknitz. 1997. Status review of chum salmon from Washington, Oregon, and California. U.S. Dep. Commerce NOAA Tech. Memo. NMFS-NWFSC-32.

Moore, S. K., N. J. Mantua, J. A. Newton, M. Kawase, M. J. Warner, and J. P. Kellogg. 2008. A descriptive analysis of temporal and spatial patterns of variability in Puget Sound oceanographic properties. Est. Coast. Shelf Sci. 80:545-554.

Mueter, F. J., B. J. Pyper, and R. M. Peterman. 2005. Relationships between coastal ocean conditions and survival rates of northeast Pacific salmon at multiple lags. Trans. Am. Fish. Soc. 134:105-119.

Ruggerone, G. T. and J. L. Nielsen. 2004. Evidence for competitive dominance of Pink salmon (*Oncorhynchus gorbuscha*) over other Salmonids in the North Pacific Ocean. Rev. Fish Biol. Fish. 14:371-390.

Urawa, S., T. D. Beacham, M. Fukuwaka, and Kaeriyama. 2018. Ocean ecology of chum salmon. Pages 161-317 *in* R. J. Beamish, editor. The ocean ecology of Pacific salmon and trout. American Fisheries Society, Bethesda.

Pink Salmon (*Oncorhynchus keta*)

Puget Sound pink (odd- and even-year pink combined)

Overall vulnerability—Moderate (30% Low, 70% Moderate)
Biological sensitivity—Moderate (30% Low, 70% Moderate)
Climate exposure –Moderate (68% Moderate, 31% High)
Adaptive capacity—Moderate (2.0)
Data quality—89% of scores ≥ 2

	Puget Sound Pink	Expert Scores	Data Quality	Expert Scores Plots (Portion by Category)
Sensitivity attributes	Early life history	2.8	2.8	
	Juvenile freshwater stage	1.4	2.8	
	Estuary stage	2.4	2.8	
	Marine stage	2.3	2.3	
	Adult freshwater stage	2	3	
	Cumulative life-cycle effects	2.3	2.3	
	Hatchery influence	1.4	2.8	
	Other stressors	2.4	2.8	
	Population viability	1.3	3	
	Ocean acidification sensitivity	2.4	1	
	Sensitivity Score	Low		
Exposure variables	Stream temperature	2.1	2.8	
	Summer water deficit	1.9	2	
	Flooding	1.7	2	
	Hydrologic regime	2.5	2.8	
	Sea level rise	2.8	2	
	Sea surface temperature	2.9	2.5	
	Ocean acidification exposure	3.9	3	
	Upwelling	1.5	2	
	Ocean currents	2.1	1	
	Exposure Score	Moderate		
Overall Vulnerability Rank		Low		

Low
Moderate
High
Very High

Life History Synopsis

Puget Sound pink salmon adults migrate a relatively short distance to spawning grounds (30-150 km), mostly during summer or early fall. Pink juveniles enter Puget Sound as age-0 fry and rear in the sound briefly, rarely residing through summer (Heard 1991). Most spend limited time in estuaries that are not constrained by temperature or flow (Heard 1991). Migration out of Puget Sound begins in summer, and along with pink salmon from other Salish Sea populations, this DPS migrates slowly northward along the continental shelf, reaching northern British Columbia by fall before heading offshore into the Gulf of Alaska (Radchenko et al. 2018). Pink salmon returns as a mature adult after

one winter at sea (Heard 1991; Ruggerone and Goetz 2004). Some regions of Puget Sound are experiencing losses in primary production and increases in eutrophication, possibly due to nutrient inputs. These losses can be exacerbated by higher temperatures and increased stratification, and could impact fish in this DPS via hypoxia and losses in secondary production (Moore et al. 2008; Mauger et al. 2015).

Climate Effects on Abundance and Distribution

We know of no studies that have examined the effect of climate impacts on productivity of Puget Sound pink, but a few have examined pink salmon marine survival with regard to climate stressors. During early marine residence, climate signals such as temperature, El Niño, and PDO explained a large proportion of variation in total return rates (Mantua et al. 1997, Ruggerone and Goetz 2004). In freshwater, warmer winters would likely result in shorter incubation periods and higher flows during emergence, which would likely increase sedimentation and result in higher egg-to-fry mortality (Myers et al. 1998; Zimmerman et al. 2015). For Puget Sound Pink salmon, *early life history* (egg incubation stage) was the only sensitivity attribute ranked ***moderate***.

Puget Sound pink ranked ***moderate*** in exposure attributes overall, with three factors contributing to that ranking: *hydrological regime*, mean *sea surface temperature* and *sea level rise*.

Summer temperature barriers exist in some lowland river systems, and these are likely to expand (Heard 1991). In Puget Sound, summer and fall temperatures in a minority of watersheds may already be near the maximum threshold. Nonetheless, stream temperatures in this region are expected to change somewhat less than in other regions.

Juveniles consume marine arthropods including copepods, which may be impacted by ocean acidification. A major component of pink salmon diets is pteropods (Armstrong et al. 2005), for which there is strong evidence of impacts from ocean acidification (Lischka and Riebesell 2012). How readily pink salmon might switch prey remains unclear (Kaczynski et al. 1973; Godin 1981; Armstrong et al. 2008). Furthermore, pink is the only Pacific salmon species with demonstrated developmental sensitivity to low pH (Ou et al. 2015).

Extrinsic Factors

Some regions of Puget Sound are experiencing losses in primary production and increases in eutrophication, possibly due to nutrient inputs. These can be exacerbated by higher temperatures and increased stratification, and could impact fish via hypoxia and losses in secondary production. In addition, when high flow events are preceded by extended dry months, non-point source pollutants have caused pre-spawn, egg, and juvenile mortality (Scholz et al. 2011).

Adaptive Capacity

Puget Sound pink salmon ranked ***moderate*** for adaptive capacity.

Literature Cited

Armstrong, J. L., J. L. Boldt, A. D. Cross, J. H. Moss, N. D. Davis, J. W. Myers, R. V. Walker, D. A. Beachamp, and L. J. Haldorson. 2005. Distribution, size, and interannual, seasonal and diel food habits of northern Gulf of Alaska juvenile pink salmon, *Oncorhynchus gorbuscha*. Deep Sea Res. II 52:247-265.

Armstrong, J. L., K. W. Myers, D. A. Beauchamp, N. D. Davis, R. V. Walker, J. L. Boldt, J. J. Piccolo, L. J. Haldorson, and J. H. Moss. 2008. Interannual and spatial feeding patterns of hatchery and wild juvenile pink salmon in the Gulf of Alaska in years of low and high survival. Trans. Am. Fish. Soc. 137:1299-1316.

Godin, J. J. 1981. Daily patterns of feeding behavior, daily rations, and diets of juvenile pink salmon (*Oncorhynchus gorbuscha*) in two marine bays of British Columbia. Can. J. Fish. Aquat. Sci. 38:10-15.

Heard, W. R. 1991. Life history of pink salmon (*Oncorhynchus gorbuscha*).*in* C. Groot and L. Margolis, editors. Pacific salmon life histories. Columbia Press, Vancouver, B.C., Canada.

Kaczynski, V. W., R. J. Feller, J. Clayton, and R. J. Gerke. 1973. Trophic analysis of juvenile pink and chum salmon (*Oncorhynchus gorbuscha* and *O. keta*) in Puget Sound. J. Fish. Res. Board Can. 30:1003-1008.

Lischka, S. and U. Riebesell. 2012. Synergistic effects of ocean acidification and warming on overwintering pteropods in the Arctic. Global Change Biol 18:3517-3528.

Mantua, N. J., S. R. Hare, Y. Zhang, J. M. Wallace, and R. C. Francis. 1997. A Pacific interdecadal climate oscillation with impacts on salmon production. Bull. Am. Meteorol. Soc. 78:1069-1079.

Mauger, G. S., J. H. Casola, H. A. Morgan, R. L. Strauch, B. Jones, B. Curry, T. M. Busch Isaksen, L. Whitely Binder, B. Krosby, and A. K. Snover. 2015. State of knowledge: Climate change in Puget Sound. Report to the Puget Sound Partnership and National Oceanic and Atmospheric Administration. Climate Impacts Group, University of Washington, Seattle.

Moore, S. K., N. J. Mantua, J. A. Newton, M. Kawase, M. J. Warner, and J. P. Kellogg. 2008. A descriptive analysis of temporal and spatial patterns of variability in Puget Sound oceanographic properties. Est. Coast. Shelf Sci. 80:545-554.

Myers, J. M., R. G. Kope, G. J. Bryant, D. Teel, L. J. Lierheimer, T. C. Wainwright, S. W. Grant, W. F. Waknitz, K. Neely, S. T. Lindley, and R. S. Waples. 1998. Status review of Chinook salmon from Washington, Idaho, Oregon, and California. U.S. Dep. Commerce NOAA Tech. Memo. NMFS-NWFSC-35.

Ou, M., T. J. Hamilton, J. Eom, E. M. Lyall, J. Gallup, A. Jiang, J. Lee, D. A. Close, S.-S. Yun, and C. J. Brauner. 2015. Responses of pink salmon to CO2-induced aquatic acidification. Nature Clim. Change 5:950-955.

Radchenko, V. I., R. J. Beamish, W. R. Heard, and O. S. Termnykh. 2018. Ocean ecology of pink salmon.*in* R. J. Beamish, editor. The ocean ecology of Pacific salmon and trout. American Fisheries Society, Bethesda. MD.

Ruggerone, G. T. and F. A. Goetz. 2004. Survival of Puget Sound chinook salmon (*Oncorhynchus tshawytscha*) in response to climate-induced competition with pink salmon (*Oncorhynchus gorbuscha*). Can. J. Fish. Aquat. Sci. 61:1756-1770.

Scholz, N. L., M. S. Myers, S. G. McCarthy, J. S. Labenia, J. K. McIntyre, G. M. Ylitalo, L. D. Rhodes, C. A. Laetz, C. M. Stehr, B. L. French, B. McMillan, D. Wilson, L. Reed, K. D. Lynch, S. Damm, J. W. Davis, and T. K. Collier. 2011. Recurrent die-offs of adult coho salmon returning to spawn in Puget Sound lowland urban streams. PLoS ONE 6:e28013.

Zimmerman, M. S., C. Kinsel, E. Beamer, E. J. Connor, and D. E. Pflug. 2015. Abundance, survival, and life history strategies of juvenile Chinook salmon in the Skagit River, Washington. Trans. Am. Fish. Soc. 144:627-641.

Sockeye Salmon (*Oncorhynchus nerka*)

Snake River sockeye

Overall vulnerability—Very high (57% High, 43% Very high)
Biological sensitivity—Very high (91% moderate, 9% low)
Climate Exposure –High (99% High)
Adaptive capacity—Low (1.4)
Data quality—79% of scores > 2

	Snake River Sockeye	Expert Scores	Data Quality	Expert Scores Plots (Portion by Category)
Sensitivity attributes	Early life history	2.1	2.8	
	Juvenile freshwater stage	2.9	2.8	
	Estuary stage	1.7	2.3	
	Marine stage	2.4	2.3	
	Adult freshwater stage	3.5	2.8	
	Cumulative life-cycle effects	3.6	2	
	Hatchery influence	2	2.5	
	Other stressors	3.1	2.5	
	Population viability	3.7	2.5	
	Ocean acidification sensitivity	2.3	1.8	
	Sensitivity Score	Very High		
Exposure variables	Stream temperature	3.6	2.8	
	Summer water deficit	2.4	2	
	Flooding	2.8	1.8	
	Hydrologic regime	3	2.8	
	Sea level rise	1.3	2	
	Sea surface temperature	3.2	2.3	
	Ocean acidification exposure	3.9	3	
	Upwelling	1.7	1.8	
	Ocean currents	1.8	1	
	Exposure Score	High		
Overall Vulnerability Rank		Very High		

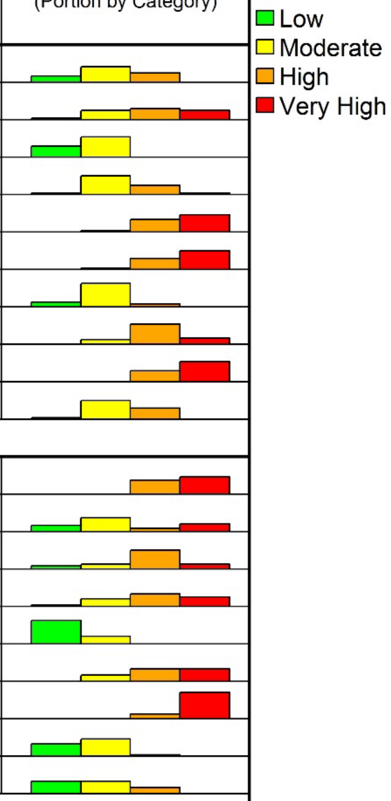

Life History Synopsis

Snake River sockeye has the longest freshwater migration (1,500 km) to the highest elevation (2,000 m above sea level) of any sockeye salmon DPS. Adults enter fresh water from mid-June to mid-July and typically arrive at Redfish Lake, Idaho, or nearby lakes in the second half of August. Adults reside in the relatively cool, deep, lake water until they spawn in October. Spawning occurs along edges of the lake and near the mouths of some tributaries. Natural-origin juveniles typically rear in lakes for one year, although a small proportion may stay longer. Smolts migrate rapidly with the spring

freshet and are thought to pass quickly through the Columbia River estuary. At present, population sizes are too low to be well-documented in marine sampling programs. However, individuals are thought to move relatively quickly northward toward the Gulf of Alaska (Tucker et al. 2015) to offshore feeding grounds (Farley et al. 2018). The majority of Redfish Lake sockeye adults return after 2 years at sea, although spawning migrations can occur after 1-6 years in the ocean (Crozier et al. 2015).

Climate Effects on Abundance and Distribution

Life-stage sensitivity attributes were high overall for this DPS, and its rank of ***very high*** sensitivity in the *adult freshwater stage* essentially caused the ***very high*** score for *cumulative life-cycle effects*. Rates of adult and juvenile migration survival are strongly correlated with temperature in the Columbia River, and catastrophic effects of temperature on the adult migration have been observed recently. Adult migration survival to spawning grounds ranged from 1% in the extremely warm year of 2015 to 60% in the more average year of 2010 (Crozier et al. 2015, 2018).

The anadromous component of the Redfish Lake population nearly disappeared altogether in the early 1990s, and has rebounded somewhat in recent years due to large releases of captive broodstock and improved ocean survival (Williams et al. 2014; Ford et al. 2015). Ocean survival is well-predicted by environmental indices, particularly upwelling and the Pacific Northwest Index (Williams et al. 2014). However, specific climate impacts on marine survival were uncertain, leading to a ***moderate*** score for this DPS at the *marine stage*.

Snake River sockeye ranked ***low*** in *estuary stage* sensitivity because of its rapid migration from fresh water to the early marine stage. Risk during *early life history* was also scored ***low*** because of the high elevation and relatively stable lake temperatures that influence the egg stage. Scores for the *juvenile freshwater stage* were spread across many bins (SD = 0.89) due to uncertainty in how juvenile rearing and migration would be affected by climate change. The primary rearing lake is likely to remain suitable for sockeye, but the long-distance migratory stage is sensitive to lower freshets that will result from reduced snowpack.

Because smolt production is now dependent on hatchery releases, there is great uncertainty in how management and fish condition will change in the future. Many juveniles are transported past the eight dams along their migration route, which improves juvenile survival but has negative effects on marine survival and adult migration success (Crozier et al. 2015, 2018). All of these anthropogenic influences make predictions about wild sockeye difficult.

In exposure attributes, this DPS scored ***very high*** for *stream temperature* and *ocean acidification* and ***high*** for *hydrologic regime* and *sea surface temperature*.

Extrinsic Factors

This DPS is listed as endangered under the U.S. Endangered Species Act, and natural production remains extremely low. Spatial diversity is limited to two main spawning areas within Redfish Lake, although some spawning occurs in other locations. Finally, genetic diversity has been maintained through careful captive breeding, but ultimately stems from very few anadromous individuals collected in the 1990s and a resident population that has continued to produce smolts. Thus, Snake River sockeye salmon scored ***very high*** for the *population viability* attribute.

Snake River sockeye also ranked ***high*** for the *other stressors* attribute, due mainly to the hydrosystem and its associated impacts on the migratory stage. Spawning and rearing habitat for this DPS is pristine in high-elevation wilderness area. Although historic mining and forestry has affected the spawning gravel in Redfish Lake, and sediment deposits in spawning areas have caused spawning locations to change over time, ongoing habitat threats are minimal.

This DPS has been totally dependent on a conservation hatchery using captive broodstock derived from a handful of Redfish Lake anadromous sockeye combined with residuals, or resident sockeye (Ford et al. 2015). A long-term sockeye reintroduction plan for the Stanley Basin is organized around three phases 1) genetic conservation of the natal stock, 2) lake recolonization and 3) promoting natural adaptation (Kline and Flagg 2014). A large-scale hatchery facility specifically designed to accommodate levels of production projected for phases 2 and 3 is now in operation. The score associated with *hatchery influence* for this DPS was ***moderate***. Best practices in maintaining this captive broodstock have proven that a conservation hatchery is capable of re-establishing an anadromous run, even after a devastating period of unsuccessful migration.

Adaptive Capacity

Snake River sockeye salmon scored ***low*** in adaptive capacity. Sockeye is unlikely to respond to climate change by changing its life history characteristics, other than reverting to a fully freshwater type. Such a reversion would constitute the complete loss of a fundamental characteristic of this DPS. It is possible that the resident population in Redfish Lake has already contributed significantly to the present anadromous broodstock.

Furthermore, little potential habitat exists that might improve in suitability. Low population abundance and spatial diversity suggest limited genetic heterogeneity that would support rapid adaptation. If anadromous adults are able to respond to natural selection, adult migration timing might shift to avoid high temperatures and low flows in summer, as has been observed in the larger, naturally reproducing Okanogan and Wenatchee sockeye DPSs (Crozier et al. 2011).

Literature Cited

Crozier, L. G., B. J. Burke, B. Sandford, G. Axel, and B. L. Sanderson. 2015. Passage and survival of adult Snake River sockeye salmon within and upstream from the Federal Columbia River Power System: 2014 Update. Report o the National Marine Fisheries Service Northwest Fisheries Science Center, Seattle, WA.

Crozier, L. G., M. D. Scheuerell, and R. W. Zabel. 2011. Using time series analysis to characterize evolutionary and plastic responses to environmental change: A case study of a shift toward earlier migration date in sockeye salmon. Am. Nat. 178:755-773.

Crozier, L. G., L. Wiesebron, J. E. Siegel, B. J. Burke, T. M. Marsh, B. P. Sandford, and D. Widener. 2018. Passage and survival of adult Snake River sockeye Salmon within and upstream from the Federal Columbia River Power System from 2008-2017. Report of the National Marine Fisheries Service, Northwest Fisheries Science Center, Seattle, WA.

Farley, E. V., T. D. Beacham, and A. V. Bugaev. 2018. Ocean ecology of sockeye salmon.*in* R. J. Beamish, editor. The ocean ecology of Pacific salmon and trout. American Fisheries Society, Bethesda.

Ford, M. J., K. Barnas, T. Cooney, L. G. Crozier, M. Diaz, J. J. Hard, E. E. Holmes, D. M. Holzer, R. G. Kope, P. W. Lawson, M. Liermann, J. M. Myers, M. Rowse, D. J. Teel, D. M. V. Doornik, T. C. Wainwright, L. A. Weitkamp, and M. Williams. 2015. Status review update for Pacific salmon and steelhead listed under the Endangered Species Act: Pacific Northwest. Report of the National Marine Fisheries Service, Northwest Fisheries Science Center, Seattle, WA. Available from www.nwfsc.noaa.gov/publications/scipubs/searchdoc.cfm.

Kline, P. A., and T. A. Flagg. 2014. Putting the red back in Redfish Lake, 20 years of progress toward saving the Pacific Northwest's most endangered salmon population. Fisheries 39:488-500.

Tucker, S., M. E. Thiess, J. F. T. Morris, D. Mackas, W. T. Peterson, J. R. Candy, T. D. Beacham, E. M. Iwamoto, D. J. Teel, M. Peterson, and M. Trudel. 2015. Coastal distribution and consequent factors influencing production of endangered Snake River sockeye salmon. Trans. Am. Fish. Soc. 144:107-123.

Williams, J. G., S. G. Smith, J. K. Fryer, M. D. Scheuerell, W. D. Muir, T. A. Flagg, R. W. Zabel, J. W. Ferguson, and E. Casillas. 2014. Influence of ocean and freshwater conditions on Columbia River sockeye salmon *Oncorhynchus nerka* adult return rates. Fish. Oceanogr. 23:210-224.

Lake Ozette sockeye

Bootstrap vulnerability—91% moderate, 9 % low
Bootstrap sensitivity—91% moderate, 9 % low
Bootstrap exposure—99% high
Adaptive capacity—Low (1.5)
Data quality—84% of scores ≥ 2

Lake Ozette Sockeye		Expert Scores	Data Quality	Expert Scores Plots (Portion by Category)
Sensitivity attributes	Early life history	2	2.8	
	Juvenile freshwater stage	2.3	2.8	
	Estuary stage	1.3	2.3	
	Marine stage	2.4	2	
	Adult freshwater stage	1.6	2.8	
	Cumulative life-cycle effects	1.9	2	
	Hatchery influence	1.8	2	
	Other stressors	2.7	2.5	
	Population viability	2.8	2.5	
	Ocean acidification sensitivity	2.3	1.8	
	Sensitivity Score	Moderate		
Exposure variables	Stream temperature	3.3	2.8	
	Summer water deficit	2.4	2	
	Flooding	1.8	2	
	Hydrologic regime	1.3	2.5	
	Sea level rise	1.3	2	
	Sea surface temperature	3.2	2.3	
	Ocean acidification exposure	3.9	3	
	Upwelling	1.7	1.8	
	Ocean currents	1.8	1	
	Exposure Score	High		
Overall Vulnerability Rank		Moderate		

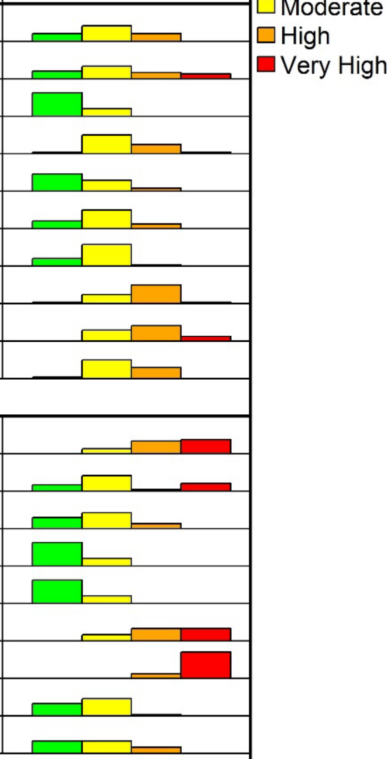

Life History Synopsis

Lake Ozette sockeye adults migrate a short distance over 2-3 days (on average) through the Ozette River between April and mid-August, with peak returns in May or June. Adults then hold in Lake Ozette for 3-9 months before spawning, when they display two different spawning behaviors: beach spawning and tributary spawning. Beach spawning occurs between mid-October and early February, with a peak in early January. The largest aggregation of tributary spawners migrates an additional 5-8 km upstream to Umbrella Creek, where early arriving fish may hold for up to several weeks before spawning. Tributary spawning occurs somewhat earlier than beach spawning, from early October to early January, with a peak in late November.

Fry typically emerge from both habitats in late March-April and rear for a full year in the lake, attaining a large size relative to other sockeye populations. Estuarine residence is not well known in terms of location and duration. After ocean entry, Lake Ozette sockeye juveniles are thought to stay nearshore until fall before migrating offshore to the Gulf of Alaska. However, a few juveniles genetically identified as Lake Ozette sockeye have been recovered in coastal waters off British Columbia (Beacham et al. 2014). Marine distributions of sockeye are thought to encompass oceanic regions of the subarctic Pacific (Farley et al. 2018). Marine survival of fish in this DPS is relatively high compared with other sockeye. Most adults spawn at age 4, but in the past decade spawner age has expanded to include age-3 and age-5 adults.

Climate Effects on Abundance and Distribution

Lake Ozette sockeye scored mostly ***moderate*** and ***low*** in life stage sensitivity to climate change. Adult migrants might face higher predation during low flow periods and can experience some temperature stress if heat spells coincide with migration periods (Haggerty 2009). However, the migration is short and occurs in spring and early summer, so adults can likely avoid both high temperatures and low flows. Lake temperatures are relatively cool and do not pose an imminent threat at either the adult or juvenile freshwater stages. High flows might reduce suitable spawning habitat because of conditions during redd construction or fine sediment accumulation lowering incubation survival.

Marine survival presumably fluctuates with climate for this DPS, as has been observed widely in other sockeye populations. However, the relatively large body size of smolts from this DPS appears to have buffered it historically from severely depressed returns during poor climate years. In terms of exposure attributes, this DPS was scored ***high*** for *sea surface temperature* and *stream temperature*, and ***very high*** for *ocean acidification*.

Extrinsic Factors

For Lake Ozette sockeye, sensitivity was ranked ***high*** for *population viability* and *other stressors*. The abundance of this population is still low, and there has been some loss in the spatial distribution of beach spawners. Nonetheless, spatial and temporal diversity has been increasing, and productivity has fluctuated.

This DPS received one of its higher sensitivity scores (mean 2.7) for *other stressors*. Extensive timber harvest in the basin has increased fine sediments in stream and beach habitats, reducing suitable spawning area. Increased peak flows and temperatures may exacerbate the effects of fine sediment. Large amounts of fine sediment were recruited into tributaries and then expelled into the lake during extensive forest harvest in the last century. Beach spawning at the tributary mouths is no longer observed, presumably because of fine sediment accumulation in the spawning gravel. The two remaining beach spawning locations are 5-56 km from tributary mouths.

Furthermore, introduced largemouth bass is now present in Lake Ozette in low numbers, and these numbers are likely to expand in a warmer climate.

Negative hatchery impacts are thought to be relatively low for this population. There is a supplementation hatchery, but use of non-native brood stock is minimal. Overall, supplementation has enhanced diversity in spatial distribution and age structure, which should improve resilience to climate change. Nonetheless, the population is not self-sustaining, and negative hatchery impacts are possible; these risks led to ***moderate*** scores for *hatchery influence*.

Adaptive Capacity

Lake Ozette sockeye scored ***low*** in adaptive capacity. Sockeye are unlikely to respond to climate change by changing life-history characteristics. Furthermore, little habitat exists that could potentially be improved to become more suitable for these fish. Low population abundance and spatial diversity suggest limited genetic heterogeneity that would support rapid adaptation. At present, adult migration spans a broad temporal window (April to mid-August), but this period may contract as adults attempt to avoid high temperatures and low flows in summer.

Literature Cited

Beacham, T. D., R. J. Beamish, J. R. Candy, C. Wallace, S. Tucker, J. H. Moss, and M. Trudel. 2014. Stock-specific migration pathways of juvenile sockeye salmon in British Columbia waters and in the Gulf of Alaska. Trans. Am. Fish. Soc. 143:1386-1403.

Farley, E. V., T. D. Beacham, and A. V. Bugaev. 2018. Ocean ecology of sockeye salmon.*in* R. J. Beamish, editor. The ocean ecology of Pacific salmon and trout. American Fisheries Society, Bethesda.

Haggerty, M. 2009. Lake Ozette sockeye limiting factors analysis. Prepared for the Makah Indian Tribe and National Marine Fisheris Service in cooperation with the Lake Ozette Sockeye Steering Committee, Port Angeles, WA.

Steelhead (*Oncorhynchus mykiss*)

Southern California Coast steelhead

Overall vulnerability—High (99% High, 1% Very high)
Biological sensitivity—High (100% High)
Climate exposure—High (99% High, 1% Very high)
Adaptive capacity—Low (1.56)
Data quality—63% of scores ≥ 2

	Southern California Steelhead	Expert Scores	Data Quality	Expert Scores Plots (Portion by Category)
Sensitivity attributes	Early life history	1.8	2.3	
	Juvenile freshwater stage	2.8	2.5	
	Estuary stage	2.8	1.8	
	Marine stage	2.5	1.5	
	Adult freshwater stage	2.3	2.5	
	Cumulative life-cycle effects	2.6	2.3	
	Hatchery influence	1.4	1.8	
	Other stressors	3.5	2	
	Population viability	3.4	2.3	
	Ocean acidification sensitivity	1.9	0.5	
	Sensitivity Score	High		
Exposure variables	Stream temperature	2.7	2.3	
	Summer water deficit	2.7	2.3	
	Flooding	3.3	1.8	
	Hydrologic regime	1.3	2.3	
	Sea level rise	3.1	2	
	Sea surface temperature	3.4	2	
	Ocean acidification exposure	4	3	
	Upwelling	2.5	1.5	
	Ocean currents	1.9	1	
	Exposure Score	High		
Overall Vulnerability Rank		High		

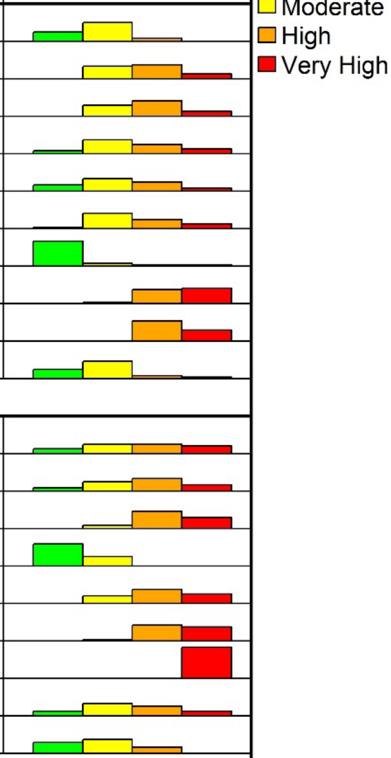

Life History Synopsis

For Southern California Coast steelhead, spawning primarily occurs from January through April and incubation from January through May. Juveniles probably migrate as a mix of age-1 and -2 smolts, but juvenile age class is poorly known. These populations are likely highly opportunistic in their movement patterns due to the natural uncertainty of rainfall and migration flows in the region. Small estuaries of numerous coastal streams provide key rearing habitat for the important "lagoon anadromous" life-history type, especially in summer and early fall (Boughton et al. 2007a; Hayes et al. 2008). In addition to the anadromous life-history, varying proportions of populations may pursue a

freshwater-resident life history, reaching sexual maturity without migrating to the ocean. Proportions of the resident type vary depending on local environmental conditions.

Ocean migration of Southern California Coast steelhead is poorly understood, but limited data indicate that fish from this DPS travel to the central subarctic North Pacific Ocean (Burgner 1992; Hayes and Kocik 2014). In general, North American steelhead undergo extensive ocean migrations into the Gulf of Alaska and subarctic North Pacific Ocean, and are estimated to tolerate an absolute thermal range of 2.8-15.8°C for all seasons and a frequently observed range of 6-12.5°C from spring to fall and 5-11°C during winter (Abdul-Aziz et al. 2011; Myers 2018). Typical age at maturity is thought to vary between age 2 and age 4, but among-year variation in age class is not well understood.

Climate Effects on Abundance and Distribution

The Southern California Coast steelhead DPS encompasses the southern range limit of the species, inhabiting coastal stream systems southward to the Tijuana River at the U.S. border with Mexico, and with scattered outlying populations on the Pacific coast of the Baja California peninsula. Migration and life history patterns are strongly constrained by geographic patterns of rainfall and surface flow in this arid region.

Rainfall in this recovery domain is substantially lower and more variable than in regions to the north, and very rarely occurs in summer, resulting in a natural pattern of disconnected surface flows during the dry season and in dry years. Disconnections are structured by geomorphic processes, typically occurring in low-gradient alluvial channels, tributary junctions, and estuary mouths, where sediment tends to accumulate. Thus, some of the most secure habitats are steep highland creeks, which are both cool and well-watered, but frequently disconnected from the ocean. Southern California Coast steelhead appears to have adapted to this challenging landscape, primarily by exhibiting resilient physiology and by retaining life-history plasticity that allows individuals to opportunistically pursue either a freshwater-resident or anadromous life history.

A critical limit seems to be the ability of parr to grow rapidly and attain the smolt stage at a large size, since large smolts have significantly higher rates of survival to adulthood (Boughton et al. 2015). This DPS ranked ***moderate*** in sensitivity at the *juvenile freshwater* and *estuary stages*, while its other life stages ranked ***low***. Exposure to sea level rise was ranked ***high*** due to the risk of losing critical lagoon rearing habitat.

Although this DPS occurs in relatively warm and xeric stream systems, it has never been shown to have higher thermal tolerance than the rest of the species. Instead, perennial creek habitats where the species occurs typically have peak daily temperatures lower than 18°C (Boughton et al. 2007b). These stream temperatures are buffered by evapotranspiration in the riparian tree canopy (Boughton et al. 2012) or by upwelling in the nearby Pacific Ocean. However, juvenile *O. mykiss* have been documented to

withstand peak daily temperatures up to ~29-30°C in the aftermath of a canopy-replacing wildfire (Sloat and Osterback 2013), suggesting a capacity for high thermal tolerance during extreme events. Southern California Coast steelhead ranked ***moderate*** for exposure to *stream temperature* and *summer water deficit*, primarily because of the link between summer climate and ocean *upwelling*, for which its exposure also ranked ***moderate***. However, we noted that the spread in expert scores was relatively high for this DPS, reflecting greater uncertainty about the stability of these habitats in the face of climate change.

Southern California Coast steelhead ranked ***high*** for flood exposure. Many river channels that serve as migration routes for this DPS are dry or have sections that are dry except immediately after winter storms. Therefore, migration occurs only during the brief periods after these storms and may not occur at all in dry years due lack of surface flow. Some years see large numbers of adults trapped in fresh water before they can migrate back to the ocean (Barnett and Spence 2011). Streamflow between storms is the primary climate-driven barrier to migration. Water velocities and sedimentation during winter storms may also limit survival (Boughton et al. 2009, 2015), depending on habitat conditions. Particularly important during such storms is the availability of large roughness elements such as boulders and coarse wood, which provide velocity refugia.

Abdul-Aziz et al. (2011) developed spatially explicit representations of open ocean thermal habitat for steelhead. They found that under a multi-model ensemble average of climate model outputs using the A1B emissions scenario, summer habitat area for steelhead declined by 36% for the 2080s, with the largest habitat losses in the northeast Pacific Ocean. Wintertime habitat area losses were 2%, with reductions at the southern end of the historical range largely offset by gains in the Bering Sea and Sea of Okhotsk. Whether a general northward and westward displacement of thermal open-ocean habitat will have substantial impacts on the life-cycle productivity or spawning distribution of West Coast steelhead is not known. However, these populations are vulnerable to the projected displacement of high-seas thermal habitat. Thus, this DPS ranked ***high*** in exposure to *sea surface temperature* and ***moderate*** in sensitivity at the *marine stage*.

Extrinsic Factors

Southern California Coast steelhead ranked ***high*** for *other stressors*. Major threats to this DPS include water withdrawals for agriculture and domestic use. Such withdrawals reduce stream flows and can make channels shallow or dry them outright, lowering water tables enough to kill riparian vegetation and destabilize channel structure. Extensive channelization in habitats occupied by this DPS has already reduced vegetative shading and hyporheic exchange. In many cases, stream banks have been completely replaced with concrete. This DPS is also confronted by many invasive species, especially in the urbanized areas of Los Angeles and San Diego, and occupies rivers such as the

Santa Ynez, which are managed for summer agricultural water supply (Moyle 2002; Marchetti et al. 2004; Robinson et al. 2009).

Fisheries impacts on Southern California Coast steelhead are thought to be low, with recreational fisheries limited to streams above impassable barriers. Poaching during low-flows may be a problem in some streams, particularly in the disconnected pools of urban areas during the summer low-flow season or between storms during the migration season.

In addition, this DPS faces many geomorphic stressors: sea level rise interacts with coastal urban development, especially around estuaries; floodplains disconnected via levees exacerbate extreme flashiness in stream flows; and dams sequester sediment in streams where arid climate and flashy storms produce large movements of sediment. Additional habitat loss is related to the presence of an invasive plant (*Arundo*) that uses much water and traps fine sediments. There are also numerous infrastructure barriers to dispersal. Finally, anthropogenic breaching of lagoon/estuary systems poses an additional threat to populations in this DPS. Climate change exacerbates all of these threats because it will reduce usable habitat throughout the range of this DPS (Moyle et al. 2017, p. 327.).

Southern California Coast steelhead is listed as endangered under the Endangered Species Act, and its sensitivity to *population viability* ranked ***high***. Abundance, productivity, spatial structure, and diversity are all at high risk for this DPS. No hatcheries influence this DPS at present, but hatchery supplementation is being discussed.

Adaptive Capacity

This DPS has already evolved life-history flexibility to deal with the challenges of a warm and arid climate, including coexistence of anadromous and resident forms in the same population. However, because habitat is already so limited, there is little room for future adaptation by this DPS, and its adaptive capacity ranked ***low***. Moyle et al. (2017) came to a similar conclusion.

Literature Cited

Abdul-Aziz, O. I., N. J. Mantua, and K. W. Myers. 2011. Potential climate change impacts on thermal habitats of Pacific salmon (*Oncorhynchus spp.*) in the North Pacific Ocean and adjacent seas. Can. J. Fish. Aquat. Sci. 68:1660-1680.

Barnett, L. A. K., and B. C. Spence. 2011. Freshwater survival of stranded steelhead kelts in coastal central California streams. N. Am. J. Fish. Manage. 31:757-764.

Boughton, D. A., P. B. Adams, E. Anderson, C. Fusaro, E. Keller, E. Kelley, L. Lentsch, J. Nielsen, K. Perry, H. Regan, J. Smith, C. Swift, L. Thompson, and F. Watson. 2007a. Viability criteria for steelhead of the south-central and southern California coast. U.S. Dep. Commerce NOAA Tech. Memo. NMFS-SWFSC-407.

Boughton, D. A., H. Fish, J. Pope, and G. Holt. 2009. Spatial patterning of habitat for *Oncorhynchus mykiss* in a system of intermittent and perennial streams. Ecol. Freshwat. Fish 18:92-105.

Boughton, D. A., M. Gibson, R. Yedor, and E. Kelley. 2007b. Stream temperature and the potential growth and survival of juvenile *Oncorhynchus mykiss* in a southern California creek. Freshwat. Biol. 52:1353-1364.

Boughton, D. A., L. R. Harrison, A. S. Pike, J. L. Riaza, and M. Mangel. 2015. Thermal potential for steelhead life history expression in a southern California alluvial river. Trans. Am. Fish. Soc. 144:258-273.

Boughton, D. A., C. Hatch, and E. Mora. 2012. Identifying distinct thermal components of a creek. Water Resources Res. 48.

Burgner, R. L. 1992. Distribution and origins of steelhead trout (*Oncorhynchus Mykiss*) in offshore waters of the North Pacific Ocean. Int. N. Pac. Fish. Comm. Bull. 51.

Hayes, S. A., M. H. Bond, C. V. Hanson, E. V. Freund, J. J. Smith, E. C. Anderson, A. J. Ammann, and R. B. MacFarlane. 2008. Steelhead growth in a small central California watershed: upstream and estuarine rearing patterns. Trans. Am. Fish. Soc. 137:114-128.

Hayes, S. A., and J. F. Kocik. 2014. Comparative estuarine and marine migration ecology of Atlantic salmon and steelhead: blue highways and open plains. Rev. Fish Biol. Fish. 24:757-780.

Marchetti, M. P., T. Light, P. B. Moyle, and J. H. Viers. 2004. Fish invasions in California watersheds: testing hypotheses using landscape patterns. Ecol. Appl. 14:1507-1525.

Moyle, P. B. 2002. Inland fishes of California. University of California Press, Berkeley.

Moyle, P. B., R. Lusardi, P. Samuel, and J. Katz. 2017. State of the salmonids: Status of California's emblematic fishes 2017. Center for Watershed Sciences, University of California, Davis and California Trout, San Francisco. Available at http://caltrout.org/sos/.

Myers, K. W. 2018. Ocean ecology of steelhead. Page 1090 *in* R. J. Beamish, editor. The ocean ecology of Pacific salmon and trout. American Fisheries Society, Bethesda.

Robinson, T., C. Hanson, S. Engblom, S. Volan, J. Baldrige, L. Riege, B. Wales, A. Shahroody, and C. Lawler. 2009. Summary and analysis of annual fishery monitoring in the lower Santa Ynez River, 1993–2004. Cachuma Conservation Release Board, Santa Barbara, CA.

Sloat, M. R., and A. M. Osterback. 2013. Maximum stream temperature and the occurrence, abundance, and behavior of steelhead trout in a southern California stream. Can. J. Fish. Aquat. Sci. 70:64-73.

South Central California Coast steelhead

Overall vulnerability—Moderate (56% Moderate, 44% High)
Biological Sensitivity –Moderate (56% Moderate, 44% High)
Climate exposure—High (99% High, 1% Very high)
Adaptive capacity—Moderate (1.6)
Data quality—63% of scores ≥ 2

	South-Central California Coast Steelhead	Expert Scores	Data Quality	Expert Scores Plots (Portion by Category)
Sensitivity attributes	Early life history	1.8	2.3	
	Juvenile freshwater stage	2.8	2.5	
	Estuary stage	2.8	1.8	
	Marine stage	2.4	1.5	
	Adult freshwater stage	2.3	2.5	
	Cumulative life-cycle effects	2.5	2.3	
	Hatchery influence	1.4	1.8	
	Other stressors	3.4	2	
	Population viability	2.9	2.3	
	Ocean acidification sensitivity	1.9	0.5	
	Sensitivity Score	Moderate		
Exposure variables	Stream temperature	2.7	2.3	
	Summer water deficit	2.7	2.3	
	Flooding	3.2	1.8	
	Hydrologic regime	1.3	2.3	
	Sea level rise	3.1	2	
	Sea surface temperature	3.4	2	
	Ocean acidification exposure	4	3	
	Upwelling	2.5	1.5	
	Ocean currents	1.9	1	
	Exposure Score	High		
Overall Vulnerability Rank		Moderate		

Low
Moderate
High
Very High

Life History Synopsis

Adults of the South Central California Coast steelhead DPS enter streams in winter and spawn shortly thereafter (Moyle et al. 2017). Spawning periods extend primarily from January through April, and incubation primarily from January through May. Smolts enter the ocean in April and May and probably rear for 1-2 years, but this rearing period is poorly understood. Small estuaries of numerous coastal streams provide key rearing habitat for the important "lagoon anadromous" life-history type, especially in summer and early fall when lagoon closures restrict ocean access (Boughton et al. 2007a; Hayes et al. 2008). Thus, in addition to the anadromous life-history, fish from this DPS may pursue a freshwater-resident life history, reaching sexual maturity without migrating

to the ocean. This strategy is employed by varying proportions of each steelhead population, depending on local environmental conditions.

Ocean migrations of South Central California Coast steelhead are poorly understood, but limited data indicate that these fish travel to the central subarctic North Pacific Ocean (Burgner et al. 1992; Hayes and Kocik 2014). In general, North American steelhead undergo extensive ocean migrations into the Gulf of Alaska and subarctic North Pacific Ocean, occupying regions with an absolute thermal range of 2.8-15.8°C for all seasons, and a frequently observed range of 6-12.5°C during spring-fall and 5-11°C during winter (Abdul-Aziz et al. 2011; Myers 2018). Typical age at maturity is thought to vary between 2 and 4 years, but the factors that determine among-year variation are not clear.

Climate Effects on Abundance and Distribution

For South Central California Coast steelhead, a critical limit seems to be the ability of parr to grow rapidly and complete the smolt transition at a large size, since large smolts survive to adulthood at considerably higher rates (Boughton et al. 2015). This DPS ranked ***moderate*** in sensitivity at both the *juvenile freshwater* and *estuary stage*, due in part to potential negative effects of warmer or more variable thermal conditions on realized growth (Boughton et al. 2007b). Its rank for exposure to *sea level rise* was ***high*** because sea level rise could lead to the loss of critical lagoon rearing habitat. Lagoons provide the primary habitat for producing large smolts in some systems.

South Central California Coast steelhead ranked low in sensitivity scores for other life stages. Although this DPS inhabits relatively warm and xeric stream systems, it has not been shown to have higher thermal tolerance than the rest of the species. Instead, perennial creek habitats where steelhead typically occur have peak daily temperatures below 18°C (Boughton et al. 2009), with stream temperature buffered by evapotranspiration in the riparian tree canopy (Boughton et al. 2012) or by the upwelling regime in the nearby Pacific Ocean. However, just south of the range occupied by South Central California Coast steelhead, juvenile *O. mykiss* were reported to have withstood peak daily temperatures up to ~29-30°C in the aftermath of a canopy-replacing wildfire (Sloat and Osterback 2013), suggesting a capacity for high thermal tolerance during extreme events. South Central California Coast steelhead ranked ***moderate*** for exposure to *stream temperature* and *summer water deficit*, primarily because of the link between summer climate and ocean *upwelling* regime, for which this DPS was also ranked at ***moderate*** exposure risk. Nevertheless, the spread in expert scores was relatively high, reflecting greater uncertainty about the stability of these habitats in the face of climate change.

This DPS ranked ***high*** for exposure to *flooding*. Increasing floods could be beneficial for this region because many of the channels in which fish migrate are dry or have sections that are dry except immediately after winter storms, and migration occurs

only during these brief periods. Migration may not occur at all in dry years due to lack of surface flow. In some years, large numbers of adults are trapped in fresh water before they can migrate to the ocean (Barnett and Spence 2011). Streamflow between storms is the primary climate-driven barrier to migration, and increasing variability in flows could pose further challenges (Black et al. 2018). Water velocities and sedimentation during winter storms may also limit survival (Boughton et al. 2009, 2015), depending on habitat conditions such as the presence of velocity refugia.

Abdul-Aziz et al. (2011) developed spatially explicit representations of thermal habitat for steelhead in the open ocean. They found that under a multi-model ensemble average of climate model outputs using the A1B emissions scenario, summer habitat area for steelhead declined by 36% for the 2080s, with the largest losses in the northeast Pacific Ocean. Wintertime habitat area losses were 2%, with reductions at the southern end of the historical range largely offset by habitat area gains in the Bering Sea and Sea of Okhotsk. Whether a general northward and westward shift of its most frequently observed thermal ocean habitat will have substantial impacts on steelhead life-cycle productivity or spawning distribution is not known. However, this DPS may be vulnerable to the projected displacement of high seas thermal habitat; therefore, it ranked ***high*** in exposure for *sea surface temperature*.

Extrinsic Factors

South Central California Coast steelhead ranked ***high*** for *other stressors*, consistent with the conclusions of Moyle et al. (2017). A major threat is water withdrawal for agriculture and domestic use. Such withdrawals lower groundwater levels and reduce stream flows, making stream channels shallow or completely dry, as well as killing riparian vegetation and widening channels to dry sandy washes (Kondolf 1982). There are many invasive species in the larger inland watersheds of the Pajaro and Salinas River, and the latter has a hydrograph that is highly altered by summer agricultural flows (Moyle 2002; Marchetti et al. 2004; Robinson et al. 2009). Fisheries impacts are thought to be low, with recreational fisheries generally limited to streams above impassable barriers or, during migration seasons, to catch-and-release fisheries in short sections of channel near the ocean. Poaching during summer low-flows may be a problem in some streams.

In addition to these, the South Central California Coast steelhead DPS faces many geomorphic stressors: sea level rise interacts with coastal urban development, especially around estuaries; levees disconnect floodplains, exacerbating the extreme flashiness of regional stream flows and reducing groundwater infiltration; and dams sequester sediment and starve downstream channels of gravel. In lower-elevation streams, arid climate and flashy storms produce large movements of fine sediment (Harrison et al. 2018). Additional habitat loss is related to the presence of an invasive plant *Arundo*, which uses much water and traps fine sediments. There are numerous infrastructure barriers to population dispersal. Finally, anthropogenic breaching of lagoon/estuary

systems poses an additional threat to populations in this DPS. Climate change will exacerbate all of these extrinsic threats by reducing usable habitat throughout the DPS range (Moyle et al. 2017, p. 327).

No hatcheries influence this DPS at present, but hatchery production is being discussed. South Central California Coast steelhead is listed as threatened under the U.S. Endangered Species Act, and its overall *population viability* ranked ***moderate***. For overall vulnerability of this DPS, abundance ranked high, productivity moderate, spatial structure low, and diversity high.

Adaptive Capacity

This DPS has already evolved life-history flexibility to deal with a warm, arid climate, including coexistence of anadromous and resident forms in the same population. Because of this life history flexibility, South Central California Coast steelhead ranked ***moderate*** for adaptive capacity overall.

Literature Cited

Abdul-Aziz, O. I., N. J. Mantua, and K. W. Myers. 2011. Potential climate change impacts on thermal habitats of Pacific salmon (*Oncorhynchus spp.*) in the North Pacific Ocean and adjacent seas. Can. J. Fish. Aquat. Sci. 68:1660-1680.

Barnett, L. A. K., and B. C. Spence. 2011. Freshwater survival of stranded steelhead kelts in coastal central California streams. N. Am. J. Fish. Manage. 31:757-764.

Black, B. A., P. van der Sleen, E. Di Lorenzo, D. Griffin, W. J. Sydeman, J. B. Dunham, R. R. Rykaczewski, M. García-Reyes, M. Safeeq, I. Arismendi, and S. J. Bograd. 2018. Rising synchrony controls western North American ecosystems. Global Change Biol. 24:2305-2314.

Boughton, D. A., P. B. Adams, E. Anderson, C. Fusaro, E. Keller, E. Kelley, L. Lentsch, J. Nielsen, K. Perry, H. Regan, J. Smith, C. Swift, L. Thompson, and F. Watson. 2007a. Viability criteria for steelhead of the south-central and southern California coast. U.S. Dep. Commerce NOAA Tech. Memo. NMFS-SWFSC-407.

Boughton, D. A., H. Fish, J. Pope, and G. Holt. 2009. Spatial patterning of habitat for *Oncorhynchus mykiss* in a system of intermittent and perennial streams. Ecol. Freshwat. Fish 18:92-105.

Boughton, D. A., M. Gibson, R. Yedor, and E. Kelley. 2007b. Stream temperature and the potential growth and survival of juvenile *Oncorhynchus mykiss* in a southern California creek. Freshwat. Biol. 52:1353-1364.

Boughton, D. A., L. R. Harrison, A. S. Pike, J. L. Riaza, and M. Mangel. 2015. Thermal potential for steelhead life history expression in a southern California alluvial river. Trans. Am. Fish. Soc. 144:258-273.

Boughton, D. A., C. Hatch, and E. Mora. 2012. Identifying distinct thermal components of a creek. Water Resource Res. 48.

Burgner, R. L., J. T. Light, L. Margolis, T. Okazaki, A. Tautz, and S. Ito. 1992. Distribution and origins of steelhead trout (*Oncorhynchus mykiss*) in offshore waters of the North Pacific Ocean. Int. N. Pac. Fish. Comm. Bull. 51.

Harrison, L. R., A. E. East, D. P. Smith, J. B. Logan, R. M. Bond, C. L. Nicol, T. H. Williams, D. A. Boughton, K. Chow, and L. Luna. 2018. River response to large-dam removal in a Mediterranean hydroclimatic setting: Carmel River, California, USA. Earth Surf. Process. Landf. doi 10.1002/esp.4464.

Hayes, S. A., M. H. Bond, C. V. Hanson, E. V. Freund, J. J. Smith, E. C. Anderson, A. J. Ammann, and R. B. MacFarlane. 2008. Steelhead growth in a small central California watershed: upstream and estuarine rearing patterns. Trans. Am. Fish. Soc. 137:114-128.

Hayes, S. A. and J. F. Kocik. 2014. Comparative estuarine and marine migration ecology of Atlantic salmon and steelhead: blue highways and open plains. Rev. Fish Biol. Fish. 24:757-780.

Kondolf, G. M. 1982. Recent channel instability and historic channel changes of the Carmel River, Monterey County, California. M.S. Thesis, University of California Santa Cruz, Santa Cruz, CA.

Marchetti, M. P., T. Light, P. B. Moyle, and J. H. Viers. 2004. Fish invasions in California watersheds: testing hypotheses using landscape patterns. Ecol. Appl. 14:1507-1525.

Moyle, P. B. 2002. Inland fishes of California. University of California Press, Berkeley, CA.

Moyle, P. B., R. Lusardi, P. Samuel, and J. Katz. 2017. State of the salmonids: Status of California's emblematic fishes 2017. Center for Watershed Sciences, University of California, Davis and California Trout, San Francisco. Available at http://caltrout.org/sos/.

Myers, K. W. 2018. Ocean ecology of steelhead. Page 1090 *in* R. J. Beamish, editor. The ocean ecology of Pacific salmon and trout. American Fisheries Society, Bethesda.

Robinson, T., C. Hanson, S. Engblom, S. Volan, J. Baldrige, L. Riege, B. Wales, A. Shahroody, and C. Lawler. 2009. Summary and analysis of annual fishery monitoring in the lower Santa Ynez River, 1993–2004. Cachuma Conservation Release Board, Santa Barbara, CA.

Sloat, M. R., and A. M. Osterback. 2013. Maximum stream temperature and the occurrence, abundance, and behavior of steelhead trout in a southern California stream. Can. J. Fish. Aquat. Sci. 70:64-73.

California Central Valley steelhead

Overall vulnerability—Moderate (76% Moderate, 24% High)
Biological Sensitivity –Moderate (76% Moderate, 23% High)
Climate exposure—High (99% High, 1% Very high)
Adaptive capacity—Moderate (1.7)
Data quality—63% of scores ≥ 2

	California Central Valley Steelhead	Expert Scores	Data Quality	Expert Scores Plots (Portion by Category)
Sensitivity attributes	Early life history	1.6	2.8	
	Juvenile freshwater stage	2.1	2.8	
	Estuary stage	2.5	1.8	
	Marine stage	1.6	1.3	
	Adult freshwater stage	1.5	2.8	
	Cumulative life-cycle effects	2.1	2.5	
	Hatchery influence	3.1	2	
	Other stressors	2.9	2	
	Population viability	2.1	2	
	Ocean acidification sensitivity	1.9	0.5	
	Sensitivity Score	Moderate		
Exposure variables	Stream temperature	2.8	2.3	
	Summer water deficit	2.6	2	
	Flooding	3.2	1.8	
	Hydrologic regime	1.9	1.8	
	Sea level rise	2.2	2	
	Sea surface temperature	3.4	2	
	Ocean acidification exposure	4	3	
	Upwelling	2.5	1.5	
	Ocean currents	1.9	1	
	Exposure Score	High		
Overall Vulnerability Rank		Moderate		

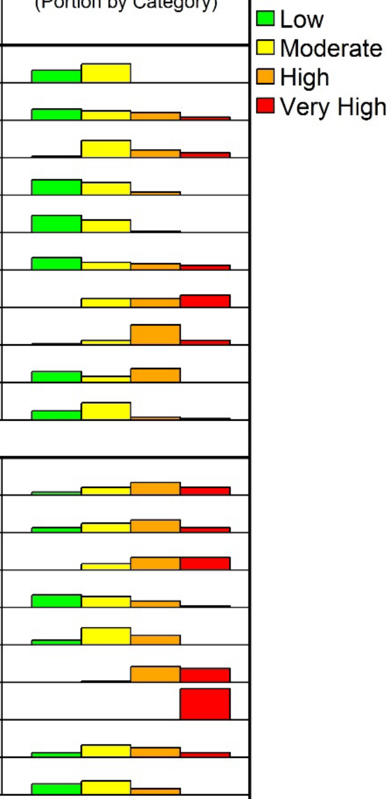

Life History Synopsis

The California Central Valley steelhead DPS includes populations spawning in the Sacramento and San Joaquin Rivers and their tributaries. Detail on the life history of Central Valley steelhead is covered by Williams (2006) and Moyle et al. (2017). For these steelhead populations, the peak adult migration seems to have occurred historically from late September to late October, with some creeks such as Mill Creek showing a small run in mid-February (Lindley et al. 2006). This DPS was once found throughout Central Valley rivers, but more than 80% of historic habitat is now above dams (Lindley et al. 2006). Juveniles generally migrate from late December through the beginning of May, with a peak in mid-March. There is a much smaller peak in fall, and juvenile migrations are generally cued to high-flow events.

Juvenile steelhead are opportunistic predators on anything available in their rearing streams, from aquatic and terrestrial insects to small fish. However, benthic aquatic insect larvae are the mainstay of their diet. Below reservoirs, zooplankton may be important as well. Diets shift with season and size of the juveniles. At times, salmon eggs, juvenile salmon, sculpins, and suckers may be important prey for yearling steelhead and may be key prey items for rapid growth. Once they migrate to sea, steelhead prey largely on fish and crustaceans. Ocean movements of fish in this DPS are poorly understood.

Steelhead juveniles typically spend little time in estuarine habitats compared to other juvenile salmonids (Hayes and Kocik 2014; Myers 2018). During marine residence, steelhead juveniles move rapidly offshore and are widely distributed across the subarctic North Pacific (Myers 2018). California Central Valley steelhead is presumed to have originally spent 1-2 years at sea before returning to spawn at sizes of 40-50 cm FL. Present-day hatchery steelhead spend 1-3 years at sea and spawn at 50-80 cm FL. There is little evidence of repeat spawning.

Climate Effects on Abundance and Distribution

Climate impacts to California Central Valley steelhead are similar to those detailed for northern steelhead in the account of Moyle et al. (2017). This DPS ranked high in climate exposure attributes overall because of ***high*** rankings for exposure to *flooding* and *sea surface temperature*, and a ***very high*** ranking in exposure to *ocean acidification*. California Central Valley steelhead ranked ***moderate*** in overall sensitivity, with the *estuary stage* being the main intrinsic contributing factor.

Extrinsic Factors

California Central Valley steelhead ranked ***high*** in sensitivity to *hatchery influence* because production hatcheries dominate composition of this DPS (Moyle et al. 2017). Indeed, catch from the U.S. Fish and Wildlife Service Chipps Island midwater trawl indicates a continued decline in steelhead natural production since 2010, with the proportion of adipose fin-clipped steelhead reaching 95% (Johnson and Lindley 2016). Nimbus Hatchery steelhead remain genetically divergent from other Central Valley lineages because the broodstock originates from coastal steelhead stocks (Pearse and Garza 2015). However, maintenance of this coastal ancestry creates vulnerability to hybridization with other Central Valley populations (NMFS 2009). For this reason, researchers have suggested that Nimbus broodstock be replaced by *O. mykiss* with native American River ancestry (Abadía-Cardoso et al. In press).

Central Valley steelhead is listed as threatened under the U.S. Endangered Species Act. This DPS was ranked ***moderate*** for *population viability*, although the latest status review considered the anadromous life history type to be at high risk of extinction. There are populations of resident *O. mykiss* above dams that may be more similar genetically to ancestral populations than the present listed populations below the dams

(Pearse and Garza 2015; Pearse and Campbell 2018). Upstream from major dams, resident *O. mykiss* use reservoirs like the ocean, and this life history type may maintain a genetic reservoir for steelhead and provide some gene flow to populations below dams.

Maintenance of varied steelhead life history types is dependent on the growth-survival-fitness landscape between resident and ocean strategies (Satterthwaite et al. 2010). Many naturally spawned steelhead and rainbow trout have adapted a resident strategy in Central Valley rivers, where temperatures are artificially cool in summer. These fish remain in freshwater, foregoing ocean growth benefits to avoid high mortality during migration through the Sacramento River and San Joaquin River Delta, along with potentially adverse ocean conditions (Lindley et al. 2009; Perry et al. 2016). Considering that dams have blocked access to the majority of historical habitat, but that rearing conditions have improved in some reaches for resident life history types, the Central Valley steelhead DPS ranked ***moderate*** the ranking for *other stressors* was ***moderate***.

Adaptive Capacity

Central Valley steelhead exhibit flexible reproductive strategies, which have historically allowed for persistence in spite of variable conditions in the Central Valley. Adaptive capacity ranked ***moderate*** for adaptive capacity because despite a flexible life history, its range is severely limited to areas below dams.

As in other steelhead DPSs, the relationship between anadromous and resident rainbow trout is complex, but populations with both life history types offer the greatest adaptive evolutionary potential (Pearse et al. 2014). Anadromous steelhead produce many more eggs than resident fish and improve gene flow among rivers, maximizing genetic diversity. Resident fish persist when ocean conditions cause poor survival of anadromous types, while anadromous fish can recolonize streams in which resident populations have been wiped out by drought or other natural disasters.

Scorers considered that populations of *O. mykiss* in the California Central Valley will likely persist, even under severe climate change scenarios, because resident and adfluvial forms will remain in the cold-water streams above dams. Presumably this DPS will be much less abundant, with more scattered populations, in response to climate change. Few populations may remain in valley floor streams except below reservoirs, where cold-water releases can be sustained through long periods of drought (e.g., Berryessa Reservoir).

Literature Cited

Abadía-Cardoso, A., A. Brodsky, B. Cavallo, M. Arciniega, J. C. Garza, J. Hannon, and D. E. Pearse. 2019. Anadromy redux? Genetic analysis of upper American River Rainbow Trout *Oncorhynchus mykiss* to inform development of an indigenous steelhead broodstock for Nimbus Hatchery. J. Fish. Wildlife Manage. **10**(1): 137-147.

Hayes, S. A., and J. F. Kocik. 2014. Comparative estuarine and marine migration ecology of Atlantic salmon and steelhead: blue highways and open plains. Rev. Fish Biol. Fish. 24:757-780.

Johnson, R. C., and S. T. Lindley. 2016. Isotopes and genes reveal freshwater origins of Chinook salmon (*Oncohynchus tshawytscha*) aggregations in California's coastal ocean. U.S. Dep. Commerce NOAA Tech. Memo. NMFS-SWFSC-564.

Lindley, S., R. S. Schick, A. Agrawal, M. Goslin, T. E. Pearson, E. Mora, J. Anderson, B. May, S. Greene, C. Hanson, A. Low, D. McEwan, R. Macfarlane, C. Swanson, and J. G. Williams. 2006. Historical population structure of Central Valley steelhead and its alteration by dams. San Francisco Est. Water Sci. 4.

Lindley, S. T., C. B. Grimes, M. S. Mohr, W. Peterson, J. Stein, J. T. Anderson, L. W. Botsford, D. L. Bottom, C. A. Busack, T. K. Collier, J. Ferguson, J. C. Garza, A. M. Grover, D. G. Hankin, R. G. Kope, P. W. Lawson, A. Low, R. B. MacFarlane, K. Moore, M. Palmer-Zwahlen, F. B. Schwing, J. Smith, C. Tracy, R. Webb, B. K. Wells, and T. H. Williams. 2009. What caused the Sacramento River fall Chinook stock collapse? U.S. Dep. Commerce NOAA Tech. Memo. NMFS-SWFSC-447.

Moyle, P. B., R. Lusardi, P. Samuel, and J. Katz. 2017. State of the salmonids: Status of California's emblematic fishes 2017. Center for Watershed Sciences, University of California, Davis and California Trout, San Francisco. Available at http://caltrout.org/sos/.

Myers, K. W. 2018. Ocean ecology of steelhead. Page 1090 *in* R. J. Beamish, editor. The ocean ecology of Pacific salmon and trout. American Fisheries Society, Bethesda.

NMFS (National Marine Fisheries Service). 2009. Biological Opinion on the Operating Criteria and Plan (OCAP) for the long-term operations of the Central Valley Project and State Water Project with recommendations for Essential Fish Habitat. National Marine Fisheries Service, Southwest Region, Long Beach, CA.

Pearse, D. E., and M. A. Campbell. 2018. Ancestry and Adaptation of Rainbow Trout in Yosemite National Park. Fisheries 43:472-484.

Pearse, D. E., and J. C. Garza. 2015. You can't unscramble and egg: Population genetic structure of *Oncorhynchus mykiss* in the California Central Valley inferred from combined microsatellite and SNP data. San Francisco Est. Water Sci. 13.

Pearse, D. E., M. R. Miller, A. Abadia-Cardoso, and J. C. Garza. 2014. Rapid parallel evolution of standing variation in a single, complex, genomic region is associated with life history in steelhead/rainbow trout. Proc. Royal Soc. B 281:20140012.

Perry, R. W., R. A. Buchanan, P. L. Brandes, J. R. Burau, and J. A. Israel. 2016. Anadromous salmonids in the Delta: New science 2006–2016. San Francisco Est. Water Sci. 14.

Satterthwaite, W. H., M. P. Beakes, E. M. Collins, D. R. Swank, J. E. Merz, R. G. Titus, S. M. Sogard, and M. Mangel. 2010. State-dependent life history models in a changing (and regulated) environment: steelhead in the California Central Valley. Evol. Appl. 3:221-243.

Williams, J. G. 2006. Central Valley salmon: A perspective on Chinook and steelhead in the Central Valley of California. San Francisco Est. Water Sci. 4.

Central California Coast steelhead

Overall vulnerability—Moderate (65% Moderate, 35% High)
Biological Sensitivity Moderate (65% Moderate, 35% High)
Climate exposure—High (100% High)
Adaptive capacity—Moderate (2.0)
Data quality—58% of scores ≥ 2

	Central California Coast Steelhead	Expert Scores	Data Quality	Expert Scores Plots (Portion by Category)
Sensitivity attributes	Early life history	1.7	2.3	
	Juvenile freshwater stage	2.5	2.3	
	Estuary stage	2.9	1.8	
	Marine stage	2.1	1.8	
	Adult freshwater stage	1.8	2.5	
	Cumulative life-cycle effects	1.9	1.8	
	Hatchery influence	2.4	2	
	Other stressors	3	1.8	
	Population viability	2.9	2	
	Ocean acidification sensitivity	1.9	0.5	
	Sensitivity Score	Moderate		
Exposure variables	Stream temperature	2.7	2.3	
	Summer water deficit	2.7	2	
	Flooding	3.1	1.8	
	Hydrologic regime	1.3	2.3	
	Sea level rise	3	2	
	Sea surface temperature	3.4	2	
	Ocean acidification exposure	4	3	
	Upwelling	2.5	1.5	
	Ocean currents	1.9	1	
	Exposure Score	High		
Overall Vulnerability Rank		Moderate		

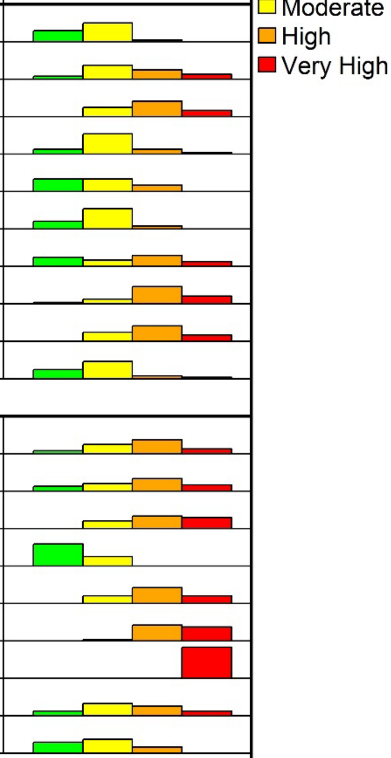

Life History Synopsis

Central California Coast steelhead exhibits considerable life history variation, though all adult migration occurs during winter (Moyle et al. 2017). Populations are found in streams below natural and man-made barriers from the Russian River south to Aptos Creek. Adults from this DPS enter rivers from October to May, depending on the system (Shapovalov and Taft 1954; Busby et al. 1996; Osterback et al. 2018), and can spawn soon after completing migration. However, most spawning occurs during late spring, thus avoiding the damaging effect on redds of winter floods common to Central California Coast watersheds. In Waddell Creek, the majority of spawners were comprised of age-3+ and age-4+ fish (35 and 46%, respectively); only 17% were repeat

spawners (Shapovalov and Taft 1954). Shapovalov and Taft (1954) identified 32 different combinations of freshwater/saltwater year type, although most were among the following four combinations: 2/1 (30%), 2/2 (27%), 3/1 (11%), and 1/2 (8%).

Central California Coast steelhead eggs hatch in 25-35 days, depending on temperature, and alevins emerge from the gravel as fry after 2-3 weeks. Fry move to the stream edges but switch to deeper water as they grow larger. The abundance of juveniles in tributaries to San Francisco Bay is positively correlated with elevation, stream gradient, dominant substrate size, and percent native species. Conversely, juvenile abundance is negatively correlated with stream order, average and maximum depth, wetted channel width, water temperature, water clarity, percent open canopy, conductivity, percent pool habitat, and total number of fish species (Leidy 2007).

Juveniles move downstream during all seasons of the year, though in smaller coastal systems, movement is limited during the low-flow periods of late summer and fall. Often, significant numbers of age-0 fish move downstream with the onset of fall rains, though most likely take up residence in lagoons rather than moving out to sea. The smolt migration typically takes place from February to May, with older age classes tending to migrate somewhat earlier in this window (Shapovalov and Taft 1954; Osterback et al. 2018). Some juveniles migrate to the estuary after spending only a few months in the upper watershed, while others spend 1-2 years rearing in fresh water. Both types typically spend 1-10 months rearing in the estuary prior to ocean entry, although some smolts migrate directly to the ocean without estuarine occupancy. Parr transitions to the smolt stage at sizes ranging from 90-150 mm FL or more.

Favorable conditions for rapid growth occur in lagoons at the mouths of streams and in stream reaches with high summer flow. Juveniles often remain in such lagoons after they close, foregoing ocean access (Hayes et al. 2008; Bond et al. 2017). These fish experience high growth rates, often doubling in length; the mean FL of fall lagoon residents can be over 200 mm. Juvenile steelhead larger than 150 mm FL have a significant survival advantage in the ocean; they comprised 85% of the returning adult population in Scott Creek, although they made up less than half of the estuary population.

Reconstructions from Central California Coast steelhead tagged with archival tags have indicated that these fish move rapidly offshore and are widely distributed across the subarctic North Pacific (Hayes et al. 2012). This pattern is consistent with high seas distributions of other West Coast steelhead populations (Myers 2018).

Climate Effects on Abundance and Distribution

Central California Coast steelhead ranked high in exposure due to ***high*** scores for *sea surface temperature*, *sea level rise*, and *flooding*, in addition to *ocean acidification*. Increased variability in winter flows may lead to increased risk of redd scour in flood years and lack of attraction flows in drought years. Sea level rise is an important threat

because of heavy reliance on lagoon habitat for juvenile rearing. Other predicted exposure problems faced by this DPS included increases in *stream temperature* and *summer water deficit*, both of which were ranked ***moderate***.

Overall sensitivity was ranked ***moderate*** for Central California Coast steelhead, but 35% of bootstrapped samples ranked ***high***. This DPS was ranked ***moderate*** at both the *estuary* and *freshwater juvenile stages* because of its extended periods in both environments. Fish from this DPS reside 1-3 years in fresh water and often an entire year in lagoons. During these life stages, steelhead from this DPS are very sensitive to water quality and habitat access.

Extrinsic Factors

For Central California Coast steelhead, risk attributes related to climate change are superimposed on existing threats, such as dams and other impassable barriers, water diversions, agricultural development, urbanization, logging practices, and hatchery influence (Busby et al. 1996). These combined risk factors increase the likelihood of rapid extirpation as time passes without dramatic action to protect and enhance habitats.

There is no directed commercial or recreational fishing for Central California Coast steelhead in the ocean. However, recreational steelhead fishing occurs in several rivers within the range of this DPS. Regulations prohibit retention of natural-origin Central California Coast steelhead in these fisheries; however, retention of up to two hatchery-origin steelhead per day is allowed. Incidental mortality from these recreational fisheries is not well documented (Spence 2016). For these reasons, Central California Coast steelhead ranked ***high*** in the *other stressors* attribute.

Central California Coast steelhead is listed as threatened under the U.S. Endangered Species Act. Quiñones and Moyle (2014) rated this DPS as “highly vulnerable” to climate change as a result of its low population abundance, greatly reduced and fragmented stream flows, and highly altered watersheds.

Adaptive Capacity

Adaptive capacity for Central California Coast steelhead was ranked ***moderate*** because this DPS presumably benefits from some buffering by rainbow trout populations in the headwaters of its natal streams. These populations can produce individuals that go out to sea and present a potential source of extensive life history variation. If conditions become unfavorable for the anadromous type, this DPS would likely shift towards a higher proportion of resident fish.

Literature Cited

Bond, M. H., P. A. H. Westley, A. H. Dittman, D. Holecek, T. Marsh, and T. P. Quinn. 2017. Combined effects of barge transportation, river environment, and rearing location on straying and migration of adult Snake River fall-run Chinook salmon. Trans. Am. Fish. Soc. 146:60-73.

Busby, P. J., T. C. Wainwright, G. J. Bryant, L. J. Lierheimer, R. S. Waples, F. W. Waknitz, and I. V. Lagomarsino. 1996. Status review of west coast steelhead from Washington, Idaho, Oregon, and California. U.S. Dep. Commerce NOAA Tech. Memo. NMFS-NWFSC-27.

Hayes, S. A., M. H. Bond, C. V. Hanson, E. V. Freund, J. J. Smith, E. C. Anderson, A. J. Ammann, and R. B. MacFarlane. 2008. Steelhead growth in a small central California watershed: upstream and estuarine rearing patterns. Trans. Am. Fish. Soc. 137:114-128.

Hayes, S. A., M. H. Bond, B. K. Wells, C. V. Hanson, A. W. Jones, and R. B. MacFarlane. 2012. Using archival tags in infer habitat use of central California steelhead and coho salmon. Pages 471-492 *in* J. McKenzie, B. Parsons, A. Seitz, R. Kopf, M. Mesa, and Q. Phelps, editors. Advances in fish tagging and marking technology. American Fisheries Society Symposium 76.

Leidy, R. A. 2007. Ecology, assemblage structure, distribution, and status of fishes in streams tributary to the San Francisco estuary, California. 530, San Francisco Estuary Institute Contribution No. 530, Richmond, CA.

Moyle, P. B., R. Lusardi, P. Samuel, and J. Katz. 2017. State of the salmonids: Status of California's emblematic fishes 2017. Center for Watershed Sciences, University of California, Davis and California Trout, San Francisco. Available at http://caltrout.org/sos/.

Myers, K. W. 2018. Ocean ecology of steelhead. Page 1090 *in* R. J. Beamish, editor. The ocean ecology of Pacific salmon and trout. American Fisheries Society, Bethesda.

Osterback, A.-M. K., C. H. Kern, E. A. Kanawi, J. M. Perez, and J. D. Kiernan. 2018. The effects of early sandbar formation on the abundance and ecology of coho salmon (*Oncorhynchus kisutch*) and steelhead trout (*Oncorhynchus mykiss)* in a central California coastal lagoon. Can. J. Fish. Aquat. Sci. 75:2184-2197.

Quiñones, R. M., and P. B. Moyle. 2014. Climate change vulnerability of freshwater fishes in the San Francisco Bay Area. San Francisco Est. Water Sci. 12.

Shapovalov, L., and A. C. Taft. 1954. The life histories of the steelhead rainbow trout (*Salmo gairdneri gairdneri*) and silver salmon (*Oncorhynchus kisutch*) with special reference to Waddell Creek, California and recommendations regarding their management. Calif Dep. Fish Game Fish Bull 98:275 pp.

Spence, B. C. 2016. North-Central California Coast Recovery Domain.*in* B. C. Williams, D. A. Spence, D. Boughton, R. C. Johnson, N. Crozier, M. Mantua, M. R. O'Farrell, and S. Lindley, editors. Viability assessment for Pacific salmon and steelhead listed under the Endangered Species Act: Southwest. U.S. Dep. Commerce NOAA Tech. Memo. NMFS-SWFSC-564.

Northern California steelhead

Overall vulnerability—Moderate (94% Moderate, 6% High)
Biological sensitivity—Moderate (94% Moderate, 6% High)
Climate exposure—High (100% High)
Adaptive capacity—High (2.4)
Data quality—58% of scores ≥ 2

	Northern California Steelhead	Expert Scores	Data Quality	Expert Scores Plots (Portion by Category)
Sensitivity attributes	Early life history	1.9	2.3	
	Juvenile freshwater stage	2.7	2.3	
	Estuary stage	2.7	1.8	
	Marine stage	2.4	1.8	
	Adult freshwater stage	2.2	2.3	
	Cumulative life-cycle effects	1.9	2	
	Hatchery influence	2.7	1.8	
	Other stressors	2.9	1.8	
	Population viability	2.6	2.3	
	Ocean acidification sensitivity	1.9	0.5	
	Sensitivity Score	Moderate		
Exposure variables	Stream temperature	2.7	2.3	
	Summer water deficit	2.5	2	
	Flooding	3.1	1.8	
	Hydrologic regime	1.5	2.3	
	Sea level rise	2.8	2	
	Sea surface temperature	3.4	2	
	Ocean acidification exposure	4	3	
	Upwelling	2.5	1.5	
	Ocean currents	1.9	1	
	Exposure Score	High		
Overall Vulnerability Rank		Moderate		

Low
Moderate
High
Very High

Life History Synopsis

Northern California steelhead adults exhibit both summer- and winter-run migration timing. In larger watersheds such as the Mad and Eel River, freshwater entry for winter-run fish can occur as early as September or October. In smaller watersheds, some of which are subject to bar formation, freshwater entry typically begins in December and continues into April or May (Busby et al. 1996). Neither flow nor temperature is generally problematic for winter-run adults. Summer-run populations migrate primarily from April to June or July (Moyle et al. 2008). Migration distances range from a few kilometers in systems such as Redwood Creek to more than 250 km in the Middle Fork Eel River, and distances were even longer prior to dam construction.

Summer-run adults depend on cold-water refuges that often occur at tributary junctions or in thermally stratified pools (Nielsen et al. 1994; Moyle et al. 2008).

Age at maturity varies considerably within and among populations of Northern California steelhead. The predominate life history of winter-run spawners includes 2 years in fresh water and 1-2 years at sea; however, fish may spend anywhere from 1-3 years in fresh water and up to 3 years at sea (Busby et al. 1996). Additionally, adults may survive to spawn a second or third time. Progeny of summer-run adults remain in fresh water 1-3 years, with 2 years being most common, and spend 1-3 years at sea, with 1-2 years being most common.

Non-anadromous individuals add to the suite of life histories in this DPS. Additionally, the DPS includes a "half-pounder" life history where fish migrate to sea for 2-4 months, return to fresh water to overwinter in non-reproductive condition, and then return back to sea. The "half-pounder" type is found in the Eel and (possibly) Mattole Basins (Snyder 1925; Moyle et al. 2008). During marine residence, steelhead moves rapidly offshore and is widely distributed across the subarctic North Pacific (Myers 2018). Juvenile steelhead typically move rapidly through estuarine habitats (Hayes and Kocik 2014; Myers 2018). However, for rivers in this DPS, ocean access can be intermittently blocked by barrier sandbars, forcing fish to inhabit lagoons until ocean access is restored (Hayes and Kocik 2014).

Climate Effects on Abundance and Distribution

In general, flood risk exposure ranked higher for DPSs in California than for those in the Pacific Northwest. Climate-related concerns from flooding include redd scour during high flows and deposition of fine sediments in spawning gravels. Risk of redd scour is exacerbated when large wood and other structural elements are lacking. Change in rainfall intensity and frequency and shifts in the timing of peak flows will affect the risk of both red scour and sediment deposition. Despite these potential risks, Northern California steelhead ranked ***low*** in sensitivity during *early life history* (egg stage), likely due to spawn timing that occurs somewhat later than peak flooding.

At present, low flow conditions during spring can result from low snowpack or low spring precipitation. Such flow conditions can hinder migration of adult summer steelhead, causing them to over-summer in suboptimal (i.e., warmer) habitats lower in the watershed. This behavior has been observed in Northern California steelhead in the Van Duzen and Middle Fork Eel Rivers (S. Thompson and S. Harris, CDFW, personal communication). Summer-run steelhead were considered to have higher vulnerability to climate change in the California multi-species recovery plan because of additional exposure to high summer stream temperatures.

Northern California steelhead ranked ***moderate*** in exposure to *stream temperature* and in sensitivity at the *juvenile freshwater stage*. Sensitivity of steelhead was ranked

lower than that of Chinook and coho because steelhead tends to use habitat over a broader range of temperatures than these other species (Myrick and Cech Jr 2001).

Steelhead uses lagoons extensively (Zedonis 1992; Cannatta 1998; Wallace and Allen 2007, 2015), and thus is likely sensitive to changes in lagoon dynamics. Poor water quality (high temperature, low dissolved oxygen, high salinity) can limit use of lagoons in some watersheds and at certain times of the year (J. Kiernan, NMFS SWFSC, unpublished data). Lagoon water quality is dictated by complex interactions between stream flow, over-bar exchange of marine water, breaching dynamics of sand bars, wind, and other factors. These processes affect the relative quantity of fresh and saline waters and the degree of mixing, which in turn affects salinity-driven stratification of the water column, with the potential to create anoxic conditions (Atkinson 2010).

In extreme cases, mixing of anoxic waters that occurs with breaching of sand bars has been known to cause fish kills (Smith 2009). However, there was uncertainty in how climate change would affect these dynamics because of the interaction between sea level rise, air temperature rise, and change in precipitation (see additional discussion of climate effects for *Central California Coast coho*). Once steelhead smolts enter the ocean, they tend to move offshore relatively quickly, heading primarily in a northward direction (Hayes and Kocik 2014). In sum, Northern California steelhead sensitivity was ranked ***moderate*** in the *estuary stage*, with ***moderate*** exposure to *sea level rise*.

However, this DPS was rated ***high*** for exposure to change in *sea surface temperature* because changes in temperature across the entire marine range of this species are projected to exceed two standard deviations. In exposure to *ocean acidification*, Northern California steelhead was rated ***very high*** because it exceeded 14 standard deviations across the marine range.

Overall the ***moderate*** vulnerability score for this DPS reflected long periods of exposure to change in both the freshwater and marine environments (multiple years per juvenile). For winter-run steelhead, these periods were tempered by moderate sensitivity stemming from tolerance of warm conditions, reproductive timing that avoids peak temperatures, and the ability to wait for intermittent precipitation events. Increased summer warming would likely reduce or eliminate some populations of the summer-run type due to loss of summer habitat. Adults residing in freshwater during summer are likely at higher risk within this DPS.

Extrinsic Factors

Northern California steelhead ranked ***moderate*** in sensitivity to *hatchery influence*. Mad River Hatchery operates the only active hatchery program in the geographical range of this DPS. However, Mad River broodstock was established with out-of-basin fish, primarily from the Eel River, and this hatchery population is not considered part of the DPS. Outbreeding between hatchery and wild steelhead in the Mad River is considered a significant risk factor, as in some years hatchery-origin

steelhead may constitute more than 60% of spawners in the Mad River and its tributaries (Spence 2016).

Northern California steelhead is listed as threatened under the U.S. Endangered Species Act, and was therefore ranked ***moderate*** for *population viability* (Spence 2016). Its sensitivity to *other stressors*, including fisheries and invasive species, was ranked ***moderate***. There is no directed commercial or recreational ocean fishing on natural-origin stocks in this DPS, and ocean harvest is thought to be relatively rare. However, recreational steelhead fishing occurs in many rivers within this DPS. While regulations prohibit retention of natural-origin Northern California steelhead in these fisheries, retention of hatchery-origin steelhead is allowed. Incidental mortality from these recreational fisheries is poorly documented (Spence 2016). Water diversion, land-use practices, pesticides, invasive species (striped bass and pikeminnow), and dams on the Eel and Mad Rivers have been identified as extrinsic threats (Moyle et al. 2008; NMFS 2015).

For a majority of Northern California steelhead populations, as well as for the Central California Coast steelhead and California Coastal Chinook DPSs, the *Multispecies Recovery Plan* assessed the quality and extent of estuary and lagoon rearing habitats. Estuary and lagoon habitats were considered in poor condition compared to other habitat components.

Adaptive Capacity

Northern California steelhead was ranked ***high*** in adaptive capacity because of its substantial life-history diversity, which includes both winter- and summer-run types, as well as a wide age range at smolt migration and maturation. Although other coastal steelhead have similar life histories, this DPS benefits from less xeric ecosystems than other California steelhead, which might provide Northern California steelhead with a greater buffer from extreme conditions with climate change.

Literature Cited

Atkinson, K. A. 2010. Habitat conditions and steelhead abundance and growth in a California lagoon. San Jose State University, San Jose, CA.

Busby, P. J., T. C. Wainwright, G. J. Bryant, L. J. Lierheimer, R. S. Waples, F. W. Waknitz, and I. V. Lagomarsino. 1996. Status review of west coast steelhead from Washington, Idaho, Oregon, and California. U.S. Dep. Commerce NOAA Tech. Memo. NMFS-NWFSC-27.

Cannatta, S. P. 1998. Observations of steelhead trout (*Oncorhynchus mykiss*), coho salmon (*O. kisutch*) and water quality of the Navarro River Estuary/Lagoon May 1997 to December 1997: Draft Report. Humboldt State University Foundation, Arcata, CA.

Hayes, S. A. and J. F. Kocik. 2014. Comparative estuarine and marine migration ecology of Atlantic salmon and steelhead: blue highways and open plains. Rev. Fish Biol. Fish. 24:757-780.

Moyle, P. B., J. A. Israel, and S. E. Purdy. 2008. Salmon, steelhead and trout in California: status of an emblematic fauna. Center for Watershed Sciences, University of California, Davis, CA

Myers, K. W. 2018. Ocean ecology of steelhead. Page 1090 *in* R. J. Beamish, editor. The ocean ecology of Pacific salmon and trout. American Fisheries Society, Bethesda.

Myrick, C. A., and J. J. Cech Jr 2001. Temperature effects on Chinook salmon and steelhead: a review focusing on California's Central Valley populations. Bay Delta Modeling Forum Technical Publication 01-1, Davis, CA

Nielsen, J. L., L. T.E., and O. V. 1994. Thermally stratified pools and their use by steelhead in northern California streams. Trans. Am. Fish. Soc. 123:613-626.

NMFS (National Marine Fisheries Service). 2015 Coastal multi-species recovery plan: California Coastal Chinook Salmon ESU (Draft), California Coastal Chinook Salmon ESU, Northern California Steelhead ESU and Central California Coast Steelhead ESU. National Marine Fisheries Service, West Coast Region. Portland, OR

Smith, K. A. 2009. Inorganic chemical oxygen demand of re-suspended sediments in a bar bound lagoon. M.S. Thesis, San Jose University, San Jose, CA.

Snyder, J. O. 1925. The half pounder of Eel River, a steelhead trout. Calif Fish Game 11:49-55.

Spence, B. C. 2016. North-Central California Coast Recovery Domain.*in* B. C. Williams, D. A. Spence, D. Boughton, R. C. Johnson, N. Crozier, M. Mantua, M. R. O'Farrell, and S. Lindley, editors. Viability assessment for Pacific salmon and steelhead listed under the Endangered Species Act: Southwest. U.S. Dep. Commerce NOAA Tech. Memo. NMFS-SWFSC- 564.

Wallace, M. and S. Allen. 2007. Juvenile salmonid use of the tidal portions of selected tributaries to Humboldt Bay, California. California Dep.Fish and Game, Fisheries Restoration Grants Program Grant P0410504.

Wallace, M. and S. Allen. 2015. Juvenile salmonid use and restoration assessment of the tidal portions of selected tributaries to Humboldt Bay, California, 2011-2012. California Dep.Fish and Wildlife, Fish. Admin. Rep. No. 2015-02.

Zedonis, P. A. 1992. The biology of the juvenile steelhead (*Oncorhynchus mykiss*) in the Mattole River Estuary/Lagoon, California. Humbolt State University, Arcata, CA.

Lower Columbia River steelhead

Overall vulnerability—Moderate (2% Low, 92% Moderate, 6% High)
Biological sensitivity—Moderate (3% Low, 93% Moderate, 3% High)
Climate exposure—High (98% High, 2% Very high)
Adaptive capacity—High (2.3)
Data quality—84% of scores ≥ 2

	Lower Columbia River Steelhead	Expert Scores	Data Quality	Expert Scores Plots (Portion by Category)
Sensitivity attributes	Early life history	1.5	2.8	
	Juvenile freshwater stage	2.1	2.8	
	Estuary stage	1.7	2.3	
	Marine stage	2	2	
	Adult freshwater stage	1.6	2.8	
	Cumulative life-cycle effects	1.8	2	
	Hatchery influence	3.2	2.5	
	Other stressors	2.7	2.5	
	Population viability	2.5	2.8	
	Ocean acidification sensitivity	1.9	1.8	
	Sensitivity Score	Moderate		
Exposure variables	Stream temperature	3.4	2.5	
	Summer water deficit	2.6	2	
	Flooding	2	2	
	Hydrologic regime	1.7	2.8	
	Sea level rise	1.8	2	
	Sea surface temperature	3.2	2.3	
	Ocean acidification exposure	3.9	3	
	Upwelling	1.6	1.8	
	Ocean currents	1.8	1	
	Exposure Score	High		
Overall Vulnerability Rank		Moderate		

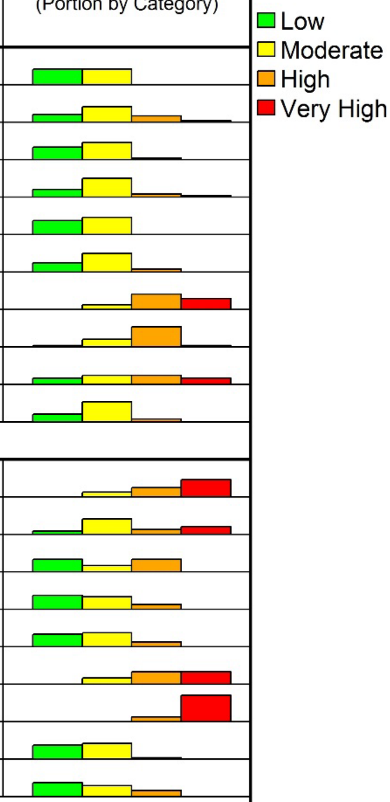

Life History Synopsis

Lower Columbia River steelhead adults migrate year-round, but are generally classified as summer- or winter-run types. Migration is generally from December to April for the winter run and from April to November for the summer run (Busby et al. 1996). Spawning in both runs occurs predominantly in winter and spring, when temperatures generally are not severe. Incubation and early rearing occurs in late spring, at temperatures generally below critical limits (Wade et al. 2013). Rearing conditions do not appear to be limited by temperature at present (Busby et al. 1996; Wade et al. 2013).

Steelhead may spend multiple years in fresh water as juveniles, increasing sensitivity to environmental conditions at the freshwater juvenile stage relative to other

salmonid species. Nonetheless, the panel considered overall evidence for tolerance of this DPS to a broad range of temperatures, leading to a sensitivity score of ***low*** for the *juvenile freshwater stage*. Although low, sensitivity for this DPS was scored slightly higher at the juvenile than at other life history stages. Fish from this DPS spend little time in the estuary as they migrate directly to marine waters (Weitkamp et al. 2015).

Lower Columbia River steelhead smolts generally exhibit early ocean migratory patterns that limit their exposure to potential climate change effects. Like most West Coast steelhead populations, juvenile migrants in this DPS exhibit rapid westward movement, indicative of an offshore distribution. This marine distribution extends across the subarctic Pacific Ocean (Myers 2018), limiting exposure to nearshore conditions such as changes in upwelling or sea level rise (Daly et al. 2014; Van Doornik et al. 2019). Spawning migration distances are also relatively short, limiting exposure of adult fish to freshwater threats, and there are no effects of mainstem dams for this DPS except for the few populations above Bonneville Dam.

In the Lower Columbia River, altered stream and riparian habitats, including loss of side-channel rearing areas, are key impacts affecting each population, especially in downstream reaches. Several populations have significant habitat area blocked by tributary dams (NMFS 2013).

Climate Effects on Abundance and Distribution

Lower Columbia River steelhead sensitivity ranks were ***moderate*** overall, reflecting substantial exposure to changes in the freshwater environment tempered by tolerance to warm conditions and reproductive timing that avoids peak temperatures.

For Lower Columbia River steelhead, exposure to *ocean acidification* ranked ***very high***, as it did for all species in this assessment. Very high scores for this attribute resulted from the strong magnitude of expected pH change, the broad spatial extent of ocean acidification, and the relatively high certainty in the direction of change. Exposure of this DPS was also ranked ***high*** for sea *surface temperature*, reflecting the broad spatial extent of this attribute. This DPS also ranked ***very high*** in exposure to *stream temperature* and ***moderate*** in exposure to *summer water deficit*.

Lower Columbia River steelhead ranked ***low*** in exposure to nearshore attributes, since juveniles tend to spend less time in the nearshore environment and migrate offshore relatively quickly. Nearshore exposure attributes for which this DPS ranked ***low*** included *sea level rise*, *upwelling*, and *ocean currents*.

Wade et al. (2013) found that relative to other Pacific Northwest steelhead, Lower Columbia River steelhead had ***moderate*** exposure to expected changes in *stream temperature* and ***high*** exposure to changes in flow. Lower Columbia River steelhead was

expected to have high sensitivity scores based on its habitat condition and threatened population status.

Lower Columbia River steelhead juveniles migrate rapidly through the estuary in late spring and experience a short window of exposure to estuarine influence relative to other species (Fresh et al. 2005). Therefore, exposure was ***low*** for *sea level rise* effects on the estuary. However, these juveniles use the estuary more extensively than many other juvenile steelhead, and therefore this DPS had slightly higher exposure scores for sea level rise than other Oregon and Washington stocks.

Extrinsic Factors

Lower Columbia River steelhead ranked ***high*** for *hatchery influence*, and ***moderate*** for both *other stressors* and current *population viability*: most hatcheries are production hatcheries. More specifically, among populations for which data are available, five of nine winter-runs had more than 40% hatchery adults in the spawning population, and 3 of 5 summer-runs had more than 25% hatchery adults (Good et al. 2005).

The Lower Columbia River was discussed as an area with high and diverse human impacts, which led to the score of ***moderate*** for *other stressors*. These other stressors include potential loss of floodplain habitats and invasive species (NMFS 2013). Dams limit or block access to the higher elevation spawning habitat that would have otherwise provided cold-water refuge from climate change (Myers et al. 2006; Ford et al. 2015). In the Lower Columbia River, except for the Wind River summer-run population, all populations of steelhead have moderate to very low probabilities of persistence (Good et al. 2005; NMFS 2013). Both abundance and productivity are low (Good et al. 2005).

Adaptive Capacity

Lower Columbia River steelhead can tolerate a broad range of temperatures and has a very flexible life history. However, this DPS may have to shift migration or spawn timing in response to hydrologic regime change effects (Wade et al. 2013). Overall, this DPS ranked ***high*** in adaptive capacity.

Literature Cited

Busby, P. J., T. C. Wainwright, G. J. Bryant, L. J. Lierheimer, R. S. Waples, F. W. Waknitz, and I. V. Lagomarsino. 1996. Status review of west coast steelhead from Washington, Idaho, Oregon, and California. U.S. Dep. Commerce NOAA Tech. Memo. NMFS-NWFSC-27.

Daly, E. A., R. D. Scheurer, R. D. Brodeur, L. A. Weitkamp, B. R. Beckman, and J. A. Miller. 2014. Juvenile steelhead distribution, migration, feeding, and growth in the Columbia River estuary, plume, and coastal waters. Mar. Coast. Fish. 6:62-80.

Ford, M. J., K. Barnas, T. Cooney, L. G. Crozier, M. Diaz, J. J. Hard, E. E. Holmes, D. M. Holzer, R. G. Kope, P. W. Lawson, M. Liermann, J. M. Myers, M. Rowse, D. J. Teel, D. M. V. Doornik, T. C. Wainwright, L. A. Weitkamp, and M. Williams. 2015. Status review update for Pacific salmon and steelhead listed under the Endangered Species Act: Pacific Northwest. National Marine Fisheries Service, Northwest Fisheries Science Center, Seattle Available from www.nwfsc.noaa.gov/publications/scipubs/searchdoc.cfm.

Fresh, K. L., L. L. Casillas, L. L. Johnson, and D. L. Bottom. 2005. Role of the estuary in the recovery of Columbia River basin salmon and steelhead: an evaluation of the effects of selected factors on salmonid population viability. U.S. Dep. Commerce NOAA Tech. Memo. NMFS-NWFSC-69.

Good, T. P., W. R.S., and A. P. 2005. Updated status of federally listed ESUs of West Coast salmon and steelhead. U.S. Dep.Commerce, NOAA Tech. Memo. NMFS-NWFSC-66.

Myers, J., C. Busack, D. Rawding, A. Marshall, D. J. Teel, D. M. Van Doornik, and M. Maher. 2006. Historical population structure of Pacific salmonids in the Willamette River and Lower Columbia River Basins. U.S. Dep. Commerce NOAA Tech. Memo. NMFS-NWFSC-73.

Myers, K. W. 2018. Ocean ecology of steelhead. Page 1090 *in* R. J. Beamish, editor. The ocean ecology of Pacific salmon and trout. American Fisheries Society, Bethesda.

NMFS (National Marine Fisheries Service). 2013. ESA recovery plan for lower Columbia River coho salmon, lower Columbia River Chinook salmon, Columbia River chum salmon, and lower Columbia River steelhead. National Marine Fisheries Service, West Coast Region. Portland, OR.

Van Doornik, D., D. Kuligowski, C. A. Morgan, B. Burke, and T. R. Seamons. 2019. Insights, from genetic analyses, into stock-specific distribution of juvenile steelhead (*Oncorhynchus mykiss*) from the Columbia River during early marine migration. Fish. Bull. NOAA: 117:97-106

Wade, A., T. Beechie, E. Fleishman, N. Mantua, H. Wu, J. Kimball, D. M. Stoms, and J. Stanford. 2013. Steelhead vulnerability to climate change in the Pacific Northwest. J. Appl. Ecol. 50:1093-1104.

Weitkamp, L. A., D. J. Teel, M. Liermann, S. A. Hinton, D. M. Van Doornik, and P. J. Bentley. 2015. Stock-specific size and timing at ocean entry of Columbia River juvenile Chinook salmon and steelhead: Implications for early ocean growth. Mar. Coast. Fish. 7:370-392.

Upper Willamette River steelhead

Overall vulnerability—High (47% Moderate, 51% High, 2% Very high)
Biological sensitivity—High (51% High, 49% Moderate)
Climate exposure—High (95% High, 5% Very high)
Adaptive capacity—Moderate (1.8)
Data quality—84% of scores ≥ 2

	Upper Willamette River Steelhead	Expert Scores	Data Quality	Expert Scores Plots (Portion by Category)
Sensitivity attributes	Early life history	1.5	2.8	
	Juvenile freshwater stage	2.4	2.8	
	Estuary stage	1.6	2.3	
	Marine stage	2	2	
	Adult freshwater stage	1.6	2.8	
	Cumulative life-cycle effects	2.5	2	
	Hatchery influence	3	2.5	
	Other stressors	3.2	2.5	
	Population viability	2.4	2.8	
	Ocean acidification sensitivity	1.9	1.8	
	Sensitivity Score	High		
Exposure variables	Stream temperature	3.7	2.5	
	Summer water deficit	2.9	2	
	Flooding	2.5	2	
	Hydrologic regime	2.1	2.8	
	Sea level rise	1.4	2	
	Sea surface temperature	3.2	2.3	
	Ocean acidification exposure	3.9	3	
	Upwelling	1.8	1.8	
	Ocean currents	1.8	1	
	Exposure Score	High		
Overall Vulnerability Rank		High		

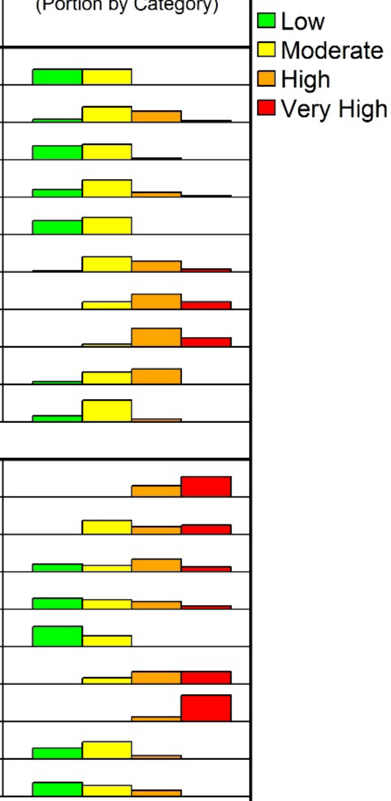

Life History Synopsis

Upper Willamette River steelhead is an ocean-maturing phenotype with adults entering fresh water from late winter to early spring (March-April). Adults spawn within a few weeks of freshwater entry (April-June), with eggs incubating in stream gravels through mid-summer (Busby et al. 1996). Juveniles generally spend 2-3 years in fresh water prior to migration, and in the Willamette and other systems, some individuals complete the life cycle entirely within fresh water (Kendall et al. 2015). Migrating steelhead juveniles are believed to spend little time in the Columbia River estuary (Weitkamp et al. 2015) and move directly offshore to feeding areas across the subarctic Pacific Ocean (Light et al. 1989; Daly et al. 2014; Van Doornik et al. 2019).

Climate Effects on Abundance and Distribution

For Upper Willamette River steelhead, two of the three contributors to ***high*** exposure scores were attributes of the marine environment: *ocean acidification* and *sea surface temperature*. Sensitivity of upper Willamette River steelhead to *ocean acidification*, however, was ranked just below ***moderate***. Similarly, sensitivity of upper Willamette River steelhead to *sea surface temperature* was ranked ***moderate***. However, data quality scores for sensitivity attributes indicated that information is lacking.

Stream temperature was the most important freshwater exposure factor for this DPS because steelhead juveniles generally rear for one or more years in fresh water before migrating (Busby et al. 1996). Of the four recognized populations of winter steelhead in the Upper Willamette River Basin (Myers et al. 2006), all inhabit rivers that drain the west slopes of the Cascade Range. However, only the North Santiam River extends to the high Cascades region, where snow melt and ground water contribute significantly to stream flows (Chang et al. 2018). Access to much of this historical spawning habitat in the North Santiam is blocked by impassable dams (Ford et al. 2015). Studies of steelhead in other basins have shown warmer summer temperatures associated with development of anadromy, whereas a resident life history type was more prevalent in streams with colder summer water temperatures (McMillan et al. 2012). In contrast, the distribution of native steelhead in the upper Willamette Basin is not cleanly associated with gradients in summer stream temperature.

In the Willamette River Basin, native late-winter migrating populations occur in watersheds draining the Cascade Mountains on the eastern edge of the basin. Interestingly, native steelhead populations are not believed to inhabit the upper extremes of the basin, nor the tributaries of its western edge, which drain the Coastal Range, although steelhead are known to migrate much longer distances to reach spawning grounds in other watersheds (Busby et al. 1996). In other systems, longer steelhead migrations are associated with adult returns in summer. Thus, the late winter entry of Willamette River steelhead, which is believed to be an adaptation to allow historical passage over Willamette Falls (Busby et al. 1996), may pose a temporal constraint on the migration distance that native steelhead can attain prior to spawning. Such time constraints may be more important than temperature in terms of the distribution of steelhead in the Willamette Basin.

Extrinsic Factors

One of the most important attributes driving sensitivity of upper Willamette River steelhead was *hatchery influence*, which was ranked ***high***. Though hatchery propagation of this lineage is no longer occurring, there are established populations of non-native winter-run steelhead, active hatchery summer-run steelhead production, and feral natural production of non-native summer- and winter-run steelhead in the basin (Busby et al.

1996; Van Doornik et al. 2015). There may also be a legacy of stocking non-native hatchery rainbow trout to support recreational harvest in reservoirs and rivers.

In the Willamette Basin, historically introduced, non-native steelhead with earlier winter run-timing have established to varying degrees in west-side tributaries that drain the Oregon Coast Range (Busby et al. 1996). Recent analyses indicate these non-native steelhead are genetically distinct from native populations to the east (Van Doornik et al. 2015). Results of this work also indicate that native steelhead and resident rainbow trout in the upper Willamette Basin are distinctive, yet form a definable lineage relative to non-native winter and non-native summer-run steelhead. Genetic analyses of native rainbow trout and native winter steelhead in the basin does not indicate a substantial level of introgression (Van Doornik et al. 2015). Genetic analysis of fish from the nearby Sandy River suggests that non-native hatchery rainbow trout do not readily hybridize with native winter steelhead (Winans et al. 2018).

Ecological effects from this suite of non-native, hatchery-derived populations of summer and winter steelhead and rainbow trout are a concern, though not well understood, partly because such effects can be extremely difficult to rigorously quantify (Weber and Fausch 2003). This applies to largely unquantified direct and indirect effects, such as attraction of very large numbers of anglers to harvest non-native steelhead and trout (and other salmonids, such as hatchery spring Chinook). These popular river and reservoir fisheries may lead to incidental mortality of native juvenile and adult winter steelhead. Such effects can be magnified in locations where access of native fish is constrained by the presence of impassible dams (Sheer and Steel 2006).

In addition to non-native salmonids, there are growing numbers of non-native fishes spreading throughout the basin, and the effects of these species may equal or exceed threats from non-native salmonids (Williams 2014). Most of these species are tolerant of much warmer water temperatures than salmonids, and climate warming could lead to their increased dominance over, or consumption of, upper Willamette River steelhead (Carey et al. 2011; Lawrence et al. 2012).

The Willamette River Basin supports the highest human population densities in the state of Oregon, and there are a variety of growing threats related to urbanization and water quality. These threats include unknown levels of pollution from a host of personal care and pharmaceutical products that are difficult to detect, often unregulated, and biologically active at extremely low concentrations (Yeakley et al. 2014).

Therefore, *other stressors* (which include non-native fish species and human caused habitat degradation) was ranked ***high***, and was the highest-ranking climate sensitivity attribute for upper Willamette River steelhead.

Adaptive Capacity

Winter steelhead in the upper Willamette River have an extended freshwater residency, and the majority of naturally produced smolts migrate during their second spring (Keefer and Caudil 2010). Although it is possible for winter steelhead to complete the life cycle as resident *O. mykiss*, there is little information on the frequency of this life history trajectory, and it is not thought to be common among naturally produced fish. While juvenile winter steelhead will redistribute themselves during freshwater residency, cooler, higher-elevation rearing habitat in tributary basins is either not present (Molalla and Calapooia River), inaccessible due to impassable dams (North Santiam, Brietenbush, and Middle Santiam River), or severely degraded (South Santiam River). There is considerable flexibility in both juvenile migration (Keefer and Caudil 2010) and adult return timing (Jepson et al. 2015) to adapt to temperature extremes. There has been no hatchery supplementation of winter-run steelhead since the late 1990s, and with the exception of hybridization with non-native summer-run and early winter-run steelhead, the genetic integrity of this DPS is thought to be relatively intact (Van Doornik et al. 2015). Upper Willamette steelhead ranked ***moderate*** for adaptive capacity.

Literature Cited

Information summarized in this narrative is provided in greater depth in a 5-year status review update for Upper Willamette Basin steelhead (Ford et al. 2011) and in a literature review by Keefer and Caudil (2010).

Busby, P. J., T. C. Wainwright, G. J. Bryant, L. J. Lierheimer, R. S. Waples, F. W. Waknitz, and I. V. Lagomarsino. 1996. Status review of west coast steelhead from Washington, Idaho, Oregon, and California. U.S. Dep. Commerce NOAA Tech. Memo. NMFS-NWFSC-27.

Carey, M. P., B. L. Sanderson, T. A. Friesen, K. A. Barnas, and J. D. Olden. 2011. Smallmouth bass in the Pacific Northwest: a threat to native species; a benefit for anglers. Rev. Fish. Sci. 19:305-315.

Chang, H., E. Watson, and A. Strecker. 2018. Climate change and stream temperature in the Willamtette River basin: implication for fish habitat. Pages 119-132 *in* H.-S. Jung and B. Wang, editors. Bridging Science and Policy Implication for Managing Climate Extremes. World Scientific Publishing, Toh Tuck Link, Singapore.

Daly, E. A., R. D. Scheurer, R. D. Brodeur, L. A. Weitkamp, B. R. Beckman, and J. A. Miller. 2014. Juvenile steelhead distribution, migration, feeding, and growth in the Columbia River estuary, plume, and coastal waters. Mar. Coast. Fish. 6:62-80.

Ford, M. J., A. Albaugh, K. Barnas, T. Cooney, J. Cowen, J. J. Hard, R. G. Kope, M. M. McClure, P. McElhany, J. M. Myers, N. J. Sands, D. J. Teel, and L. A. Weitkamp. 2011. Status review update for Pacific salmon and steelhead listed under the Endangered Species Act: Pacific Northwest. U.S. Dep.Commerce NMFS-NWFSC-113.

[

Ford, M. J., K. Barnas, T. Cooney, L. G. Crozier, M. Diaz, J. J. Hard, E. E. Holmes, D. M. Holzer, R. G. Kope, P. W. Lawson, M. Liermann, J. M. Myers, M. Rowse, D. J. Teel, D. M. V. Doornik, T. C. Wainwright, L. A. Weitkamp, and M. Williams. 2015. Status review update for Pacific salmon and steelhead listed under the Endangered Species Act: Pacific Northwest. National Marine Fisheries Service, Northwest Fisheries Science Center, Seattle. Available from www.nwfsc.noaa.gov/publications/scipubs/searchdoc.cfm.

Jepson, M. A., M. L. Keefer, C. C. Caudill, T. S. Clabough, C. S. Erdman, T. J. Blubaugh, and C. S. Sharpe. 2015. Migratory behavior, run timing, and distribution of radio-tagged adult winder steelhead, summer steelhead, spring Chinook salmon, and Coho salmon in the Willamette River: 2011-2014. Oregon Dep.Fish and Wildlife, Corvallis Research Lab, Corvallis, Oregon.

Keefer, M. L., and C. C. Caudil. 2010. A review of adult salmon and steelhead life history and behavior in the Willamette River basin: identification of knowledge gaps and research needs. University of Idaho College of Natural Resoures, Moscow, ID. Available from http://www.webpages.uidaho.edu/uiferl/pdf%20reports/2010-08_Keefer-Caudill%20WIL%20lit%20review%20(FINAL).pdf.

Kendall, N. W., J. R. McMillan, M. R. Sloat, T. W. Buehrens, T. P. Quinn, G. R. Pess, K. V. Kuzishchin, M. M. McClure, and R. W. Zabel. 2015. Anadromy and residency in steelhead and rainbow trout (*Oncorhynchus mykiss*): a review of the processes and patterns. C. J. Fish. Aquat. Sci. 72:319-342.

Lawrence, D. J., J. D. Olden, and C. E. Torgensen. 2012. Spatiotemporal patterns and habitat associations of smallmouth bass (*Micropterus dolomieu*) invading salmon-rearing habitat. Freshw. Biol. 57:1929-1946.

Light, J. T., C. K. Harris, and R. L. Burgner. 1989. Ocean distribution and migration of steelhead (*Oncorhynchus mykiss*, formerly *Salmo gairdneri*). Fisheries Research Institute, University of Washington, 8912, Seattle, WA.

McMillan, J. R., J. B. Dunham, G. H. Reeves, J. S. Mills, and C. E. Jordan. 2012. Individual condition and stream temperature influence early maturation of rainbow and steelhead trout, *Oncorhynchus mykiss*. Environ. Biol. Fish. 93:343-355.

Myers, J., C. Busack, D. Rawding, A. Marshall, D. J. Teel, D. M. Van Doornik, and M. Maher. 2006. Historical population structure of Pacific salmonids in the Willamette River and Lower Columbia River Basins. U.S. Dep. Commerce NOAA Tech. Memo. NMFS-NWFSC-73.

Sheer, M. B., and E. A. Steel. 2006. Lost watersheds: barriers, aquatic habitat connectivity, and salmon persistence in the Willamette and Lower Columbia River basins. Trans. Am. Fish. Soc. 135:1654-1669.

Van Doornik, D., D. Kuligowski, C. A. Morgan, B. Burke, and T. R. Seamons. 2019. Insights, from genetic analyses, into stock-specific distribution of juvenile steelhead (*Oncorhynchus mykiss*) from the Columbia River during early marine migration. Fish. Bull. NOAA. 117:97-106.

Van Doornik, D. M., D. M. Hess, M. A. Johnson, D. J. Teel, T. A. Friesen, and J. M. Myers. 2015. Genetic population structure of Willamette River steelhead and the influence of introduced stocks. Transactions of the American Fisheries Society 144:150-162.

Weber, E. D., and K. D. Fausch. 2003. Interactions between hatchery and wild salmonids in streams: differences in biology and evidence for competition. Canadian Journal of Fisheries and Aquatic Sciences 60:1018-1036.

Weitkamp, L. A., D. J. Teel, M. Liermann, S. A. Hinton, D. M. Van Doornik, and P. J. Bentley. 2015. Stock-specific size and timing at ocean entry of Columbia River juvenile Chinook salmon and steelhead: Implications for early ocean growth. Marine and Coastal Fisheries 7:370-392.

Williams, J. E. 2014. Habitat relationships of native and non native fishes of the Willamette River, Oregon. M.S. Thesis, Oregon State University, Corvalis, OR.

Winans, G. A., M. B. Allen, J. Baker, E. Lesko, F. Shrier, B. Strobel, and J. Myers. 2018. Dam trout: Genetic variability in *Oncorhynchus mykiss* above and below barriers in three Columbia River systems prior to restoring migrational access. PLoS ONE 13:e0197571.

Yeakley, J. A., K. G. Maas-Hebner, and R. M. Hughes. 2014. Wild salmonids in the urbanizing Pacific Northwest. Springer, New York, NY.

Middle Columbia River steelhead

Overall vulnerability—High (10% Moderate, 90% High)
Biological sensitivity—High (10% Moderate, 90% High)
Climate exposure—High (99% High)
Adaptive capacity—Moderate (2.0)
Data quality—84% of scores ≥ 2

	Middle Columbia River Steelhead	Expert Scores	Data Quality	Expert Scores Plots (Portion by Category)
Sensitivity attributes	Early life history	1.5	2.8	
	Juvenile freshwater stage	2.1	2.8	
	Estuary stage	1.6	2.3	
	Marine stage	2	2	
	Adult freshwater stage	3.1	2.5	
	Cumulative life-cycle effects	2.8	2	
	Hatchery influence	3.2	2.5	
	Other stressors	3.1	2.5	
	Population viability	2.3	2.8	
	Ocean acidification sensitivity	1.9	1.8	
	Sensitivity Score	High		
Exposure variables	Stream temperature	3.1	2.5	
	Summer water deficit	2.9	2	
	Flooding	2.9	2	
	Hydrologic regime	3.1	2.8	
	Sea level rise	1.5	2	
	Sea surface temperature	3.2	2.3	
	Ocean acidification exposure	3.9	3	
	Upwelling	1.6	1.8	
	Ocean currents	1.8	1	
	Exposure Score	High		
Overall Vulnerability Rank		High		

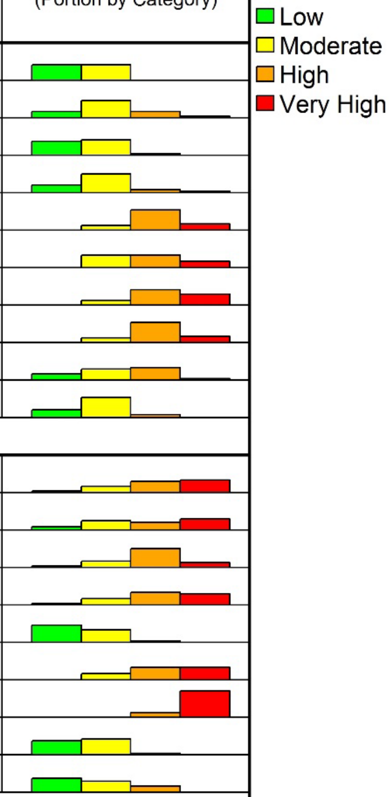

Life History Synopsis

Middle Columbia River steelhead includes both a summer run, or stream-maturing phenotype and a winter-run, ocean-maturing phenotype. In the Columbia River, arrival of adults from this DPS may occur nearly every month of the year, with those that return early spending well over 6 months in fresh water before spawning. Following this period, spawning occurs over a 7-month window, from January to July. Spawn timing varies by phenotype (ocean or freshwater maturing) and ambient conditions (e.g., flow, temperature). Emergence occurs over a smaller window, generally ranging from May through July.

As do juveniles from other steelhead DPSs, Middle Columbia River steelhead juveniles generally spend 2-3 years in fresh water prior to migration, and some individuals complete the life cycle entirely within fresh water (Kendall et al. 2015). Migrating juvenile steelhead are believed to spend little time in the Columbia River estuary (Daly et al. 2014; Weitkamp et al. 2015) and move directly to offshore feeding areas across the subarctic Pacific Ocean (Myers 2018; Van Doornik et al. 2019).

Climate Effects on Abundance and Distribution

Though marine exposure attributes ranks were ***high*** for Middle Columbia River steelhead, the corresponding sensitivity attributes of this species are poorly understood, and this was reflected in the generally low-ranking data quality for both marine and estuarine attributes. Linkages between adult returns and marine conditions have not been extensively evaluated for this DPS, although some inferences can be made from general ocean distribution information and temporal patterns in smolt-to-adult return rates.

Although detailed information on ocean distributions for Columbia River steelhead is not available, past studies suggest that steelhead from Pacific coastal systems generally occur in the Gulf of Alaska and subarctic waters south of the Aleutian Islands (Light et al. 1989). Abdul-Aziz et al. (2011) developed spatially explicit representations of open ocean thermal habitat for steelhead. They found that under a multimodel ensemble average of climate model outputs using the A1B emissions scenario, summer habitat area declined by 36% for the 2080s, with the largest losses in the northeast Pacific Ocean. Wintertime habitat area losses were 2%, with reductions at the southern end of the historical range largely offset by gains in the Bering Sea and Sea of Okhotsk.

Whether a general northward and westward displacement of the most frequently observed thermal open ocean habitat will have substantial impacts on the life-cycle, productivity, or spawning distribution of these steelhead is not known. A recent study of smolt-to-adult return ratios found similarities in annual marine survival patterns, with regional groupings for Puget Sound, British Columbia and coastal Washington and Oregon (Kendall et al. 2015). These groupings suggest that for steelhead, marine/estuarine factors associated with the point of ocean entry may be a more important determinants of year class survival than general conditions in the adult ocean range.

The life stage at which Middle Columbia River steelhead had the highest sensitivity to climate change was the *adult freshwater stage*. Because many adults spend months in fresh water prior to spawning and hold during the warmest temperatures and lowest flows of the year, they may be particularly vulnerable to climate-related impacts to freshwater habitat. This general threat to the summer-run life history contributed to the ***moderate*** rank in *cumulative life cycle effects* for this DPS.

Extrinsic Factors

Exposure to *other stressors* ranked ***high***, and for Middle Columbia River steelhead, these include particular challenges from dams, which limit adult movement up and downstream during summer pre-spawn periods and repeat spawning attempts. Adults are also vulnerable to predators and angling in thermal or flow refuges. Many of these stressors influence adults primarily, but juveniles also face habitat stress. Other stressors likely to be exacerbated in the face of climate change include widespread invasion of non-native, warm-water species (Sanderson et al. 2009) and biological effects from contaminants (Yeakley et al. 2014). *Hatchery influence*, both within and outside of the mid-Columbia, was ranked ***high*** in reducing the resilience of this DPS. Straying of hatchery fish from populations outside of this DPS is a well-known phenomenon. Mid-Columbia River steelhead is listed as threatened under the U.S. Endangered Species Act, but is considered at lower risk than other interior steelhead (Ford et al. 2015).

Adaptive Capacity

Mid-Columbia River steelhead exhibits diverse life histories. Recent work on interactions between anadromous and resident types of *O. mykiss* in the middle Columbia region and throughout the range of steelhead has shown that both types can and often do interbreed (Hard et al. 2015; Kendall et al. 2015), as is the case for many salmonids (Sloat et al. 2014). Local factors such as reproductive isolation in space or time or population sizes of either type may govern how different forms of *O. mykiss* interact. There is good evidence that resident rainbow trout can increase resilience of steelhead, as returning adults of the latter can be traced to resident parents (Kendall et al. 2015). Middle Columbia River steelhead ranked ***moderate*** for overall adaptive capacity.

Literature Cited

Abdul-Aziz, O. I., N. J. Mantua, and K. W. Myers. 2011. Potential climate change impacts on thermal habitats of Pacific salmon (*Oncorhynchus spp.*) in the North Pacific Ocean and adjacent seas. Can. J. Fish. Aquat. Sci. 68:1660-1680.

Daly, E. A., R. D. Scheurer, R. D. Brodeur, L. A. Weitkamp, B. R. Beckman, and J. A. Miller. 2014. Juvenile steelhead distribution, migration, feeding, and growth in the Columbia River estuary, plume, and coastal waters. Mar. Coast. Fish. 6:62-80.

Ford, M. J., K. Barnas, T. Cooney, L. G. Crozier, M. Diaz, J. J. Hard, E. E. Holmes, D. M. Holzer, R. G. Kope, P. W. Lawson, M. Liermann, J. M. Myers, M. Rowse, D. J. Teel, D. M. V. Doornik, T. C. Wainwright, L. A. Weitkamp, and M. Williams. 2015. Status review update for Pacific salmon and steelhead listed under the Endangered Species Act: Pacific Northwest. National Marine Fisheries Service, Northwest Fisheries Science Center, Seattle. Available from www.nwfsc.noaa.gov/publications/scipubs/searchdoc.cfm.

Hard, J. J., J. M. Myers, E. J. Conner, R. A. Hayman, R. G. Kope, G. Lucchetti, A. R. Marshall, G. R. Pess, and B. E. Thomspson. 2015. Viability criteria for steelhead within the Puget Sound distinct population segment. U.S. Dep. Commerce NOAA Tech. Memo. NMFS-NWFSC-129.

Kendall, N. W., J. R. McMillan, M. R. Sloat, T. W. Buehrens, T. P. Quinn, G. R. Pess, K. V. Kuzishchin, M. M. McClure, and R. W. Zabel. 2015. Anadromy and residency in steelhead and rainbow trout (*Oncorhynchus mykiss*): a review of the processes and patterns. Can. J. Fish. Aquat. Sci. 72:319-342.

Light, J. T., C. K. Harris, and R. L. Burgner. 1989. Ocean distribution and migration of steelhead (*Oncorhynchus mykiss*, formerly *Salmo gairdneri*). Fisheries Research Institute, University of Washington, 8912, Seattle, WA.

Myers, K. W. 2018. Ocean ecology of steelhead. Page 1090 *in* R. J. Beamish, editor. The ocean ecology of Pacific salmon and trout. American Fisheries Society, Bethesda.

Sanderson, B. L., K. A. Barnas, and A. M. W. Rub. 2009. Nonindigenous species of the Pacific Northwest: an overlooked risk to endangered salmon? Bioscience 59:245-256.

Sloat, M. R., D. J. Fraser, J. B. Dunham, J. A. Falke, C. E. Jordan, J. R. McMillan, and H. A. Ohms. 2014. Ecological and evolutionary patterns of freshwater maturation in Pacific and Atlantic salmonines. Rev. Fish Biol. Fish. 24:689-707.

Van Doornik, D., D. Kuligowski, C. A. Morgan, B. Burke, and T. R. Seamons. 2019. Insights, from genetic analyses, into stock-specific distribution of juvenile steelhead (*Oncorhynchus mykiss*) from the Columbia River during early marine migration. Fish. Bull. NOAA: 117:97-106

Weitkamp, L. A., D. J. Teel, M. Liermann, S. A. Hinton, D. M. Van Doornik, and P. J. Bentley. 2015. Stock-specific size and timing at ocean entry of Columbia River juvenile Chinook salmon and steelhead: Implications for early ocean growth. Mar. Coast. Fish. 7:370-392.

Yeakley, J. A., K. G. Maas-Hebner, and R. M. Hughes. 2014. Wild salmonids in the urbanizing Pacific Northwest. Springer, New York, NY.

Snake River Basin steelhead

Overall vulnerability—High (9% Moderate, 88% High, 3% Very high)
Biological sensitivity—High (9% Moderate, 91% High)
Climate exposure—High (97% High, 3% Very high)sa
Adaptive capacity—Moderate (1.8)
Data quality—84% of scores ≥ 2

	Snake River Basin Steelhead	Expert Scores	Data Quality	Expert Scores Plots (Portion by Category)
Sensitivity attributes	Early life history	1.5	2.8	
	Juvenile freshwater stage	2.1	2.8	
	Estuary stage	1.6	2.3	
	Marine stage	2	2	
	Adult freshwater stage	3	2.5	
	Cumulative life-cycle effects	2.7	2	
	Hatchery influence	3.3	2.5	
	Other stressors	3.1	2.5	
	Population viability	2.5	2.5	
	Ocean acidification sensitivity	1.9	1.8	
	Sensitivity Score	High		
Exposure variables	Stream temperature	3.5	2.5	
	Summer water deficit	3	2	
	Flooding	2.8	2	
	Hydrologic regime	2.4	2.8	
	Sea level rise	1.4	2	
	Sea surface temperature	3.2	2.3	
	Ocean acidification exposure	3.9	3	
	Upwelling	1.6	1.8	
	Ocean currents	1.8	1	
	Exposure Score	High		
Overall Vulnerability Rank		High		

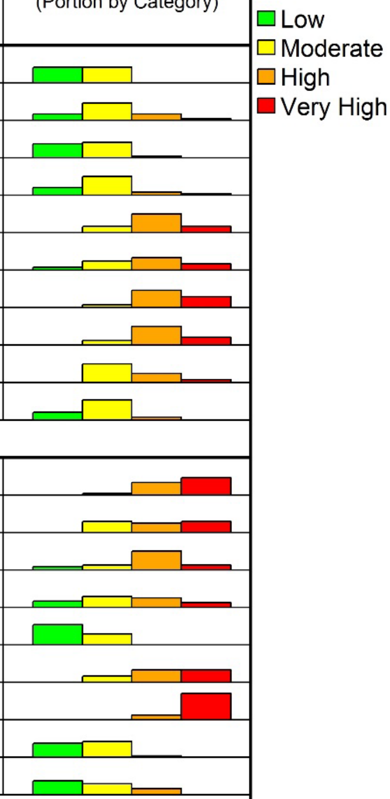

Life History Synopsis

The majority of Snake River steelhead adults enter the Columbia River and migrate upstream in late summer/early fall, with migration peaking from August to October (Robards and Quinn 2002; Copeland et al. 2017). Many stocks hold through winter in mainstem reaches of the Columbia or Snake River and complete migration the following spring (Keefer et al. 2008). Peak spawning generally occurs from April to May, depending on temperature and flow conditions, and spawning tributaries are 800 to 1,500 km upstream from the Columbia River mouth. Depending on spawn timing and temperature, fry emerge from the gravel from May through July.

Age at juvenile migration varies among populations of this DPS, but is generally correlated with elevation and annual stream temperature (Busby et al. 1996). Lower-elevation populations are dominated by 2-year-old juvenile migrants, whereas higher elevation areas tend to produce both 2- and 3-year-olds.

Steelhead juveniles migrate through the Columbia River estuary relatively rapidly in late spring, with a very short window of exposure to estuarine habitats (Fresh et al. 2005; Weitkamp et al. 2015). However, avian predation imposes significant mortality on juveniles during movement through the estuary (Evans et al. 2012, 2016). Once in the ocean, Snake River Basin steelhead generally exhibit rapid westward movement and have an offshore marine distribution across the subarctic Pacific Ocean (Light et al. 1989; Daly et al. 2014; Myers 2018; Van Doornik et al. 2019).

Climate Effects on Abundance and Distribution

Snake River Basin steelhead ranked ***high*** in sensitivity attributes overall, in part because of ***high*** sensitivity at the *adult freshwater stage*. Most populations are subject to high stream temperatures during the upstream migration and pre-spawn holding phases (Wade et al. 2013). Moreover, for populations in Lower Snake River tributaries, the presence of mainstem dams (particularly Lower Granite Dam) may exacerbate straying. Snake River Basin steelhead also ranked ***high*** to ***very high*** in exposure to increased *stream temperature* and *summer water deficit* during the upstream migration and holding periods, indicating ***high*** vulnerability to climate change at the *adult freshwater stage*.

Although detailed information on ocean distributions for this DPS is not available, past studies suggest that steelhead from Pacific coastal systems generally occur in the Gulf of Alaska and in subarctic waters south of the Aleutian Islands (Light et al. 1989). Abdul-Aziz et al. (2011) developed spatially explicit representations of open ocean thermal habitat for steelhead. They found that under a multimodel ensemble average of climate model outputs using the A1B emissions scenario, summer habitat area declined by 36% for the 2080s, with the largest losses in the northeast Pacific Ocean. Wintertime habitat area losses were 2%, with reductions at the southern end of the historical range largely offset by gains in the Bering Sea and Sea of Okhotsk.

Whether a general northward and westward displacement of the most frequently observed thermal open ocean habitat will have substantial impacts on the life-cycle, productivity, or spawning distribution of these steelhead is not known. A recent study of smolt-to-adult survival trends found similar patterns in annual marine survival for stocks within regional groupings for Puget Sound, British Columbia and coastal Washington and Oregon (Kendall et al. 2017). Such patterns suggest that marine/estuarine factors associated with the point of ocean entry may be more important determinants of year-class survival for steelhead than general conditions in the adult ocean range.

Despite ***moderate*** to ***high*** exposure scores for *flooding*, *stream temperature*, and *summer water deficit*, Snake River Basin steelhead ranked ***low*** to ***moderate*** for sensitivity at the *early life history* (egg incubation) and *juvenile freshwater stages*. Sensitivity of egg incubation was rated low because stream temperature and flows are generally well within tolerance limits. Therefore, this DPS is likely somewhat less vulnerable at the egg incubation and juvenile rearing stages. Sensitivity scores were ***low*** to ***moderate*** for the *estuary stage*. Snake River Basin steelhead ranked ***high*** in exposure to *sea surface temperature*, with ***low*** exposure to *ocean currents*, *upwelling*, and *sea level rise*. Sensitivity was ranked ***moderate*** for the *marine stage*.

Extrinsic Factors

High sensitivity scores for *hatchery influence* and *other stressors* contributed to an overall high vulnerability ranking for Snake River Basin steelhead. Both production and conservation hatchery programs are present, and potential for interactions varies considerably across major population groups and populations (Ford et al. 2015; Copeland et al. 2017). Just over one-half of populations in this DPS have relatively low levels of direct hatchery influence, but the potential for such impacts is high for many remaining populations. Populations with little direct hatchery influence represent a wide range of habitat conditions across the DPS. However, the majority of populations with high hatchery influence are in upper reaches of the Salmon River.

Several populations are subject to relatively ***high*** exposures to *other stressors*, including water withdrawals, riparian and stream habitat degradation, etc. Water withdrawals in some populations have resulted in reduced flows that impact spawning/rearing habitat and downstream migration/rearing corridors. Altered stream and/or riparian habitats, including loss of side-channel rearing areas, are key impacts affecting all major population groups. These factors could have significant impacts to more than one-half of extant populations.

Adaptive Capacity

Snake River Basin steelhead ranked ***moderate*** for adaptive capacity overall, but there was a large number of low scores. This DPS could potentially shift its adult return and upstream migration timing to avoid peak temperatures in late summer, but such a shift may lead to increased negative effects from lower flows. For populations in high temperature or low flow areas, there are limited opportunities to shift juvenile rearing patterns to avoid climate change effects.

Literature Cited

Abdul-Aziz, O. I., N. J. Mantua, and K. W. Myers. 2011. Potential climate change impacts on thermal habitats of Pacific salmon (*Oncorhynchus spp.*) in the North Pacific Ocean and adjacent seas. Can. J. Fish. Aquat. Sci. 68:1660-1680.

Busby, P. J., T. C. Wainwright, G. J. Bryant, L. J. Lierheimer, R. S. Waples, F. W. Waknitz, and I. V. Lagomarsino. 1996. Status review of west coast steelhead from Washington, Idaho, Oregon, and California. U.S. Dep. Commerce NOAA Tech. Memo. NMFS-NWFSC-27.

Copeland, T., M. W. Ackerman, K. K. Wright, and A. Byrne. 2017. Life history diversity of Snake River steelhead populations between and within management categories. N. Am. J. Fish. Manage. 37:395-404.

Daly, E. A., R. D. Scheurer, R. D. Brodeur, L. A. Weitkamp, B. R. Beckman, and J. A. Miller. 2014. Juvenile steelhead distribution, migration, feeding, and growth in the Columbia River estuary, plume, and coastal waters. Mar. Coast. Fish. 6:62-80.

Evans, A. F., N. J. Hostetter, D. D. Roby, K. Collis, D. E. Lyons, B. P. Sandford, R. D. Ledgerwood, and S. Sebring. 2012. Systemwide evaluation of avian predation on juvenile salmonids from the Columbia River based on recoveries of passive integrated transponder tags. Trans. Am. Fish. Soc. 141:975-989.

Evans, A. F., Q. Payton, A. Turecek, B. Cramer, K. Collis, D. D. Roby, P. J. Loschl, L. Sullivan, J. Skalski, M. Weiland, and C. Dotson. 2016. Avian pedation on jvenile slmonids: satial and tmporal aalysis bsed on aoustic and passive integrated transponder tags. Trans. Am. Fish. Soc. 145:860-877.

Ford, M. J., K. Barnas, T. Cooney, L. G. Crozier, M. Diaz, J. J. Hard, E. E. Holmes, D. M. Holzer, R. G. Kope, P. W. Lawson, M. Liermann, J. M. Myers, M. Rowse, D. J. Teel, D. M. V. Doornik, T. C. Wainwright, L. A. Weitkamp, and M. Williams. 2015. Status review update for Pacific salmon and steelhead listed under the Endangered Species Act: Pacific Northwest. National Marine Fisheries Service, Northwest Fisheries Science Center, Seattle. Available from www.nwfsc.noaa.gov/publications/scipubs/searchdoc.cfm.

Fresh, K. L., L. L. Casillas, L. L. Johnson, and D. L. Bottom. 2005. Role of the estuary in the recovery of Columbia River basin salmon and steelhead: an evaluation of the effects of selected factors on salmonid population viability. U.S. Dep. Commerce NOAA Tech. Memo. NMFS-NWFSC-69.

Keefer, M. L., C. T. Boggs, C. A. Peery, and C. C. Caudill. 2008. Overwintering distribution, behavior, and survival of adult summer steelhead: Variability among Columbia river populations. N. Am. J. Fish. Manage. 28:81-96.

Kendall, N. W., G. W. Marston, and M. M. Klungle. 2017. Declining patterns of Pacific Northwest steelhead trout (*Oncorhynchus mykiss*) adult abundance and smolt survival in the ocean. Can. J. Fish. Aquat. Sci 74:1275-1290.

Light, J. T., C. K. Harris, and R. L. Burgner. 1989. Ocean distribution and migration of steelhead (*Oncorhynchus mykiss*, formerly *Salmo gairdneri*). Fisheries Research Institute, University of Washington, 8912, Seattle, WA.

Myers, K. W. 2018. Ocean ecology of steelhead. Page 1090 *in* R. J. Beamish, editor. The ocean ecology of Pacific salmon and trout. American Fisheries Society, Bethesda.

Robards, M. D., and T. P. Quinn. 2002. The migratory timing of adult summer-run steelhead in the Columbia River over six decades of environmental change. Trans. Am. Fish. Soc. 131:523-536.

Van Doornik, D., D. Kuligowski, C. A. Morgan, B. Burke, and T. R. Seamons. 2019. Insights, from genetic analyses, into stock-specific distribution of juvenile steelhead (*Oncorhynchus mykiss*) from the Columbia River during early marine migration. Fish. Bull. NOAA. 117:97-106

Wade, A., T. Beechie, E. Fleishman, N. Mantua, H. Wu, J. Kimball, D. M. Stoms, and J. Stanford. 2013. Steelhead vulnerability to climate change in the Pacific Northwest. J. Appl. Ecol. 50:1093-1104.

Weitkamp, L. A., D. J. Teel, M. Liermann, S. A. Hinton, D. M. Van Doornik, and P. J. Bentley. 2015. Stock-specific size and timing at ocean entry of Columbia River juvenile Chinook salmon and steelhead: Implications for early ocean growth. Mar. Coast. Fish. 7:370-392.

Upper Columbia River steelhead

Overall vulnerability—High (1% Moderate, 98% High, 1% Very high)
Biological sensitivity—High (1% Moderate, 99% High)
Climate exposure—High (99% High, 1% Very high)
Adaptive capacity—Moderate (1.8)
Data quality—84% of scores ≥ 2

	Upper Columbia River Steelhead	Expert Scores	Data Quality	Expert Scores Plots (Portion by Category)
Sensitivity attributes	Early life history	1.5	2.8	
	Juvenile freshwater stage	2.1	2.8	
	Estuary stage	1.6	2.3	
	Marine stage	2	2	
	Adult freshwater stage	3	2.5	
	Cumulative life-cycle effects	3	2	
	Hatchery influence	3.3	2.5	
	Other stressors	3.2	2.5	
	Population viability	3.1	2.8	
	Ocean acidification sensitivity	1.9	1.8	
	Sensitivity Score	High		
Exposure variables	Stream temperature	3.4	2.5	
	Summer water deficit	2.8	2	
	Flooding	2.5	2	
	Hydrologic regime	2.6	2.8	
	Sea level rise	1.4	2	
	Sea surface temperature	3.2	2.3	
	Ocean acidification exposure	3.9	3	
	Upwelling	1.6	1.8	
	Ocean currents	1.8	1	
	Exposure Score	High		
Overall Vulnerability Rank		High		

Low
Moderate
High
Very High

Life History Synopsis

The majority of Upper Columbia River steelhead adults enter fresh water and migrate upstream from July through October, with the migration generally peaking in August and September. Adults from all populations overwinter in the Columbia River or in its major tributaries upstream from Priest Rapids and Rock Island Dam. They complete migration the following spring. Spawning tributaries are 800-900 km upstream from the river mouth, and spawning occurs from March through June. Depending on spawn timing and temperature, fry emerge from the gravel in June and July, although emergence may be as late as September in upper-elevation tributaries due to cool temperatures.

Age at juvenile migration varies among populations in this DPS, and migration timing is generally correlated with elevation and annual stream temperatures. Most juveniles migrate at age-2 and age-3, although smolts ranging from age-1 to age-7 have been observed (Busby et al. 1996).

Smolts migrate through the estuary relatively rapidly in late spring, with a very short window of exposure to estuarine habitats (Fresh et al. 2005; Weitkamp et al. 2015). However, avian predation imposes significant mortality on juvenile steelhead moving through the estuary (Evans et al. 2012, 2016). Once in the ocean, Columbia River steelhead generally exhibits a rapid westward movement with an offshore marine distribution across the subarctic Pacific Ocean (Light et al. 1989; Daly et al. 2014; Myers 2018; Van Doornik et al. 2019).

Climate Effects on Abundance and Distribution

Upper Columbia River steelhead ranked ***high*** overall in sensitivity attributes, in part because of its ***high*** score for the *adult freshwater stage*. Populations encounter peak summer temperatures during adult migration and pre-spawn holding (Wade et al. 2013). This DPS also ranked high in exposure to *stream temperature*, reflecting its high vulnerability at multiple life stages and contributing to its ***high*** ranking for *cumulative life cycle effects*.

Although detailed information on the ocean distribution of Columbia River steelhead is not available, past studies suggest that steelhead from Pacific coastal systems generally occur in the Gulf of Alaska and in subarctic waters south of the Aleutian Islands (Light et al. 1989). Abdul-Aziz et al. (2011) developed spatially explicit representations of open ocean thermal habitat for steelhead. They found that under a multimodel ensemble average of climate model outputs using the A1B emissions scenario, summer habitat area declined by 36% for the 2080s, with the largest losses in the northeast Pacific Ocean. Wintertime habitat area losses were 2%, with reductions at the southern end of the historical range largely offset by gains in the Bering Sea and Sea of Okhotsk.

Whether a general northward and westward displacement of the most frequently observed thermal open ocean habitat will have substantial impacts on the life-cycle, productivity, or spawning distribution of these steelhead is not known. A recent study of smolt-to-adult survival trends found similarities in patterns of annual marine survival for steelhead stocks, with regional groupings for Puget Sound, British Columbia, and coastal Washington and Oregon (Kendall et al. 2017). Such patterns suggest that marine/estuarine factors associated with the point of ocean entry may be a more important determinants of year class survival for steelhead than general conditions in the adult ocean range.

Upper Columbia River steelhead ranked ***moderate*** to ***high*** in exposure to *flooding*, *stream temperature*, and *summer water deficit*. However, sensitivity at the *early life history* and *juvenile freshwater stage* was rated ***low*** because at present, stream temperatures and flows are well within tolerance limits. Sensitivities were ***low*** to ***moderate*** at the *estuary stage*. Exposure was ***high*** for *sea surface temperature* and ***low*** for *ocean currents*, *upwelling*, and *sea level rise*. Sensitivity was ***moderate*** at the *marine stage*.

Extrinsic Factors

Upper Columbia River steelhead scored ***high*** in all three extrinsic sensitivity attributes (*population viability*, *hatchery influence*, and *other stressors*). This DPS is listed as threatened under the U.S. Endangered Species Act, similar to other interior Columbia steelhead. However, Upper Columbia River steelhead is at higher risk than other northwest steelhead, and was reported to be in danger of extinction in a prior status review. Spatial diversity is low because all extant populations are in a single population group.

Large-scale mitigation hatchery programs are operating in three of the four tributaries that sustain natural production of this DPS, with ongoing straying into the fourth tributary (Entiat River). Proportions of hatchery adults in natural spawning areas of the Wenatchee River have declined in recent years due to relative increases in natural production, changes in hatchery release levels and locations, and (possibly) changes in broodstock practices aimed at increasing natural characteristics. In the Methow and Okanogan Rivers, proportions of hatchery spawners in natural spawning areas remain very high, although efforts have been initiated to evaluate relative reproductive success as well as release and broodstock strategies.

Altered stream and riparian habitats, including loss of side-channel rearing areas, are key impacts affecting all populations, especially in downstream reaches. Water withdrawals result in reduced flows that impact spawning and rearing habitats, especially for the Methow and Okanogan populations, as well as downstream migration corridors.

Adaptive Capacity

Upper Columbia River steelhead ranked ***moderate*** for adaptive capacity overall. This DPS may have some latitude to shift timing of adult migrations to avoid peak late summer temperatures (Robards and Quinn 2002), but the consequences of such timing shifts are not known. In each river population, individuals occupying the mid-to-lower reaches are subject to annual high stream temperatures and summer water deficits, and there are limited opportunities to shift juvenile rearing patterns. Anadromous *O. mykiss* may have some opportunities to expand summer rearing and overwintering to habitat areas upstream, but the amount of suitable habitat is limited compared to the potential loss of habitat in downstream reaches.

Literature Cited

Abdul-Aziz, O. I., N. J. Mantua, and K. W. Myers. 2011. Potential climate change impacts on thermal habitats of Pacific salmon (*Oncorhynchus spp.*) in the North Pacific Ocean and adjacent seas. Can. J. Fish. Aquat. Sci. 68:1660-1680.

Busby, P. J., T. C. Wainwright, G. J. Bryant, L. J. Lierheimer, R. S. Waples, F. W. Waknitz, and I. V. Lagomarsino. 1996. Status review of west coast steelhead from Washington, Idaho, Oregon, and California. U.S. Dep. Commerce NOAA Tech. Memo. NMFS-NWFSC-27.

Daly, E. A., R. D. Scheurer, R. D. Brodeur, L. A. Weitkamp, B. R. Beckman, and J. A. Miller. 2014. Juvenile steelhead distribution, migration, feeding, and growth in the Columbia River estuary, plume, and coastal waters. Mar. Coast. Fish. 6:62-80.

Evans, A. F., N. J. Hostetter, D. D. Roby, K. Collis, D. E. Lyons, B. P. Sandford, R. D. Ledgerwood, and S. Sebring. 2012. Systemwide evaluation of avian predation on juvenile salmonids from the Columbia River based on recoveries of passive integrated transponder tags. Trans. Am. Fish. Soc. 141:975-989.

Evans, A. F., Q. Payton, A. Turecek, B. Cramer, K. Collis, D. D. Roby, P. J. Loschl, L. Sullivan, J. Skalski, M. Weiland, and C. Dotson. 2016. Avian pedation on jvenile slmonids: satial and tmporal aalysis bsed on aoustic and passive integrated transponder tags. Trans. Am. Fish. Soc. 145:860-877.

Fresh, K. L., L. L. Casillas, L. L. Johnson, and D. L. Bottom. 2005. Role of the estuary in the recovery of Columbia River basin salmon and steelhead: an evaluation of the effects of selected factors on salmonid population viability. U.S. Dep. Commerce NOAA Tech. Memo. NMFS-NWFSC-69.

Kendall, N. W., G. W. Marston, and M. M. Klungle. 2017. Declining patterns of Pacific Northwest steelhead trout (*Oncorhynchus mykiss*) adult abundance and smolt survival in the ocean. Can. J. Fish. Aquat. Sci. 74:1275-1290.

Light, J. T., C. K. Harris, and R. L. Burgner. 1989. Ocean distribution and migration of steelhead (*Oncorhynchus mykiss*, formerly *Salmo gairdneri*). Fisheries Research Institute, University of Washington, 8912, Seattle, WA.

Myers, K. W. 2018. Ocean ecology of steelhead. Page 1090 *in* R. J. Beamish, editor. The ocean ecology of Pacific salmon and trout. American Fisheries Society, Bethesda.

Robards, M. D., and T. P. Quinn. 2002. The migratory timing of adult summer-run steelhead in the Columbia River over six decades of environmental change. Trans. Am. Fish. Soc. 131:523-536.

Van Doornik, D., D. Kuligowski, C. A. Morgan, B. Burke, and T. R. Seamons. 2019. Insights, from genetic analyses, into stock-specific distribution of juvenile steelhead (*Oncorhynchus mykiss*) from the Columbia River during early marine migration. Fish. Bull. NOAA. 117:97-106

Wade, A., T. Beechie, E. Fleishman, N. Mantua, H. Wu, J. Kimball, D. M. Stoms, and J. Stanford. 2013. Steelhead vulnerability to climate change in the Pacific Northwest. J. Appl. Ecol. 50:1093-1104.

Weitkamp, L. A., D. J. Teel, M. Liermann, S. A. Hinton, D. M. Van Doornik, and P. J. Bentley. 2015. Stock-specific size and timing at ocean entry of Columbia River juvenile Chinook salmon and steelhead: Implications for early ocean growth. Mar. Coast. Fish. 7:370-392.

Puget Sound steelhead

Overall vulnerability—High (39% Moderate, 69% High)
Biological sensitivity—High (30% Moderate, 70% High)
Climate exposure—High (1% Moderate, 99% High)
Adaptive capacity—High (2.1)
Data quality—84% of scores ≥ 2

Puget Sound Steelhead		Expert Scores	Data Quality	Expert Scores Plots (Portion by Category)
Sensitivity attributes	Early life history	1.5	2.8	
	Juvenile freshwater stage	2.1	2.8	
	Estuary stage	1.7	2.3	
	Marine stage	2.7	2	
	Adult freshwater stage	1.7	2.8	
	Cumulative life-cycle effects	2.3	2	
	Hatchery influence	3.2	2.5	
	Other stressors	3.1	2.5	
	Population viability	2.7	2.5	
	Ocean acidification sensitivity	1.9	1.8	
	Sensitivity Score	High		
Exposure variables	Stream temperature	3	2.5	
	Summer water deficit	2.7	2	
	Flooding	3.1	2	
	Hydrologic regime	2.8	2.8	
	Sea level rise	1.4	2	
	Sea surface temperature	3.2	2.3	
	Ocean acidification exposure	3.9	3	
	Upwelling	1.5	1.8	
	Ocean currents	1.8	1	
	Exposure Score	High		
Overall Vulnerability Rank		High		

Low
Moderate
High
Very High

Life History Synopsis

Within the Puget Sound steelhead DPS, winter-run adults migrate from November to May, and summer-run adults from May to October. Migration distances in Puget Sound are relatively short (<150 km). Spawning occurs primarily in March and April, although it can start as early as January in lowland, rain-dominated areas, and peak as late as May or June in cooler, snow-dominated locations (Myers et al. 2015). In some locations there is a clear distinction between winter-run and summer-run spawning areas. For example, in basins with partially impassable barriers, winter migrants are blocked, and only the summer-run is successful. Conversely, in short, rain-dominated basins, most spawners are winter-run. However, there is also overlap in spawning areas and timing, where segregation of the two run types is not clear (Myers et al. 2015).

Survival through the incubation period is not well documented, but there are potential temperature effects for summer-run Puget Sound steelhead. Temperatures and flow levels during incubation are generally below maximum limits for most populations. Most juveniles migrate to Puget Sound at age 2. In the White, Nisqually, and Green Rivers, 16-20% of juvenile migrants are age 1, while in the Skagit, Snohomish, and Elwha Rivers, 15-27% are age 3. Puget Sound steelhead appears to have a relatively short estuary residence time, with juveniles moving to the Strait of Juan de Fuca within 1-3 weeks (Weitkamp et al. 2014). Ocean migrations are poorly understood, but limited data indicate that they travel to the central subarctic North Pacific Ocean (Burgner et al. 1992, Myers 2018). In general, North American steelhead undergo extensive ocean migrations into the Gulf of Alaska and subarctic North Pacific Ocean, areas with an absolute thermal range of 2.8-15.8°C for all seasons and a frequently observed range of 6-12.5°C from spring through fall and 5-11°C in winter (Abdul-Aziz et al. 2011).

There is a moderate amount of variation in age at maturity, but most individuals in this DPS spend 2 years at sea and typically mature after 1 to 3 years. Deer Creek summer-run steelhead spend one year at sea (Myers et al. 2015).

Climate Effects on Abundance and Distribution

Puget Sound steelhead rear in the sound year-round; thus, it ranked ***moderate*** in exposure to *summer water deficit* and ***high*** in exposure to *stream temperature* and *flooding* (see also Wade et al. 2013). All three freshwater life stages were scored ***low*** to ***moderate*** in sensitivity. However, because spawning typically occurs after the peak storm season, this DPS received a lower sensitivity score for *early life history* (egg stage) compared with Puget Sound Chinook and coho. Winter-run adults are not sensitive to summer stream temperature or low flows during migration (Beechie et al. 2013). However, the summer run may have higher sensitivity to the extent that partially impassable barriers constrain their migration timing. The relatively short juvenile migration distances reduce dependence on the size of the spring freshet, so that these juveniles are less sensitive to changes in snowpack than those from interior steelhead DPSs.

Abdul-Aziz et al. (2011) developed spatially explicit representations of open ocean thermal habitat for steelhead. They found that under a multimodel ensemble average of climate model outputs using the A1B emissions scenario summer, habitat area declined by 36% for the 2080s, with the largest losses in the northeast Pacific Ocean. Wintertime habitat area losses were 2%, with reductions at the southern end of the historical range largely offset by gains in the Bering Sea and Sea of Okhotsk.

Whether a general northward and westward displacement of the most frequently observed thermal open ocean habitat will have substantial impacts on the life-cycle, productivity, or spawning distribution of this DPS is not known. However, West Coast steelhead salmon populations may be vulnerable to the projected displacement of high

seas thermal habitat. Puget Sound steelhead ranked ***high*** for exposure to *sea surface temperature*, and ***moderate*** in sensitivity at the *marine stage*.

Extrinsic Factors

Puget Sound steelhead is listed as threatened under the U.S. Endangered Species Act, and therefore ranked ***moderate*** for sensitivity to *population viability*. All extant and viable summer-run populations are in the North Cascades population group, indicating a relatively limited spatial distribution. Habitat fragmentation (Hard et al. 2015) led to a ***high*** rank in the *other stressors* category. Outplanting of hatchery fish has historically been widespread in Puget Sound, and this DPS ranked ***high*** in sensitivity to *hatchery influence*. Most Puget Sound DPSs had low scores for diversity and spatial structure, largely because of extensive hatchery influence, breeding populations, and freshwater habitat fragmentation or loss. There has been some loss of accessible habitat above dams in some river basins (Hard et al. 2015).

Adaptive Capacity

Puget Sound steelhead ranked ***high*** for adaptive capacity. Steelhead has a very flexible life history that is responsive to environmental conditions. If growth conditions change, these fish are relatively capable of changing the age at which they smolt. Adults migrate across a wide temporal window even to the same spawning areas, and thus this DPS likely has the flexibility to change migration timing if needed. Nonetheless, some habitat locations with partially impassable barriers may become inaccessible due to altered temperatures and flows.

Literature Cited

Abdul-Aziz, O. I., N. J. Mantua, and K. W. Myers. 2011. Potential climate change impacts on thermal habitats of Pacific salmon (*Oncorhynchus spp.*) in the North Pacific Ocean and adjacent seas. Can. J. Fish. Aquat. Sci. 68:1660-1680.

Beechie, T., H. Imaki, J. Greene, A. Wade, H. Wu, G. Pess, P. Roni, J. Kimball, J. Stanford, P. Kiffney, and N. Mantua. 2013. Restoring salmon habitat for a changing climate. River Res. Appl. 29:939-960.

Burgner, R. L., J. T. Light, L. Margolis, T. Okazaki, A. Tautz, and S. Ito. 1992. Distribution and origins of steelhead trout (*Oncorhynchus mykiss*) in offshore waters of the North Pacific Ocean. Int. N. Pac. Fish. Comm. Bull. 51

Hard, J. J., J. M. Myers, E. J. Conner, R. A. Hayman, R. G. Kope, G. Lucchetti, A. R. Marshall, G. R. Pess, and B. E. Thomspson. 2015. Viability criteria for steelhead within the Puget Sound distinct population segment. U.S. Dep. Commerce NOAA Tech. Memo. NMFS-NWFSC-129.

Myers, J. M., J. J. Hard, E. J. Connor, R. A. Hayman, R. G. Kope, A. Luchetti, A. R. Marshall, G. Pess, and B. E. Thompson. 2015. Identifying historical populations of steelhead within the Puget Sound distinct population segment. U.S. Dep. Commerce NOAA Tech. Memo. NMFS-NWFSC-128.

Myers, K. W. 2018. Ocean ecology of steelhead. Page 1090 *in* R. J. Beamish, editor. The ocean ecology of Pacific salmon and trout. American Fisheries Society, Bethesda.

Wade, A., T. Beechie, E. Fleishman, N. Mantua, H. Wu, J. Kimball, D. M. Stoms, and J. Stanford. 2013. Steelhead vulnerability to climate change in the Pacific Northwest. J. Appl. Ecol. 50:1093-1104.

Weitkamp, L. A., G. Goulette, J. Hawkes, M. O'Malley, and C. Lipsky. 2014. Juvenile salmon in estuaries: comparisons between North American Atlantic and Pacific salmon populations. Rev. Fish Biol. Fish. 24:713-736.
