# Supplementary figures and images for: Climate vulnerability assessment for Pacific salmon and steelhead in the California Current Large Marine Ecosystem
Source: PLoS One. 2019 Jul 24;14(7):e0217711. doi: 10.1371/journal.pone.0217711 (PMC6655584; doi:10.1371/journal.pone.0217711)

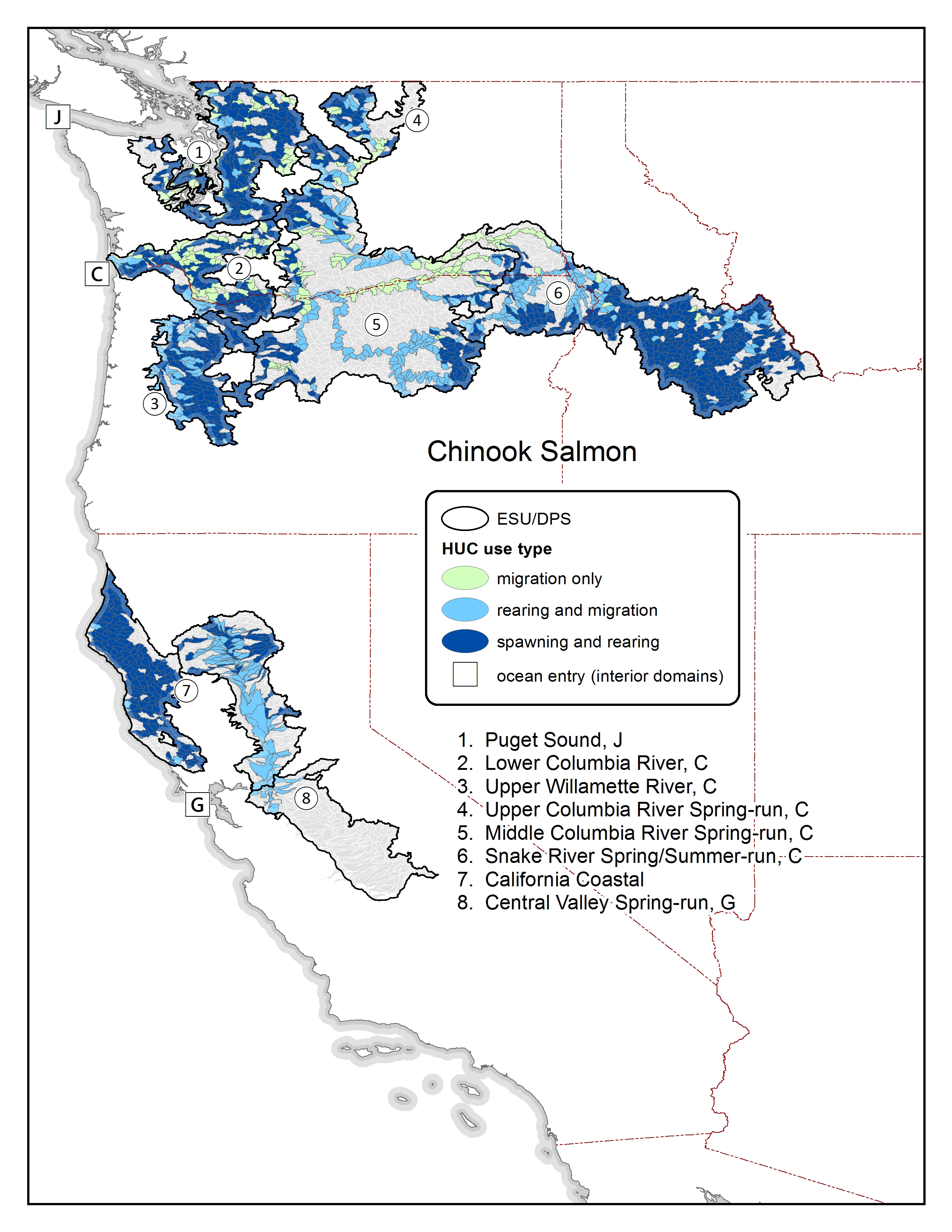

Supplement: S1 Fig — (JPG) [file pone.0217711.s007.jpg]

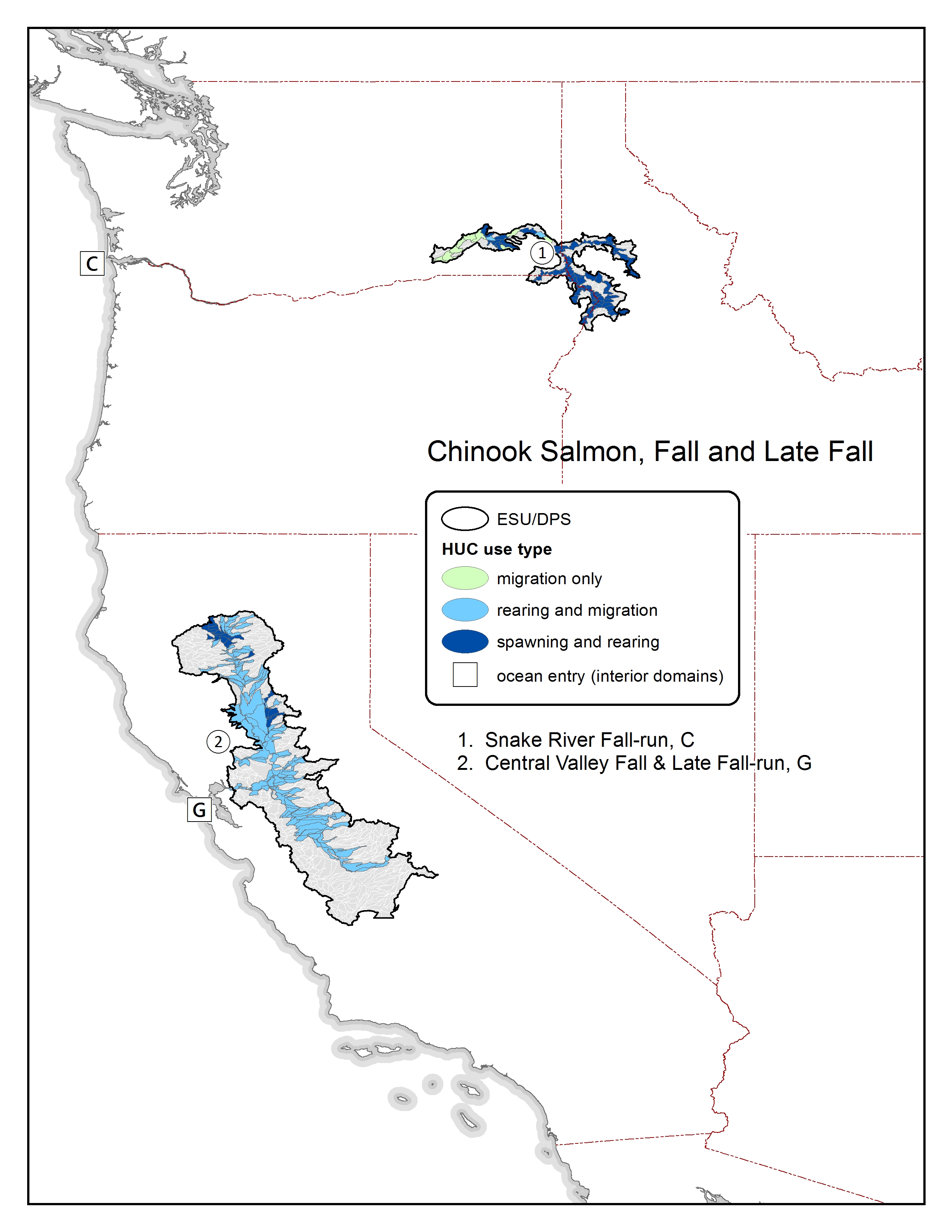

Supplement: S2 Fig — (JPG) [file pone.0217711.s008.jpg]

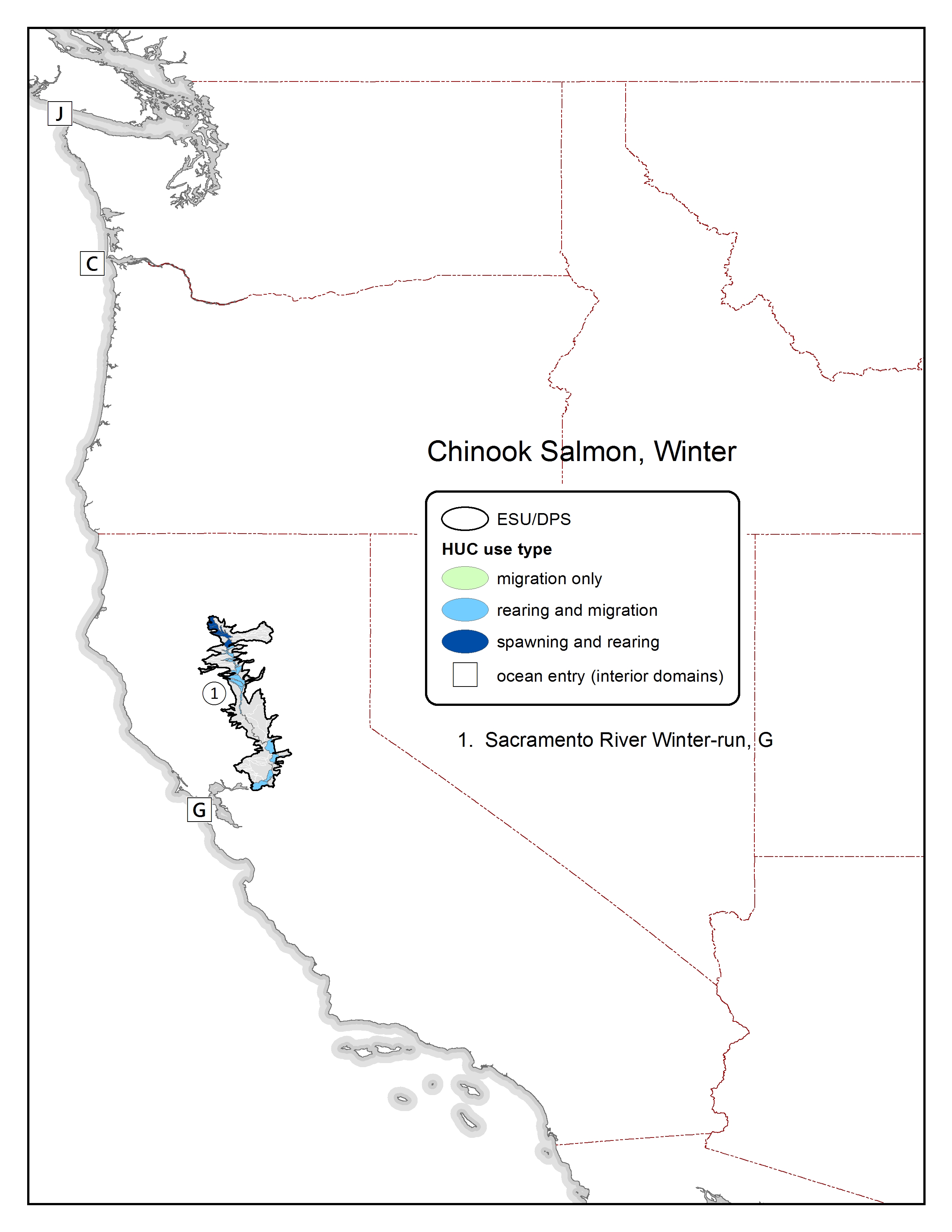

Supplement: S3 Fig — (JPG) [file pone.0217711.s009.jpg]

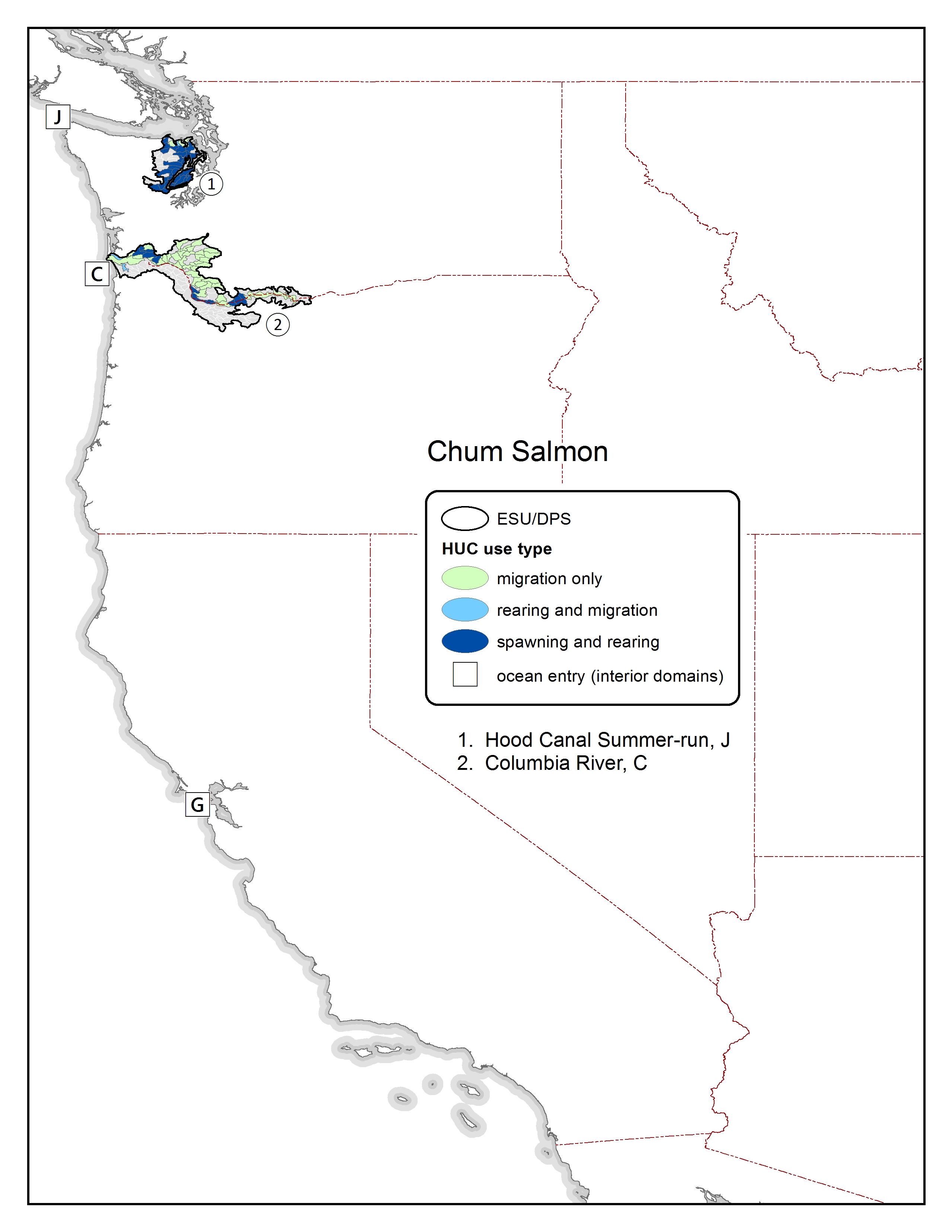

Supplement: S4 Fig — (JPG) [file pone.0217711.s010.jpg]

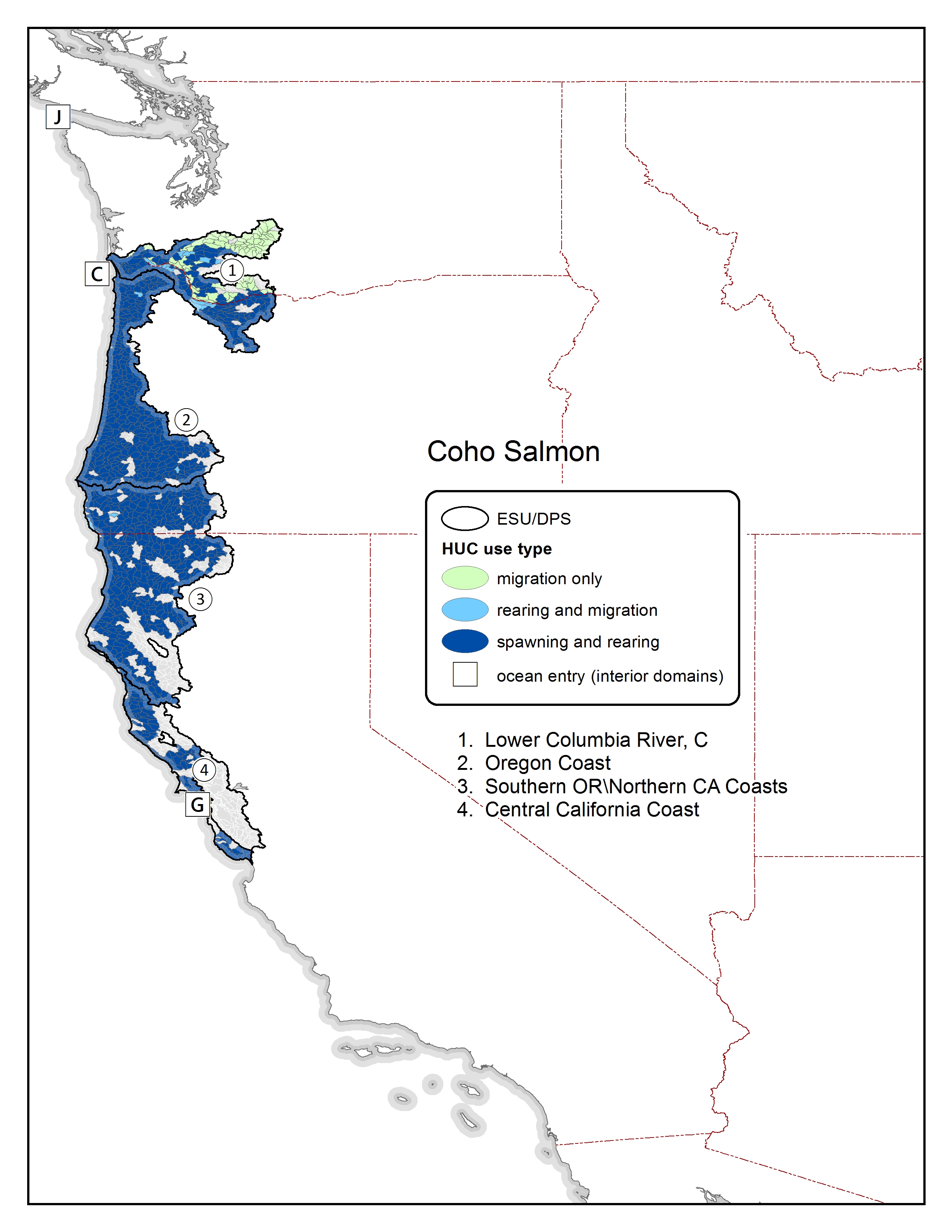

Supplement: S5 Fig — (JPG) [file pone.0217711.s011.jpg]

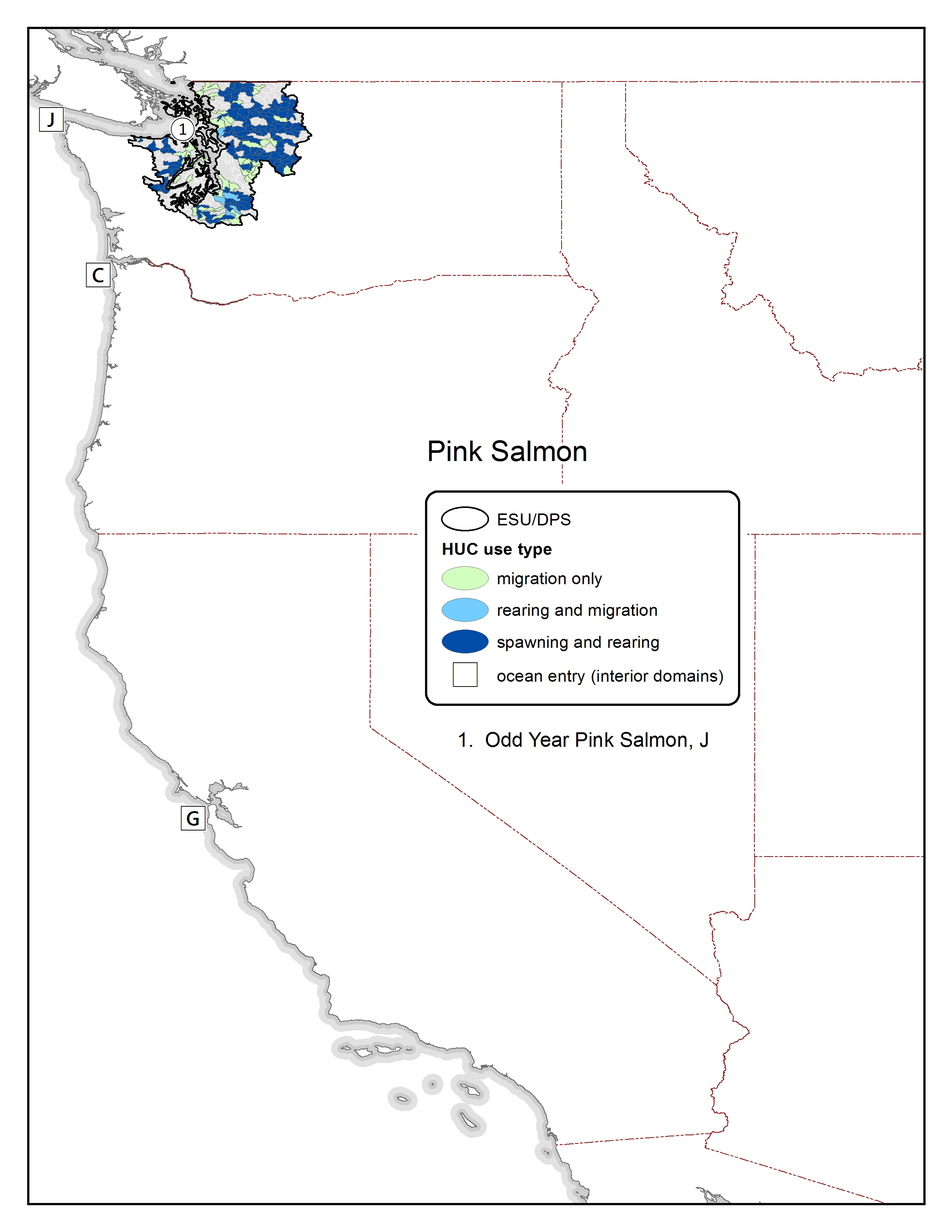

Supplement: S6 Fig — (JPG) [file pone.0217711.s012.jpg]

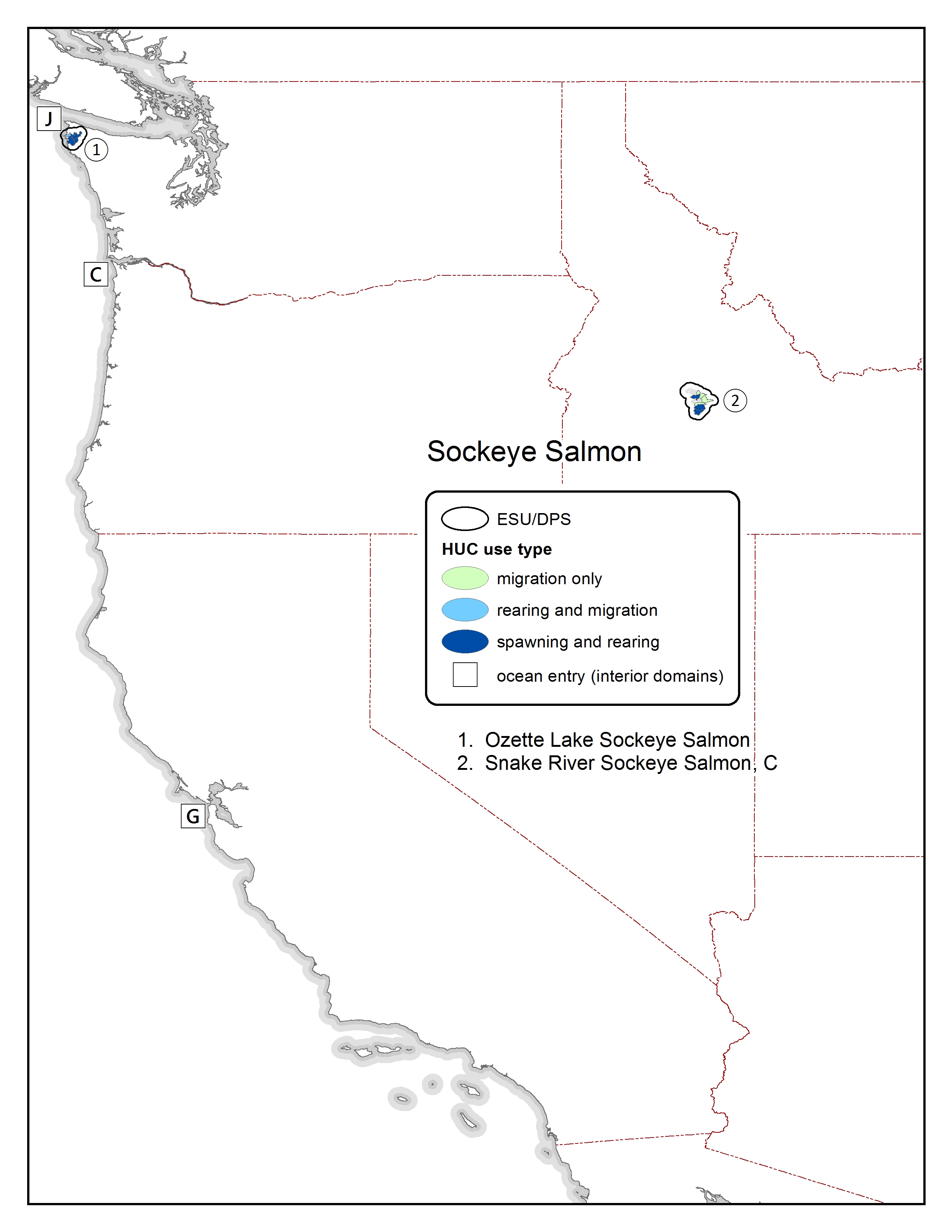

Supplement: S7 Fig — (JPG) [file pone.0217711.s013.jpg]

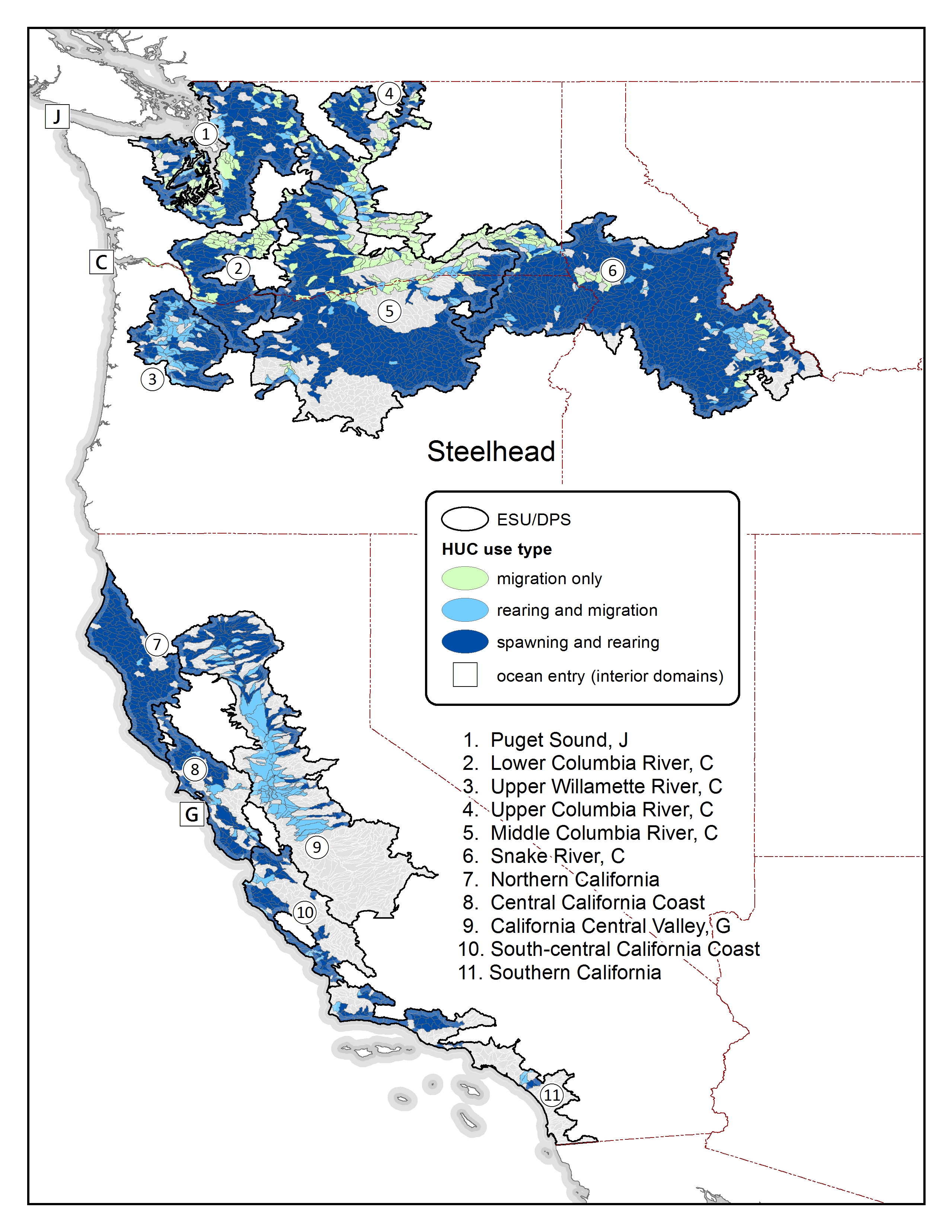

Supplement: S8 Fig — (JPG) [file pone.0217711.s014.jpg]
