# Supplementary material for: Climate vulnerability assessment for Pacific salmon and steelhead in the California Current Large Marine Ecosystem
Source: PLoS One. 2019 Jul 24;14(7):e0217711. doi: 10.1371/journal.pone.0217711 (PMC6655584; doi:10.1371/journal.pone.0217711)
Supplement: S9 Fig — (PDF) [file pone.0217711.s015.pdf]

S9 Fig. Classification and regression tree results

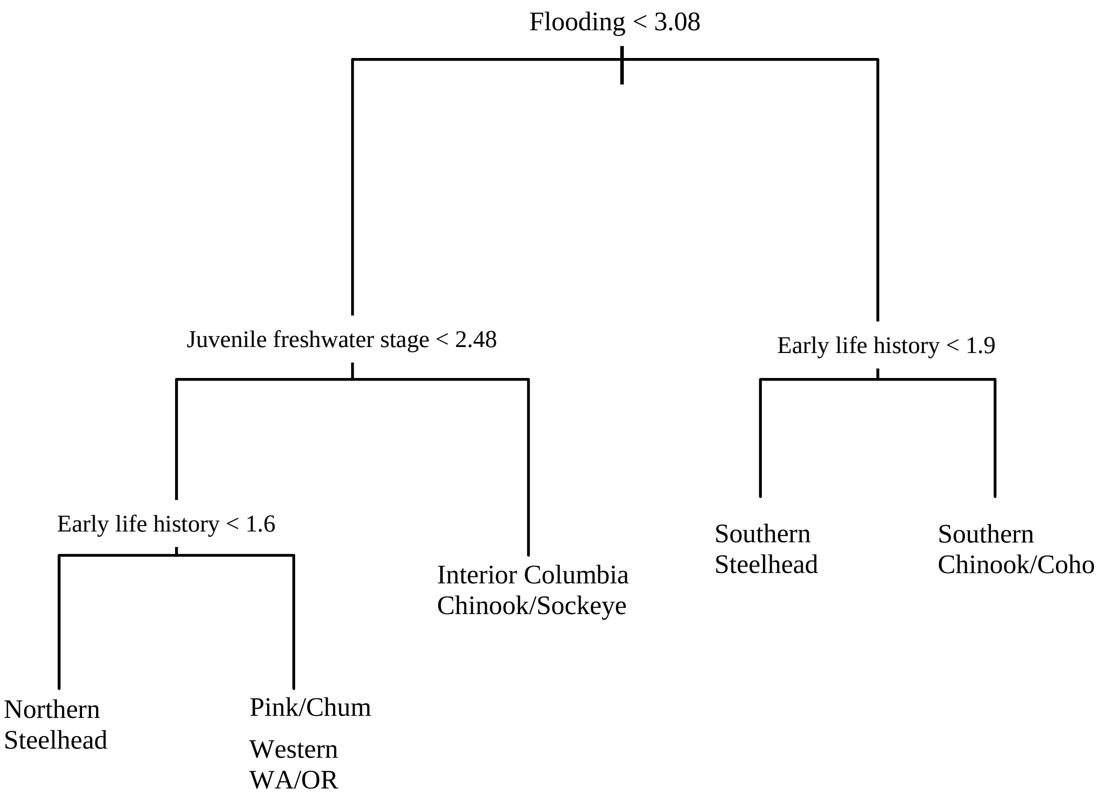

S4 Appendix. Classification and regression tree results showing factors that were sufficient to differentiate clusters. Additional factors that differed among groups are shown in Table 4.
