# Supplementary material for: Climate vulnerability assessment for Pacific salmon and steelhead in the California Current Large Marine Ecosystem
Source: PLoS One. 2019 Jul 24;14(7):e0217711. doi: 10.1371/journal.pone.0217711 (PMC6655584; doi:10.1371/journal.pone.0217711)
Supplement: S10 Fig — (DOCX) [file pone.0217711.s016.docx]

# S10 Fig. Data quality scores and standard deviation of scores

**S5. Data quality scores and standard deviation of tallies for each attribute**.

The median (horizontal line), interquartile range (boxes) and 1.5 times the interquartile range (whiskers) across DPSs. Attributes associated with freshwater stages are green, marine stages are blue, extrinsic sensitivity metrics are grey for both data quality scores (top) and the standard deviation in the scores across experts (bottom).
